# Generic revision and species classification of the Microdontinae (Diptera, Syrphidae)

**DOI:** 10.3897/zookeys.288.4095

**Published:** 2013-04-12

**Authors:** Menno Reemer, Gunilla Ståhls

**Affiliations:** 1 Naturalis Biodiversity Center, European Invertebrate Survey – the Netherlands, P.O. Box 9517, 2300 RA Leiden, the Netherlands; 2 Finnish Museum of Natural History, Entomology dept., P.O. Box 17, FIN-00014 University of Helsinki, Finland

**Keywords:** Key, revision, new genera, new species, new synonyms, new combinations, catalogue

## Abstract

With 552 species group names available (excluding misspellings), the Microdontinae constitute the smallest of the three subfamilies of Syrphidae. Paradoxically, this subfamily is taxonomically the least organized of the three: 388 species names were previously classified in a single genus, *Microdon* Meigen, 1803. The present paper introduces a new generic classification of the Microdontinae, relying partly on the results of phylogenetic analyses of morphological and molecular data as published in other papers, and partly on examination of primary type specimens of 347 taxa, plus additional material, and original descriptions. A total number of 67 genus group names (excluding misspellings) are evaluated, redescribed, diagnosed and discussed, with several implications for their taxonomic status. Of these, 43 names are considered as valid genera, 7 as subgenera, 17 as synonyms. Two generic names (*Ceratoconcha* Simroth, 1907, *Nothomicrodon* Wheeler, 1924) are left unplaced, because they are known from immature stages only and cannot be reliably associated with taxa known from adults. The following 10 new genera are described by Reemer: *Domodon*, *Heliodon*, *Laetodon*, *Menidon*, *Mermerizon*, *Metadon*, *Peradon*, *Piruwa*, *Sulcodon* and *Thompsodon*. A key to all genera, subgenera and species groups is given. A total number of 26 new species are described in the following genera: *Archimicrodon* Hull, 1945, *Ceratrichomyia* Séguy, 1951, *Domodon*, *Furcantenna* Cheng, 2008, *Heliodon*, *Indascia* Keiser, 1958, *Kryptopyga* Hull, 1944, *Masarygus* Brèthes. 1908, *Mermerizon*, *Metadon*, *Microdon*, *Paramixogaster* Brunetti, 1923, *Piruwa*, *Pseudomicrodon* Hull, 1937, *Rhopalosyrphus* Giglio-Tos, 1891, and *Thompsodon*. New lectotypes are designated for *Ceratrichomyia behara* Séguy, 1951 and *Microdon iheringi* Bezzi, 1910. A total number of 267 new combinations of species and genera are proposed. New synonyms are proposed for 19 species group names. Three replacement names are introduced for primary and secondary junior homonyms: *Microdon shirakii* **nom. n.** (= *Microdon tuberculatus* Shiraki, 1968, primary homonym of *Microdon tuberculatus* de Meijere, 1913), *Paramixogaster brunettii* **nom. n.** (= *Mixogaster vespiformis* Brunetti, 1913, secondary homonym of *Microdon vespiformis* de Meijere, 1908), *Paramixogaster sacki* **nom. n.** (= *Myxogaster variegata* Sack, 1922, secondary homonym of *Ceratophya variegata* Walker, 1852). An attempt is made to classify all available species names into (sub)genera and species groups. The resulting classification comprises 454 valid species and 98 synonyms (excluding misspellings), of which 17 valid names and three synonyms are left unplaced. The paper concludes with a discussion on diagnostic characters of Microdontinae.

## Introduction

The Microdontinae (Diptera: Syrphidae) are found on all continents except Antarctica. The vast majority of more than 400 described species occurs in the tropics, of which almost half in the Neotropics. With little more than 50 species known from the Holarctic region, the group is relatively poorly represented in temperate regions. This partly explains why the taxonomy of the group has so far received little attention compared to other Syrphidae. This can also be explained by the morphological variation within the Microdontinae, which is arguably larger than in many families of Diptera Cyclorrhapha. Several authors have commented on the group’s paradoxical combination of a wealth of morphological diversity at the species level and a scarceness of group-defining characters (Bezzi 1915, Curran 1941, Shannon 1927). As a result, more than 300 out of approximately 400 valid species names are classified currently in the single genus *Microdon* Meigen, 1803. This apparent taxonomic indecisiveness seems to result not so much from a lack of morphological variation, but rather from an excess of it.

The classification of taxa, generic as well as specific, within the Microdontinae is the subject of the present paper. All available generic taxa of Microdontinae, as well as many species, are studied and compared in detail. Although phylogenetic relationships are still unclear for many taxa, we prefer to employ an ‘old-fashioned’ method of classification based on detailed comparative morphology over a ‘waste basket’ approach, despite their morphological differences (for more on this see Procedure under Material and Methods). A first phylogenetic analysis of the group is in press (Reemer and Ståhls in press), and in a number of instances the results of that study will be referred to.

### Classification of Microdontinae within Syrphidae

When Meigen (1803) introduced the generic name *Microdon*, there was no intrafamilial classification of the family Syrphidae. The first family group name proposed for *Microdon* and its allies was Aphritadae Fleming, 1821 (spelled Aphritidae by Fleming 1822), separated from the ‘Syrphadae’ based on the absence of a facial tubercle. The Aphritidae also included *Milesia* Latreille, 1804 and related genera, which are nowadays included in the Eristalinae. Although the family group name Aphritidae has priority over Microdontinae, the latter name is maintained because Aphritidae has not been used after 1899, whereas Microdontinae has been used by many authors since (ICZN 1999: article 23.9, Sabrosky 1999).

Rondani (1845) first introduced the family group name Microdontinae (spelled as ‘Microdonellae’), based on the dentate scutellum of the type species *Microdon mutabilis* Linnaeus, 1758. Ever since, this group has been recognized as distinct from other Syrphidae, albeit under different spellings and taxonomic rankings. In early days (Lioy 1864, Brauer 1883, Williston 1886) and the single more recent case of Shatalkin (1975a, b), authors included genera which are nowadays considered to belong to other subfamilies. The placement of the group relative to other Syrphidae, however, has been far from stable. It would exceed the aim of the present paper to repeat here every author’s argumentations for their subsequent classifications over more than one and a half century. Table 1 lists the many different historical taxonomic treatments (spellings and classifications) the group has received.

**Table 1.** Chronological overview of spellings, classifications and rankings of the family group names Aphritadae Fleming, 1821 and Microdonellae Rondani, 1845. All known references introducing a novel spelling or classification are included, as well as all known works that explicitly deal with the classification of the group. Works merely using previously suggested classifications are omitted.

**Author**	**Name / spelling**	**Ranking and remarks**
Fleming 1821: 55	Aphritadae	Included *Milesia* Latreille and related genera.
Fleming 1822: 584	Aphritidae	See Fleming 1821.
Rondani 1845: 451	Microdonellae	One of eight ‘lineas’, equivalent to subfamilies.
Rondani 1856: 20, 54	Microdonina	One of seven lineages, equivalent to subfamilies.
Rondani 1857: 206	Microdoninae	See Rondani 1856.
Lioy 1864: 740	*Microdon* included in Psariti	One of five subdivisions of Syrphidae, equivalent to subfamilies, including genera *Chrysotoxum* Meigen, 1803 and *Psarus* Latreille, 1804.
Nowicki 1873: 24	Microdontina	One of eight subdivisions, equivalent to subfamlies.
Brauer 1883: 70	Microdinae	Equivalent to tribe within subfamily (‘Gruppe’) Chrysotoxinae, including genera *Chrysotoxum* Meigen, 1803, *Pipiza* Meigen, *Orthonevra* Macquart, 1829 among other.
Williston 1886: xvi	Microdonini	Tribe within subfamily Syrphinae, including genera *Chrysotoxum* Meigen, 1803 and *Psarus* Latreille, 1804.
Verrall 1901: 658	Microdontinae	One of seven subfamilies.
Shannon 1921: 67, 123; 1922: 35	Microdontinae	One of ten subfamilies.
Sack 1928-1932: 234	Microdontinae	One of 14 subfamilies.
Hull 1949: 305	Microdontinae	One of 14 subfamilies, related to Eumerinae and Nausigasterinae. *Spheginobaccha* included.
Goffe 1952: 112	Microdontina	Subtribe of tribe Volucellini, within subfamily Sphixinae (= Milesiinae of Wirth et al. 1965).
Wirth et al. 1965	Microdontini	Tribe within subfamily Milesiinae
Thompson 1969: 75	Microdontinae	*Spheginobaccha* excluded.
Thompson 1972: 85	Microdontidae	Family.
Shatalkin 1975a, b	Microdontinae	Subfamily. *Spheginobaccha* included, as well as *Alipumilio* Shannon, 1927 and *Nausigaster* Williston, 1884.
Speight 1987: 172	Microdontidae	Family.
Ståhls et al. 2003: 449	Microdontinae	Subfamily. *Spheginobaccha* included. *Alipumilio* and *Nausigaster* excluded.
Cheng and Thompson 2008: 21	Microdontinae	Subfamily.

The first to regard the Microdontinae as “presumably an old group early differentiated from the family” was Hull (1949). Goffe (1952) extensively reviewed the prior classifications of Syrphidae, including Microdontinae.

He placed the Microdontinae as a subtribe (‘Microdontina’) in the tribe Volucellini, together with the subtribe Volucellina, as part of the subfamily Sphixinae (more or less equivalent to the current Eristalinae). Thompson (1969) did not agree and treated the group again as basal within the Syrphidae. Then Thompson (1972) proposed to raise the group to family level. Shatalkin (1975a, b) did not follow this proposal, basing his argumentation only on the number of male pre-abdominal segments, but he agreed on the basal position of the group as a subfamily within the Syrphidae.

The proposal of Thompson (1972) to treat the Microdontinae as a separate family has not generally been followed. Speight (1987), however, based on his considerations of syrphid morphology, found *Microdon* to be aberrant from other Syrphidae to such an extent that he chose to follow Thompson’s proposal. In the study of Rotheray and Gilbert (1999), based on characters of immature stages, Microdontinae were placed as follows: (Eristalinae + (Microdontinae + (Syrphinae + Pipizini)). Subsequently, a number of studies recovered the Microdontinae as the sister-group of all other Syrphidae: Skevington and Yeates (2000) (based on molecular data), Ståhls et al. (2003) (based on molecular data combined with larval and adult morphology), and Rotheray and Gilbert (2008) (based on characters of the larval head). The results of Hippa and Ståhls (2005) (based on an extended set of adult morphological characters) differed from those previously mentioned by the placement of (*Neoascia* Williston, 1886 + *Sphegina* Meigen, 1822) as sister-group to all other Syrphidae. However, all of these authors treated the Microdontinae as a subfamily, as did Cheng and Thompson (2008). Speight (2010) has, however, continued to use familial rank. Reemer and Ståhls (in press), evaluating previous phylogenetic results as well as their newly generated evidence, see no scientific reason for changing the prevailing ranking of the Microdontinae and for that reason prefer nomenclatural stability.

### Classifications and phylogenetic relationships within Microdontinae

There have been few previous attempts to generate a tribal classification of Microdontinae. Apart from the names Aphritidae Fleming and Microdontinae Rondani (see previous paragraph), only three family-group names have been proposed: Masarygidae Brèthes, 1908, Ceratophyini Hull, 1949 and Spheginobacchini Thompson, 1972. See Reemer and Ståhls (in press) for discussion on availability of these names. Application of the first two names is at present considered undesirable, as most phylogenetic relationships at suprageneric level are still too uncertain to recognize tribes, due to limited availability of taxa for molecular phylogenetic analysis and the obtained low support values for most of the resolved larger clades (Reemer and Ståhls in press). The tribe Spheginobacchini is the only of these names that continues to be recognized here, as we consider the sister group relationship of this taxon to the remaining Microdontinae well enough established (Ståhls et al. 2003, Reemer and Ståhls in press).

**Figure 1.**
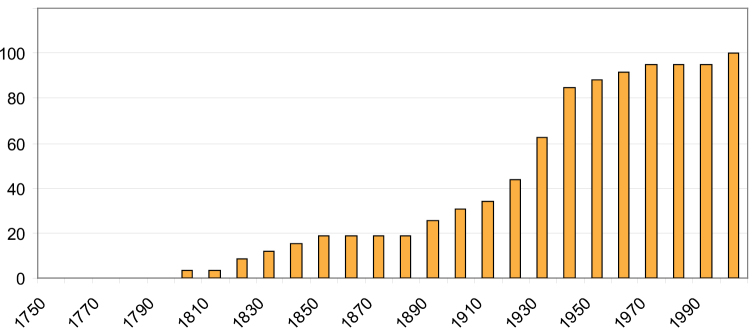
Cumulative graph of introduced genus-group names of Microdontinae per decade (as percentage of total number of 59).

Cheng and Thompson (2008) gave an extensive overview of generic names of Microdontinae, which formed the starting point for the present paper. Since Meigen (1803) introduced the name *Microdon*, 59 genus-group names applicable to Microdontinae have been introduced (misspellings excluded) (Fig. 1). This number increased most rapidly during the first half of the 20th century. Since then, only nine new genus-group names have been proposed.

**Figure 2.**
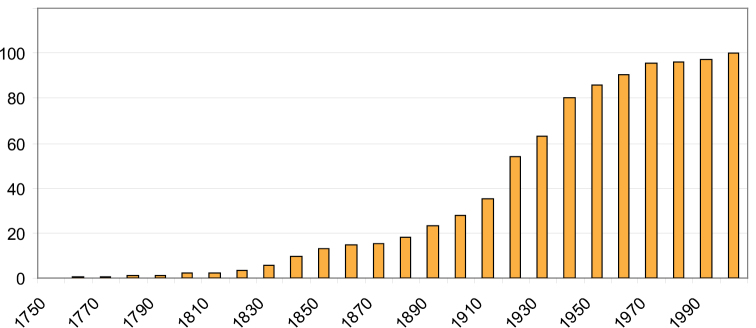
Cumulative graph of introduced species-group names of Microdontinae per decade (as percentage of total number of 514).

The number of previously introduced species-group names in Microdontinae is 514 (including synonyms and unvalid names). The cumulative graph of the number of species names per decade is similar to the one for genus-group names (Fig. 2). A majority of these species names (388) are currently classified into the genus *Microdon*. Most of the other (sub)genera contain only a few species. The very large genus *Microdon* thus constitutes one of the greatest taxonomic challenges of Syrphidae. The classification of so many species into one genus was a consequence of pragmaticism, as no comprehensive revisions were available.

## Material and methods

### Procedure

The phylogenetic results of Reemer and Ståhls (in press) are used as a first cue for the generic classification. When the evidence provided by these analyses is not conclusive or considered unconvincing (e.g. because of low support values), morphological characters are evaluated subjectively. Considerable weight is given to the structure of the male genitalia, but in all cases there are also external characters to support the groups. The species classification relies largely on this morphological evaluation.

Generally, a conservative approach is adopted towards changing the rank of taxa. Generic or subgeneric ranks as indicated by Cheng and Thompson (2008) are mostly maintained, unless these are contradicted by the results of the phylogenetic analyses of Reemer and Ståhls (in press). This is mainly relevant in the case of the genus *Microdon*. The species previously assigned to this genus were resolved as scattered over the phylogenetic trees of Reemer and Ståhls (in press). For some of these groups, genus group names are available, for some there are none. In several cases, genus group names that were previously treated as subgenera are now raised to generic level. In addition, new genus group names needed to be erected for several taxa that were previously included in *Microdon*. Given the uncertainties in the deeper branches of Microdontinae-phylogeny, these new group names could also have been given subgeneric rank within *Microdon*. However, this would suggest a close affinity with that genus, despite the fact that this is not indicated by the phylogenetic results. Moreover, we found it useful to split the genus *Microdon* into smaller natural groups which are more manageable than a genus containing more than 300 species. As this is only done for groups which have a high probability of being monophyletic (as indicated by phylogenetic results of Reemer and Ståhls (in press) or by subjective judgement of supposed synapomorphies), this procedure will facilitate further research on intergeneric phylogenetic relationships.

### Acronyms of collections

The following acronyms are used to indicate entomological collections.

**AMNH** American Museum of Natural History, New York

**AMS** Australian Museum, Sydney

**ANIC** Australian National Insect Collection, Canberra

**ANSP** Academy of Natural Sciences of Pennsylvania, Philadelphia

**BMNH** British Museum of Natural History, London

**CASB** Chinese Acadamy of Science, Bejing

**CM** Carnegie Museum, Pittsburgh

**CNC** Canadian National Collection, Ottawa

**CSCA** California State Collection of Athropods, Sacramento

**CSCS** Central South University of Forestry and Technology, Changsha, Hunan

**CU** Cornell University, Ithaca

**DEI** Deutsches Entomologisches Institut, Müncheberg

**DZUP** Departamento de Zoologia da Universidade Federal do Paraná, Curitiba

**HNHM** Hungarian Natural History Museum, Budapest

**INBIO** Instituto Nacional de Biodiversidad, Heredia

**MACN** Museo Argentino de Ciencias Naturales, Buenos Aires

**MCGD** Museo Civico di Storia Naturale ‘G. Doria’, Genova

**MCSN** Museo Civico di Storia Naturale, Milan

**MCZ** Museum of Comparative Zoology, Harvard

**MNHN** Muséum National d’Histoire Naturelle, Paris

**MRHNB** Musée Royal d’Histoire Naturelle de Belgique, Brussels

**MRSN** Museu Regionale di Scienze Naturali, Turin

**MZH** Finnish Museum of Natural History, Helsinki

**MZLU** Museum of Zoology Lund University, Lund

**MZM** Museum of Zoology, University of Michigan, Ann Arbor

**MZUN** Museo Zoologico di Università degli Studi, Naples

**MZUSP** Museu de Zoologia da Universidade de São Paulo, São Paulo

**NHRS** Naturhistoriska Riksmuseet, Stockholm

**NIAS** Laboratory of Insect Systematics, National Institute of Agro-Environmental Sciences, Kannondai

**NMB** Naturhistorisches Museum Basel, Basel

**NMSA** Natal Museum, Pietermaritzburg

**NMW** Naturhistorisches Museum Wien, Vienna

**NSMT** National Science Museum Tokyo, Tokyo

**NZCS** National Zoological Collection of Surinam, Paramaribo

**OHSU** Ohio State University, Columbus

**OUMNH** Oxford University Museum of Natural History, Oxford

**RBIN** Institut Royal des Sciences Naturelles, Brussels

**RMCA** Musée Royal de l’Afrique Centrale, Tervuren

**RMNH** National Museum of Natural History NCB Naturalis, Leiden

**QMBA** Queensland Museum, Brisbane

**QSBG** Queen Sirikit Botanical Gardens, Chiang Mai (Thailand)

**SAMA** South Australian Museum, Adelaide

**SAMC** South African Museum, Cape Town

**SEHU** Systematic Entomology Hokkaido University, Sapporo

**SEMC** Snow Entomological Collections, University of Kansas, Lawrence

**SMF** Forschungsinstitut und Naturmuseum Senckenberg, Frankfurt

**SMNS** Staatliches Museum für Naturkunde, Stuttgart

**SNSD** Senckenberg Naturhistorische Sammlungen Dresden, Dresden

**UFPR** Universidade Federal dor Paraná, Curitiba

**UMSP** University of Minnesota, St. Paul

**USNM** United States National Museum, Smithsonian Institution, Washington D.C.

**UTOR** Instituto e Museo di Zoologia di Torino, Turin

**WSU** Washington State University, Pullman

**ZFMK** Zoologisches Forschungsinstitut und Museum Alexander Koenig, Bonn

**ZISP** Russian Academy of Sciences, Zoological Institute, St. Petersburg

**ZMAN** Zoological Museum of Amsterdam - now housed in RMNH, Leiden

**ZMHU** Zoologisches Museum der Humboldt Universität, Berlin

**ZMUC** Zoological Museum University of Copenhagen, Copenhagen

**ZSI** Zoological Survey of India, Calcutta

**ZSM** Zoologische Staatssammlung, Munich

A few private collections have also been studied. These are referred to in text and appendices by giving the initials and full surname of the owner.

If specimens referred to in the species descriptions in Appendix 1 were used for DNA extraction, this is mentioned by citing the voucher codes on the specimen label (e.g. “DNA voucher G. Ståhls Y0909”). These codes are used by the molecular lab of the Finnish Museum of Natural History (MZH), Helsinki.

### Dissection and microscopy

Male genitalia were dissected and macerated in an aqueous 10% KOH solution at ambient temperature for 12–24 hours, rinsed in water and stored in glycerol. Drawings of male genitalia were made with the aid of a drawing tube attached to a Wild M20 compound microscope. Photographs of (parts of) specimens were taken through an Olympus SZX12 motorized stereozoom microscope, using Analysis Extended Focal Imaging Software.

### Morphology

Most of the morphological terminology used in this paper is derived from McAlpine (1981), as specifically applied to Syrphidae by Thompson (1999), who also introduced some new terms. Cheng and Thompson (2008) introduced a few more with special relevance to Microdontinae. For some characters used in the present paper, these works do not provide applicable terms. In these cases terminology is based on Hippa and Ståhls (2005) (e.g. antennal fossa, antetergite) and Speight (1987) (e.g. anterolateral callus of tergite 1, anterior sclerite of sternite 2). For the terminology of the male genitalia McAlpine (1981) was used, supplemented with some more recent considerations as summarized by Sinclair (2000) and adopted by Cumming and Wood (2009). This terminology is worked out for Microdontinae in detail in Reemer and Ståhls (in press).

## Key to genera and species groups

Two keys to genera and generic groups of Microdontinae have been published previously: Hull (1949) and Cheng and Thompson (2008). Characters used in those keys have been considered and some are also used here, but many new characters were necessary to accomodate for new genera and redefined genera. Several taxa are keyed out more than once, either because they are borderline cases or because the key characters are variable between species within these groups. In a few cases the key leads to a species, when this species is an exception among its congeners with regard to the key characters.

A discussion of diagnostic characters of Microdontinae can be found in the Discussion paragraph. A key to distinguish this subfamily from other Syrphidae is also presented there.

**Table:** 

1	Postmetacoxal bridge incomplete (metapleura separated from each other)	97
–	Postmetacoxal bridge complete (metapleura connected posteriad of metacoxa, often only narrowly)	2
2	Vein R4+5 without posterior appendix extending into cell r4+5 (Figs 3, 28, 404)	74
–	Vein R4+5 with posterior appendix extending into cell r4+5 (Figs 14, 17, 206)	3
3	Postpronotum bare	67
–	Postpronotum pilose	4
4	Abdomen constricted	58
–	Abdomen not constricted (oval, parallel-sided or tapering)	5
5	Anepisternum with bare part limited to ventral half of the anepisternum, or entirely pilose	43
–	Anepisternum extensively bare, with bare part reaching dorsad to above half the height of the anepisternum	6
6	Propleuron (proepimeron) bare	13
–	Propleuron (proepimeron) pilose	7
7	Postero-apical corner of wing cell r4+5 more or less rectangular or acute, always with small appendix (e.g. Figs 14, 17, 28, 55)	11
–	Postero-apical corner of wing cell r4+5 widely rounded, sometimes with small appendix (e.g. Figs 177, 206, 210, 289)	8
8	Katepimeron more or less flat (may be a little elevated or with an ill-developed carina, but not convex), sometimes with rows of microtrichia. Abdomen narrow, clearly less than 1.5 times as wide as thorax (Fig. 294)	*Peradon*: *flavofasium-group* (in part)
–	Katepimeron convex, never with microtrichia. Abdomen wide, about 1.5 times as wide as thorax (Figs 176, 197, 200)	9
9	Apical crossvein M1 with outward angle, usually with a small outward appendix, anteriorly recurrent (Fig. 177)	*Microdon (Chymophila)*
–	Apical crossvein M1 without outward angle (Fig. 206, 210)	10
10	Lateral oral margins not or only slightly produced: anterolateral corners not angular (Fig. 202, 207)	*Microdon* s.s.
–	Lateral oral margins strongly produced: anterolateral corners angular (Fig. 229).	*Microdon* s.s.: *virgo*-group
11	Tergites 3 and 4 not fused, able to articulate independently (Fig. 44)	*Ceratophya* (in part)
–	Tergites 3 and 4 fused, not able to articulate independently, although a suture between the tergites is usually visible (look at lateral margins for best judgement)	12
12	Eye bare. Male genitalia: phallus apically furcate (Figs 369, 371, 376)	*Serichlamys*
–	Eye pilose. Male genitalia: phallus unfurcate (Fig. 135)	*Laetodon*
13	Sternites 2 and 3 (often also 1 and 2) separated by unusually wide membraneous part, about as wide as sternite 2 medially or wider (Fig. 393, 394). Antetergite enlarged, longer than tergite 1 medially, almost at level with tergite 1	*Stipomorpha*
–	Sternites 2 and 3 not separated by unusually wide membraneous part. Antetergite small, shorter than or as long as tergite 1 medially, often not at level with tergite 1 but making a smaller angle	14
14	Postero-apical corner of wing cell r4+5 more or less rectangular or acute (usually with small appendix) (Figs 14, 17, 28, 55)	29
–	Postero-apical corner of wing cell r4+5 widely rounded (sometimes with small appendix) (Figs 177, 206, 210, 289)	15
15	Basoflagellomere shorter than scape (Fig. 229)	24
–	Basoflagellomere as long as or longer than scape (Fig. 293)	16
16	Sternite 1 pilose (sometimes only short and sparsely)	21
–	Sternite 1 bare	17
17	Entire body with metallic green to bluish colouration, densely punctate. Mimics of chrysidid wasps (Hymenoptera: Chrysididae) (Figs 63–67)	*Chrysidimyia*
–	At most thorax with faint metallic hues	18
18	Abdomen constricted basally (Fig. 295)	*Peradon*: *trivittatum*-group (in part)
–	Abdomen not constricted	19
19	Male with bifurcate basoflagellomere (Fig. 61). Female unknown, possibly with curved or sickle-shaped basoflagellomere. Australian taxon	*Cervicorniphora*
–	Basoflagellomere unfurcate; oval or parallel-sided. Neotropical taxa	20
20	Tergites without fasciae or vittae of golden or silver pile. Basoflagellomere less than twice as long as scape	*Peradon*: *bidens*-group
–	Tergites usually with fasciae and/or vittae of golden or silver pile. If not, then basoflagellomere more than twice as long as scape	*Peradon*: *flavofascium*-group (in part)
21	Tergite 2 with tubercle halfway on lateral margin (Fig. 421)	*Ubristes*
–	Tergite 2 without tubercle on lateral margin	22
22	Antenna shorter than distance between antennal fossa and anterior oral margin. Basoflagellomere less than twice as long as wide	*Microdon rieki* (Australia)
–	Antenna longer than distance between antennal fossa and anterior oral margin. Basoflagellomere at least four times as long as wide	23
23	Brownish species with long, bee-like pilosity. Scutellum without calcars	*Microdon (Myiacerapis)*
–	Metallic green, sparsely pilose species, reminiscent of chrysidid wasp. Scutellum with calcars	*Microdon* s.s. (in part: *macquartii*)
24	Wings hyaline, at most subtly infuscated	26
–	Wings with black and yellow colour pattern	25
25	Abdomen without conspicuous fasciae of long pile. Scutellum without calcars. < 20 mm	*Microdon* s.l.: *mirabilis*-group
–	Abdomen with conspicuous fasciae of long, white pile; apex long, orange pilose. Scutellum with large calcars. >20 mm. Mimics of *Eulaema* (Hymenoptera: Euglossidae)	*Syrphipogon*
26	Vertex convex and shining	*Pseudomicrodon* (in part: *biluminiferus*)
–	Vertex more or less flat, dull	27
27	Tergites 3 and 4 about equally wide, with lateral margins parallel	*Microdon waterhousei* Ferguson
–	Tergites 3 wider than tergite 4, with lateral margins converging posteriad	28
28	Lateral oral margins strongly produced: anterolateral corners angular (Fig. 229)	*Microdon* s.s.: *virgo*-group (in part)
–	Lateral oral margins not or only slightly produced: anterolateral corners not angular (Figs 202, 207)	*Microdon* s.l.: *erythros*-group
29	Antenna shorter than distance between antennal fossa and anterior oral margin	40
–	Antenna as long as or longer than distance between antennal fossa and anterior oral margin	30
30	Scutellum with apical calcars	34
–	Scutellum without apical calcars, but sometimes sulcate apicomedially or with small patches of microtrichia where calcars could be expected	31
31	Tergites 3 and 4 not fused, able to articulate independently	33
–	Tergites 3 and 4 fused, not able to articulate independently, although a suture between the tergites is usually visible	32
32	Sternite 1 bare	*Menidon falcatus* (in part)
–	Sternite 1 pilose	*Microdon (Dimeraspis) adventitus*
	[The Australian *Archimicrodon browni* (Thompson) keys here too, but condition of pilosity of sternite 1 unknown.]
33	Male basoflagellomere without long pile. Both sexes: hind tibia in lateral view at least 1.5 times as wide as hind metatarsus	*Ceratophya* (in part), South America
–	Male basoflagellomere with long pile. Both sexes: hind tibia in lateral view about as wide as hind metatarsus	*Kryptopyga* (in part), Southeast Asia
34	Occiput dorsally widened (even if only slightly): dorsal eye margin diverging from hind margin of head (Figs 5, 86, 229)	36
–	Occiput evenly narrow over entire length: dorsal eye margin parallel to hind margin of head (Fig. 191).	35
35	Male: first tarsomere of hind leg dorsally without longitudinal groove; strongly swollen: about twice as wide as apex of hind tibia	*Microdon* (s.l.) *tarsalis*
–	Male: first tarsomere of hind leg dorsally with wide longitudinal groove; at most 1.5 times as wide as apex of hind tibia	*Megodon*
36	Scutellar calcars large and blunt (Fig. 183). Male: first tarsomere of hind leg about twice as wide as apex of hind tibia	*Microdon (Dimeraspis) globosus*
–	Scutellar calcars either absent, very small or well-developed and pointed apically. Male: first tarsomere of hind leg at most 1.5 times as wide as apex of hind tibia	37
37	Vertex convex and shining, bare or sparsely pilose only on posterior half (Figs 70, 71)	*Domodon*
–	Vertex not convex and shining, entirely pilose	38
38	Basoflagellomere oval (Figs 365, 367, 370)	*Serichlamys*
–	Basoflagellomere sickle-shaped (Fig. 154)	39
39	Abdomen largely or entirely yellow (Fig. 151)*Menidon falcatus* (in part)
–	Abdomen black	*Archimicrodon* (in part: one undescribed African species)
40	Anepimeron bare on ventral half. Male with eye margins parallel at level of frons, not approaching	*Mermerizon*
–	Anepimeron entirely pilose. Male with eye margins approaching each other at level of frons.	41
41	Scutellum with large, apically rounded and flattened calcars.	*Archimicrodon (Hovamicrodon)*
–	Scutellum without calcars or with calcars pointed apically	42
42	Male genitalia: surstylus in lateral view without long posterior process (Figs 9, 15)	*Archimicrodon* s.s.
–	Male genitalia: surstylus in lateral view with long posterior process (Figs 19, 22–26)	*Archimicrodon* s.l.
43	Basoflagellomere more or less oval or parallel-sided, sometimes with acute apex (Figs 66, 255, 325).	45
–	Basoflagellomere sickle-shaped or flag-shaped (Figs 252, 425)	44
44	Basoflagellomere sickle-shaped: thickened basally, curved dorsad apically. Arista bare. Eye reduced, so gena, vertex and occiput wide (Fig. 252)	*Oligeriops*
–	Basoflagellomere flag-shaped: strongly widened and laterally flattened (Fig. 425). Arista pilose (pile at least half as long as width of arista). Eyes of normal size	Undescribed genus #1, species AUS-01
45	Basoflagellomere shorter than scape	54
–	Basoflagellomere as long as or longer than scape	46
46	Antenna as long as or longer than distance between antennal fossa and anterior oral margin	49
–	Antenna shorter than distance between antennal fossa and anterior oral margin	47
47	Tergite 2 with pair of depressed areas (as in Fig. 287); lateral margins of tergite 2 subcircular, widest point clearly before posterior margin	*Omegasyrphus*
–	Tergite 2 without depressed areas; widest point of tergite 2 at posterior margin.	48
48	Wing with conspicuous black markings in apical half (Fig. 230)	*Microdon pictipennis*
–	Wing without conspicuous black markings, only vaguely infuscated along crossveins	*Microdon nigromarginalis*
49	Tergites 3 and 4 fused, not able to articulate independently, although a suture between the tergites is usually visible (look at lateral margins for best judgement)	51
–	Tergites 3 and 4 not fused, able to articulate independently (Fig. 44)	50
50	Dorsal half of occiput slightly widened: maximum width in lateral view less than 1/4 of eye width. Tergite 4 in lateral view approximately perpendicular to tergite 2 (Fig. 43, 44)	*Ceratophya*
–	Dorsal half of occiput strongly widened: maximum width in lateral view about 1/2 of eye width. Tergite 4 in lateral view not perpendicular to tergite 2	*Microdon shirakii*
51	Metallic green species, mimics of chrysidid wasps (Fig. 63, 64)	*Chrysidimyia*
–	Brownish or partly orange species	52
52	Basoflagellomere more than three times as long as scape; with long pilosity in male (Fig. 325)	*Ptilobactrum*
–	Basoflagellomere less than three times as long as scape; bare in male	53
53	Abdomen narrow: more than 1.5 times as long as wide (Figs 163, 167)	*Metadon*
–	Abdomen wide: less than 1.5 times as long as wide (Fig. 181)	*Microdon (Dimeraspis)*
54	Abdomen about as long as wide	*Microdon (Dimeraspis) abditus*
–	Abdomen clearly longer than wide	55
55	Metallic green or blue flies, mimics of chrysidid wasps (Fig. 63, 64)	*Chrysidimyia*
–	Not metallic green or blue flies	56
56	Tergite 1 long, with hind margin very rounded; length : width ratio 1:2 or longer (Fig. 81, 83)	*Heliodon*
–	Tergite 1 shorter, with hind margin less rounded; length : width ratio 1:2.5 or shorter	57
57	Tergite 2 with pair of depressed areas (Fig. 287). Abdomen more than 2.5 times as long as wide (Fig. 285). Alula bare	*Parocyptamus*
–	Tergite 2 without depressed areas. Abdomen less than 2.5 times as long as wide (Fig. 163, 167). Alula microtrichose along margins	*Metadon*
58	Transverse suture incomplete: not visible medially on mesoscutum	61
–	Transverse suture complete: extending from one notopleuron to the other	59
59	Katepimeron pilose. Male basoflagellomere with long pile (Fig. 47, 54)	*Ceratrichomyia*
–	Katepimeron bare. Male basoflagellomere without long pile	60
60	Frons laterally without concave area; without sharply defined ridge from lunula to eye margin	*Indascia* (in part)
–	Frons laterally with concave area, covered with dense golden pilosity; ventrally this area is delimited by a sharply defined ridge, which runs from the lunula to the eye margin (Figs 410, 413, 414)	*Thompsodon conspicillifrons*
61	Tergites 3 and 4 not fused, able to articulate independently (Fig. 120). Male: sternite 4 not visible in ventral view: completely covered by sternite 3 and lateral margins of tergites (Fig. 123). Male basoflagellomere with long pile (Fig. 122)	*Kryptopyga pendulosa*
–	Tergites 3 and 4 fused, not able to articulate idependently (although a suture between these tergites is usually visible)	62
62	Basoflagellomere longer than scape	64
–	Basoflagellomere shorter than or as long as scape	63
63	Tergite 2 at most as long as anterior width (Fig. 81)	*Heliodon*
–	Tergite 2 more than twice as long as anterior width (Fig. 347)	*Rhopalosyrphus* (s.l.) *oreokawensis*
64	Vertex convex, shining, sparsely pilose to bare (Fig. 310, 315)	*Pseudomicrodon*
–	Vertex more or less flat, dull and entirely pilose (Fig. 339, 344)	65
65	Tergite 2 with anterior margin about as wide as posterior margin (Fig. 295)	*Peradon: trivittatum*-group
–	Tergite 2 with anterior margin at least 1.5 times as wide as posterior margin (Fig. 332, 337, 342)	66
66	Katepimeron pilose (sometimes only along anterior margin)	*Rhopalosyrphus* s.s.
–	Katepimeron bare	*Rhopalosyrphus* s.l.
67	Abdomen oval or elongate, not constricted in dorsal view (Fig. 35, 269, 293, 294)	69
–	Abdomen constricted in dorsal view (i.e. with narrowest point (in dorsal view) at tergite 2 and widest point at tergite 3 or 4) (Fig. 271, 272, 276)	68
68	Postero-apical corner of wing cell r4+5 widely rounded. Segment 2 longer than thorax (Fig. 59)	*Ceriomicrodon petiolatus*
–	Postero-apical corner of wing cell r4+5 more or less rectangular or acute, with small appendix (Fig. 271). Segment 2 usually shorter than or as long as thorax (Figs 269, 284) (except in one undescribed African taxon)	*Paramixogaster* (in part)
69	Basoflagellomere about six times as long as scape (Fig. 36)	*Bardistopus*
–	Basoflagellomere at most four times as long as scape	70
70	Abdomen about as long as wide, with tergite 2 about as long as tergites 3 and 4 together (Fig. 397, 398)	*Sulcodon*
–	Abdomen at least 1.5 times as long as wide, with tergite 2 less than half as long as tergites 3 and 4 together	71
71	Face medially with vitta of transversely wrinkled texture (Fig. 291)	*Peradon*: *flavofascium-group* (in part)
–	Face medially smooth	72
72	Basoflagellomere longer than scape	*Paramixogaster* (in part: *Paramixogaster acantholepidis*, *Paramixogaster crematogastri*)
–	Basoflagellomere shorter than scape	73
73	Tergite 2 twice as wide as long or wider; entirely black	*Metadon* (in part: *Metadon bifasciatus*)
–	Tergite 2 about 1.5 times as wide as long; with large yellow marking in shape of an inverted “V”	*Microdon trigonospilus* Bezzi
74	Vein M anteriorly without small stump extending into cell r4+5 (Fig. 3, 389, 404)	76
–	Vein M anteriorly with small stump extending into cell r4+5 (Fig. 28, 242, 244)	75
75	Crossvein r-m located between basal 1/4 and 1/3 of cell dm (Fig. 242, 244)	*Mixogaster*
–	Crossvein r-m located within basal 1/7 of cell dm (Fig. 28)	*Aristosyrphus* (in part: some specimens of *Aristosyrphus primus*)
76	Face with median tubercle on dorsal half (Fig. 31)	*Aristosyrphus* (*Eurypterosyrphus*)
–	Face without median tubercle (Fig. 5)	77
77	Vein M1 more or less straight, not parallel to wing margin, making straight angle with vein R4+5 (Fig. 14, 166, 219)	79
–	Vein M1 at least in anterior half (sometimes also in posterior half) oblique, more or less parallel to wing margin, making acute angle with vein R4+5 (Fig. 3, 28)	78
78	Abdomen constricted or parallel-sided, not or only slightly wider than thorax (Fig. 27)	*Aristosyrphus* s.s.
–	Abdomen oval, clearly wider than thorax (cf. Fig. 7, 20)	*Afromicrodon*
79	Abdomen constricted (i.e. with narrowest point at tergite 2 and widest point at tergite 3 or 4) or elongate and parallel-sided (Figs 103, 262, 284)	89
–	Abdomen oval (Figs 7, 10, 20, 401) or tapering / triangular (Figs 388, 392) (tergite 2 may be quite narrow anteriorly, but then the abdomen does not get wider beyond posterior margin of tergite 2)	80
80	Sternites 2 and 3 (often also 1 and 2) separated by unusually wide membraneous part, about as wide as sternite 2 medially or wider (Figs 393, 394). Antetergite of tergite 1 enlarged, longer than tergite 1 medially, almost at level with tergite 1	*Stipomorpha*
–	Sternites 2 and 3 not separated by unusually wide membraneous part. Antetergite small, shorter than or as long as tergite 1 medially, often not at level with tergite 1 but making a smaller angle	81
81	Basoflagellomere shorter than or as long as scape (basoflagellomere never furcate)	93
–	Basoflagellomere longer than scape (basoflagellomere sometimes furcate in male)	82
82	Antenna at least as long as distance between antennal fossa and anterior oral margin, furcate in male (Figs 39, 77, 138, 144, 356–361)	86
–	Antenna shorter than distance between antennal fossa and anterior oral margin, never furcate	83
83	Thorax and abdomen black	*Archimicrodon* s.l. (undescribed taxa from Papua New Guinea)
–	Thorax and abdomen yellow and black	84
84	Postpronotum bare	*Surimyia*
–	Postpronotum pilose	85
85	Position of crossvein r-m at same level as bm-cu (Fig. 258)	*Paragodon*
–	Position of crossvein r-m more apical: approximately at basal 1/8 of cell dm	*Hypselosyrphus* (in part)
86	Scutellum sulcate apicomedially (cf. Fig. 183)	88
–	Scutellum not sulcate apicomedially, more or less semicircular	87
87	Antenna inserted dorsally on head: at or above dorsal eye margin. Male basoflagellomere multifurcate (Figs 138, 142–144, 149)	*Masarygus*
–	Antenna inserted below dorsal eye margin. Male basoflagellomere bifurcate (Figs 356–360)	*Schizoceratomyia*
88	Katepisternum pilose. Metasternum developed and pilose	*Furcantenna*
–	Katepisternum bare. Metasternum underdeveloped and bare	*Carreramyia*
89	Postpronotum pilose	91
–	Postpronotum bare	90
90	Antenna longer than distance between antennal fossa and anterior oral margin. Basoflagellomere more than 3 times as long as wide (Figs 269–277)	*Paramixogaster* (in part: *Paramixogaster decipiens* (de Meijere) and undescribed Australian sp.)
–	Antenna shorter than distance between antennal fossa and anterior oral margin. Basoflagellomere less than 2 times as long as wide (Figs 301, 302, 306)	*Piruwa*
91	Mesoscutum with transverse suture complete (reaching from one notopleuron to the other)	*Indascia*
–	Mesoscutum with transverse suture not complete (not visible medially)	92
92	Antenna longer than distance between antennal fossa and anterior oral margin. Male basoflagellomere bifurcate (Figs 430, 432)	Undescribed genus #2, species MCR-02
–	Antenna shorter than distance between antennal fossa and anterior oral margin. Male basoflagellomere not furcate (Figs 262–266)	*Paramicrodon*
93	Katepimeron pilose	*Hypselosyrphus ulopodus*
–	Katepimeron bare	94
94	Occiput wide, both dorsally and ventrally (Fig. 328)	*Rhoga*
–	Occiput narrow, at least on ventral half (Figs 5, 229, 403)	95
95	Postpronotum bare	*Surimyia*
–	Postpronotum pilose	96
96	Vertex not produced, more or less flat (Figs 3–5)	*Afromicrodon*
–	Vertex produced, more or less convex (Figs 99, 100)	*Hypselosyrphus*
97	Abdomen oval or more or less parallel-sided, not constricted (Fig. 330). Occiput without creases (Fig. 328)	*Rhoga* (in part: *maculata, mellea, sepulchrasilva*)
–	Abdomen elongate and constricted (narrowest point at transition between tergites 2 and 3) (Figs 377, 382). Occiput with distinct creases (Figs 379, 384)	98
98	Proanepisternum without row of long stiff pile. Eye bare	*Spheginobaccha* (*perialla*-group)
–	Proanepisternum with row of long stiff pile. Eye bare or pilose	99
99	Eye pilose. Alula microtrichose	*Spheginobaccha* (*macropoda*-group)
–	Eye bare. Alula partially bare	*Spheginobaccha* (*rotundiceps*-group)

## Genera accounts

### Order and format

The genera accounts are presented in alphabetic order. Accounts are only given for taxa considered as valid genera or subgenera. Synonyms and misspelled names can be found under the valid genera to which they belong. Each group account starts with information on the original description and the type species. This is followed by the following components.

**Description.** Body length (intended only as an approximation, as not all specimens have been measured). A short characterization of the habitus is given, followed by a general description, which is intended to give characters considered (potentially) useful for identification, and to indicate the variability of characters. Unless stated otherwise, all listed characters apply to both sexes. Illustrations are given to illustrate habitus, important external characters and male genitalia. Additional morphological characters can be found in the character matrix of Reemer and Ståhls (in press).

**Diagnosis.** The shortest possible enumeration of external characters considered sufficient to distinguish the genus from all other Microdontinae. Characters of the male genitalia are only given in a few cases. The combination of the given characters is necessary for the diagnosis; all characters not given are considered unnecessary for this purpose. In some cases this diagnosis will not add much to the characters given in the key, but in other cases it will provide a ‘short-cut’ to the recognition of the genus.

**Discussion.** Arguments are given for the proposed classification. Other comments are given when necessary, e.g. on type specimens, history of classification, and morphological characters.

**Diversity and distribution.** The number of described species is given, sometimes with a speculation on the possible number of undescribed species. When available, a reference to species keys is given. The known geographic range is indicated.

**Etymology.** Only given for newly described genera.

### Afromicrodon

Thompson

http://species-id.net/wiki/Afromicrodon

Figs 3–6

Afromicrodon Thompson, 2008: 26 (in Cheng and Thompson 2008). Type species: *Microdon johannae* van Doesburg, 1957: 109, by original designation.

#### Description.

Body length: 6–9 mm. Relatively small flies with short antennae and oval abdomen. Head slightly wider than thorax. Face evenly convex; narrower than an eye. Lateral oral margins not produced. Vertex flat. Occiput narrow over entire length. Eye bare. Eyes in male strongly approaching each other at level of frons; with mutual distance about equal to width of antennal fossa. Antennal fossa about as high as wide. Antenna shorter than distance between antennal fossa and anterior oral margin. Basoflagellomere approximately as long as scape; oval, short; bare. Postpronotum pilose. Anepisternum without sulcus; pilose, except bare on ventral 1/4. Anepimeron pilose on dorsal half, bare on ventral half. Katepimeron convex; bare. Scutellum semicircular; without calcars. Wing: vein R4+5 without appendix; vein M1 anterior half directed somewhat outward, making acute angle with R4+5, posterior half perpendicular to vein M; crossvein r-m located around basal 1/4 of cell dm. Abdomen oval. Male genitalia: phallus straight, not furcate; hypandrium with bulb-like base and basolateral bulges; epandrium without ventrolateral ridge; surstylus large: about as long as hypandrium, somewhat sickle-shaped.

#### Diagnosis.

Vertex flat. Occiput narrow. Antenna shorter than distance between antennal fossa and anterior oral margin. Postpronotum pilose. Katepimeron bare. Vein R4+5 without posterior appendix. Abdomen oval.

#### Diversity and distribution.

Described species: 5. Restricted to Madagascar and the Comorean islands.

### Archimicrodon

Hull

http://species-id.net/wiki/Archimicrodon

Figs 7 [Fig F4] –26

Archimicrodon Hull, 1945: 75. Type species: *Microdon digitator* Hull, 1937: 19, by original designation.
Hovamicrodon (Subgenus) Keiser, 1971: 248. Type species: *Hovamicrodon silvester* Keiser, 1971: 251, by original designation. **stat. n.**

#### Description.

Body length: 4–11 mm. Small to moderately sized flies with short antennae and oval abdomen. Head about as wide as thorax or slightly wider. Face convex; narrower than an eye. Lateral oral margins not produced. Vertex flat. Occiput ventrally narrow, dorsally widened. Eye bare. Eye margins in male strongly converging at level of frons, with mutual distance about as large as width of antennal fossa. Antennal fossa about as wide as high. Antenna shorter than distance between antennal fossa and anterior oral margin; basoflagellomere as long as or longer than scape, oval, sometimes with acute apex and concave dorsal margin; bare. Postpronotum pilose. Scutellum semicircular; with or without calcars, sometimes apicomedially sulcate; in subgenus *Hovamicrodon* calcars are spatulate (spoon-shaped). Anepisternum weakly sulcate; pilose anteriorly and posteriorly, widely bare in between. Anepimeron entirely pilose. Katepimeron convex; bare. Wing: vein R4+5 with or without posterior appendix (this appendix only lacks in certain undescribed species from New Guinea); vein M1 perpendicular to vein R4+5; postero-apical corner of cell r4+5 rectangular, with small appendix; crossvein r-m located around basal 1/5 to 1/4 of cell dm. Abdomen oval, about 1.5 to 2 times as long as wide. Tergites 3 and 4 fused. Sternite 1 pilose or bare. Male genitalia: phallus furcate, with furcation point near apex; hypandrium with basal part bulb-like; epandrium without ventrolateral ridge; surstylus unfurcate.

#### Diagnosis.

Abdomen oval. Antenna shorter than distance between antennal fossa and anterior oral margin. Postpronotum pilose. Postero-apical corner of cell r4+5 rectangular. Proepimeron bare. Anepisternum widely bare medially, also on dorsal half. Anepimeron entirely pilose. Vein R4+5 usually with posterior appendix; if not: thorax and abdomen entirely black.

#### Discussion.

*Archimicrodon* was described as a subgenus of *Microdon* by Hull (1945), and considered as such by subsequent authors, including Cheng and Thompson (2005), who stated that three species are included. Most of the species assigned to *Archimicrodon* in the present paper were previously placed in *Microdon*. However, they do not agree with the more strict definition of *Microdon* used here. Its independent position from *Microdon* and its monophyly are supported by the phylogenetic results of Reemer and Ståhls (in press). These are reasons to raise *Archimicrodon* to generic status.

Three groups are recognized within this genus: *Archimicrodon* s.s., the subgenus *Hovamicrodon*, and a ‘leftover’ group, here called *Archmicrodon* s.l. *Archmicrodon* s.s. is based on *Archmicrodon simplicicornis* (de Meijere, 1908), a subjective senior synonym of the type species of the genus, *Microdon digitator* Hull, 1937 syn. n. *Archimicrodon* s.s. is here defined by the shape of the surstylus: more or less oval, without a long posterior process (Fig 9, 15); scutellar calcars are either present or absent, but never spatulate. The subgenus *Hovamicrodon* is defined (following Keiser 1971) by the spatulate shape of the scutellar calcars (Fig. 18); the surstylus has a long posterior process (Fig. 19). *Archimicrodon* s.l. is here defined as containing all other species, in which the scutellar calcars are absent or - if present - not spatulate, and in which the surstylus has a long posterior process (Figs 22–26). As far as the African species are concerned, this group corresponds with the *brevicornis*-group of Bezzi (1915).

The three groups are very similar in their morphology, except for the small differences as noted above. It seems likely that the groups are closely related. The subgenus *Hovamicrodon* is probably monophyletic, considering the spatulate scutellar calcars in combination with its restricted distribution (Madagascar). However, as the phylogenetic analyses by Reemer and Ståhls (in press) indicate, it is so closely related to *Archimicrodon* s.l. (which is recovered as paraphyletic with respect to *Hovamicrodon*) that a separate generic status seems not warranted. Besides, a spatulate shape of the scutellar calcars can also be found in certain species of the New World genera *Laetodon* gen. n. and *Serichlamys* Curran, 1925. The latter genus is recovered as sister to *Archimicrodon* by Reemer and Ståhls (in press). As this character is not unique, it does not provide sufficient basis to base a genus on.

Sexual dimorphism can be pronounced, especially in the African species of this group (including *Hovamicrodon*). Females tend to be much larger than males, and are different in colouration (usually darker). As several species were described from one sex only (such as certain Madagascar species described by Keiser 1971), it is possible that some of these species are actually synonyms. However, as many taxa are represented by only one specimen, these matters cannot yet be resolved.

*Hova* is the name of one of the social castes of the Merina, an ethnic group indigenous to Madagascar. Keiser (1971) used this name for his genus *Hovamicrodon*. Surprisingly, he did not include the Madagascar species *Microdon hova* Hervé-Bazin, 1913 in this genus, although this species clearly belongs to this group (spatulate scutellar calcars). Keiser (1971) does mention a specimen which he believes to be *Microdon hova*, based on the description, but for some reason this species is not listed under *Hovamicrodon*. However, when Keiser died in 1969, his paper was not finished yet. It was published posthumously, after the manuscript was finished and submitted by E. Lindner. Therefore, it is seems possible that Keiser intended to include *Microdon hova* in *Hovamicrodon*.

#### Notes on species.

In genitalia, *Microdon browni* Thompson, 1968 is similar to *Archimicrodon* s.l.: phallus short, apically furcate, with dorsobasal projection; hypandrium with bulb-like base; surstylus with two elongate lobes; epandrium without ventrolateral ridge. In external morphology, the only difference with *Archimicrodon* seems to be that the antennae are longer than the distance between the antennal fossa and the anterior oral margin. This character is considered not important enough for group definition, as antennal length is quite variable within many genera of Microdontinae. For these reasons, *Microdon browni* is here considered as a species of *Archimicrodon* s.l. The phylogenetic analysis of morphological characters by Reemer and Ståhls (in press) provides no further clue to the taxonomic affinities of this taxon.

#### Diversity and distribution.

Described species: 45. Widely distributed in the Afrotropical, Oriental and Australasian regions, with one species known from the Eastern Palaearctic (*Archimicrodon simplex* (Shiraki, 1930)). *Archimicrodon* s.s. is only known from the Oriental region. The number of species of *Archimicrodon* s.s. is not known, as the male genitalia of several species were not studied. The subgenus *Hovamicrodon* (six species) is restricted to Madagascar.

### Aristosyrphus

Curran

http://species-id.net/wiki/Aristosyrphus

Figs 27–34

Aristosyrphus Curran, 1941: 247. Type species: *Aristosyrphus primus* Curran, 1941: 252, by original designation.
Protoceratophya Hull, 1949: 314. Type species: *Ceratophya carpenteri* Hull, 1945: 76, by original designation. For synonymy see Cheng and Thompson (2008).
Paraceratophya Fluke, 1957: 38. Misspelling of *Protoceratophya* Hull.
Eurypterosyrphus (Subgenus) Barretto & Lane, 1947: 141. Type species: *Eurypterosyrphus melanopterus* Barretto & Lane, 142, by original designation. Status as subgenus: Cheng and Thompson (2008).

#### Description.

*Aristosyrphus (Aristosyrphus)*. Body length: 6–18 mm. Slender flies, often with constricted abdomen. Head wider than thorax. Face convex or almost straight in profile; about as wide as an eye or narrower. Lateral oral margins not produced. Vertex flat. Occiput narrow over entire length. Eye bare. Eyes in male weakly converging at level of frons, with mutual distance 2 to 3 times the width of antennal fossa. Antennal fossa about as wide as high. Antenna longer or shorter than distance between antennal fossa and anterior oral margin; basoflagellomere longer than scape, oval; bare. Postpronotum pilose. Scutellum semicircular; without calcars. Anepisternum without or with weak sulcus; anteriorly pilose or bare, posteriorly pilose, with pile limited to dorsal half. Anepimeron entirely pilose. Katepimeron convex; bare. Wing: vein R4+5 without posterior appendix; vein M1 making acute angle with vein R4+5, anterior part or entire vein M1 parallel to wing margin; postero-apical corner of cell r4+5 angular, with small appendix; crossvein r-m located within basal 1/7 of cell dm, often very close to base. Abdomen elongate: slightly oval, parallel-sided or constricted at segment 2; more than twice as long as wide. Tergites 3 and 4 fused. Male genitalia: phallus unfurcate, straight or bent dorsad; ejaculatory hood apicodorsally separately developed from actual phallus into prong-like structure, which may be mistaken for dorsal aedeagal process, but does not contain a sperm-duct; apical part of hypandrium consists of two separate lobes (separated ventromedially); epandrium without ventrolateral ridge; surstylus furcate or unfurcate.

#### Description.

*Aristosyrphus (Eurypterosyrphus)* Body length: 8-14 mm. Slender flies with parallel-sided, constricted or kite-shaped abdomen. Head wider than thorax. Face more or less straight, with median tubercle on dorsal half; about as wide as an eye or narrower. Vertex flat. Occiput narrow over entire length. Eye bare. Eyes in male not or only slightly converging at level of frons, with mutual distance 4 to 5 times the width of antennal fossa. Antennal fossa about as wide as high. Antenna longer than distance between antennal fossa and anterior oral margin; basoflagellomere shorter or longer than scape, oval, sometimes appearing swollen: more than twice as wide as scape; bare. Postpronotum pilose. Scutellum semicircular; without calcars. Anepisternum without or with weak sulcus; pilose on dorsal half, bare ventrally. Anepimeron pilose on dorsal half, bare ventrally. Katepimeron convex; bare. Wing: vein R4+5 without posterior appendix; vein M1 making straight or acute angle with vein R4+5; postero-apical corner of cell r4+5 angular, with small appendix; crossvein r-m located around basal 1/3 of cell dm. Abdomen parallel-sided, constricted or kite-shaped; more than twice as long as wide. Tergites 3 and 4 fused. Male genitalia: phallus unfurcate, straight or bent dorsad; ejaculatory hood apicodorsally enveloping phallus; apical part of hypandrium consists of two separate lobes (separated ventromedially); hypandrium in some species with elongate ventromedian structure parallel to phallus Figs 32, 34), resembling the lingula of certain taxa of the subfamily Syrphinae; epandrium without ventrolateral ridge; surstylus furcate or unfurcate.

#### Diagnosis.

Vein R4+5 without posterior appendix. Abdomen elongate and parallel-sided or constricted. Postpronotum pilose. Mesoscutum with transverse suture incomplete. Antenna longer than distance between antennal fossa and anterior oral margin.

*Aristosyrphus* s.s. Vein M1 oblique, at least anterior half parallel to wing margin. Face evenly convex. Anepimeron entirely pilose. Crossvein r-m located around basal 1/3 of cell dm. Ejaculatory hood apicodorsally developed into prong-like structure, separate from actual phallus (phallus may seem furcate under casual observation, but ejaculatory hood does not contain sperm duct).

*Eurypterosyrphus*. Vein M1 oblique or straight. Face with median tubercle. Anepimeron bare on ventral half. Crossvein r-m located within basal 1/7 of cell dm. Ejaculatory hood apicodorsally enveloping phallus, not developed into separate, prong-like structure.

#### Discussion.

Morphological variation within this group is large, especially in the male genitalia (Figs 29, 32–34). In some specimens of *Aristosyrphus primus* Curran, 1941 an anterior stump is present at vein M (Fig. 28). This character has always been used as diagnostic for *Mixogaster* (Hull 1954, Cheng and Thompson 2008).

#### Diversity and distribution.

Described species: 7 (*Aristosyrphus* s.s.: 4; *Eurypterosyrphus*: 3). Several undescribed species are known to the first author. Central and South America.

### Bardistopus

Mann

http://species-id.net/wiki/Bardistopus

Figs 35–37

Bardistopus Mann, 1920: 61. Type species: *Bardistopus papuanum* Mann, 1920: 61, by original designation.

#### Description.

Body length: 6–7 mm. Small, dark flies with very long antennae and oval abdomen, which in lateral view appears constricted. Head slightly wider than thorax. Face evenly convex. Lateral oral margins not produced. Vertex flat. Occiput ventrally narrow; dorsally slightly widened. Eye bare. Eyes in male not converging at level of frons; mutual distance much larger than width of antennal fossa. Antennal fossa about as high as wide. Antenna longer than distance between antennal fossa and anterior oral margin. Basoflagellomere about six times as long as scape. Postpronotum bare. Scutellum semicircular; without calcars. Anepisternum without sulcus; pilose anteriorly and posteriorly, widely bare in between. Anepimeron pilose on dorsal half, bare on ventral half. Katepimeron flat; pilose. Wing: vein R4+5 with posterior appendix; vein M1 perpendicular to vein R4+5 and vein M; crossvein r-m located around basal 1/7 of cell dm. Abdomen oval in dorsal view, but in lateral view appearing constricted due to flattened segment 2. Tergites 3 and 4 fused. Male genitalia: phallus furcate, with furcation point in apical half, strongly bent dorsad; epandrium without ventrolateral ridge; surstylus elongate, bent dorsad.

#### Diagnosis.

Vein R4+5 with posterior appendix. Postpronotum bare. Abdomen in dorsal view oval; in lateral view constricted at segment 2. Basoflagellomere about six times as long as scape.

#### Discussion.

No statements about taxonomic affinities of *Bardistopus* have been made previously, except Cheng and Thompson (2008) who wrote that the name was ‘established for a *Microdon* species, which has a greatly elongate basoflagellomere’. Clearly the taxon does not belong to *Microdon*, because of the long basoflagellomere and the structure of the male genitalia (apically furcate phallus). These characters, combined with the bare postpronotum, suggest it might be related to *Paramixogaster*. However, the phylogenetic analysis of morphological characters by Reemer and Ståhls (in press) place it as a sister group of a clade containing taxa of which the male has a furcate basoflagellomere: *Schizoceratomyia*, *Furcantenna* and *Carreramyia*. Future studies employing molecular data could help elucidate the phylogenetic affinities of *Bardistopus*.

According to Mann (1920) the type specimens of the type species are females, but actually both are males (coll. USNM).

#### Diversity and distribution.

Described species: 1. Solomon Islands: Ugi.

### Carreramyia

Doesburg stat. n.

http://species-id.net/wiki/Carreramyia

Figs 38–41

Carreramyia van Doesburg, 1966: 93. Type species: *Microdon megacephalus* Shannon, 1925: 213, by original designation.

#### Description.

Body length: 5–8 mm. Yellowish brown or black flies, tergites sometimes yellow with dark vittae. Mimics of stingless, *Trigona*-like bees (Apidae: Meliponini), due to the brush-like pilosity of the hind tibiae and the more or less triangular abdomen. Head wider than thorax. Face more or less straight in profile; wider than eye. Lateral oral margins not produced. Vertex strongly produced. Occiput ventrally narrow, dorsally widened. Eye bare. Eyes in male not approaching each other; separated over distance much wider than antennal fossa. Antennal fossa about as high as wide. Antenna longer than distance between antennal fossa and anterior oral margin. Antenna inserted below dorsal eye margin; basoflagellomere at least four times as long as scape, bifurcate in male, unfurcate in female; bare. Postpronotum pilose. Anepisternum without sulcus; continually pilose on dorsal half, bare on ventral half. Anepimeron pilose on dorsal half, bare on ventral half. Katepimeron convex; bare. Wing: vein R4+5 without posterior appendix; vein M1 perpendicular to R4+5 and M; crossvein r-m located close to bm-cu. Abdomen more or less triangular, with tergites 3 and 4 narrower than tergite 2. Tergites 3 and 4 fused. Sternite 1 bare or pilose. Male genitalia: phallus straight, furcate near apex; hypandrium with bulb-like base and basolateral bulges; epandrium without ventrolateral ridge.

#### Diagnosis.

Hind tibia widened and with long, brush-like pilosity. Vein R4+5 without posterior appendix. Vertex strongly produced but not shining and convex. Basoflagellomere at least four times as long as scape, bifurcate in male.

#### Diversity and distribution.

Described species: 2. Only the type species, *Carreramyia megacephalus* (Shannon, 1925), is known from more than one specimen (Panama and Costa Rica). The other species was found in Peru. Descriptions of two additional species from Peru and Surinam are in preparation by the first author. Apparently the genus is widespread in the Neotropical region.

### Ceratophya

Wiedemann

http://species-id.net/wiki/Ceratophya

Figs 42–45

Ceratophya Wiedemann, 1824: 14. Type species *Ceratophya notata* Wiedemann, 1824: 14, by subsequent designation of Blanchard (1846: 145).
Ceratophyia Osten Sacken, 1858: 46. Misspelling.

#### Description.

Body length: 7–9 mm. Relatively small, black and yellow flies with long antennae and oval abdomen. Face in profile straight, with anterior oral margin somewhat produced ventrad; laterally depressed, therefore slightly carinate medially; somewhat wider than an eye. Lateral oral margins not produced. Vertex flat. Occiput narrow ventrally, slightly widened dorsally. Eye bare. Eyes in male not approaching each other, eye margins parallel; mutual distance much larger than width of antennal fossa. Antennal fossa about as high as wide. Antenna longer than distance between antennal fossa and anterior oral margin; basoflagellomere longer than scape; elongate, oval. Postpronotum pilose. Anepisternum with shallow sulcus; entirely short pilose, except bare on ventral 1/4. Anepimeron entirely pilose. Katepimeron weakly convex; bare. Scutellum semicircular or apicomedially sulcate; without calcars. Wing: vein R4+5 with posterior appendix; vein M1 perpendicular to vein R4+5 and vein M. Legs: hind tibia somewhat swollen; hind metatarsus enlarged, quadrate, sometimes with strong basoventral tooth. Abdomen with tergite 4 in lateral view more or less perpendicular to tergite 2. Tergites 3 and 4 not fused, able to articulate independently; in female with posterior margin of tergite 3 strongly overlapping tergite 4. Male genitalia: phallus strongly bent dorsally, furcate basally, with ejaculatory hood dorsally strongly elongate and thus forming a third process about equally long as two aedeagal processes; epandrium with ventrolateral ridges.

#### Diagnosis.

Tergites 3 and 4 not fused, strongly overlapping. Tergite 4 in lateral view more or less perpendicular to tergite 2. Basoflagellomere bare; longer than scape.

#### Discussion.

Cheng and Thompson (2008) point out the confused taxonomic history of *Ceratophya*. Unlike these authors, who consider the group as a subgroup of *Microdon*, it here treated as a separate genus. This is done because of the phylogenetic results of Reemer and Ståhls (in press) and because it does not agree with the diagnosis of *Microdon* as defined in the present paper.

#### Diversity and distribution.

Described species: 4. Description of one additional species from Argentina is in preparation by the first author. Known from Central and South America (Panama to northern Argentina).

### Ceratrichomyia

Séguy stat. n.

http://species-id.net/wiki/Ceratrichomyia

Figs 46 –58

Ceratrichomyia Séguy, 1951: 14. Type species: *Ceratrichomyia behara* Séguy, 1951: 14, by original designation.

#### Description.

Body length: 7–10 mm. Slender, black flies with yellow markings and a constricted abdomen. Head wider than thorax, face and vertex wider than an eye. Face ventrally produced in profile; wider than an eye. Lateral oral margins not produced. Vertex swollen. Occiput narrow ventrally, strongly widened dorsally. Eye bare. Eyes in male not approaching each other; smallest mutual distance much larger than width of antennal fossa. Antennal fossa about as high as wide. Antenna longer than height of head. Basoflagellomere at least three times as long as scape; with long pilosity. Postpronotum pilose or bare. Mesoscutum with transverse suture complete. Scutellum without calcars. Anepisternum with deep sulcus; entirely pilose. Anepimeron entirely pilose. Katepimeron convex; pilose or bare. Wing: vein R4+5 with posterior appendix; vein M1 straight, perpendicular to R4+5 and M; postero-apical corner of cell r4+5 rectangular, with small appendix; crossvein r-m located around basal 1/4 of cell dm. Abdomen constricted at segment 2. Tergites 3 and 4 not fused, able to articulate independently. Sternite 1 bare. Sternite 4 in male covered by genital capsule, therefore not visible without removing genitalia. Male genitalia: phallus straight or slightly bent dorsad, with spherical base very large, at least as long as remaining part of phallus; phallus furcate near apex; epandrium with or without ventrolateral ridge; surstylus deeply furcate.

**Diagnosis.** The combination of a complete transverse suture on the mesoscutum and a constricted abdomen is only found in *Ceratrichomyia*,*Indascia* Keiser, Indascia, 1958, *Thompsodon* gen. n. and certain species of *Paramixogaster* Brunetti, 1923. Males are easily distinguished from all these taxa by the long pilosity of the basoflagellomere, and also by sternite 4, which is covered by the genital capsule. From *Paramixogaster* this genus also differs by the unfused tergites 3 and 4. Females are unknown.

**Discussion.** Séguy (1951) attributed one species to this genus. He designated a male and a female as ‘types’, and another male as ‘cotype’. These are here all considered as syntypes. Examination of these three specimens made clear that they belong to three different species, which makes it necessary to designate a lectotype. The male with the following label data is here designated as lectotype. Label 1: “Madagascar, Behara”; label 2 (blue): “Museum Paris, III-38, A. Seyrig”; label 3 (red): “Type”; label 4: “*Ceratrichomyia behara* type du genre [male symbol] Séguy 50”; coll. MNHN. A redescription of the lecotype is given in the next section of the present paper. By this lectotype designation, the other two syntypes become paralectotypes. The male collected in Bekily (Madagascar) belongs to a new species of *Ceratrichomyia*, which is described in the present paper as *Ceratrichomyia bullabucca* spec. n. The female paralectotype, collected in Bekily, is here considered to belong to a previously undescribed species of *Paramixogaster*, because it possesses all characters described as diagnostic for that genus (see genus account). A description of that species is given under the name *Paramixogaster piptotus* sp. n. A third species attributed to this genus, *Ceratrichomyia angolensis* sp. n., is described from Angola.

The long pilosity of the male basoflagellomere was used by Séguy (1951) as a character to set his African genus *Ceratrichomyia* apart from other Microdontinae. This character is also present in *Ptilobactrum* Bezzi, 1915, another African taxon. Apparently Séguy was not aware of this, as he did not refer to *Ptilobactrum*. Cheng and Thompson (2008) did notice the similarity in antennal structure in both taxa and, based on the descriptions, proposed to regard *Ceratrichomyia* as a subjective junior synonym of *Ptilobactrum*.

Study of the type specimens of *Ceratricomyia* and *Ptilobactrum* revealed that these taxa are in fact very different. While *Ceratrichomyia* has, for instance, a constricted abdomen with unfused tergites 3 and 4, *Ptilobactrum* has a conical abdomen with fused tergites 3 and 4. The structures of the male genitalia are also very different (compare Figs 56–58 with 326), e.g. with a deeply furcate surstylus in *Ceratrichomyia* and an unfurcate one in *Ptilobactrum*. Considering these morphological differences, and supported by the phylogenetic results of Reemer and Ståhls (in press), *Ceratrichomyia* is here re-instated as a valid genus.

**Diversity and distribution.** Described species: 3. Two species are known from Madagascar, one from the African mainland (Angola).

### Ceriomicrodon

Hull

http://species-id.net/wiki/Ceriomicrodon

Figs 59–60

Ceriomicrodon Hull, 1937a: 25. Type species: *Ceriomicrodon petiolatus* Hull, 1937: 25, by original designation.

#### Description.

Body length: 11 mm. Very slender, wasp-like flies with long antennae and constricted abdomen. Face convex, somewhat produced on ventral half; narrower than an eye. Lateral oral margins clearly produced. Vertex flat. Occiput ventrally narrow, dorsally somewhat widened. Eye bare; frontally with narrow, horizontal area of enlarged ommatidia at level of antenna. Eyes in male strongly convergent at level of frons. Antennal fossa about 1.5 times as wide as high. Antenna longer than height of head; basoflagellomere more than twice as long as scape; bare. Postpronotum bare. Anepisternum with shallow sulcus; pilose along posterior margin and sparsely anterodorsally, widely bare in between. Anepimeron entirely pilose. Katepimeron flat; bare. Scutellum semicircular; without calcars. Wing: vein R4+5 with posterior appendix; vein M1 perpendicular to vein R4+5; postero-apical corner of cell r4+5 widely rounded; crossvein r-m located around basal 1/3 of cell dm. Abdomen very slender, constricted at tergite 2. Tergite 2 longer than thorax, about as long as tergites 3-5 together. Tergites 3 and 4 fused. Male genitalia: phallus furcate near apex, with dorsal process long and whip-like, ventral process very short; epandrium with ventrolateral ridge.

#### Diagnosis.

Postpronotum bare. Vein R4+5 with posterior appendix. Postero-apical corner of cell r4+5 widely rounded. Abdomen constricted. Tergite 2 longer than thorax.

#### Discussion.

*Ceriomicrodon* is treated as a subgenus of *Microdon* by Thompson et al. (1976) and Cheng and Thompson (2008). However, it does not agree with the diagnosis of *Microdon* as used in the present paper, because of several characters (e.g. postpronotum bare, abdomen petiolate, phallus with dorsal process long and whip-like). In addition, the phylogenetic results of Reemer and Ståhls (in press) indicate a relationship with e.g. *Pseudomicrodon* and *Rhopalosyrphus*.

#### Diversity and distribution.

Described species: 1. Known from Central (Mato Grosso) and Northern Brazil (Roraima).

### Cervicorniphora

Hull stat. n.

http://species-id.net/wiki/Cervicorniphora

Figs 61–62

Cervicorniphora Hull, 1945: 75. Type species: *Microdon alcicornis* Ferguson, 1926a: 171, by original designation.

#### Description.

Body length: 8 mm. Broadly built flies with oval abdomen. Head wider than thorax. Face convex in profile; wider than an eye. Lateral oral margins not produced. Antennal fossa about as wide as high. Vertex flat. Occiput rather wide, dorsally strongly widened. Eye bare. Eye margins in male not converging at level of frons; with mutual distance about five times the width of antennal fossa. Antenna longer than distance between antennal fossa and anterior oral margin; basoflagellomere longer than scape, bare, bifurcate, with dorsal branch narrower and shorter than ventral branch, ventral branch strongly curved; arista well-developed. Postpronotum pilose. Scutellum semicircular; without calcars. Anepisternum moderately sulcate; pilose anteriorly and posteriorly, bare medially. Anepimeron entirely pilose. Katepimeron convex; bare. Wing: vein R4+5 with posterior appendix; vein M1 perpendicular to vein R4+5; postero-apical corner of cell r4+5 widely rounded; crossvein r-m located around basal 1/4 of cell dm. Abdomen oval, about 1.5 times as long as wide. Tergites 3 and 4 fused. Male genitalia: phallus unfurcate; epandrium without ventrolateral ridge; surstylus with long posterior process and wide anterior lamella. Female unknown.

#### Diagnosis.

Basoflagellomere bifurcate. Vein R4+5 with posterior appendix.

#### Discussion.

Although Ferguson (1926a) argued that the furcate antenna provides insufficient basis for erecting a new genus for *Microdon alcicornis*, Hull (1945) decided to erect *Cervicorniphora* for this species, as a subgenus of *Microdon*. Cheng and Thompson (2008) also considered this genus-group as a subgenus of *Microdon*. The phylogenetic analysis of morphological characters by Reemer and Ståhls (in press) did not provide many clues as to the taxonomic affinities of this taxon, although it seems clear that it is not related to other taxa in which the male has a furcate basoflagellomere. As the characters of *Cervicorniphora* (e.g. phallus not furcate) do not fit in the concept of *Microdon* s.s. (phallus furcate near base) as defined in the current paper, *Cervicorniphora* is here raised to genus rank, to avoid disrupting the monophyly of *Microdon*.

The female is unknown. In most other microdontine taxa in which the male has a furcate basoflagellomere (e.g. *Carreramyia*, *Schizoceratomyia*), the female has an unfurcate basoflagellomere. So, the possibility that the female of *Cervicorniphora* has unfurcate antennae should be taken into account.

#### Diversity and distribution.

Described species: 1. Australia: New South Wales, Queensland and Tasmania (Ferguson 1926a).

### Chrysidimyia

Hull

http://species-id.net/wiki/Chrysidimyia

Figs 63–67

Chrysidimyia Hull, 1937b: 116. Type species: *Chrysidimyia chrysidimima* 1937: 116, by original designation. Name emended by Thompson et al. (1976).

#### Description.

Body length: 8–10 mm. Metallic green to bluish flies (legs may be yellowish), entire body densely and coarsely punctate, mimics of Chrysididae (Hymenoptera). Head about as wide as thorax. Face convexly produced in profile; about as wide as an eye. Lateral oral margins produced. Vertex flat. Occiput ventrally narrow, dorsally strongly widened. Eye densely pilose. Eyes in male with mutual distance smaller than width of antennal fossa. Antennal fossa twice as wide as high, dorsally covered by ‘shelf-like’ extension of frons. Antenna longer than distance between antennal fossa and anterior oral margin; basoflagellomere longer than scape, oval; bare. Postpronotum pilose. Notal wing lamina strongly developed; partly overlapping membranes around wing insertion. Scutellum semicircular; with calcars. Anepisternum moderately sulcate; with bare part limited to ventral half. Anepimeron entirely pilose. Katepimeron flat; bare. Katatergum carinate. Wing: vein R4+5 with posterior appendix; vein M1 perpendicular to vein R4+5; postero-apical corner of cell r4+5 widely rounded; crossvein r-m located around basal 1/4 of cell dm. Abdomen oval, about 1.5 times as long as wide. Posterior margin of tergite 1 angular. Tergites 3 and 4 fused. Male genitalia: phallus unfurcate; epandrium without ventrolateral ridge; surstylus furcate, with anterior part short and wide, posterior process long and narrow.

#### Diagnosis.

Head, thorax and abdomen metallic green or blue. Antennal fossa twice as wide as high, dorsally covered by ‘shelf-like’ extension of frons.

#### Discussion.

*Chrysidimyia* was treated as a synonym of *Microdon* by Thompson et al. (1976), but the unfurcate phallus and the phylogenetic results of Reemer and Ståhls (in press) indicate that this status cannot be maitained. Instead, the male genitalia of *Chrysidimyia* (Fig. 65) resemble those of *Laetodon* (Fig. 135); these taxa share an unfurcate phallus and a long posterior process on the surstylus. These taxa also have their metallic body colouration and pilose eyes in common. These characters may suggest a phylogenetic relationship, although this is not found by Reemer and Ståhls (in press), who recovered *Chrysidimyia* in a large polytomy. Besides, the ‘shelf-like’ extension of the frons and dense punctuation of the body are not found in *Laetodon*. For this reason, we prefer to treat the groups separately.

#### Diversity and distribution.

Described species: 1. One additional, undescribed species is known to the first author. All known records are from the Amazon region of South America, including the Guyana shield.

### *Chymophila* Macquart (subgenus, see *Microdon*). *Dimeraspis* Newman (subgenus, see *Microdon*)

### Domodon

Reemer gen. n.

urn:lsid:zoobank.org:act:EB942C77-8F1C-4095-B98A-74BF58F6E810

http://species-id.net/wiki/Domodon

Figs 68–73

#### Type species:

*Domodon zodiacus* Reemer spec. n. Type locality: Surinam, Paramaribo.

#### Description.

Body length: 6–8 mm. Moderately small flies with short antennae and oval abdomen. Head a little wider than thorax. Face convex; about as wide as or narrower than an eye. Lateral oral margins weakly produced. Vertex convexly produced, more or less shining, sparsely pilose, almost bare on anterior half. Occiput ventrally narrow, dorsally widened. Eye bare. Eye margins in male weakly converging at level of frons, with mutual distance 3-5 times width of antennal fossa. Antennal fossa about as wide as high. Antenna longer than distance between antennal fossa and anterior oral margin; basoflagellomere as long as or longer than scape, bare. Postpronotum pilose. Scutellum semicircular; with calcars. Anepisternum sulcate; pilose anterodorsally and posteriorly, widely bare in between. Anepimeron entirely pilose. Katepimeron almost flat to convex; often with wrinkled texture; bare. Wing: vein R4+5 with posterior appendix; vein M1 perpendicular to vein R4+5; postero-apical corner of cell r4+5 rectangular, with small appendix; crossvein r-m located between basal 1/6 to 1/4 of cell dm. Abdomen oval, about 1.5 to 2 times as long as wide. Tergites 3 and 4 fused. Sternite 1 bare. Male genitalia: phallus furcate near apex, with dorsal process long and whip-like, ventral process very short; epandrium with ventrolateral ridge.

#### Diagnosis

**.** Vertex convexly produced. Abdomen oval. Vein R4+5 with posterior appendix. Tergites 3 and 4 fused. Membrane between sternites 2 and 3 much less wide than sternite 2.

#### Discussion.

All species assigned to this genus were previously undescribed or are still undescribed.

The phylogenetic analysis based on morphology places the type species (*Domodon zodiacus* sp. n.) in the same clade as *Omegasyrphus* Giglio-Tos, 1891, *Pseudomicrodon* Hull, 1937 and *Rhopalosyrphus* Giglio-Tos, 1891 (Reemer and Ståhls in press). In addition to this phylogenetic evidence, the male genitalia of these taxa are all similar in the structure of the phallus and the shape of the surstylus. Because of the oval, non-constricted abdomen, *Domodon* species superficially may seem most similar to *Omegasyrphus*, but differ from that genus by the convex and sparsely pilose vertex, the long antenna, and the medially widely bare anepisternum. With *Pseudomicrodon* it shares the convex and sparsely pilose vertex, as well as the structure of the male genitalia, but *Domodon* differs from that genus by the oval (instead of constricted) abdomen.Instead of arbitrarily assigning the species in question to one of the mentioned genera, it is here considered preferable to erect a new genus, so as to emphasize the distinctive features of this group.

#### Diversity and distribution.

Described species: 1. Surinam. Four additional, undescribed species are known by the first author from French Guyana, Surinam and Costa Rica. Probably the group is widespread in Central and South America.

#### Etymology.

The generic name is a combination of *domus* and *odon*, with the latter used as a suffix derived from *Microdon*. The Latin word *domus* is here used in the meaning of ‘dome’ and refers to the convex (dome-shaped) vertex of the species in this genus. The name is to be treated as masculine.

### Furcantenna

Cheng

http://species-id.net/wiki/Furcantenna

Figs 74 –80

Furcantenna Cheng, 2008: 29 (in Cheng and Thompson 2008). Type species: *Furcantenna yangi* Cheng, 2008: 29, by original designation.

#### Description.

Body length: 9–10 mm. Broadly built flies with very wide head, long antennae and widened hind tibiae, bee mimics. Head much wider than thorax. Face slightly convex in profile; wider than eye; laterally depressed; medially weakly carinate. Lateral oral margins not produced. Vertex produced. Occiput ventrally narrow, dorsally widened. Eye bare. Eyes in male not convergent at level of frons; separated over distance much larger than width of antennal fossa. Antennal fossa about as high as wide. Antenna much longer than height of head; basoflagellomere bifurcate at base, with ventral branch a little longer than dorsal branch, both branches entirely long pilose; arista absent. Postpronotum pilose. Anepisternum sulcate. Scutellum apicomedially sulcate. Katepisternum dorsally pilose. Metasternum developed and pilose. Wing: vein R4+5 without posterior appendix; vein M1 perpendicular to R4+5 and M; crossvein r-m located around basal 1/5 of cell dm. Hind tibia and tarsus widened. Abdomen oval. Male genitalia: phallus slightly bent dorsad, with large spherical base; phallus furcate near apex; epandrium without ventrolateral ridge; surstylus approximately oval. Females unknown.

#### Diagnosis.

Male with bifurcate basoflagellomere. Katepisternum pilose. Metasternum pilose.

#### Diversity and distribution.

Described species: 2. The type species was found in a mountainous area in southeastern China. The second known species, *Furcantenna nepalensis* sp. n., was collected in the Nepalese Himalaya at an altitude of approximately 1800 meters. The discovery of these species in these areas sheds an interesting light on the biogeography of the taxa with a furcate basoflagellomere in the male. Prior to the description of *Furcantenna*, such taxa were almost exclusively known from South America (except for the the apparently unrelated Australian *Cervicorniphora*). The occurrence of the obviously related (Reemer and Ståhls in press) *Furcantenna* in Oriental mountains on the Asian mainland could possibly be explained as a relict of a wider distribution in early eras.

### Heliodon

Reemer gen. n.

urn:lsid:zoobank.org:act:6981D4FC-AE41-45D7-942D-10707E8045CE

http://species-id.net/wiki/Heliodon

Figs 81 [Fig F13] –96

#### Type species:

*Microdon tricinctus* de Meijere, 1908: 208. Type locality: Java.

#### Description.

Body length: 8–12 mm. Moderately slender to broadly built flies with long antennae; abdomen oval, slightly tapering or basally slightly constricted; often with fasciate patterns of golden pile on thorax and abdomen, sometimes with yellow abdominal markings. Head slightly wider or slightly narrower than thorax. Face convex; narrower than to as wide as an eye. Lateral oral margins produced. Vertex flat. Occiput ventrally narrow, dorsally widened. Eye short pilose or bare. Eye margins in male converging at level of frons, with mutual distance 1.5–2 times as large as width of antennal fossa. Antennal fossa about as wide as high. Antenna about as long as distance between antennal fossa and anterior oral margin; basoflagellomere shorter than scape; bare. Postpronotum pilose. Scutellum semicircular; with calcars. Anepisternum sulcate; entirely pilose, except for small bare part ventrally. Anepimeron entirely pilose. Katepimeron convex or nearly flat; with or without wrinkled texture; bare or pilose. Wing: vein R4+5 with posterior appendix; vein M1 more or less straight, perpendicular to vein R4+5; postero-apical corner of cell r4+5 rounded or rectangular, with or without small appendix; crossvein r-m located between basal 1/6 and 1/5 of cell dm. Abdomen oval or basally constricted, 1.5-3 times as long as wide. Tergites 3 and 4 fused. Sternite 1 pilose. Male genitalia: phallus projecting little beyond apex of hypandrium, bent dorsad, furcate with furcation point from halfway to near apex, with both processes about equally long; epandrium without ventrolateral ridge; surstylus with subbasal excavation, dividing surstylus into a basal lamella and a long posterior process.

#### Diagnosis.

Vein R4+5 with posterior appendix. Postpronotum pilose. Propleuron bare. Anepisternum almost entirely pilose, at most ventrally with small bare part. Mesonotum with transverse suture incomplete. Basoflagellomere shorter than scape. Tergite 1 long: length/width ratio 1:1.4 to 1:2. Tergite 2: anterior margin less than 1.5 times as wide as posterior margin. Body not entirely metallic green or blue.

#### Discussion.

All previously described species included in this genus were originally described in the genus *Microdon*. In the most recent catalogue of Oriental Microdontinae these species were listed under that genus (Knutson et al. 1975). As *Microdon* is defined more strictly in the present paper, the species can no longer be placed in that genus, hence a new genus is erected. Three new species are described in the present paper.

#### Diversity and distribution.

Described species: 8. Oriental, ranging from Sri Lanka to Thailand, Vietnam, Java and Borneo.

#### Etymology.

The generic name is composed of the Greek words *helios* (sun) and *odon*, with the latter part used as a suffix derived from *Microdon*. The first part was chosen to emphasize the Oriental (‘where the sun rises’) distribution of the genus.

### Hypselosyrphus

Hull stat. n.

http://species-id.net/wiki/Hypselosyrphus

Figs 97 –102

Hypselosyrphus Hull, 1937a: 21. Type species *Hypselosyrphus trigonus* Hull, 1937: 21, by original designation.

#### Description.

Body length: 7–10 mm. Stingless bee mimicking flies with short to moderately long antennae and oval to triangular abdomen. Head slightly wider than thorax. Face convex; narrower than an eye. Lateral oral margins not produced. Vertex narrow, in most species convexly produced and shining, flat in some species. Occiput narrow over entire length, except ventrally strongly widened in *Hypselosyrphus ulopodus*. Eye with short, sparse pile. Eye margins in male strongly converging at level of frons, with mutual distance smaller than width of antennal fossa, except 3 times as wide in *Hypselosyrphus ulopodus*. Antennal fossa about as wide as high. Antenna as long as or shorter than distance between antennal fossa and anterior oral margin; basoflagellomere shorter to longer than scape, oval; bare. Postpronotum pilose. Scutellum semicircular, triangular or apicomedially sulcate; without calcars. Anepisternum without or with weak sulcus; pilose anterodorsally and posteriorly, widely bare in between. Anepimeron entirely pilose. Katepimeron convex; bare. Wing: vein R4+5 without posterior appendix; vein M1 perpendicular to vein R4+5; postero-apical corner of cell r4+5 rectangular, with small appendix; crossvein r-m located between basal 1/8 to 1/4 of cell dm. Abdomen oval or kite-shaped, 1.2 to 2 times as long as wide. Tergites 3 and 4 fused. Sternite 1 pilose. Male genitalia: phallus furcate near apex, with dorsal process in some species a little longer than ventral process; epandrium with or without ventrolateral ridge.

#### Diagnosis.

Vein R4+5 without posterior appendix. Crossvein r-m located between basal 1/8 and 1/4 of cell dm. Subcostal vein joins costal vein after level of crossvein r-m. Postpronotum pilose. Abdomen oval or kite-shaped (tergite 2 wide, subsequent tergites triangularly narrowing). Antenna as long as or shorter than distance between antennal fossa and anterior oral margin. Basoflagellomere not furcate. Occiput narrow in dorsal half (usually also in ventral half, except in *Hypselosyrphus ulopodus* Hull, 1944).

#### Discussion.

When Hull (1937a) erected the genus *Hypselosyrphus* for his species *trigonus*, he mentioned its similarity to *Ubristes* Walker, 1852 species, without clearly stating the differences. In his key to the groups of Microdontinae, Hull (1949) separated these taxa by the absence (*Hypselosyrphus*) or presence (*Ubristes*) of an appendix on vein R4+5. This character serves well to separate *Hypselosyrphus* from *Ubristes* s.s. as defined in the present paper, and almost always for *Stipomorpha* (appendix on vein R4+5 seldomly missing), which was included in *Ubristes* until now. In later keys and catalogues, *Hypselosyrphus* was treated as a junior synonym of *Ubristes* (Thompson 1969, Thompson et al. 1976). Cheng and Thompson (2008) also considered *Hypselosyrphus* (and *Stipomorpha* Hull, 1945) synonymous with *Ubristes*, but nevertheless differentiated the groups in their key. They consider abdominal shape to be diagnostic: oval or rectangular in *Ubristes*, short, almost equilaterally triangular in *Hypselosyrphus*, much longer, isosceles triangular in *Stipomorpha*. As there are many varieties in abdominal shape among the the taxa involved, it is hard to decide where to draw the line. Other characters are necessary to distinguish these taxa satisfyingly (see key and diagnoses).

#### Diversity and distribution.

Described species: 11. Descriptions of five additional species are in preparation by the first author. *Hypselosyrphus* is known from Panama, the Amazon region and southern Brazil. Considering the small number of specimens known, it seems likely that the genus is widespread in tropical South America.

### Indascia

Keiser

http://species-id.net/wiki/Indascia

Figs 103 –118

Indascia Keiser, 1958: 221. *Type species*: *Ascia brachystoma* Wiedemann, 1824: 33, by original designation.

#### Description.

Body length: 4–10 mm. Small, slender flies with more or less constricted abdomen. Head wider than thorax. Face convex in profile; narrower than to wider than an eye. Lateral oral margins not produced. Vertex flat. Occiput ventrally narrow, dorsally strongly widened. Antennal fossa about as wide as high. Eye bare. Eye margins in male parallel, not converging at level of frons. Antenna shorter to longer than distance between antennal fossa and anterior oral margin. Basoflagellomere as long as to longer than scape, 1.5 to 5 times as long as wide; parallel-sided or with dorsal margin somewhat concave; bare. Postpronotum pilose. Mesoscutum with transverse suture complete. Scutellum semicircular, apex may be slightly acute; without or with very small calcars. Anepisternum convex or sulcate; entirely pilose or with bare part limited to ventral half. Anepimeron entirely pilose. Katepimeron (moderately) convex; bare. Wing: vein R4+5 with or without posterior appendix; vein M1 perpendicular to vein R4+5 and vein M; postero-apical corner of cell r4+5 rectangular, with small appendix; crossvein r-m located within basal 1/4 of cell dm, sometimes very close to base. Abdomen elongate, at least 3 times as long as wide; constricted, with narrowest point at posterior margin of tergite 2 and widest point at tergite 4. Tergites 3 and 4 not fused. Male genitalia: phallus furcate, with furcation point in distal half; epandrium without ventrolateral ridge; surstylus furcate, with anterior part short, posterior part about twice as long.

#### Diagnosis.

Abdomen constricted. Postpronotum pilose. Mesoscutum with transverse suture complete. Katepimeron bare. Frons laterally without concave area.

#### Discussion.

Originally this genus was included in the tribe Sphegini, as part of a subfamily Cheilosiinae (Keiser 1958). Thompson (1969) correctly recognized that it belongs to the Microdontinae, where it has remained since.

Originally, *Indascia* was based on two species with short antennae and without a posterior appendix on vein R4+5 (Keiser 1958). In two of the species included in the phylogenetic analyses of Reemer and Ståhls (in press) the antennae are long and the appendix on vein R4+5 is present (*Indascia gigantica* sp. n. and *Indascia spathulata* sp. n.). Both characters are also found in additional undescribed species known to the first author. Therefore, these characters are considered not to be of diagnostic value for this genus.

Superficially, species of *Indascia* look similar to those of *Paramicrodon* de Meijere, 1913 (as noticed by Cheng and Thompson 2008). For discussion on similarities with *Paramixogaster* Brunetti, 1923 see there.

#### Diversity and distribution.

Described species: 4. At least four undescribed species are known to the first author. The genus appears to be strictly Oriental, with species known from India, Sri Lanka, Pakistan, Thailand and Vietnam. The origin of the type specimens of the type species (‘India orientalis’) is not exactly known.

### Kryptopyga

Hull

http://species-id.net/wiki/Kryptopyga

Figs 119 –131

Kryptopyga Hull, 1944a: 129. Type species: *Kryptopyga pendulosa* Hull, 1944a: 130, by original designation.

#### Description.

Body length: 12–14 mm. Large flies with long antennae (pilose in male) and oval abdomen, which may be constricted basally. Head wider than thorax. Face in profile more or less straight, ventrally produced below eye margin; wider than eye. Lateral oral margins weakly produced. Vertex strongly swollen. Occiput narrow ventrally, strongly widened dorsally. Eye bare. Eyes in male not converging at level of frons; mutual distance about 5 times width of antennal fossa. Antennal fossa about as high as wide. Antenna longer than height of head. Basoflagellomere 3.5-4 (male) or 2.5 (female) times as long as scape; with long pilosity in male, bare in female. Postpronotum pilose. Mesoscutum with transverse suture incomplete. Scutellum semicircular, without calcars. Anepisternum with deep sulcus; pilose anterodorsally and posteriorly, widely bare in between. Anepimeron entirely pilose. Katepimeron convex; with or without wrinkled texture; with rows of microtrichia. Wing: vein R4+5 with posterior appendix; vein M1 in anterior half with outward angle; postero-apical corner of cell r4+5 rectangular, with small appendix; crossvein r-m located between basal 1/6 and 1/5 of cell dm. Abdomen either oval or somewhat constricted at base, in the latter case with tergite 4 curved downward and more or less perpendicular to tergite 2. Tergites 3 and 4 not fused, able to articulate independently. In male *Kryptopyga pendulosa*, sternite 4 is covered by the genital capsule and therefore not visible without removing genitalia, while the lateral margins of tergite 3 are strongly curved and ‘tucked away’ under sternite 3 (Fig. 123). Male genitalia: phallus slender, furcate near apex, basally complexly bent into curves, interconnected by a membrane; epandrium without ventrolateral ridge; surstylus approximately oval.

#### Diagnosis.

Vein R4+5 with posterior appendix. Postpronotum pilose. Propleuron bare. Mesonotal transverse suture incomplete. Tergites 3 and 4 not fused, able to articulate independently. Anepisternum widely bare of pile (but with microtrichia) medially, also on dorsal half. Male basoflagellomere with long pile.

#### Discussion.

Hull (1944a) erected the genus and assigned one species to it: *Kryptopyga pendulosa* Hull, 1944. He considered it close to the African genus *Ptilobactrum* Bezzi, 1915 because of the long pile on the basoflagellomere, but considered it distinct because of the subpetiolate abdomen and the remarkable structure of the 3rd and 4th abdominal segments. The pilose basoflagellomere in the male is also found in *Ceratrichomyia*, with which *Kryptopyga* also shares the swollen vertex and dorsal occiput, and the unfused tergites 3 and 4. The male genitalia, however, are quite different, and in *Kryptopyga* the mesonotal transverse suture is incomplete.

Together with the Nearctic *Microdon craigheidi* Walton, 1912, *Kryptopyga* is the only known taxon of Microdontinae in which the phallus is not simply curved between base and apex, but complexly bent into a couple of curves basally, interconnected by a membrane (compare Fig. 131 with Fig. 232). Despite this common character, there is no reason to suspect a closer relationship between these taxa.

The abdomen in *Kryptopyga pendulosa* is much more modified than in *Kryptopyga sulawesiana* sp. n., but the latter species is nevertheless regarded as belonging to the genus based on the pilose basoflagellomere, the shape of the head, the wing venation and the structure of the male genitalia, in which it is all very similar to *Kryptopyga pendulosa*.

*Microdon tuberculatus* Shiraki, 1968 might also belong in *Kryptopyga*, because of its unfused tergites 3 and 4 and similarity in head shape (strongly swollen vertex and dorsal occiput, face ventrally produced below eye margin). However, only the female of this species is known, so it is unknown whether the male has long pile on the basoflagellomere and the characteristic genitalia of *Kryptopyga*. Therefore, this species is presently left unclassified. As de Meijere (1913) had already used the same species name, the replacement name *shirakii* is here proposed.

#### Diversity and distribution.

Described species: 2. Indonesia: Bangka, Java and Sulawesi.

### Laetodon

Reemer gen. n.

urn:lsid:zoobank.org:act:98DA55E3-2041-4F23-B5B8-141BDF62DEC5

http://species-id.net/wiki/Laetodon

Figs 132–135

#### Type species:

*Microdon laetus* Loew, 1864: 74, by original designation. Type locality: Cuba.

#### Description.

Body length: 6–9 mm. Small, metallic green to blue flies, with long antennae and oval abdomen. Head about as wide as thorax or slightly wider. Face convex; narrower than an eye. Lateral oral margins weakly produced. Vertex flat. Occiput ventrally narrow, dorsally widened. Eye pilose. Eye margins in male converging at level of frons, with mutual distance 2 to 4 times as large as width of antennal fossa. Antennal fossa about as wide as high. Antenna about as long as to longer than distance between antennal fossa and anterior oral margin; basoflagellomere longer than scape, oval; bare. Postpronotum pilose. Scutellum semicircular; with calcars, which may be spatulate (widened and dorsoventrally flattened). Anepisternum weakly sulcate; pilose anteriorly and posteriorly, widely bare in between. Anepimeron entirely pilose. Katepimeron convex; smooth; bare. Wing: vein R4+5 with posterior appendix; vein M1 perpendicular to vein R4+5; postero-apical corner of cell r4+5 rectangular, with small appendix; crossvein r-m located between basal 1/6 to 1/5 of cell dm. Abdomen oval, about 1.5 to 2 times as long as wide. Tergites 3 and 4 fused. Sternite 1 pilose or bare. Male genitalia: phallus unfurcate, projecting slightly beyond apex of hypandrium; hypandrium with basal part bulb-like; epandrium without ventrolateral ridge; surstylus shallowly furcate, with long posterior process.

#### Diagnosis.

Vein R4+5 with posterior appendix. Postpronotum pilose. Abdomen oval. Anepisternum widely bare medially. Propleuron pilose. Postero-apical corner of cell r4+5 rectangular. Eye pilose.

#### Discussion.

The species included in this genus used to be placed in *Microdon* (Thompson 1981b). Morphology of the male genitalia, however, is quite distinct from that of *Microdon* as defined in the present paper: the phallus is short and unfurcate, the epandrium lacks the ventrolateral ridge. Based on these morphological differences and the phylogenetic results of Reemer and Ståhls (in press), *Laetodon* is here erected as a new genus. See *Chrysidimyia* for discussion on possible relationships with that genus.

#### Diversity and distribution.

Described species: 5. Nearctic (4 species) and Neotropical (1 species).

#### Etymology.

The generic name is composed of *laetus* and *odon*, with the first part derived from *Microdon laetus* Loew, 1864 (the type species of the genus), and the latter used as a suffix derived from *Microdon*.

### Masarygus

Brèthes

http://species-id.net/wiki/Masarygus

Figs 136 –150

Masarygus Brèthes, 1909: 441. Type species: *Masarygus planifrons* Brèthes, 1909: 442, by original designation.

#### Description.

Body length: 4–7 mm. Small, delicate flies with long antennae and flat abdomen. Head slightly to much wider than thorax. Face concave, either entirely or only laterally; wider than an eye. Mouth parts undeveloped: oral opening absent or hardly visible. Vertex more or less flat, not strongly produced or convex. Occiput ventrally narrow or widened, dorsally widened. Eye bare. Eyes in male not converging at level of frons, with mutual distance about 4 times the width of antennal fossa. Antennal fossa about as wide as high or about 1.5 times as wide as high. Antenna as long as or longer than distance between antennal fossa and anterior oral margin; basoflagellomere longer than scape, multifurcate in male (3 to 14 branches), unfurcate in female; bare; arista absent in male, present in female. Postpronotum bare. Scutellum semicircular; without calcars. Anepisternum convex; entirely with sparse, bristle-like pile. Anepimeron bare or pilose. Katepimeron convex; bare; with or without wrinkled texture. Wing: vein R4+5 without posterior appendix; vein M1 perpendicular to vein R4+5; postero-apical corner of cell r4+5 widely rounded or rectangular, with or without small appendix; crossvein r-m located very close to base of cell dm (within basal 1/10). Abdomen dorsoventrally flattened; more or less trapezoid, with lateral margins gradually widening posteriad, with largest width at tergite 4; 1.5-2.5 times as long as wide. Tergites 3 and 4 fused. Male genitalia: phallus furcate near apex, straight, projecting not or hardly beyond apex of hypandrium; epandrium without ventrolateral ridge; surstylus unfurcate, more or less oval.

#### Diagnosis.

Vein R4+5 without posterior appendix. Postpronotum bare. Antenna at least as long as distance between antennal fossa and anterior oral margin. Antenna inserted on head above dorsal eye margin.

#### Discussion.

Originally, this genus was erected as the first known member of a new family, the Masarygidae (Brèthes 1908; but journal publication was 1909, see Sabrosky 1999). Brèthes associated it with Conopidae and Scenopinidae because of the wing venation, and with Oestridae because of the reduced mouthparts. He also noted a superficial resemblance to certain Stratiomyidae. Bezzi (1910) was the first to recognize *Masarygus* as belonging to the Syrphidae and related to *Microdon*, by pointing out its resemblance to *Ceratophya* and the apparent relationship with ants (as noted by Brèthes 1908). Shannon (1925) considered *Masarygus* as a synonym of *Microdon*. Brèthes (1928) objected by pointing out that *Masarygus* differs from *Microdon* in the distinct sexual dimorphism and also in wing venation. All subsequent authors have included *Masarygus* in the Microdontinae.

*Masarygus* was the first described syrphid taxon with a furcate basoflagellomere (in the male sex only). A few other taxa with this character were described during the 20th century: *Schizoceratomyia* Carrera, Lopes & Lane, 1947, *Johnsoniodon* Curran, 1947 and *Carreramyia* Doesburg, 1966. *Masarygus*, *Schizoceratromyia* and *Johnsoniodon* were considered synonymous by Hull (1949), who also regarded “*Masarygus* as a *Rhoga* with fissicorn antennae”, without explicitly including all of these taxa in *Rhoga* (the oldest name). Papavero (1962) also considered *Masarygus*, *Schizoceratomyia* and *Johnsoniodon* synonymous, because he found that the number of branches on the basoflagellomere (four in *Masarygus planifrons*, two in the other taxa) was a species-level character rather than a generic character. Van Doesburg (1966) did not agree and considered *Masarygus* and *Schizoceratomyia* (including *Johnsoniodon*) as distinct genera, because of distinct differences in shape of head, antenna and abdomen. Thompson et al. (1976) followed the opinion of Papavero (1962). Cheng and Thompson (2008) considered *Masarygus* and *Schizoceratomyia* as distinct groups.

*Masarygus palmipalpus* sp. n. is considered related to *Masarygus planifrons* because of the following shared characters: male basoflagellomere multifurcate; base of antenna in lateral view placed above dorsal eye margin; head strongly flattened; face concave; oral opening absent; abdomen dorsoventrally flattened; gradually widening hindward, with widest point at tergite 4; phallus furcate near apex, with both processes equally long.

In addition to *Masarygus planifrons* and *Masarygus palmipalpus*, two undescribed species are considered to belong to this genus. These species are included in the phylogenetic analyses of Reemer and Ståhls (in press) under the names *Masarygus* sp. 1 and sp. 2. The latter has three branches on the basoflagellomere, the first approximately 14. Whereas sp. 1 is placed in the same clade as *Masarygus planifrons* and *Masarygus palmipalpus* by Reemer and Ståhls (in press) (based on adult morphology), sp. 2 is placed in the clade containing *Schizoceratomyia* and *Carreramyia*. Species 2 is nevertheless included in *Masarygus*, because of the following characters: basoflagellomere multifurcate and bare (instead of bifurcate and pilose as in *Schizoceratomyia*); arista absent (present in *Schizoceratomyia*); base of antenna inserted on head above dorsal eye margin (not below as in *Schizoceratomyia*); vertex not strongly produced (in contrast with *Carreramyia*); crossvein r-m located within basal 1/10 of cell dm (between basal 1/4 and 1/8 in *Schizoceratomyia*); hind tibia not swollen and without long, brush-like pile (in contrast with *Carreramyia*). Unfortunately, the genitalia of the only known specimen of *Masarygus* species 2 are lost: there is a microvial containing postabdominal segments attached to the pin, but there are no genitalia in it.

#### Diversity and distribution.

Described species: 2. Neotropical. At least two undescribed species are known to occur (see *Discussion*). All species known so far, including the undescribed ones, have only been collected on one occasion.

### *Megodon* Keiser (subgenus, see *Microdon*)

### Menidon

Reemer gen. n.

urn:lsid:zoobank.org:act:56864290-24D2-4BEB-9FB8-5B96BCABAAFD

http://species-id.net/wiki/Menidon

Figs 151 –156

#### Type species:

*Microdon falcatus* Williston, 1887: 9. Type locality: Mexico.

#### Description.

Body length: 5–10 mm. Small, broadly built flies with long antennae and short, almost round abdomen. Head about as wide as thorax. Face convex; slighly narrower to slightly wider than an eye. Lateral oral margins not produced. Vertex flat. Occiput ventrally narrow, dorsally widened. Eye bare. Eye margins in male parallel, not converging at level of frons, with mutual distance 4 times as large as width of antennal fossa. Antennal fossa about as wide as high. Antenna longer than distance between antennal fossa and anterior oral margin; basoflagellomere longer than scape, sickle-shaped; bare. Postpronotum pilose. Scutellum semicircular; with small calcars or only with pair of small tufts of black microtrichiae posteriorly. Anepisternum without sulcus; pilose on slightly less than dorsal half, bare on slightly more than ventral half. Anepimeron entirely pilose. Katepimeron convex; bare. Wing: vein R4+5 with posterior appendix; vein M1 perpendicular to vein R4+5; postero-apical corner of cell r4+5 rectangular, with small appendix; crossvein r-m located between basal 1/8 and 1/10 of cell dm. Abdomen approximately round, 1 to 1.2 times as long as wide. Tergites 3 and 4 fused. Sternite 1 bare. Male genitalia: phallus straight, furcate near apex, with both processes about equally long; hypandrium without apical part; epandrium without ventrolateral ridge; surstylus furcate, with anterior lobe small and narrow, posterior lobe larger and wider.

#### Diagnosis.

Basoflagellomere sickle-shaped: curved upward. Anepisternum bare on ventral half. Cell r4+5 with postero-apical corner rectangular. Sternite 1 bare.

#### Discussion.

This is the only one known taxon among the Microdontinae in which the apical part of the hypandrium is entirely lacking (Fig. 156). Among the Neotropical taxa, this taxon is unique in the sickle-shaped basoflagellomere. The latter character also occurs to some extent in some Nearctic (*Microdon adventitus*, *Microdon globosus*)and Old World taxa (some *Archimicrodon*, *Myiacerapis*, *Oligeriops* Hul, 1937), but these differ from *Menidon* in several other important characters, such as a furcate phallus (unfurcate in *Oligeriops*) and absence of apical part of hypandrium (present in all other Microdontinae). These morphological singularities, combined with the phylogenetic results of Reemer and Ståhls (in press) (sister of (*Piruwa* + *Paramicrodon*)), are reasons to place *Microdon falcatus* in its own genus. Thompson (2007) clarified the taxonomy of the type species, which has several synonyms.

#### Diversity and distribution.

Described species: 1. Central and South America. Unpublished molecular evidence suggests that more than one species is involved, but this needs further study.

#### Etymology.

The generic name is a combination of the Greek words *mene* (moon) and *odon*, with the latter used as a suffix derived from *Microdon*. The prefix *meni-* was chosen because of the crescent-shaped basoflagellomere in the type species.

### Mermerizon

Reemer gen. n.

urn:lsid:zoobank.org:act:D8DDC5FF-1258-4C11-895C-50208995F444

http://species-id.net/wiki/Mermerizon

Figs 157–162

#### Type species:

*Mermerizon inbio* spec. n. Type locality: Costa Rica.

#### Description.

Stingless bee mimicking flies with moderately long antennae and elongate oval abdomen. Head slightly wider than thorax. Face convex; narrower than an eye. Lateral oral margins not produced. Vertex flat. Occiput ventrally narrow, dorsally widened. Eye bare. Eye margins in male parallel, not converging at level of frons, with mutual distance 3–4 times as large as width of antennal fossa. Antennal fossa about as wide as high. Antenna shorter than (may be almost as long as) distance between antennal fossa and anterior oral margin; basoflagellomere slightly shorter to longer than scape, oval; bare. Postpronotum pilose. Scutellum semicircular; without calcars. Anepisternum without sulcus; pilose on dorsal half, bare on ventral half. Anepimeron pilose on dorsal half, bare on ventral half. Katepimeron convex; bare. Wing: vein R4+5 with posterior appendix; vein M1 perpendicular to vein R4+5; postero-apical corner of cell r4+5 rectangular, with small appendix; crossvein r-m located around basal 1/10 of cell dm. Abdomen oval, 2 to 3 times as long as wide. Tergites 3 and 4 fused. Sternite 1 pilose. Male genitalia: phallus slightly bent dorsad, furcate near apex, with dorsal process at least twice as long as ventral process; hypandrium with bulb-like base; epandrium without ventrolateral ridge.

#### Diagnosis.

Vein R4+5 with posterior appendix. Postero-apical corner of cell r4+5 rectangular, with small appendix. Postpronotum pilose. Propleuron bare. Membrane between sternites 2 and 3 less wide than sternite 2. Abdomen oval. Anepisternum bare on ventral half, pilose on dorsal half, except for small median bare part on dorsal half. Anepimeron bare on ventral half. (Male: eye margins parallel at level of frons, not converging).

#### Discussion.

The species of this genus are obvious mimics of stingless, *Trigona*-like bees in their tawny colouration and long pilose hind tibiae. At first sight they may be confused with *Hypselosyrphus*, *Rhoga*, or *Stipomorhpa*. From the first two genera, *Mermerizon* can be distinguished by the presence of a posterior appendix on vein R4+5, from *Stipomorpha* by the absence of a wide membrane between sternites 2 and 3. A presently undescribed Argentinian species lacks the long pilosity of the hind tibiae and does not seem to mimic these bees. Instead, it resembles *Paragodon* Thompson, 1969 and *Surimyia* Reemer, 2008 in general habitus, but is easily told apart by the presence of a posterior appendix on vein R4+5 and the male genitalia, which are very similar to those of the other two *Mermerizon* species.

#### Diversity and distribution.

Described species: 1. Descriptions of two additional species are in preparation by the first author. Neotropical (presently known from Costa Rica and Argentina).

#### Etymology.

The generic name is derived from the ancient Greek verb *mermerizo*, meaning ‘to deliberate’ or ‘to ponder’. This name was chosen because it took some deliberation before making the decision that a new genus was to be erected for the involved species. The name is to be treated as masculine.

### Metadon

Reemer gen. n.

urn:lsid:zoobank.org:act:A00DBDC3-F8BE-4A89-A421-04903BC81B1D

http://species-id.net/wiki/Metadon

Figs 163 –175

#### Type species:

*Microdon wulpii* Mik, 1899: 143. Type locality: Indonesia, Sumatra. Replacement name for *Microdon apicalis* Wulp, 1892: 29 (preoccupied by Walker, 1858).

#### Description.

Body length: 7–21 mm. Slender to moderately broadly built flies with oval abdomen and long antennae. Head slightly wider than thorax. Face almost straight to convex in profile; narrower to wider than an eye. Lateral oral margins produced or not produced. Vertex flat. Occiput ventrally narrow, dorsally widened. Eye bare. Eye margins in male converging at level of frons, with mutual distance 2-3 times as large as width of antennal fossa. Antennal fossa about as wide as high. Antenna longer than distance between antennal fossa and anterior oral margin; basoflagellomere shorter than scape; bare. Postpronotum pilose. Scutellum semicircular; with or without calcars. Anepisternum sulcate; entirely pilose, except sometimes with small bare part ventrally (only known exception: *Metadon bifasciatus*, in which anepisternum is bare on entire ventral half). Anepimeron entirely pilose. Katepimeron flat to somewhat convex; smooth or with wrinkled texture; not pilose, but often with rows of microtrichia. Katatergum with oblique rows of microtrichia. Wing: vein R4+5 with posterior appendix; vein M1 more or less straight, perpendicular to vein R4+5; postero-apical corner of cell r4+5 angular to widely rounded, with or without appendix; crossvein r-m located between basal 1/7 and 1/4 of cell dm. Abdomen oval, 1.5-2.5 times as long as wide. Tergites 3 and 4 fused. Sternite 1 pilose. Male genitalia: phallus projecting not or little beyond apex of hypandrium (except projecting well beyond apex of hypandrium in *Metadon bifasciatus*), bent dorsad, furcate in apical half, with both processes about equally long (except ventral process much longer in *Metadon bifasciatus*); epandrium with or without ventrolateral ridge; surstylus unfurcate, sometimes with long posterior process.

#### Diagnosis.

Body never metallic green or blue. Vein R4+5 with posterior appendix. Abdomen oval, longer than wide but less than 2.5 times as long as wide. Postpronotum pilose. Anepisternum with bare part limited to ventral half, or entirely pilose. Antenna longer than distance between antennal fossa and anterior oral margin. Basoflagellomere shorter than or as long as scape. Tergite 1 short: length/width ratio 1:25 or less.

#### Discussion.

All included species (except the ones here described) were originally described in the genus *Microdon*. However, the morphology of *Metadon* is distinct. Characters that separate these taxa in all examined species (except *Metadon bifasciatus*, see below) are: anepisternum (almost) entirely pilose; phallus projecting not or only little beyond apex of hypandrium; aedegus furcate in apical half. Additional characters for distinguishing *Metadon* from *Microdon* (that may not work for all species) are: katepimeron more or less flat, with wrinkled texture; katatergum with oblique rows of microtrichia. In general, the abdomen of *Metadon* species is more elongate than that of *Microdon* species.

The East Palaearctic species *Metadon bifasciatus* Matsumura, 1916 is aberrant in certain characters. In this species the bare part of the anepisternum reaches up to about half the height of the sclerite. In addition, the genitalia are aberrant as the ventral aedeagal process is much longer than the dorsal process (Fig. 172), a character not known from any other species of Microdontinae. Nevertheless, this species is placed in *Metadon* because of the elongate abdomen and the oblique rows of microtrichia on the katatergum. This is supported by the results of Reemer and Ståhls (in press). As the Chinese species *Microdon brunneipennis* Huo, Ren & Zheng, 2007 and *Microdon pingliensis* Huo, Ren & Zheng, 2007 and *Microdon spuribifasciatus* Huo, Ren & Zheng, 2007 are similar to *Microdon bifasciatus*, the characters as mentioned may also be valid for those species.

*Metadon* is erected as a new genus distinct from *Microdon* in order to facilitate distinction between these apparently monophyletic groups. Results of phylogenetic analyses by Reemer and Ståhls (in press) support this decision.

#### Diversity and distribution.

Described species: 42. About half of the species (22) are described from the Oriental region. Several undescribed species from this region were seen by the first author in different collections. From the Afrotropical region, 14 species are described, remarkably none of which is from Madagascar. Four species are known from the Palaearctic region. These seem to form a closely related species group, all related to *Microdon bifasciatus*, restricted to eastern China, Korea and Japan. Two species are known from the Aru Islands off the southwest coast of New Guinea (these were collected by Alfred Russel Wallace in 1857, to be described by Walker 1858). These are the only known records of this group from the Australian region.

#### Etymology.

The generic name is a combination of the ancient Greek words *meta* and *odon*, with the latter used as a suffix derived from *Microdon*. The prefix *meta* is used in the sense of ‘near’ or ‘close’, in order to indicate the resemblance in habitus to *Microdon* s.s. It is a masculine name.

### *Microdon* Meigen

Figs 176–240

*Microdon* Meigen, 1803: 275. Type species: *Musca mutabilis* Linnaeus, 1758: 592, by monotypy.

*Aphritis* Latreille, 1804: 193. Type species: *Aphritis auropupescens* Latreille, 1805, by subsequent monotypy. See Cheng and Thompson (2008) for synonymy.

*Colacis* Gistel, 1848: x. New name for *Microdon* Meigen. See Cheng and Thompson (2008) for synonymy.

*Scutelligera* Spix, 1824: 148. Type species: *Scutelligera ammerlandia* Spix, 1824: 124, by monotypy. See Cheng and Thompson (2008) for synonymy.

*Parmula* Heyden, 1825: 589. Type species: *Parmula cocciformis* Heyden, 1825: 589, by monotypy. See Cheng and Thompson (2008) for synonymy.

*Scutigerella* Haas, 1924: 148. Misspelling of *Scutelligera* Spix, 1824. See Cheng and Thompson (2008) for synonymy.

**Subgenera (see separate accounts below)**

*Chymophila* Macquart, 1834

*Dimeraspis* Newman, 1838 (= *Mesophila* Walker, 1849, **syn. n.**)

*Megodon* Keiser, 1971

*Microdon* s.s.

*Myiacerapis* Hull, 1949

*Syrphipogon* Hull, 1937

**Species groups (see under *Microdon* s.l., after subgenera)**

*craigheadii*-group

*erythros*-group

*mirabilis*-group

*tarsalis*-group

*virgo*-group

**Unplaced species (see under *Microdon* s.l., after subgenera)**

*Microdon amabilis* Ferguson, 1926

*Microdon carbonarius* Brunetti, 1923

*Microdon macquariensis* Ferguson, 1926

*Microdon nigromarginalis* Curran & Bryan, 1926

*Microdon pagdeni* Curran, 1942

*Microdon pictipennis* (Macquart, 1850)

*Microdon rieki* Paramonov, 1957

*Microdon trimacula* Curran, 1928

*Microdon tsara* Keiser, 1971

*Microdon unicolor* Brunetti, 1915

*Microdon waterhousei* Ferguson, 1926

### Subgenus Chymophila

Macquart

Figs 176–180

Chymophila Macquart, 1834: 485. Type species: *Chymophila splendens* Macquart, 1834: 486, by monotypy.
Chimophila Osten Sacken, 1875: 46. Misspelling.
Eumicrodon Curran, 1925a: 50. Type species: *Microdon fulgens* Wiedemann, 1830: 82, by original designation. See Cheng and Thompson (2008) for synonymy.

#### Description.

Body length: 10–16 mm. Broadly built flies with oval to round abdomen and long antennae. Head about as wide as to slightly narrower than thorax. Face convex in profile; slightly narrower to slightly wider than an eye. Lateral oral margins produced. Vertex flat. Occiput ventrally narrow, dorsally widened. Eye bare or very short pilose. Eye margins in male converging at level of frons, with mutual distance 1-3 times as large as width of antennal fossa. Antennal fossa about as wide as high. Antenna longer than distance between antennal fossa and anterior oral margin; basoflagellomere shorter than scape; bare. Postpronotum pilose. Scutellum trapezoid; with calcars. Propleuron pilose. Anepisternum with sulcus; pilose anterodorsally and posteriorly, extensively bare ventrally and medially. Anepimeron entirely pilose. Katepimeron convex; smooth; bare. Katatergum uniformly microtrichose. Wing: vein R4+5 with posterior appendix; vein M1 with outward angle, often with outward appendix, anteriorly recurrent; postero-apical corner of cell r4+5 widely rounded, with or without appendix; crossvein r-m located between basal 1/5 and 1/3 of cell dm. Abdomen oval, 1-1.5 times as long as wide. Tergites 3 and 4 fused. Sternite 1 pilose. Male genitalia: phallus projecting far beyond apex of hypandrium, bent dorsad, furcate basally, with both processes equally long and very slender; epandrium with ventrolateral ridge; surstylus with two wide lobes; subepandrial sclerite with elongate anterior projection projecting well beyond surstylus in lateral view.

#### Diagnosis.

Vein R4+5 with posterior appendix. Abdomen oval. Vein M1 of characteristic shape: with outward angle, usually with small outward appendix, anteriorly recurrent (Fig. 177). In addition to this character, this subgenus also differs from *Microdon* s.s. in the aedeagal processes being longer and more slender, and in the subepandrial sclerite projecting anteriorly well beyond the surstylus in lateral view (Fig 178–180).

#### Discussion.

Species of this group are similar in overall habitus to *Microdon* s.s. Many species have metallic colours, but some are dull black or have a ‘tiger-striped’ abdomen. Previously, this group was considered to be exclusively Neotropical (Cheng and Thompson 2008). However, several Oriental and one Japanese species are very similar to the Neotropical species in both external characters and morphology of the male genitalia.

#### Diversity and distribution.

Described species: 34. Neotropical (25 species), Oriental (7 species), Nearctic (1 species) and Eastern Palaearctic (1 species from southern Japan).

### Subgenus Dimeraspis

Newman

Figs 181–186

Dimeraspis Newman, 1838: 372. Type species: *Dimeraspis podagra* Newman, 1838, by monotypy.
Mesophila Walker, 1849: 1157. Type species: *Ceratophya fuscipennis* Macquart, 1834, by monotypy. **Syn. n.**

#### Description.

Body length: 8–12 mm. Broadly built flies with oval to round abdomen and long antennae. Head narrower than to about as wide as thorax. Face convex in profile; narrower to wider than an eye. Lateral oral margins not produced. Vertex flat. Occiput ventrally narrow, dorsally widened or narrow (only in *Microdon abditus* Thompson, 1981). Eye bare. Eye margins in male converging at level of frons, sometimes only weakly so (*Microdon adventitius* Thompson, 1981, *Microdon fuscipennis* (Macquart, 1834)) with mutual distance 2-5 times as large as width of antennal fossa. Antennal fossa about as wide as high. Antenna longer than or as long as distance between antennal fossa and anterior oral margin; basoflagellomere shorter to longer than scape; bare. Postpronotum pilose. Scutellum semicircular to trapezoid; without calcars, but large and blunt calcars may seem to be present due to strong apicomedian sulcus. Propleuron bare. Anepisternum without sulcus (or only a very weak one dorsally); pilose dorsally, extensively bare on slightly more or slighly less than ventral half. Anepimeron entirely pilose. Katepimeron more or less convex; smooth or with wrinkled texture (*Microdon fuscipennis*); bare. Katatergum uniformly microtrichose. Wing: vein R4+5 with posterior appendix; vein M1 more or less straight, perpendicular to vein R4+5, slightly recurrent; postero-apical corner of cell r4+5 rectangular, with appendix; crossvein r-m located between basal 1/7 and 1/4 of cell dm. Abdomen oval, 1-1.5 times as long as wide. Tergites 3 and 4 fused. Sternite 1 pilose or bare. Male genitalia: phallus projecting little beyond apex of hypandrium, bent dorsad, furcate apically, with both processes equally long; epandrium with ventrolateral ridge; surstylus with wide basal lobe and narrow posterior lobe.

#### Diagnosis.

Difficult to diagnose, because included species vary strongly in several key characters. See key and discussion.

#### Discussion.

This group was erected for the Nearctic *Dimeraspis podagra* Newman, 1838, a subjective synonym of *Mulio globosus* Fabricius, 1805 (Thompson 1981b). This species differs from *Microdon* s.s. in the unsulcate anepisternum, the bare propleuron, the rectangular postero-apical corner of cell r4+5, and the male genitalia: phallus apically furcate, hypandrium with bulb-like base. Some other Nearctic (and one Cuban) species are very similar in morphology of the male genitalia: *Microdon abditus* Thompson, 1981, *Microdon adventitius* Thompson, 1981, *Microdon fuscipennis* (Macquart, 1834), *Microdon marmoratus* Bigot, 1883, and *Microdon remotus* Knab, 1917. Thompson (1981b) also regarded these species as related, with the ‘*globosus* complex’ (*Microdon abditus*, *Microdon globosus*, *Microdon marmoratus*) as sister to the *fuscipennis*-group (*Microdon adventitius*, *Microdon fuscipennis*, *Microdon remotus*). These species are also similar in their overall brownish colouration and in the wing venation. The morphological similarities are here taken as a reason to include all species in *Dimeraspis*. Because of similarities in male genitalia this group might tentatively be considered related to *Archimicrodon*, *Menidon* or *Serichlamys*. However, because of considerable uncertainty, the group is here treated as a subgenus of *Microdon*.

*Mesophila* Walker, 1849 was erected for *Ceratophya fuscipennis* Macquart. As this species is here included in the older genus group *Dimeraspis*, *Mesophila* becomes a junior synonym of *Dimeraspis*.

#### Diversity and distribution.

Described species: 5. Nearctic (4 species) and West Indian (1 species from Cuba).

### Subgenus Megodon

Keiser

Figs 187–195

Megodon Keiser, 1971: 252. Type species: *Megodon stuckenbergi* Keiser, 1971: 253, by original designation.

#### Description.

Body length: 8–13 mm. Broadly built flies with oval abdomen and long antennae. Head about as wide as thorax. Face convex in profile; narrower than an eye. Lateral oral margins slightly produced. Vertex flat. Occiput narrow and parallel-sided over entire length. Eye bare. Eye margins in male converging at level of frons, with mutual distance about equal to width of antennal fossa. Antennal fossa about as wide as high. Antenna longer than distance between antennal fossa and anterior oral margin; basoflagellomere shorter than to as long as scape; bare. Postpronotum pilose. Scutellum trapezoid; with strongly developed calcars. Anepisternum weakly sulcate; pilose anterodorsally and along posterior margin, widely bare in between. Anepimeron entirely pilose. Katepimeron convex; smooth; bare. Wing: vein R4+5 with posterior appendix; vein M1 more or less straight, perpendicular to vein R4+5; postero-apical corner of cell r4+5 angular to weakly rounded, with or without appendix; crossvein r-m located around basal 1/6 of cell dm. Abdomen oval, around 1.5 times as long as wide. Tergites 3 and 4 fused. Sternite 1 pilose or bare. Male genitalia: phallus furcate near base, with processes equally long and projecting well beyond apex of hypandrium; epandrium with ventrolateral ridge; surstylus unfurcate, elongate, curved dorsad.

#### Diagnosis.

Vein R4+5 with posterior appendix. Postpronotum pilose. Abdomen oval. Anepisternum widely bare medially. Propleuron bare. Postero-apical corner of cell r4+5 more or less rectangular. Antenna longer than distance between antennal fossa and anterior oral margin. Occiput narrow and parallel-sided over entire length (not widened dorsally). First tarsomere of hind leg dorsally with wide longitudinal groove.

#### Discussion.

Keiser (1971) erected this genus to include a species with very large, cone-shaped scutellar calcars. Cheng and Thompson (2008) did not study this species and refrained from commenting on the status of the group. The first author was able to study the holotype of *Megodon stuckenbergi* Keiser, 1971, as well as some additional material.

*Megodon* is very similar in external morphology to *Microdon*. Their genitalia also share important characters, like the deeply furcate phallus, the long aedeagal processes and the presence of a ventrolateral ridge on the epandrium. There are also differences, most notably the entirely narrow and parallel-sided occiput, and the dorsal, longitudinal groove on the first tarsomere of the hind leg. The shared characters are here considered more important than the differences. Because of these considerations, combined with the phylogenetic results of Reemer and Ståhls (in press), *Megodon* is here treated as a subgenus of *Microdon*.

*Microdon planitarsus* Keiser, 1971 is here also assigned to *Megodon*, because it agrees with the diagnostic characters as described above, and its male genitalia are very similar to those of *Microdon stuckenbergi* (Figs 193, 195). In *Microdon planitarsis*, the scutellar calcars are not as large and cone-shaped as in *Microdon stuckenbergi*. This indicates that the size and shape of these calcars should not be regarded as group-defining.

#### Diversity and distribution.

Described species: 2. Madagascar. One undescribed species from Madagascar is known to the first author.

### Subgenus Microdon

Meigen s.s.

**Figures 196–205.**
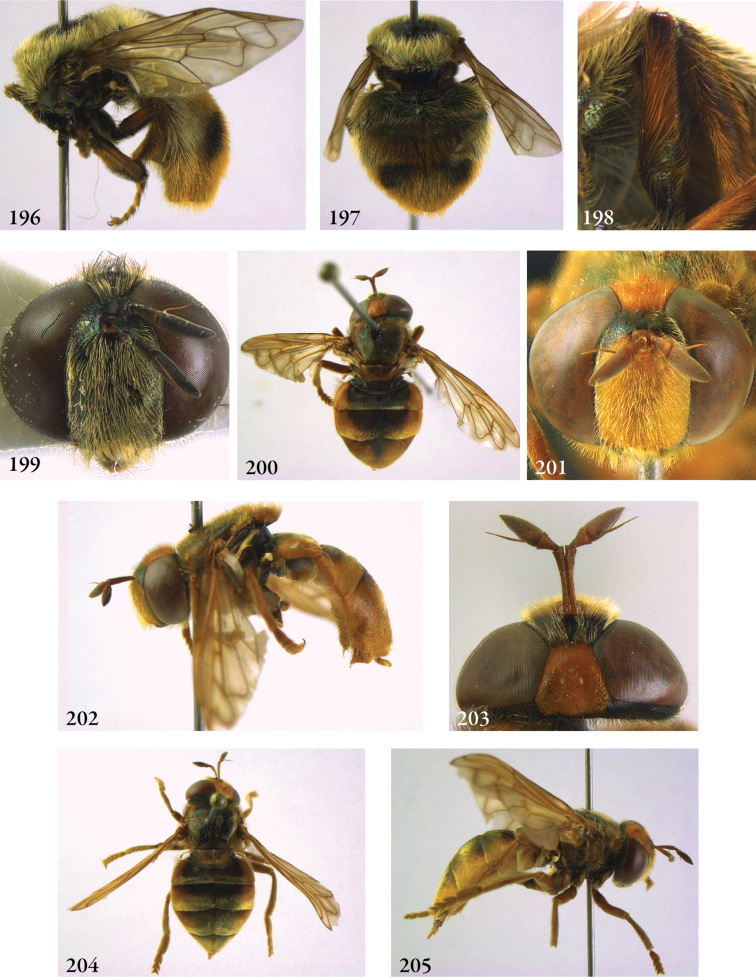
**196–199** *Microdon (Microdon) hauseri* male (holotype): **196** habitus lateral **197** abdomen dorsal **198** hind femur, frontal **199** head frontal **200–203** *Microdon (Microdon) mandarinus* male (holotype): **200** habitus dorsal **201** head frontal **202** habitus lateral **203** head dorsal. **204–205** *Microdon (Microdon) mandarinus* female (paratype): **204** habitus dorsal **205** habitus lateral.

Figs 196 [Fig F28] –214

#### Description.

Body length: 7–14 mm. Broadly built flies with oval abdomen and long antennae. Head narrower to slightly wider than thorax. Face convex in profile; slightly narrower to wider than an eye. Lateral oral margins not or weakly produced. Vertex flat. Occiput ventrally narrow to wide, dorsally widened. Eye bare. Eye margins in male converging at level of frons, with mutual distance 2-4 times as large as width of antennal fossa. Antennal fossa about as wide as high. Antenna longer than distance between antennal fossa and anterior oral margin; basoflagellomere shorter to longer than scape; bare. Postpronotum pilose. Scutellum semicircular to trapezoid; with or without calcars. Propleuron pilose. Anepisternum sulcate; pilose anterodorsally and posteriorly, widely bare ventrally and medially. Anepimeron entirely pilose. Katepimeron convex; smooth; bare. Katatergum uniformly microtrichose. Wing: vein R4+5 with posterior appendix; vein M1 more or less straight, perpendicular to vein R4+5, sometimes with slight inward angle in anterior 1/3; postero-apical corner of cell r4+5 rounded, with or without appendix; crossvein r-m located between basal 1/6 and 1/4 of cell dm. Abdomen oval, 1-1.5 times as long as wide. Tergites 3 and 4 fused. Sternite 1 pilose. Male genitalia: phallus projecting clearly beyond apex of hypandrium, bent dorsad, furcate close to base, with both processes about equally long or dorsal process longer than ventral process; epandrium with ventrolateral ridge; surstylus with two short, wide lobes.

#### Diagnosis.

Vein R4+5 with posterior appendix. Postpronotum pilose. Abdomen oval. Anepisternum extensively bare ventrally and medially. Postero-apical corner of cell r4+5 rounded. Katepimeron convex, without microtrichia. Apical crossvein M1 without outward angle. Lateral oral margins not or weakly produced.

#### Discussion.

As Cheng and Thompson (2008) wrote, this genus has remained “somewhat a catch all for various unrelated species not placed in other genera”. Many species previously placed in*Microdon* are transferred to other genera in the present paper, e.g. *Archimicrodon*, *Metadon* and *Peradon*. These classificatory changes are supported by the results of the phylogenetic analysis of combined molecular and morphological characters by Reemer and Ståhls (in press). The analysis of only morphological characters by Reemer and Ståhls (in press) included many additional species which do not obviously belong to any of the previously recognized genus groups, nor to the genera erected in the present paper. The phylogenetic results offer little or no clues as to their taxonomic affinities. As most of these species were originally described in *Microdon*, and were subsequently maintained in that genus, the pragmatic solution is here chosen to keep these taxa in *Microdon* s.l. (see below). This category should not be confused with the supposedly monophyletic *Microdon* s.s. as defined above, as *Microdon* s.l. is probably not monophyletic. For some of these taxa, genus group names are available, which are here treated as subgenera (see separate accounts). The other taxa are here placed in species groups or left unplaced. These taxa are discussed below.

Unlike the other species groups discussed below, the *virgo* species group is considered to belong within *Microdon* s.s.

#### Diversity and distribution.

Described species: 62. Occurs in Nearctic (13 species), Neotropical (14), Oriental (9) and Palaearctic (26) regions.

### Subgenus Myiacerapis

Hull

Figs 215–220

Myiacerapis Hull, 1949: 309. Type species: *Microdon villosus* Bezzi, 1915: 135, by original designation.

#### Description.

Body length: 12 mm. Broadly built flies with bee-like pilosity and long antennae. Head wider than thorax. Face convex; wider than an eye. Lateral oral margins not produced. Vertex flat. Occiput ventrally narrow, dorsally widened. Eye bare or very short and sparsely pilose. Eye margins in male hardly converging at level of frons, with mutual distance about 5 times as large as width of antennal fossa. Antennal fossa about as wide as high. Antenna longer than distance between antennal fossa and anterior oral margin; basoflagellomere longer than scape; bare. Postpronotum pilose. Scutellum semicircular; without calcars. Anepisternum weakly sulcate; pilose anteriorly and posteriorly, widely bare in between. Anepimeron entirely pilose. Katepimeron convex; with wrinkled texture; bare. Wing: vein R4+5 with posterior appendix; vein M1 slightly recurrent, but more or less perpendicular to vein R4+5; postero-apical corner of cell r4+5 rounded, without appendix; crossvein r-m located around basal 1/4 of cell dm. Abdomen oval, about 1.5 times as long as wide. Tergites 3 and 4 fused. Sternite 1 pilose. Male genitalia: phallus furcate, with furcation point near base; epandrium with ventrolateral ridge; surstylus unfurcate.

#### Diagnosis.

Abdomen oval, about 1.5 times as long as wide. Vein R4+5 with posterior appendix. Postpronotum pilose. Proepimeron bare. Antenna longer than distance between antennal fossa and anterior oral margin. Basoflagellomere longer than scape. Anepisternum with bare ventromedian part extending to dorsal half. Sternite 1 pilose. Scutellum without calcars.

#### Discussion.

*Myiacerapis* was described as a subgenus of *Microdon*. In morphology it is quite similar to *Microdon* s.s. as defined in the present paper, also in the male genitalia (deeply furcate phallus with equally long processes, epandrium with ventrolateral ridge). However, unlike *Microdon* s.s. it has a bare proepimeron (pilose in *Microdon* s.s.) and a wrinkled texture of the katepimeron. Therefore, it is not placed in *Microdon* s.s. here, but in *Microdon* s.l, in awaitance of better understanding of its phylogenetic affinities.

#### Diversity and distribution.

Described species: 1. Africa (Uganda). An undescribed species is known from South Africa (coll. BMNH).

### Subgenus Syrphipogon

Hull

Figs 221–222

Syrphipogon Hull, 1937b: 120. Type species: *Syrphipogon fucatissimus* Hull, 1937: 120, by original designation.

#### Description.

Body length: 25–28 mm. Very large flies with oval abdomen and long, colourful pilosity. Mimics of orchid bees of the genus *Eulaema* (Euglossidae). Head about as wide as thorax. Face more or less straight in profile; narrower than an eye; on ventral half with very long, thick and dense pile, resembling a beard (‘mystax’). Eye margins in male converging at level of frons, with mutual distance about twice as large as width of antennal fossa. Antennal fossa about as wide as high. Antenna longer than distance between antennal fossa and anterior oral margin; basoflagellomere shorter than scape, oval, about four times as long as wide, bare. Postpronotum bare. Scutellum trapezoid; with very large, cone-shaped calcars. Anepisternum sulcate; pilose anterodorsally and posteriorly, widely bare medially. Anepimeron entirely pilose. Katepimeron convex; smooth; bare. Wing: vein R4+5 with posterior appendix; vein M1 straight, perpendicular to vein R4+5; postero-apical corner of cell r4+5 widely rounded, without appendix; crossvein r-m located around basal 2/7 of cell dm. Abdomen oval, about 1.3 times as long as wide. Tergites 3 and 4 fused. Sternite 1 pilose. Male genitalia: phallus furcate, with furcation point near base, both processes about equally long, curved dorsad, projecting well beyond apex of hypandrium; epandrium without ventrolateral ridge; surstylus shallowly furcate, with two short and wide lobes.

#### Diagnosis.

Body length more than 20 mm. Face with very long, thick and dense pile, resembling a beard (‘mystax’).

#### Discussion.

Hull (1937b) erected *Syrphipogon*, and considered it related to *Microdon*. Steyskal (1953) referred to Hull’s description in his own description of an apparently very similar species (*Microdon gaigei* Steyskal, 1953), but he considered the differences with *Microdon* insufficient for generic status. In external characters and male genitalia *Microdon* and *Syrphipogon* are quite similar. For that reason, *Syrphipogon* is here still treated as a subgenus of *Microdon*.

The differences between the two species of *Syrphipogon* are not very convincing when comparing the description of Steyskal (1953), based on a female, with the holotype of *Syrphipogon fucatissimus* Hull, 1937, a male. The differences as noted by Steyskal (1953) may be due to sexual dimorphism, but in order to establish this, the type of *Microdon gaigei* needs to be examined.

#### Diversity and distribution.

Described species: 2. Neotropical. Only two specimens are known: one from Panama and one from “South America”.

### *Microdon* s.l. (species groups and unplaced species)

***craigheadii*-group.**

Only one species is included in this group: *Microdon craigheadii* Walton, 1912. This slender, metallic green Nearctic species is similar in habitus to *Laetodon* and many species of *Microdon* s.l. From these groups, *Microdon craigheadii* differs in the structure of the basal part of the phallus: the part of the phallus connecting the basal spherical part with the apical part is complexly curved (Fig. 232). This is a very unusual structure in Microdontinae, only found in this species and in *Kryptopyga*. In other genitalic structures (phallus deeply furcate, epandrium with ventrolateral ridge) as well in external morphology, *Microdon craigheadii* is very similar to *Microdon* s.s. Because of the peculiar morphology of the genitalia, the species is placed in a separate species group within *Microdon* s.l.

***erythros*-group.**

In overall habitus and many external characters, the species of this group remind of both *Microdon* s.s. and *Metadon*. Placement in *Microdon* s.s. is contradicted by the phallus being furcate apically (Fig. 233), whereas placement in *Metadon* is contradicted by the extensively bare anepisternum. As the phylogenetic analysis of morphological characters (Reemer and Ståhls in press) provides no information on the affinities of *Microdon erythros* Bezzi, 1908, this species is placed in *Microdon* s.l., along with the similar *Microdon luteiventris* Bezzi, 1915.

***mirabilis*-group.**

The species of this Neotropical group have contrasting yellow and black colour patterns on the wings, combined with remarkably long hind legs, evoking a resemblance to certain Pompilidae (Hymenoptera). Apart from this, they differ from *Microdon* s.s. in the bare propleuron and the aedeagal processes projecting hardly beyond the apex of the hypandrium.

Apart from *Microdon mirabilis*, this group includes *Microdon bertonii* Bezzi, 1910 (= *Microdon arcuatus* Curran, 1941, **syn. n.**) and *Microdon iheringi* Bezzi, 1910. The species seem to differ only in colouration of wings, legs and abdomen. However, quick glances in museum collections (e.g. USNM) suggest that intermediate specimens exist. This indicates that species taxonomy of this group needs further attention.

Bezzi (1910) wrote that he had two male specimens *Microdon iheringi* in his collection, which he both considered as ‘cotypes’. The collection of the MCSN (Milan) presently holds only one specimen (a male), which was examined by the first author. It is uncertain whether the other specimen still exists. In order to assure stability of this taxon, the specimen in the MCSN-collection is here designated as lectotype. Label information is as follows: label 1: “5695”; label 2: “S. Paulo / Brasile / 26.X.06 / Hering”; label 3: “iheringi”; label 4 (red): “LECTOTYPE / *Microdon iheringi* / Bezzi, 1910 / Des. M. Reemer 2009”.

***tarsalis*-group.**

This group only includes the Afrotropical species *Microdon tarsalis* Hervé-Bazin, 1913 and its synonym *Microdon bequaerti* Curran, 1929 (**syn. n.**). In the phylogenetic analysis of morphological characters (Reemer and Ståhls in press) this species was recovered in the *Microdon* s.l. clade, but its exact relationship with the other groups in this clade were unresolved. This group differs from *Microdon* s.l. in e.g. the entirely narrow occiput, the short and characteristically shaped phallus with the dorsal process longer than the ventral process, and the absence of a ventrolateral ridge on the epandrium. Besides, there is a patch of pile with hook-shaped apexes on the hind basitarsus dorsally on its inner surface.

In overall habitus (including swollen hind basitarsus), *Microdon tarsalis* is remarkably similar to the Nearctic *Microdon (Dimeraspis) abditus* Thompson, 1981 but considering the differences in male genitalia this similarity is probably merely superficial. These differences are: phallus with dorsal process longer than ventral process (equally long in *Microdon abditus*), epandrium without ventrolateral ridge (with ventrolateral ridge in *Microdon abditus*), surstylus simple shaped, without distinct posterior process (with posterior process in *Microdon abditus*).

***virgo*-group.**

**Figure 229–236.**
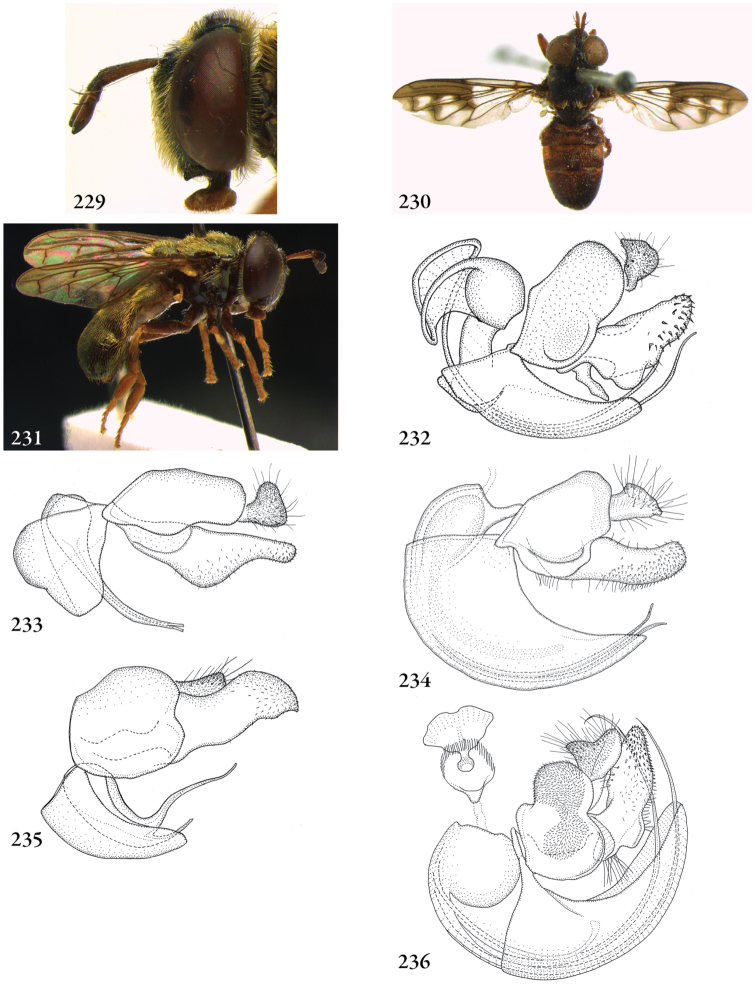
**229** *Microdon* (*virgo*-group) *rufiventris* (Surinam, coll. RMNH), head lateral **230–231** *Microdon* (s.l.), male, habitus: **230** *Microdon pictipennis* (Australia, coll. MNHN) **231** *Microdon tsara* (holotype) **232–236** *Microdon* (s.l.), male genitalia: **232** *Microdon craigheadi* (USA, Georgia, coll. RMNH) **233** *Microdon erythros* (Congo, coll. RMNH) **234** *Microdon bertonii* (Brazil, coll. RMNH) **235** *Microdon tarsalis* (holotype) **236** *Microdon rufiventris* (Surinam, coll. RMNH).

This group consists of Neotropical metallic green, blue or bronze flies, sometimes partly reddish. It is differentiated from *Microdon* s.s. in the key by the bare propleuron and the strongly produced lateral oral margins, of which the anterolateral corners are distinctly angular (Fig. 229). The latter character is presented with some hesitation, as it is uncertain whether it works for all species. Possibly, certain species here placed in *Microdon* s.s. also belong in this group. Therefore, the *virgo*-group is here considered as a species group within *Microdon* s.s., instead of within *Microdon* s.l. As it is presently uncertain which species should be assigned to it, this group is not recognized in the species catalogue in this paper.

**Unplaced species.**

**Figures 237–244.**
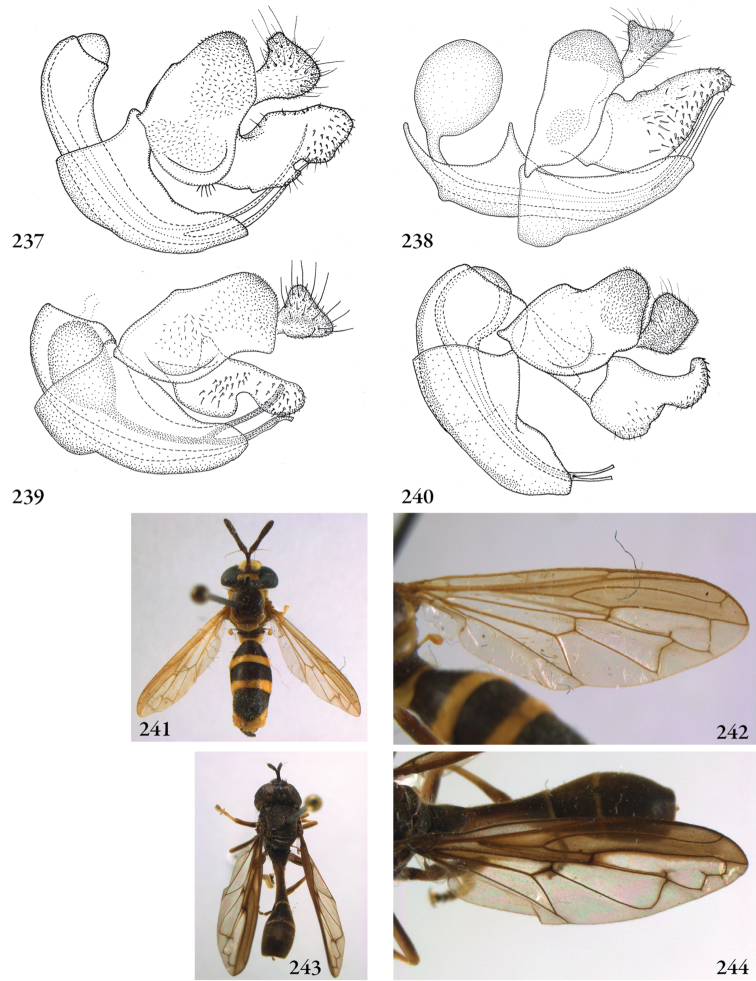
**237–240** *Microdon* (s.l.), male genitalia: **237** *Microdon carbonarius* (holotype) **238** *Microdon pictipennis* (Australia, coll. W. van Steenis) **239** *Microdon tsara* (holotype) **240** *Microdon waterhousei* (Australia, coll. M. Hauser) **241–242** *Mixogaster breviventris* male (USA, coll. RMNH): **241** habitus dorsal **242** wing **242–244** *Mixogaster thecla* male (Brazil, coll. J.T. Smit): **243** habitus dorsal **244** wing.

Several species of *Microdon* s.l. (see Appendix 2) do not fit into any of the groups described above. In the phylogenetic analyses of Reemer and Ståhls (in press), the following species belonging to this group were included: *Microdon amabilis* Ferguson, 1926, *Microdon carbonarius* Brunetti, 1923, *Microdon nigromarginalis* Curran & Bryan, 1926, *Microdon pictipennis* (Macquart, 1850), *Microdon rieki* Paramonov, 1957, *Microdon trimacula* Curran, 1928, *Microdon tsara* Keiser, 1971,*Microdon waterhousei* Ferguson, 1926. The results hardly offer solid clues as to their exact relationships with *Microdon* s.s. For examples of morphology of these species see Figs 230, 231, 237–240.

**Diversity and distribution.**

In total, 126 species (from all continents except Antarctica) are here classified under *Microdon*. Of these, 62 are placed in *Microdon* s.s., 46 in other subgenera, and 18 are not placed in subgenera (although some of them in species groups) but left in *Microdon* s.l.

### Mixogaster

Macquart

http://species-id.net/wiki/Mixogaster

Figs 241 [Fig F33] –248

Mixogaster Macquart, 1842: 14. Type species: *Mixogaster conopsoides* Macquart, 1872: 14, by original designation.
Myxogaster Kertész, 1910: 351. Misspelling.
Myxogaster Shiraki, 1930: 8. Misspelling.

#### Description.

Body length: 9–15 mm. Slender flies with constricted abdomen, wasp-like. Head wider than thorax. Face convex or almost straight in profile; about as wide as an eye or narrower. Lateral oral margins not produced. Vertex flat. Occiput narrow, except slightly widened dorsally. Eye bare. Eyes in male not or hardly converging at level of frons, with mutual distance 4 to 5 times as large as width of antennal fossa. Antennal fossa about as wide as high. Antenna longer or shorter than distance between antennal fossa and anterior oral margin; basoflagellomere shorter to longer than scape, oval; bare. Postpronotum pilose. Scutellum semicircular; without calcars. Anepisternum with weak sulcus; entirely bare or pilose anterodorsally, or pilose anterodorsally and along posterodorsal margin. Anepimeron entirely pilose or bare on ventral half. Katepimeron convex; bare. Wing vein R4+5 without posterior appendix. Vein M with small anterior appendix into cell r4+5. Vein M1 either straight or with anterior part directed outward, with one or two angles, whether or not with small inward appendix and /or small outward appendix. Postero-apical corner of cell r4+5 angular. Crossvein r-m located betwee basal 1/4 to 2/5 of cell dm. Abdomen constricted at base, with tergite 2 varying in length and width. Tergites 3 and 4 not fused. Male genitalia: phallus unfurcate, bent dorsad, with either lateral or dorsal combined with ventral lamellae, sometimes with apical spines; hypandrium with bulb-like base and apical part consisting of separate lobes, or hypandrium entirely consisting of two separate parts, which are not interconnected; epandrium without ventrolateral ridge; surstylus of varying shape.

#### Diagnosis.

Vein M with small anterior appendix into cell r4+5. Abdomen constricted. Metapleura connected, postmetacoxal bridge complete.

#### Discussion.

An important diagnostic character of *Mixogaster*, the anterior appendix of vein M, is also found in *Spheginobaccha* de Meijere, 1908 and certain specimens of *Aristosyrphus primus*. These taxa also share the character of the apical part of the hypandrium consisting of two separate lobes. See genus account of *Aristosyrphus* for discussion.

**Figures 245–250. F33:**
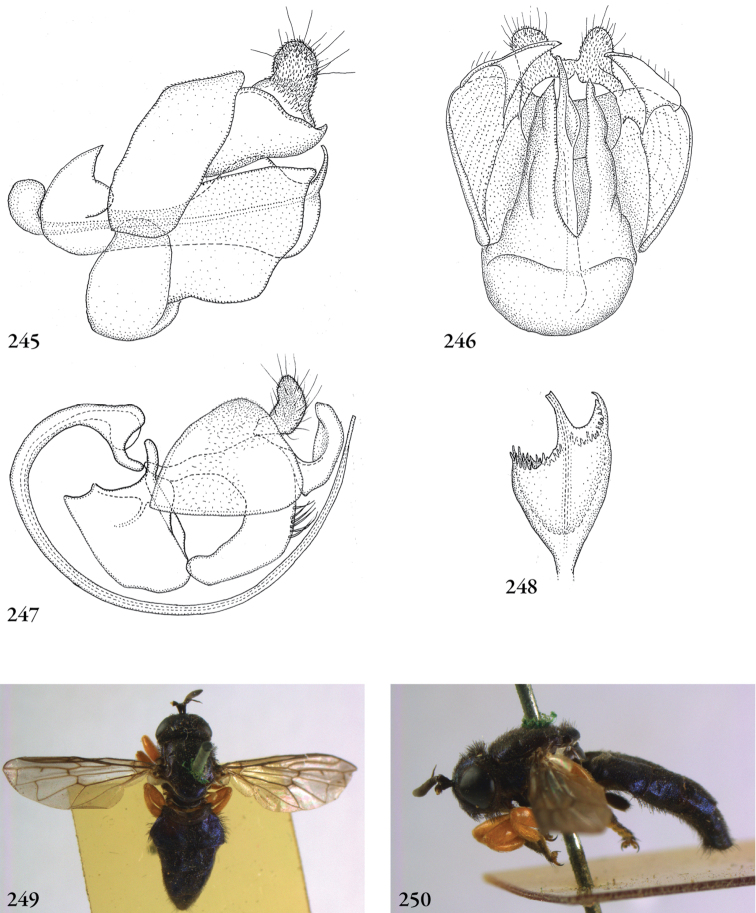
**245–246** *Mixogaster breviventris* male (USA, coll. RMNH), genitalia: **245** lateral **246** ventral. **Figure 247.** *Mixogaster thecla* male (Brazil, coll. J.T. Smit), genitalia lateral **248** *Mixogaster* spec. nov. male (Colombia, coll. RMNH), aedeagus ventral **249–250** *Oligeriops dimorphon* female (Australia, coll. BMNH): **249** habitus dorsal **250** habitus lateral.

The morphology of the male genitalia is remarkably diverse in this genus, much more so than in other groups of Microdontinae (except perhaps *Aristosyrphus* / *Eurypterosyrphus*). Some species have characters not known from any other Microdontinae. Some examples are illustrated in Figs 245–248. In *Mixogaster breviventris* Kahl, 1897, the phallus has wide dorsal and ventral lamellae (Fig. 245). This type of genitalia is found in all Nearctic species, which also have a straight vein M1 in common. In *Microdon thecla* Hull, 1954 (Fig. 247), the hypandrium consists of two separate lobes, which are not interconnected ventrally to envelope the phallus, as is the case in all other studied Microdontinae. Besides, the subepandrial sclerite is strongly developed in this species, and produced well beyond the epandrium in lateral view. In an undescribed species (Fig. 248), the phallus is asymmetric in ventral view, with wide lateral lamellae, which are apically densely occupied with irregular spines. This is the only known case of asymmetric genitalia among Microdontinae. The spinose phallus is also a unique character.

The keys to the species by Hull (1954) and Carrera and Lenko (1958) (Brazilian species only) work reasonably well, but the existence of several undescribed species makes it necessary to check original descriptions or, preferably, type material in order to verify identifications. Considering the large interspecific variation in the male genitalia, these characters should be further explored in future (re)descriptions of species.

#### Diversity and distribution.

Described species: 21. Mainly Neotropical, with three species in the Nearctic. At least one Nearctic and several Neotropical species are undescribed.

### *Myiacerapis* Hull (subgenus, see *Microdon*)

### Oligeriops

Hull

http://species-id.net/wiki/Oligeriops

Figs 249 –253

Oligeriops Hull, 1937a: 26. Type species: *Microdon chalybeus* Ferguson, 1926a: 176, by original designation.

#### Description.

Body length: 7–10 mm. Dark-coloured, stout-legged flies with oval abdomen and moderately long antennae. Head about as wide as thorax. Face convex; wider than an eye. Lateral oral margins produced. Vertex flat. Occiput wide over entire length, narrowest point halfway. Eye bare. Eye margins in male not converging at level of frons, with mutual distance around 4 times as large as width of antennal fossa. Antennal fossa about as wide as high. Antenna longer than distance between antennal fossa and anterior oral margin; basoflagellomere longer than scape; with dorsal margin curved dorsad, more or less sickle-shaped; bare. Postpronotum pilose. Scutellum semicircular; without calcars. Anepisternum weakly sulcate; pilose, with small bare part on ventral half. Anepimeron entirely pilose. Katepimeron convex; with wrinkled texture; bare. Wing: vein R4+5 with posterior appendix; vein M1 more or less straight, perpendicular to vein R4+5; postero-apical corner of cell r4+5 rectangular, with small appendix; crossvein r-m located between basal 1/6 of cell dm. Abdomen oval, about twice as long as wide. Tergites 3 and 4 fused. Sternite 1 pilose. Male genitalia: phallus not or little projecting beyond apex of hypandrium, slightly bent dorsad, shallowly furcate, with both processes about equally long; epandrium without ventrolateral ridge; surstylus unfurcate.

#### Diagnosis.

Vein R4+5 with posterior appendix. Postpronotum pilose. Abdomen oval. Anepisternum largely pilose, at most with small bare part on ventral half. Basoflagellomere sickle-shaped: dorsal margin curved upward.

#### Discussion.

Hull (1937a) described *Oligeriops* as a genus, with only *Microdon chalybeus* Ferguson, 1926 included, without indicating its diagnostic generic characters. Hull (1949) used the reduced size of the eyes (due to widened occiput and gena) and the sickle-shaped antenna as key characters. Thompson and Vockeroth (1989) list *Oligeriops* as synonym of *Microdon*. Cheng and Thompson (2008) express their doubts about ranking *Oligeriops* as a genus, while referring to the antennae of Australian *Microdon* species as illustrated in Ferguson (1926b). These illustrations show that other species originally described in *Microdon* also have a curved basoflagellomere, just like *Microdon chalybeus* Ferguson, 1926, but nevertheless these species were not included in *Oligeriops* by Hull (1937a, 1949). Cheng and Thompson (2008) state that ‘Whether these other species have reduced eyes remains to be seen!’. However, as Ferguson (1926a, b) already noticed, the four species he described are all ‘close’ and ‘very similar’. Examination of type specimens, additional material and original descriptions, has confirmed this, and has made clear that all five species presently included in *Oligeriops* have reduced eyes and sickle-shaped basoflagellomeres indeed. Based on these and other morphological similarities, there is no doubt that they are closely related.

*Oligeriops* does not fit into the concept of *Microdon* s.s. as defined in the present paper. In addition to the reduced size of the eye and the curved basoflagellomere, the following characters distinguish *Oligeriops* from *Microdon*: anepisternum almost entirely pilose, at most with small bare part ventrally; propleuron bare; postero-apical corner of cell r4+5 rectangular; phallus projecting little beyond apex of hypandrium, furcate near apex. Considering these characters in combination with the results of Reemer and Ståhls (in press), it is deemed not appropriate to include this taxon in *Microdon*.

#### Diversity and distribution.

Described species: 5. Australia (incl. Tasmania).

### Omegasyrphus

Giglio-Tos stat. n.

http://species-id.net/wiki/Omegasyrphus

Figs 254–256

Omegasyrphus Giglio-Tos, 1891: 4. Type species: *Microdon coarctatus* Loew, 1864: 74, by subsequent designation of Giglio-Tos (1892: 3).

#### Description.

Body length: 7–9 mm. Small, dark flies with relatively short antennae and characteristically shaped abdomen. Head slightly wider than thorax. Face convex; about as wide as or narrower than an eye. Lateral oral margins hardly produced. Vertex flat or slightly produced, densely pilose. Occiput ventrally narrow, dorsally widened. Eye bare. Eye margins in male slightly converging at level of frons, with mutual distance 2.5-3 times width of antennal fossa. Antennal fossa about as wide as high. Antenna shorter than distance between antennal fossa and anterior oral margin; basoflagellomere as long as or longer than scape, oval; bare. Postpronotum pilose. Scutellum semicircular; with calcars. Anepisternum sulcate; entirely pilose. Anepimeron entirely pilose. Katepimeron moderately convex; with wrinkled texture; bare. Wing: vein R4+5 with posterior appendix; vein M1 perpendicular to vein R4+5; postero-apical corner of cell r4+5 rectangular to weakly rounded, with small appendix; crossvein r-m located between basal 1/6 to 1/5 of cell dm. Abdomen 2.5-3 times as long as wide; with characteristic shape: widest point about halfway tergite 2, which has strongly arcuate lateral margins and pair of depressed areas dorsally; tergites 3-4 narrower and almost parallel-sided. Tergites 3 and 4 fused. Sternite 1 pilose. Male genitalia: phallus furcate near apex, with dorsal process long and whip-like, ventral process very short; epandrium with ventrolateral ridge.

#### Diagnosis.

Vein R4+5 with posterior appendix. Antenna shorter than distance between antennal fossa and anterior oral margin. Tergite 2 with strongly arcuate lateral margins, tergites 3-4 narrower and almost parallel-sided. Sternite 2 and 3 separated by membrane that is much less wide than sternite 2.

#### Discussion.

This group was treated as a subgenus of *Microdon* by Thompson (1981b). Based on the phylogenetic evidence of Reemer and Ståhls (in press) this ranking cannot be maintained. Instead, *Omegasyrphus* is treated as a distinct genus. Thompson (1981b), who gives a key to the North American species, points out that species level taxonomy is necessary for this genus. This is still true.

#### Diversity and distribution.

Described species: 5. North and Central America, from South Dakota in the U.S.A. southward to Guatemala. The south border of this range is marked by *Microdon brunnipennis* Hull, 1944, which was described as a variety of *Microdon baliopterus* Loew, 1872 by Hull (1944b). The assignment of this taxon to *Omegasyrphus* is based only on this description, as the type has not been examined.

### Paragodon

Thompson

http://species-id.net/wiki/Paragodon

Figs 257–261

Paragodon Thompson, 1969: 74. Type species: *Paragodon paragoides* Thompson, 1969: 81, by original designation.

#### Description.

Body length: 4–5 mm. Small flies with short antennae and oval abdomen. Head slightly wider than thorax. Face convex; narrower than an eye. Lateral oral margins not produced. Vertex flat. Occiput ventrally narrow, dorsally widened. Eye bare. Eye margins in male not converging at level of frons, with mutual distance about three times as large as width of antennal fossa. Antennal fossa about as wide as high. Antenna shorter than distance between antennal fossa and anterior oral margin; basoflagellomere longer than scape, oval, about 1.5 times as long as wide, bare. Postpronotum pilose. Scutellum semicircular; without calcars. Anepisternum convex; pilose anteriorly and posterodorsally, widely bare in between. Anepimeron bare or with a few thick, seta-like pile dorsally. Katepimeron convex; bare. Wing: vein R4+5 without posterior appendix; vein M1 straight, perpendicular to vein R4+5; postero-apical corner of cell r4+5 rectangular, with small appendix; crossvein r-m located very close to base of cell dm. Abdomen oval, about 1.5 times as long as wide. Tergites 3 and 4 fused. Sternite 1 bare. Male genitalia: phallus unfurcate, straight, projecting only little beyond apex of hypandrium; hypandrium with bulb-like base; epandrium without ventrolateral ridge; surstylus unfurcate.

#### Diagnosis.

Abdomen oval; yellow and black. Vein R4+5 without posterior appendix. Crossvein r-m almost at same level as base of cell dm. Antenna shorter than distance between antennal fossa and anterior oral margin. Postpronotum pilose.

#### Discussion.

When Thompson (1969) described this genus, he stated that it appeared to be the most primitive microdontine fly known. This was based on a number of presumed plesiomorphic characters: 1) unsclerotized ejaculatory apodeme and sac; 2) short antenna; 3) underdeveloped and bare metasternum; 4) lack of basal setal patches on hind femur; 5) lack of a spurious vein; 6) lack of appendix on vein R4+5; 7) presence of a double sustentacular apodeme; 8) unfurcate phallus. Now that a larger number of taxa of Microdontinae could be studied, all of these characters were also found in other taxa (Reemer and Ståhls in press), except for the unsclerotized ejaculatory apodeme. See also discussion under *Surimyia*.

#### Diversity and distribution.

Described species: 1. Central America (Mexico, Costa Rica and Panama).

### Paramicrodon

de Meijere

http://species-id.net/wiki/Paramicrodon

Figs 262–268

Paramicrodon de Meijere, 1913: 359. Type species: *Paramicrodon lorentzi* Meijere, 1913: 360, by monotypy.
Syrphinella Hervé-Bazin, 1926: 73. Type species: *Syrphinella miranda* Hervé-Bazin, 1926: 74, by monotypy.
Myxogasteroides Shiraki, 1930: 9. Type species: *Myxogaster nigripennis* Sack, 1922: 275, by original designation.
Nannomyrmecomyia Hull, 1945: 75. Type species: *Paramicrodon delicatulus* Hull, 1937: 24, by original designation. Described as subgenus of *Spheginobaccha*.

#### Description.

Body length: 4–11 mm. Small, slender flies with short antennae and more or less parallel-sided abdomen. Head slightly wider than thorax. Face convex; narrower than an eye. Lateral oral margins not produced. Vertex flat. Occiput ventrally narrow, dorsally strongly widened. Eye bare. Eye margins in male only slightly converging at level of frons, with mutual distance 1.5-2.5 times as large as width of antennal fossa. Antennal fossa about as wide as high. Antenna shorter than distance between antennal fossa and anterior oral margin; basoflagellomere longer than scape, oval, about 1.5 times as long as wide, bare. Postpronotum pilose. Scutellum semicircular; without calcars. Anepisternum convex; pilose anteriorly and posteriorly, widely bare in between. Anepimeron entirely pilose. Katepimeron convex; bare. Wing: vein R4+5 without posterior appendix; vein M1 straight, perpendicular to vein R4+5; postero-apical corner of cell r4+5 rectangular, with small appendix; crossvein r-m located within basal 1/10 of cell dm. Abdomen elongate: more or less parallel-sided, may be subtly constricted at tergite 3 (male), or slightly oval (female); 2.5–4 times as long as wide. Tergites 3 and 4 fused (but distinct suture visible). Sternite 1 bare or pilose. Sternites 3-4 strongly narrowed; narrower than sternite 2, with wide membraneous parts laterally. Male genitalia: phallus furcate near apex, slightly bent dorsad, projecting well beyond apex of hypandrium; hypandrium with apical part consisting of two separate lobes; epandrium without ventrolateral ridge; surstylus of varying shape.

#### Diagnosis.

Vein R4+5 without posterior appendix. Postpronotum pilose. Antenna shorter than distance between antennal fossa and anterior oral margin. Vein M1 straight, not parallel to wing margin, perpendicular to both vein R4+5 and M. Mesonotum with transverse suture incomplete. Sternites 3-4 strongly narrowed; narrower than sternite 2, with wide membraneous parts laterally.

#### Discussion.

The synonymy of *Syrphinella* Hervé-Bazin, 1926 with *Paramicrodon* was suspected by Hull (1937a) and stated explicitly by Hull (1949). This subjective synonymy is here confirmed, based on examination of the type specimen of the type species. *Myxogasteroides* Shiraki, 1930 was treated as a synonym of *Paramicrodon* by Hull (1949) and Cheng and Thompson (2008), a synonymy followed here based on the description of the type species. The synonymy of *Nannomyrmecomyia* Hull, 1945 and *Paramicrodon* was stated by Thompson (1969, 1981a) and is also confirmed here based on examination of the type specimens.

#### Diversity and distribution.

Described species: 8. The range of this genus is interestingly disjunct, with six species from the Oriental Region (Thailand to Moluccas), one from New Guinea and two from the Neotropical region. At least one additional species occurs in the Neotropical region (unpublished observations by the first author), but more species-level work is needed to sort this out.

### Paramixogaster

Brunetti

http://species-id.net/wiki/Paramixogaster

Figs 269 –284

Paramixogaster Brunetti, 1923: 319. Type species: *Paramixogaster vespiformis* Brunetti, 1923: 320, by original designation.
Paramixogasteroides Shiraki, 1930: 8. Type species: *Myxogaster variegata* Sack, 1922: 16, by original designation.
Tanaopicera Hul, 1945: 76. Type species: *Ceratophya variegatus* Walker, 1852: 220, by original designation.

#### Description.

Body length: 5–13 mm. Slender flies with constricted abdomen and long antennae, usually with black and yellow colour pattern, wasp mimics. Head wider than thorax. Face convex in profile; narrower than to wider than an eye. Lateral oral margins not produced. Vertex flat to strongly swollen. Occiput ventrally narrow, dorsally widened. Antennal fossa about as wide as high. Eye bare. Eye margins in male parallel, not converging at level of frons. Antenna longer than distance between antennal fossa and anterior oral margin. Basoflagellomere usually much longer than scape, except shorter in *Paramixogaster illucens* (Bezzi) and *Paramixogaster luxor* Curran (see Discussion); bare. Postpronotum bare. Mesoscutum with transverse suture usually incomplete, except complete in *Paramixogaster contractus*, *Paramixogaster conveniens* and *Paramixogaster omeanus* (see Discussion). Scutellum semicircular; without or with small calcars. Anepisternum convex or sulcate; entirely pilose or partly bare on ventral half. Anepimeron entirely pilose. Katepimeron convex; bare. Wing: vein R4+5 with or without posterior appendix; vein M1 perpendicular to vein R4+5 and vein M; postero-apical corner of cell r4+5 rectangular to somewhat acute, with small appendix; crossvein r-m located within basal 1/4 of cell dm. Abdomen elongate, at least 3 times as long as wide; constricted, with narrowest point at tergite 2 and widest point at tergite 3 or 4. Tergites 3 and 4 fused. Male genitalia: phallus furcate, with furcation point in distal 1/3; epandrium without ventrolateral ridge; surstylus weakly furcate, only in *Paramixogaster luxor* consisting of three distinct branches.

#### Diagnosis.

Postpronotum bare. Basoflagellomere at least three times as long as wide. Posterio-apical corner of wing cell r4+5 rectangular or somewhat acute. Abdomen usually constricted (i.e. with narrowest point at tergite 2 and widest point at tergite 3 or 4); if not, then the following three characters apply: 1) basoflagellomere 2-4 times as long as scape, 2) tergite 2 less than half as long as tergites 3 and 4 together, 3) face smooth medially (without vitta of transversely wrinkled texture).

#### Discussion.

Cheng and Thompson (2008) regarded *Paramixogasteroides* Shiraki, 1930 and *Tanaopicera* Hull, 1945 as subjective synonyms of *Paramixogaster*. Examination of the type species of *Tanaopicera*, *Ceratophya variegata* Walker, 1852, confirmed this opinion with regard to *Tanaopicera*. One of the characters Hull (1945) used to characterize *Tanaopicera* was ‘the high, greatly developed vertex’. However, the vertex in the holotype of *Ceratophya variegata* is neither high nor greatly developed. This species is very similar to other *Paramixogaster*-species in all important characters. The type species of *Paramixogasteroides*, *Myxogaster variegata* Sack, was not examined, but its description by Sack (1922) is clear enough to include this taxon in *Paramixogaster*.

Morphological variation among the species presently included in *Paramixogaster* is large. Although most species have a constricted abdomen in dorsal view, this is not the case in the African taxa *Paramixogaster acantholepidis* (Speiser, 1913) and *Paramixogaster crematogastri* (Speiser, 1913), and the Australian species *Paramixogaster praetermissus* (Ferguson, 1926). However, tergite 2 is dorsoventrally flattened in these species, so in lateral view their abdomen appears constricted. In all other important characters of external morphology and male genitalia these taxa belong in *Paramixogaster*, as corroborated by the results of the phylogenetic analysis based on morphology (Reemer and Ståhls in press).

*Paramixogaster illucens* (Bezzi, 1915) and *Paramixogaster luxor* (Curran, 1931) are the only species included in this genus in which the basoflagellomere is shorter than the scape. In *Paramixogaster luxor*, the shape of the surstylus also differs from the other species, as it consists of three separate branches (Fig. 282). Nevertheless, both species are included in *Paramixogaster* because agree with the diagnosis.

*Paramixogaster contractus* (Brunetti, 1923), *Paramixogaster conveniens* (Brunetti, 1923) and *Paramixogaster omeanus* (Paramonov, 1957) are aberrant from all other known species of *Paramixogaster* in their complete transverse suture. This character is also found in *Indascia*, which includes species which look superficially similar to these *Paramixogaster*-species. However, these species are here assigned to *Paramixogaster*, based on the phylogenetic analysis of their morphology (Reemer and Ståhls in press). In addition, they possess a diagnostic character for *Paramixogaster*: the bare postpronotum. The first two species, *Paramixogaster contractus* and *Paramixogaster conveniens*, differ from all other studied species of Microdontinae in the presence of pile on the metaepisternum. It will be interesting to re-evaluate their taxonomic affinities when additional material becomes available. At present, the species are only known from the holotypes, which both are females, so no characters of male genitalia or DNA could be analyzed.

As a consequence of transferring some species from other genera to *Paramixogaster*, replacement names had to be chosen for two species. Examination of the type of *Microdon vespiformis* de Meijere, 1908 made clear that this is a species of *Paramixogaster*. As *Mixogaster vespiformis* Brunetti, 1913 was later designated as the type species of *Paramixogaster*, these two names are now secondary homonyms. For the junior name, *vespiformis* Brunetti, the nomen novum *Paramixogaster brunettii* is proposed here. The other new name introduced here is *Paramixogaster sacki* for *Paramixogasteroides variegata* Sack, 1922, which is a junior secondary homonym of *Ceratophya variegata* Walker, 1852.

#### Diversity and distribution.

Described species: 26. Afrotropical (5 species), Oriental (12) and Australian region (9). Several additional species, from all three regions, await description.

### Parocyptamus

Shiraki

http://species-id.net/wiki/Parocyptamus

Figs 285–290

Parocyptamus Shiraki, 1930: 11. Type species: *Parocyptamus sonamii* Shiraki, 1930: 12, by original designation.
Stenomicrodon Hull, 1937a: 26. Type species: *Stenomicrodon purpureus* Hull, 1937: 26, by original designation. See Hull (1949) for synonymy.

#### Description.

Body length: 11–15 mm. Slender flies with elongate, tapering abdomen and long antennae, black with metallic hues, wings infuscated. Head about as wide as thorax. Face approximately straight in profile, except for slight bulge below antenna; narrower than eye. Lateral oral margins strongly produced. Vertex flat. Occiput ventrally narrow, dorsally slightly widened. Antennal fossa about as wide as high. Eye bare. Eye margins in male parallel, not converging at level of frons, mutual distance about three times width of antennal fossa. Antennal fossa about as wide as high. Antenna longer than distance between antennal fossa and anterior oral margin. Basoflagellomere shorter than scape; oval; bare. Postpronotum pilose. Scutellum semicircular; without calcars. Anepisternum deeply sulcate; almost entirely pilose, except bare on small part ventrally. Anepimeron entirely pilose. Katepimeron convex; bare. Wing: vein R4+5 with posterior appendix; vein M1 perpendicular to vein R4+5; postero-apical corner of cell r4+5 widely rounded; crossvein r-m located around basal 1/6 of cell dm. Abdomen elongate, more than 3 times as long as wide; in male gradually tapering from anterior half of tergite 2 to apex; in female slightly constricted between tergites 3 and 4. Tergites 3 and 4 fused. Male genitalia: phallus furcate basally, with dorsal process much longer than ventral one, projecting far beyond apex of hypandrium; epandrium with ventrolateral ridge; surstylus weakly furcate, divided into two short lobes.

**Diagnosis.** Vein R4+5 with posterior appendix. Postpronotum pilose. Anepisternum almost entirely pilose, except bare on small ventral part. Basoflagellomere shorter than scape. Abdomen at least three times as long as wide. Tergite 2 with pair of depressed areas (Fig. 287).

#### Discussion.

When Shiraki (1930) described *Parocyptamus*, this genus was diagnosed in a key by the following two characters: abdomen narrow and elongate, frons with antennal prominence (‘Fühlervorsprung’). The latter character is of limited use, as the frons is more or less extended above the antennae in many other taxa of Microdontinae. Hull (1937a) did not state which characters he considered diagnostic in his description of *Stenomicrodon*. Judging from his remarks in Hull (1949), the shape of the abdomen and the presence of a patch of short, spinose setae at the base of the front and mid femora were considered important characters. Although the anterobasal patches of setae are well-developed, such patches are also found in several other taxa of Microdontinae. Perhaps the spines are somewhat stronger developed than in most taxa, but it is hard to describe this as a discrete character state. Therefore, this character is not used in the present key and Diagnosis.

The abdomen is constricted (slightly) only in the female, not in the male, as might be erroneously concluded from the key of Cheng and Thompson (2008).

The synonymy of *Stenomicrodon* with *Parocyptamus* was already established by Hull (1949). Examination of the involved type specimens by the first author has confirmed this (subjective) synonymy. The type species of both genus group names are here also considered as synonyms (*Parocyptamus sonamii* Shiraki, 1930 = *Stenomicrodon purpureus* Hull, 1937 **syn. n.**). *Microdon stenogaster* Curran, 1931 also belongs to this genus, as it is almost identical to the type species in colouration, external morphology and male genitalia. Closer examination of available specimens, also from Sumatra and Thailand, is necessary to resolve species level taxonomy.

Shiraki (1930) based his description of *Parocyptamus sonamii* on three males. Two of these syntypes are kept in the NIAS collection. The third male (from Sokotsu) is apparently lost. Label information is as follows. Syntype 1: label 1: “Formosa, Shinchiku, -18. VII 1-30. J. Sonan, K. Miyake”; label 2: “Parocyptamus sonamii”; label 3 (round, red-bordered): “Type”. Syntype 2: label 1: “CIHpOn, 17.VII.1922, M. Yoshino”; label 2 (round, red-bordered): “Type”. The date on the label of syntype 1 is a bit cryptic (“-18. VII 1-30”). It is unlikely to assume the specimen has been collected in July 1930, because Shiraki’s work was published on the 30th of January 1930. It seems more plausible that the date was 1-30 July 1918. Shiraki (1930) only mentions the month (VII).

#### Diversity and distribution.

Described species: 2. Oriental: known from Taiwan, Thailand, Sumatra and Borneo.

### Peradon

Reemer gen. n.

urn:lsid:zoobank.org:act:4FB70E62-18D6-4B74-B96D-296B925BACBB

http://species-id.net/wiki/Peradon

Figs 291 –298

#### Type species:

*Mulio bidens* Fabricius, 1805. Type locality: “America Meridionalo”.

#### Description.

Body length: 6–18 mm. Slender to moderately broadly built flies with oval or basally constricted abdomen and long antennae. Head wider than thorax. Face straight to slightly convex or slightly concave in dorsal half; narrower to wider than an eye; medially with vitta of transversely wrinkled texture (except in some smaller species of the *flavofascium*-group); gena distinctly ventrally produced. Lateral oral margins produced. Vertex flat. Occiput ventrally narrow, dorsally widened. Eye bare. Eye margins in male converging at level of frons, with mutual distance 1.5-4 times as large as width of antennal fossa. Antennal fossa about as wide as high. Antenna longer than distance between antennal fossa and anterior oral margin; basoflagellomere shorter to longer than scape; bare. Postpronotum pilose or bare. Scutellum semicircular; with calcars. Anepisternum sulcate; pilose anterodorsally and posteriorly, widely bare in between. Anepimeron entirely pilose. Katepimeron flat; with wrinkled texture; bare. Wing: vein R4+5 with posterior appendix; vein M1 more or less straight, perpendicular to vein R4+5; postero-apical corner of cell r4+5 widely rounded, without appendix; crossvein r-m located between basal 1/6 and 1/3 of cell dm. Abdomen oval or basally constricted, 2-4 times as long as wide. Tergites 3 and 4 fused. Sternite 1 bare. Male genitalia: phallus not or little projecting beyond apex of hypandrium, slightly bent dorsad, shallowly furcate, with both processes about equally long and with their apexes wide at the furcation point but pointed apically; epandrium without ventrolateral ridge; surstylus unfurcate.

#### Diagnosis.

Vein R4+5 with posterior appendix. Postero-apical corner of cell r4+5 widely rounded. Katepimeron flat, with wrinkled texture, bare. Face in profile slightly convex, straight or slightly concave, but never bulged in ventral half. Vertex flat.

Three species groups are recognized here. These groups may not be monophyletic, but they may be useful for purposes of species identification. They are diagnosed as follows:

*bidens*-group: Abdomen oval or parallel-sided. Tergites without golden pile. Basoflagellomere less than twice as long as scape.

*flavofascium*-group: Abdomen oval. Tergite 4 often with golden or silver pile. If not, then basoflagellomere more than twice as long as scape.

*trivittatus*-group: Abdomen constricted basally.

#### Discussion.

The species here assigned to this genus (see Appendix 2) used to be placed in *Microdon* (e.g. Thompson et al. 1976), but the results of the phylogenetic analyses by Reemer and Ståhls (in press) indicate that they do not belong there. Based on external characters this group is difficult to diagnose, but usually the species can be distinguished at a glance from those of *Microdon* because of their more or less elongate (sometimes constricted) abdomen. In addition, morphology of the phallus is very constant (differences with *Microdon* in parentheses): projecting not or little beyond apex of hypandrium (far beyond apex of hypandrium), slightly bent dorsad (strongly bent), shallowly furcate (deeply furcate), with both processes about equally long and with their apexes wide at the furcation point but pointed apically.

Only one species here included in *Peradon* was previously not classified in *Microdon*: *Ubristes chrysopygus* Giglio-Tos, 1892.

#### Diversity and distribution.

Described species: 24. Neotropical. Several undescribed species are known to the first author.

#### Etymology.

The generic name is a combination of the Greek words *peras* (west) and *odon*, with the latter used as a suffix derived from *Microdon*. The prefix *pera-* is used to emphasize that this genus is restricted in its distribution to the western hemisphere.

### Piruwa

Reemer gen. n.

urn:lsid:zoobank.org:act:70D34EA7-E2E7-4868-AE3E-AC5E74A3DDFD

http://species-id.net/wiki/Piruwa

Figs 299–306

#### Type species:

*Piruwa phaecada* spec. n. Type locality: Peru, Sachavacayoc.

#### Description.

Body length: 4 mm. Small, slender flies with short antennae and constricted abdomen. Head slightly wider than thorax. Face convex; narrower than an eye. Lateral oral margins not produced. Vertex flat. Occiput narrow over entire length. Eye bare. Eye margins in male not converging at level of frons, with mutual distance 3 times as large as width of antennal fossa. Antennal fossa about as wide as high. Antenna shorter than distance between antennal fossa and anterior oral margin; basoflagellomere longer than scape, oval, about twice as long as wide, bare. Postpronotum bare. Scutellum semicircular; without calcars; with long bristly pile along margin, clearly longer and thicker than pile on rest of scutellum. Anepisternum convex; pilose anterodorsally and along posterodorsal margin. Anepimeron pilose along dorsal margin. Katepimeron convex; bare. Wing: vein R4+5 without posterior appendix; vein M1 straight, perpendicular to vein R4+5; postero-apical corner of cell r4+5 rectangular, with small appendix; crossvein r-m located within basal 1/10 of cell dm. Abdomen constricted, narrowest at transition between tergites 1 and 2, widest at tergite 4; about 2.5 times as long as wide. Tergites 3 and 4 fused, no suture visible. Sternite 1 bare. Male genitalia: phallus furcate near apex, slightly bent dorsad, projecting only a little beyond apex of hypandrium; hypandrium with bulb-like base, with apical part entire, not consisting of two separate lobes; epandrium without ventrolateral ridge; surstylus consisting of two lobes, with basal lobe angular, apical lobe rounded.

#### Diagnosis.

Vein R4+5 without posterior appendix. Antenna shorter than distance between antennal fossa and anterior oral margin. Postpronotum bare. Abdomen constricted.

#### Discussion.

Although there is a superficial similarity in habitus to *Paramicrodon* (small, slender, short antennae, vein R4+5 without posterior appendix), *Piruwa* differs from that genus in the following important characters: occiput narrow over entire length; postpronotum bare; scutellum with long bristly pile along margin; anepimeron pilose only along dorsal margin; sternites 3-4 about as wide as sternite 2; hypandrium with apical part not consisting of two separate lobes. Considering these differences, a close relationship between these taxa seems unlikely. Because of these differences and the uncertainy of taxonomic affinities, this distinct taxon is given generic rank.

#### Diversity and distribution.

Described species: 1. Neotropical. Only known from Peru.

#### Etymology.

The name *Piruwa* is derived from Piruw, the word for Peru in Quechuan, a native Andean-Ecuadorian language. It is to be treated as feminine.

### Pseudomicrodon

Hull

http://species-id.net/wiki/Pseudomicrodon

Figs 307 [Fig F41] –320

Pseudomicrodon Hull, 1937a: 24. Type species: *Microdon beebei* Curran, 1936: 4, by original designation.

#### Description.

Body length: 7–9 mm. Slender flies with long antennae and petiolate abdomen. Head a little wider than thorax. Face more convex or straight in profile; narrower than to as wide as an eye. Lateral oral margins weakly produced. Vertex convex and shining; sparsely pilose, sometimes bare on anterior half. Occiput ventrally narrow, dorsally strongly widened. Eye bare or with very short and sparse pile. Eye margins in male converging at level of frons, with mutual distance 1-2 times width of antennal fossa. Antennal fossa about as wide as high to 1.5 times as wide as high. Antenna longer than distance between antennal fossa and anterior oral margin; basoflagellomere shorter to longer than scape, oval; bare. Postpronotum pilose. Scutellum semicircular; with or without calcars. Anepisternum sulcate; entirely pilose or widely bare medially. Anepimeron entirely pilose. Katepimeron flat to convex; usually with wrinkled texture; bare. Wing: vein R4+5 with posterior appendix; vein M1 perpendicular to vein R4+5; postero-apical corner of cell r4+5 widely rounded to rectangular, with or without small appendix; crossvein r-m located between basal 1/6 to 1/3 of cell dm. Abdomen elongate, more than three times as long as wide, constricted, with narrowest point between halfway tergite 2 and transition between tergites 2 and 3. Tergites 3 and 4 fused. Sternite 1 pilose or bare. Male genitalia: phallus furcate near apex, with dorsal process long and whip-like, ventral process very short; epandrium with ventrolateral ridge.

#### Diagnosis.

Vein R4+5 with posterior appendix. Vertex convex and shining, sparsely pilose to bare. Abdomen petiolate, except parallel-sided in *Pseudomicrodon biluminiferus* Hull, but tergite 2 distinctly dorsoventrally flattened in that species.

#### Discussion.

Among Microdontinae with a petiolate abdomen, *Pseudomicrodon* species are recognized by their convex and shining vertex. *Microdon biluminiferus* Hull, 1944 is the only included species without a petiolate abdomen. Instead, the abdomen is parallel-sided, but in lateral view appears constricted because of the dorsoventrally flattened segment 2. This species is assigned to *Pseudomicrodon* based on the convex vertex and the morphology of the male genitalia (Figs 319, 320), combined with the results of the phylogenetic analysis of morphological characters (Reemer and Ståhls in press).

At present, the basis for distinguishing *Ceriomicrodon*, *Pseudomicrodon* and *Rhopalosyrphus* is narrow. The groups are certainly related, but as presently defined it is doubtful whether they are monophyletic, considering the variation in several morphological characters.

Keiser (1971) described *Pseudomicrodon elisabethae* from Madagascar. This species is here included in *Paramixogaster*. Cheng and Thompson (2008) mention the similarity of the South African taxon *Microdon illucens* Bezzi, 1915 to *Pseudomicrodon*, which is here also included in *Paramixogaster*.

#### Diversity and distribution.

Described species: 15. Neotropical.

### Ptilobactrum

Bezzi

http://species-id.net/wiki/Ptilobactrum

Figs 321 –326

Ptilobactrum Bezzi, 1915: 136. Type species: *Ptilobactrum neavei* Bezzi, 1915: 137, by original designation.

#### Description.

Body length: 13 mm. Broadly built flies with very wide head, long antennae and orange markings on abdomen. Head wider than thorax. Face much wider than eye; dorsally with oblique groove from lunule to eye margin; convex in profile. Lateral oral margins weakly produced. Vertex not swollen, more or less flat, but much wider than eye. Occiput narrow ventrally, moderately widened dorsally. Eye bare. Eye margins in male not converging at level of frons, with mutual distance about seven times width of antennal fossa. Antennal fossa somewhat higher than wide. Antenna longer than height of head. Basoflagellomere five times as long as scape; with long pilosity. Postpronotum pilose. Mesoscutum with transverse suture incomplete. Scutellum without calcars. Anepisternum with deep sulcus; entirely pilose. Anepimeron entirely pilose. Katepimeron convex; smooth; bare. Wing: vein R4+5 with posterior appendix; vein M1 straight, somewhat recurrent; postero-apical corner of cell r4+5 angular, with small appendix; crossvein r-m located around basal 1/3 of cell dm. Abdomen oval, widest at posterior margin of tergite 2. Tergites 3 and 4 fused. Sternite 1 bare. Sternite 4 in male visible from below. Male genitalia: phallus bent dorsad, except extreme apex bent ventrad; phallus furcate near apex; epandrium without ventrolateral ridge; surstylus broad, unfurcate, with short posterior lobe. Female unknown.

#### Diagnosis.

Vein R4+5 with posterior appendix. Basoflagellomere with long pile. Abdomen oval. Tergites 3 and 4 fused.

#### Discussion.

Bezzi (1915) distinguished *Ptilobactrum* from *Microdon* species by the “breadth of the head, the face being furrowed, and by the unusual shape of the antennae.” Indeed, the grooves on the face, running fom the lunula obliquely downward to the eye margins, are quite unusual among Microdontinae. They are reminescent of the ptilinal sutures of Diptera Schizophora. Similar grooves are found in certain species of *Furcantenna*,*Schizoceratomyia*, *Paramixogaster* and *Thompsodon*, but usually less distinct. The antennae are unusual in their long pilosity, a character shared with *Ceratrichomyia*, *Furcantenna*, *Kryptopyga* and *Schizoceratomyia*.

See *Ceratrichomyia* for a discussion on synonymy of that genus with *Ptilobactrum*, as proposed by Cheng and Thompson (2008).

#### Diversity and distribution.

Described species: 1. Afrotropical, only known from Kenya.

### Rhoga

Walker

http://species-id.net/wiki/Rhoga

Figs 327–331

Rhoga Walker, 1857: 157. Type species: *Rhoga lutescens* Walker, 1857: 157, by monotypy.
Papiliomyia Hull, 1937a: 27. Type species: *Papiliomyia sepulchrasilva* Hull, 1937: 28, by original designation. For synonymy see Hull (1949).

#### Description.

Body length: 5–10 mm. Stingless bee mimicking flies with short to moderately long antennae and oval, kite-shaped or more or less parallel-sided abdomen. Head slightly wider than thorax. Face convex; narrower than an eye. Lateral oral margins not produced. Vertex narrow, convexly produced and shining in most species, flat in some. Occiput wide and parallel-sided over entire length. Eye with short, sparse pile. Eye margins in male not converging at level of frons, with mutual distance 2 to 3 times as large as width of antennal fossa. Antennal fossa about as wide as high. Antenna as long as or shorter than distance between antennal fossa and anterior oral margin; basoflagellomere shorter to longer than scape, oval; bare. Postpronotum pilose. Scutellum semicircular, in some species weakly sulcate apicomedially; without calcars. Anepisternum without sulcus; pilose anterodorsally and posteriorly, widely bare in between. Anepimeron entirely pilose. Katepimeron convex; bare. Metapleurae either separated or forming postmetacoxal bridge. Wing: vein R4+5 without posterior appendix; vein M1 perpendicular to vein R4+5; postero-apical corner of cell r4+5 rectangular, with small appendix; crossvein r-m located within 1/4 of cell dm, usually within basal 1/10. Abdomen oval or kite-shaped, 1.5 to 2.5 times as long as wide. Tergites 3 and 4 fused. Sternite 1 pilose or bare. Male genitalia: phallus furcate near apex, with dorsal and ventral process equally long; epandrium without ventrolateral ridge.

#### Diagnosis.

Vein R4+5 without posterior appendix. Occiput widened and parallel-sided over entire length.

#### Discussion.

In some species (e.g. *Rhoga mellea* (Curran, 1940), *Rhoga maculata* (Shannon, 1927)) the metapleura are separated and do not form a postmetacoxal bridge. So far in Microdontinae, this character state was known only in the genus *Spheginobaccha* (Cheng and Thompson 2008).

The type specimen of the type species, *Rhoga lutescens* Walker, 1857, is not present in the BMNH-collection (pers. comm. N. Wyatt), where it is supposed to be according to Thompson et al. (1976) and Thompson (2010). Apparently it is lost.

#### Diversity and distribution.

Described species: 5. Central and South America. Several undescribed species are known to the first author.

### Rhopalosyrphus

Giglio-Tos

http://species-id.net/wiki/Rhopalosyrphus

**Figures 332–341.**
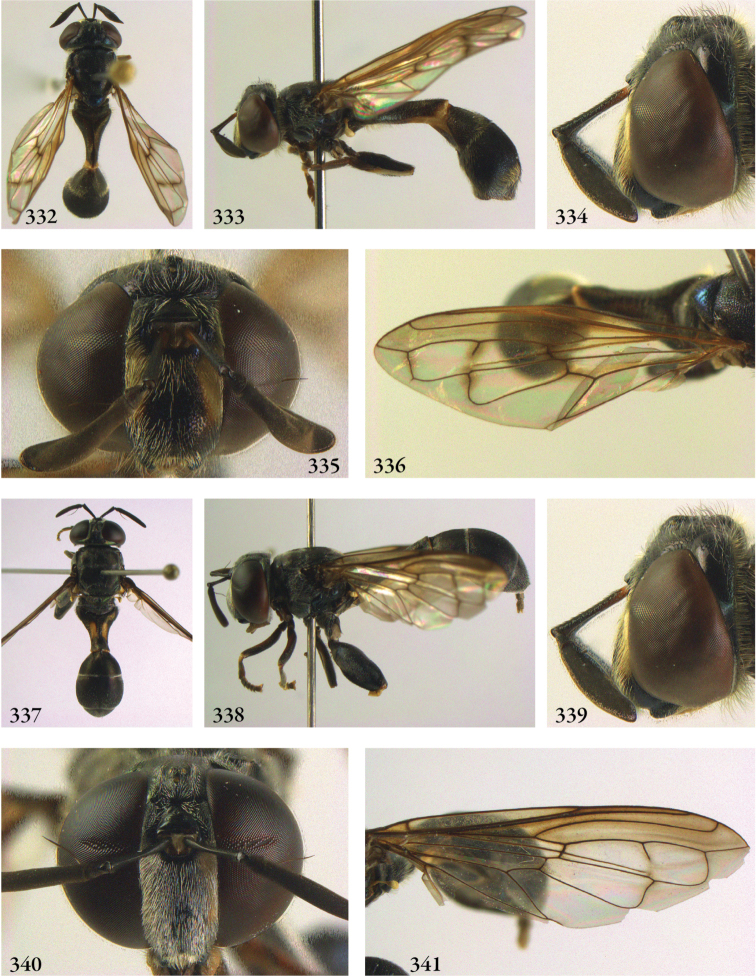
**332–336** *Rhopalosyrphus ecuadoriensis* male (holotype): **332** habitus dorsal **333** habitus lateral **334** head lateral **335** head frontal **336** wing **337–341** *Rhopalosyrphus robustus* female (holotype) **337** habitus dorsal **338** habitus lateral **339** head lateral **340** head frontal **341** wing.

Figs 332 [Fig F45] –355

Rhopalosyrphus Giglio-Tos, 1891: 189. Type species: *Holmbergia guentherii* Lynch Arribalzaga, 1891, by subsequent designation of Giglio-Tos (1892: 2).
Holmbergia Lynch Arribalzaga, 1891: 196. Type species: *Holmbergia guentherii*, 1891: 195, by monotypy. See Weems et al. (2003) and Cheng and Thompson (2008) for synonymy.

#### Description.

Body length: 9–15 mm. Slender flies with long antennae and petiolate abdomen. Head a little wider than thorax. Face more or less convexly produced on ventral half; narrower than an eye. Lateral oral margins produced. Vertex flat, entirely pilose. Occiput ventrally narrow, dorsally strongly widened. Eye bare. Eye margins in male converging at level of frons, with mutual distance 1-2 times width of antennal fossa. Antennal fossa about 1.5 times as wide as high. Antenna longer than distance between antennal fossa and anterior oral margin; basoflagellomere longer than scape, oval; bare. Postpronotum pilose. Scutellum semicircular; with or without calcars, if present, then small and with mutual distance small. Anepisternum convex or with weak sulcus; entirely pilose. Anepimeron entirely pilose. Katepimeron flat to weakly convex; with wrinkled texture; bare, partly pilose or entirely pilose. Wing: vein R4+5 with posterior appendix; vein M1 perpendicular to vein R4+5; postero-apical corner of cell r4+5 widely rounded to rectangular, with or without small appendix; crossvein r-m located between basal 1/8 to 1/4 of cell dm. Abdomen elongate, more than three times as long as wide, constricted, with narrowest point between halfway tergite 2 and transition between tergites 2 and 3. Tergites 3 and 4 fused. Sternite 1 pilose or bare. Male genitalia: phallus furcate near apex, with dorsal process long and whip-like, ventral process very short; epandrium with ventrolateral ridge.

#### Diagnosis.

Vein R4+5 with posterior appendix. Abdomen petiolate. Vertex flat, entirely pilose. Postpronotum pilose. Mesonotal transverse suture incomplete. Tergites 3 and 4 fused. Anterior margin of tergite 2 at least twice as wide as posterior margin. *Rhopalosyrphus* s.s.: katepimeron pilose. *Rhopalosyrphus* s.l.: katepimeron bare.

#### Discussion.

Previous authors have defined this genus more strictly than is done in the present paper. Weems et al. (2003) and Cheng and Thompson (2008) only included species with a pilose katepimeron. A number of additional species are known from the Neotropical region which are similar to *Rhopalosyrphus* auct. in most characters, but which have a bare or almost bare katepimeron. In *Rhopalosyrphus robustus* sp. n. the katepimeron is only narrowly pilose along the anterior margin. In all other characters, this species has the diagnostic characters of *Rhopalosyrphus* as described by Weems et al. (2003): abdomen petiolate, antenna longer than face, scape and basoflagellomere elongate, face produced ventrally (variable), occiput strongly widened dorsally, metasternum developed, hind tibia flared apically. The male genitalia of *Rhopalosyrphus robustus* are very similar to those of *Rhopalosyrphus* auct., with an apically furcate phallus, of which the dorsal process is very long and whip-like (Figs 352-355).

*Microdon abnormis* Curran, 1925 is also similar to *Rhopalosyrphus* in the characters mentioned above, but has a bare katepimeron. In the analysis of morphological characters by Reemer and Ståhls (in press), a closely related species (*Rhopalosyrphus abnormoides* sp. n.) is placed within *Rhopalosyrphus*.

Based on the results of the phylogenetic analyses and the (subjective) evaluation of external and genitalic characters, *Rhopalosyrphus* is here extended to include also the species with a bare or almost bare katepimeron, which includes species previously grouped in the *abnormis* group (see account of *Pseudomicrodon* in Cheng and Thompson 2008), as well as *Microdon cerioides* Hull, 1943. Species with a pilose katepimeron are included in *Rhopalosyrphus* s.s., while the other species are treated as *Rhopalosyrphus* s.l.

The inclusion of *Rhopalosyrphus oreokawensis* sp. n. in this genus is to be regarded as preliminary. Unlike the other species included in *Rhopalosyrphus*, this species has very short antennae, an oblique vein M1 and a more slender tergite 2. Analysis of its morphological characters (Reemer and Ståhls in press) resolves its phylogenetic position near *Rhopalosyrphus*. Possibly, it would be better to erect a new genus for this species. This is nevertheless not done here, in awaitance of a better understanding of the relationships of the taxa included in the ‘*Rhopalosyrphus-*clade’.

#### Diversity and distribution.

Described species: 9. Mainly Neotropical, with two species in southern parts of the U.S.A. (Arizona, Texas, Florida).

### Schizoceratomyia

Carrera, Lopes & Lane

http://species-id.net/wiki/Schizoceratomyia

Figs 356–363

Schizoceratomyia Carrera, Lopes & Lane, 1947a: 245. Type species: *Schizoceratomyia barretoi* Carrera, Lopes & Lane 1947a: 245, by original designation.
Johnsoniodon Curran, 1947: 1. Type species: *Johnsoniodon malleri* Curran, 1947: 1, by original designation. See Papavero (1962) for synonymy.

#### Description.

Body length: 4–9 mm. Broadly built flies with long antennae (bifrucate in male) and oval abdomen. Head wider than thorax. Face slightly convex or medially concave; wider than an eye. Mouth parts weakly developed, small; oral opening small and round, with lateral margins not produced. Vertex more or less flat, not strongly produced or convex. Frontal ocellus normal, split in two medially or absent. Occiput ventrally narrow, dorsally weakly widened. Eye bare. Eyes in male not or only slightly converging at level of frons, with mutual distance 3–4 times the width of antennal fossa. Antennal fossa about as wide as high. Antenna longer than distance between antennal fossa and anterior oral margin. Basoflagellomere longer than scape, bifurcate in male, in some species also in female; both branches long pilose, especially on inner side; in one (undescribed) species occupied with more than 20 long, narrow tubercles. Arista in male well-developed (longer than pedicel) or reduced to a small stump (shorter than pedicel); in female well-developed, sometimes almost as long as basoflagellomere and thickened. Postpronotum pilose or bare. Scutellum semicircular; without calcars. Anepisternum convex, sometimes with weak sulcus in dorsal 1/4; pilose on dorsal 2/3 to 3/4. Anepimeron pilose on dorsal 3/4 to 1/4, or only along posterior margin. Katepimeron convex; bare; smooth. Wing: vein R4+5 without posterior appendix; vein M1 straight and perpendicular to vein R4+5, or with weak outward angle in anterior 1/2; postero-apical corner of cell r4+5 rectangular to widely rounded, with or without small appendix; crossvein r-m located between basal 1/8 and 1/4 of cell dm. Abdomen dorsoventrally flattened; more oval, with largest width at tergite 3; 1.5-2 times as long as wide. Tergites 3 and 4 fused. Male genitalia: phallus furcate near apex, straight or apically bent ventrad, projecting not or hardly beyond apex of hypandrium; hypandrium with bulb-like base; epandrium without ventrolateral ridge; surstylus unfurcate, elongate or wide.

#### Diagnosis.

Vein R4+5 without posterior appendix. Abdomen oval. Antenna longer than distance between antennal fossa and anterior oral margin. Antenna inserted below dorsal eye margin. Vertex more or less flat. Katepisternum bare. Metasternum bare.

#### Discussion.

Hull (1949) and Papavero (1962) treated *Schizoceratomyia* as a synonym of *Masarygus*. See *Masarygus* for discussion on this synonymy, which is not followed here. These authors, as well as Cheng and Thompson (2008) also considered *Johnsoniodon* as a synonym of *Schizoceratomyia*, as is also done in the present paper. Although in the two species originally included in *Schizoceratomyia* (*Schizoceratomyia barretoi* Carrera, Lopes & Lane, 1947 and *Schizoceratomyia flavipes* Carrera, Lopes & Lane, 1947) the basoflagellomere is bifurcate in the male only, whereas in *Johnsoniodon* this character is found in the female, these taxa are otherwise very similar.

Apparently, Curran (1947) was unaware of the description of *Schizoceratomyia* by Carrera et al. (1947a, b) when his description of *Johnsoniodon malleri* Curran, 1947 was published, as this happened almost simultaneously. According to Van Doesburg (1966), the name *Schizoceratomyia* was published on the 3rd of July 1947, and *Johnsoniodon* on 14th of July 1947. Cheng and Thompson (2008) stated that *Schizoceratomyia* was published on the 12th of July 1947. Regardless of whether the date is 3^rd^ or 12^th^ of July *Schizoceratomyia* has priority over *Johnsoniodon*.

Besides *Schizoceratomyia malleri* (Curran), a furcate basoflagellomere in the female is also found in *Masarygus carrerai* Papavero, 1962. This species is also included in *Schizoceratomyia*.

Remarkably, in some specimens of *Schizoceratomyia*, the frontal ocellus is split in two, strongly reduced or even absent, whereas the posterior ocelli are well-developed. Following present species definitions, different states for this character seem to occur within the same species. However, more taxonomic work at species-level is necessary to establish whether this character state variation is intra- or inter-specific. In most Diptera and other flying insects, all three ocelli are well-developed. Reduced or absent ocelli occur in certain terrestrial insects, like certain ants and cockroaches. Among Diptera, they are partly or entirely absent only in a few groups, apparently mainly in certain nematocerous families and some brachypterous or apterous taxa (Cumming and Wood 2009). It will be interesting to try to correlate the degree of development of the frontal ocellus to behaviour and life-history of *Schizoceratomyia*-species; aspects which are currently unknown, unfortunately.

#### Diversity and distribution.

Described species: 4. Neoptropical. A few undescribed species are known to the first author.

### Serichlamys

Curran stat. n.

http://species-id.net/wiki/Serichlamys

Figs 364 –376

Serichlamys Curran, 1925a: 50. Type species: *Aphritis rufipes* Macquart, 1842: 71, by monotypy.

#### Description.

Body length: 7–13 mm. Small to medium-sized flies, black, brownish or metallic green, with moderately short to long antennae and oval abdomen. Head about as wide as thorax or slightly wider. Face convex; about as wide as an eye or narrower. Lateral oral margins not produced. Vertex flat. Occiput ventrally narrow, dorsally widened. Eye bare or pilose. Eye margins in male converging at level of frons, with mutual distance two to four times as large as width of antennal fossa. Antennal fossa about as wide as high. Antenna shorter to longer than distance between antennal fossa and anterior oral margin; basoflagellomere shorter to longer than scape, oval or slightly sickle-shaped with swollen base, with rounded apex; bare. Postpronotum pilose. Scutellum semicircular; with narrow, elongated calcars, often quite parallel and with small mutual distance, sometimes dorsoventrally flattened. Anepisternum weakly sulcate; pilose anteriorly and posteriorly, widely bare ventrally and medially. Anepimeron entirely pilose. Katepimeron convex; smooth or with wrinkled texture; bare. Wing: vein R4+5 with posterior appendix; vein M1 perpendicular to vein R4+5; postero-apical corner of cell r4+5 rectangular or weakly rounded, always with small appendix; crossvein r-m located between basal 1/5 to 1/3 of cell dm. Abdomen oval, about 1.5 to 2 times as long as wide. Tergites 3 and 4 fused. Sternite 1 pilose or bare. Male genitalia: phallus furcate, with furcation point near apex; hypandrium with basal part bulb-like; epandrium without ventrolateral ridge.

#### Diagnosis.

Vein R4+5 with posterior appendix. Abdomen oval. Vertex flat. Occiput dorsally (slightly) widened. Postpronotum pilose. Scutellum with calcars. Postero-apical corner of cell r4+5 rectangular, with small appendix. Proepimeron pilose. Anepisternum widely bare medially, also on dorsal half. Anepimeron entirely pilose. Male genitalia: phallus furcate near apex.

#### Discussion.

Curran (1925a) erected *Serichlamys* as a subgenus of *Microdon*, but subsequent authors considered *Serichlamys* as a synonym of the typic subgenus of *Microdon* (Wirth et al. 1965, Thompson 1981b, Cheng and Thompson 2008). Curran (1925a) did not clearly state which characters he considered diagnostic. In his key, Curran keyed out the type species *Microdon rufipes* (Macquart, 1842) by its eyes being pilose, which was based on a translation of the original description of *Aphritis rufipes*. Indeed, Macquart (1842) wrote that this species has ‘yeux peu velus’ (eyes little pilose). However, examination of the type specimen (coll. OUMNH) revealed that its eyes are bare. Either pile have been wiped off or eroded in the course of time, or Macquart (1842) made an error in his description. Whether *Aphritis rufipes* has pilose eyes or not, *Serichlamys* is here recognized as distinct as all included species differ in other characters from *Microdon* s.s., e.g. postero-apical corner of cell r4+5 rectangular (rounded in *Microdon* s.s.), phallus furcate apically, hypandrium with bulb-like base.

The differences with *Microdon* s.s. could be used as arguments for reinstating the subgeneric status of *Serichlamys*. However, a subgeneric status is contradicted by the phylogenetic results of Reemer and Ståhls (in press), who recovered two Neotropical species of *Serichlamys* as sister group to *Archimicrodon*, without apparent close affinities to *Microdon*. The type species *Serichlamys rufipes* and *Serichlamys scutifer* (Knab, 1917) were included in a phylogenetic analysis based only on morphology, and placed in a large and rather uninformative polytomy, but not within a clade containing species of *Microdon* s.s. For this reason, *Serichlamys* is here raised to genus level.

Three Nearctic species are included in *Serichlamys*: *Serichlamys rufipes* (Macquart, 1842), *Serichlamys scutifer* (Knab, 1917), and *Serichlamys diversipilosus* (Curran, 1925). The latter species is included with uncertainty, based only on the description, as no specimens were examined. Two Neotropical species are included: *Serichlamys mitis* (Curran, 1940) and *Serichlamys mus* (Curran, 1936). The Neotropical species differ from the Nearctic ones in the shape of the surstylus, which has a long posterior process which is lacking in the Nearctic species. Otherwise the species are very similar. Species of *Serichlamys* quite similar to the Old World genus *Archimicrodon* in general habitus and important morphological characters, including the male genitalia. Generally, the antennae of *Serichlamys*-species are longer and the scutellar calcars are longer.

#### Diversity and distribution.

Described species: 4 or 5. Nearctic (2 or 3 described species) and Neotropical (2 described species). Several undescribed species from the Neotropical regioan are known to the first author.

### Spheginobaccha

de Meijere

http://species-id.net/wiki/Spheginobaccha

Figs 377–387

Spheginobaccha de Meijere, 1908: 327. Type species: *macropoda* Bigot, 1883: 331, by monotypy.
Dexiosyrphus Hull, 1944a: 131. Type species: *Spheginobaccha funeralis* Hull, 1944: 131, by original designation. Described as subgenus of *Spheginobaccha*.

#### Description.

Body length: 7–19 mm. Slender flies with short antennae and constricted abdomen. Head about as wide as to wider than thorax. Face in profile straight to slightly concave in dorsal 2/3, with a faint convex tubercle in ventral 1/3; narrower than an eye. Lateral oral margins not produced. Vertex flat. Occiput narrow ventrally, widening dorsally, with distinct crease in dorsal 2/3. Eye bare (African species) or short pilose (Oriental species). Eyes in male not (African species) or strongly (Oriental species) converging at level of frons, in one Oriental species (*Spheginobaccha chilcotti* Thompson) even nearly contiguous. Antennal fossa about twice as wide as high. Antenna shorter than distance between antennal fossa and anterior oral margin. Basoflagellomere longer than scape, oval, except more or less triangularly enlarged in males of some African species; bare. Postpronotum pilose. Scutellum semicircular; without calcars. Anepisternum without sulcus; entirely sparsely pilose, sparsely pilose only posteriorly, or entirely bare. Anepimeron pilose on dorsal half or bare. Katepimeron flat; bare or pilose; smooth. Wing vein R4+5 without posterior appendix. Vein M1 oblique and more or less parallel to wing margin, in African species only so in anterior 1/2, posterior 1/2 straight. Postero-apical corner of cell widely rounded and without appendix in Oriental species, rectangular and with appendix in African species. Crossvein r-m located between basal 1/6 to 1/3 of cell dm. Abdomen constricted, narrowest halfway or at posterior margin of tergite 2, widest at tergite 4. Tergites 3 and 4 fused. Male genitalia: phallus unfurcate, straight (African species) or bent dorsad (Oriental species), articulating with hypandrium apically (*perialla*-group) or basally (*macropoda*- and *rotundiceps*-group); hypandrium with apical part consisting of separate lobes; epandrium without ventrolateral ridge; surstylus unfurcate, oval or more or less rectangular to triangular.

#### Diagnosis.

Metapleura not connected, not forming a postmetacoxal bridge. Abdomen constricted. Occiput with deep crease on dorsal 2/3.

#### Discussion.

Hull (1949) was the first to include *Spheginobaccha* in the Microdontinae. Thompson (1969) excluded it, after which Ståhls et al. (2003) included it again. The latter placement was based on a sister-group relationship of *Spheginobaccha* to all other Microdontinae, as recovered in a phylogenetic analysis of combined molecular and morphological characters. Species can be identified using Thompson (1974), supplemented with Dirickx (1995).

*Dexiosyrphus* was described by Hull (1949) as a subgenus of *Spheginobaccha*, based on *Spheginobaccha rotundiceps* (Loew, 1857). Thompson (1974) argued that if *Dexiosyrphus* was to be recognized, then another subgenus would have to be erected for the *perialla*-group. He considered this unnecessary, as the three species groups he recognized were sufficient for proper segregation of the species. We see no reason to adopt a different point of view.

#### Diversity and distribution.

Described species: 16. Oriental (10 species) and Afrotropical (6 species). Oriental records range from Nepal through Burma, Thailand and Vietnam to Java and Borneo. Afrotropical records are from Malawi, South Africa and Madagascar.

### Stipomorpha

Hull stat. n.

http://species-id.net/wiki/Stipomorpha

Figs 388–396

Stipomorpha Hull, 1945: 74. Type species: *Microdon fraudator* Shannon, 1927, by original designation.

#### Description.

Body length: 6–11 mm. Stingless bee mimicking flies with moderately long antennae and more or less triangular abdomen. Head slightly wider than thorax. Face in profile straight to convex; narrower to wider than an eye. Lateral oral margins hardly to moderately produced. Vertex flat, convex or irregularly swollen. Occiput narrow ventrally, slightly widened dorsally. Eye bare. Eye margins in male converging at level of frons, with mutual distance 1–3 times width of antennal fossa. Antennal fossa about as wide as high. Antenna shorter to longer than distance between antennal fossa and anterior oral margin; basoflagellomere shorter to longer than scape, oval; bare. Postpronotum pilose. Scutellum semicircular, sometimes weakly sulcate apicomedially; without calcars. Anepisternum convex, without sulcus; anterodorsally pilose, posteriorly pilose or bare, widely bare in between. Anepimeron with pile limited to dorsal half, if pilose on ventral half then only sparsely. Katepimeron convex; bare. Wing: vein R4+5 usually with posterior appendix (seldomly missing); vein M1 perpendicular to vein R4+5; postero-apical corner of cell r4+5 widely rounded to rectangular, with or without small appendix; crossvein r-m located between basal 1/5 to 1/3 of cell dm. Abdomen widest at tergite 2, with next tergites either gradually narrowing (kite-shaped abdomen) or more or less parallel-sided; 1.5 to 3.5 times as long as wide. Antetergite almost fused to tergite 1; in most species enlarged, concave and smooth. Tergites 3 and 4 fused. Sternite 1 bare. Male genitalia: phallus unfurcate, bent dorsad, in most species projecting beyond apex of hypandrium; hypandrium with bulb-like base; epandrium without ventrolateral ridge; surstylus in most species with two wide lobes, but other shapes also occur.

#### Diagnosis.

Sternites 2 and 3 separated by membraneous part as wide as or wider than sternite 2.

#### Discussion.

When Hull (1945) erected *Stipomorpha* as a subgenus of *Microdon*, he did so based on the shape of the abdomen: “...the first two abdominal segments greatly flared and flattened and wider than the thorax; remainder of the abdomen immediately compressed into a rounded, subcylindrical pipe-like form.” Shortly after, Hull (1949) ranked *Stipomorpha* as a subgenus of *Paramixogasteroides* Shiraki, 1930, without stating a reason for this. Subsequent authors have regarded *Stipomorpha* as synonymous with *Ubristes*. See under *Ubristes* for a discussion on the relationship between these groups, which are here considered as separate genera. *Stipomorpha* as presently defined contains most species listed under *Ubristes* by Thompson et al. (1976).

#### Diversity and distribution.

Described species: 16. Descriptions of nine additional species are in preparation by the first author. Neotropical, with records ranging from Costa Rica to Argentina.

### Sulcodon

Reemer gen. n.

urn:lsid:zoobank.org:act:1E585145-3AD1-4FCE-8A29-4B18F170E98F

http://species-id.net/wiki/Sulcodon

Figs 397–399

#### Type species:

*Microdon sulcatus* Hull, 1944: 256. Type locality: Java, Soekaboemi.

#### Description.

Body length: 7–9 mm. Broadly built flies with moderately long antennae and short abdomen. Head about as wide as thorax or slightly wider. Face convex; about as wide as an eye. Lateral oral margins distinctly produced. Vertex irregularly swollen. Occiput ventrally narrow, dorsally widened. Eye bare. Eye margins in male converging at level of frons, with mutual distance 2.5 times as large as width of antennal fossa. Antennal fossa about as wide as high. Antenna longer than distance between antennal fossa and anterior oral margin; basoflagellomere about as long as to slightly longer than scape, parallel-sided; bare. Postpronotum bare. Scutellum semicircular; with large, blunt calcars, separated by deep sulcus. Anepisternum weakly sulcate; entirely pilose. Anepimeron entirely pilose. Katepimeron flat; bare. Wing: vein R4+5 with posterior appendix; vein M1 perpendicular to vein R4+5; postero-apical corner of cell r4+5 rectangular, with small appendix; crossvein r-m located around basal 1/4 of cell dm. Abdomen heart-shaped, about as long as wide. Tergites 3 and 4 fused. Sternite 1 bare. Male genitalia: phallus furcate, with furcation point near apex; hypandrium with basal part bulb-like; epandrium without ventrolateral ridge; surstylus deeply furcate.

#### Diagnosis.

Postpronotum bare. Abdomen about as long as wide, with tergite 2 about as long as tergites 3 and 4 together.

#### Discussion.

The only species included in this group, the Oriental *Microdon sulcatus* Hull, 1944, does not have any obvious relatives. Because of the bare postpronotum, the rectangular postero-apical corner of cell r4+5, the entirely pilose anepisternum and the characters of the male genitalia, the species does not fit into *Microdon* s.s.

#### Diversity and distribution.

Described species: 1. Indonesia: Java. The species seems not to be uncommon, as specimens collected by different collectors in different years are present in several entomological collections (BMNH, KBIN, MZH, RMNH, ZMAN). Although entomological collectors have been active in other parts of the Sunda region, such as peninsular Malaysia, Sumatra and Borneo, this species has so far not been found there. This suggests that this singular species is endemic to Java.

#### Etymology.

The generic name is composed of *sulcus* and *odon*. The first part means ‘furrow’ or ‘groove’ in Latin, but in this case it is derived from *Microdon sulcatus*, the type species of the genus. The second part of the name is used as a suffix derived from *Microdon*.

### Surimyia

Reemer

http://species-id.net/wiki/Surimyia

Figs 400–405

Surimyia Reemer, 2008: 179. Type species: *Surimyia rolanderi* Reemer, 2008: 180, by original designation.

#### Description.

Body length: 4–5 mm. Small flies with short antennae and oval abdomen. Head slightly wider than thorax. Face convex; narrower than an eye. Lateral oral margins not produced. Vertex flat. Occiput ventrally narrow, dorsally widened. Eye bare. Eye margins in male not converging at level of frons, with mutual distance about 3 times as large as width of antennal fossa. Antennal fossa about as wide as high. Antenna shorter than distance between antennal fossa and anterior oral margin; basoflagellomere shorter to longer than scape, oval, about twice as long as wide, bare. Postpronotum bare. Scutellum semicircular; without calcars. Anepisternum convex; dorsally with thick, setae-like pile, ventrally bare. Anepimeron dorsally with thick, setae-like pile, ventrally bare. Katepimeron convex; bare. Wing: vein R4+5 without posterior appendix; vein M1 straight, perpendicular to vein R4+5; postero-apical corner of cell r4+5 rectangular, with or without small appendix; crossvein r-m located very close to base of cell dm. Abdomen oval, about 1.5 times as long as wide. Tergites 3 and 4 fused. Sternite 1 bare. Male genitalia: phallus furcate, with furcation point about halfway, curved dorsad, straight, projecting not or slightly beyond apex of hypandrium; hypandrium without bulb-like base; epandrium without ventrolateral ridge; surstylus unfurcate.

#### Diagnosis.

Abdomen oval; yellow and black. Vein R4+5 without posterior appendix. Postpronotum bare. Antenna shorter than distance between antennal fossa and anterior oral margin.

#### Discussion.

Reemer (2008) included in his new genus *Surimyia* a species previously assigned to *Paragodon* (*Paragodon minutula* van Doesburg, 1966). Several morphological characters were mentioned to indicate the differences between these genera (Reemer 2008). Especially the structure of the phallus seems fundamentally different: short, straight and unfurcate in *Paragodon*, and long, curved and bifurcate in *Surimyia*. Other distinctive differences are the bare postpronotum in *Surimyia* (pilose in *Paragodon*) and the bare anatergum in *Surimyia* (microtrichose in *Paragodon*).

#### Diversity and distribution.

Described species: 2. Neotropical (presently only known from Surinam).

##### *Syrphipogon* Hull (subgenus, see *Microdon*)

### Thompsodon

Reemer gen. n.

urn:lsid:zoobank.org:act:C5C25319-6F81-42B6-A805-1B897435AC8B

http://species-id.net/wiki/Thompsodon

Figs 406 –416

#### Type species:

*Thompsodon conspicillifrons* Reemer spec. n. Type locality: Costa Rica.

#### Description.

Body length: 8 mm. Moderately slender flies with long antennae and basally constricted abdomen. Face in profile slightly convex, almost straight; laterally weakly depressed, therefore slightly carinate dorsomedially; about as wide as an eye. Lateral oral margins not produced. Frons laterally with round, concave areas, filled with dense golden pile, ventrally delimited by a sharply defined ridge. Vertex irregularly swollen. Occiput narrow ventrally, strongly widened dorsally. Eye bare. Antennal fossa about as high as wide. Antenna longer than distance between antennal fossa and anterior oral margin; basoflagellomere about as long as scape; elongate, with dorsal margin straight and ventral margin convex, apex slightly acute. Postpronotum pilose. Anepisternum with shallow sulcus; entirely long pilose. Anepimeron entirely pilose. Katepimeron weakly convex; bare; with wrinkled texture. Scutellum semicircular, weakly triangular; without calcars. Wing: vein R4+5 with posterior appendix; vein M1 perpendicular to vein R4+5; postero-apical corner of cell r4+5 rectangular, with small appendix; crossvein r-m located around basal 1/7 of cell dm. Abdomen constricted at tergite 1, narrowest at tergite 1, widest at posterior margin of tergite 3. Tergites 3 and 4 not fused, able to articulate independently.

#### Diagnosis.

Frons laterally with round, concave areas, filled with dense golden pile, ventrally delimited by a sharply defined ridge. Transverse suture complete. Tergites 3 and 4 not fused.

#### Discussion.

The only known specimen representing this genus has some characters that are not often found among Microdontinae: mesonotal transverse suture complete, tergites 3 and 4 not fused. The lateral concave and densely golden pilose areas on the frons, which are ventrally delimited by a sharply defined ridge, are even unique within the subfamily. The unfused tergites 3 and 4 may suggest affinity with the Neotropical *Ceratophya* or the Oriental *Kryptopyga*, whereas the complete transverse suture reminds of *Ceratrichomyia* and*Indascia*. However, the specimen does not agree with the diagnoses of any of these genera, so a new genus seems the best way to make sure that this singular taxon will get the attention it deserves in future studies on the taxonomy of Microdontinae. Hopefully, male specimens will be collected in the near future, which can be used for study of the male genitalia and molecular analyses.

#### Diversity and distribution.

Described species: 1. Only known from Costa Rica.

#### Etymology.

This genus is dedicated to Dr. F. Christian Thompson, in acknowledgement of the valuable work he has done on the taxonomy of the Syrphidae in general, and the Microdontinae in particular.

### Ubristes

Walker

http://species-id.net/wiki/Ubristes

Figs 417–421

Ubristes Walker, 1852: 217. Type species: *Ubristes flavitibia* Walker, 1852: 217, by original designation.

#### Description.

Body length: 10–11 mm. Slender flies with long antennae and long, brush-like pilosity on hind tibiae. Mimics of *Trigona*-like stingless bees. Head wider than thorax. Face slightly convex, almost straight in lateral view; wider than eye. Lateral oral margins produced. Vertex flat. Occiput ventrally narrow, dorsally widened. Eye very sparsely and short pilose, appearing bare under low magnification. Eye margins in male converging at level of frons; mutual distance about three times width of antennal fossa. Antennal fossa about as high as wide. Antenna longer than distance between antennal fossa and anterior oral margin; basoflagellomere longer than scape. Postpronotum pilose. Anepisternum sulcate; pilose anteriorly and posteriorly, widely bare in between. Katepimeron convex; bare. Scutellum semicircular; without calcars. Wing: vein R4+5 with posterior appendix; vein M1 perpendicular to vein R4+5; cell r4+5 with postero-apical angle widely rounded; crossvein r-m located between basal 2/5 and 1/2 of cell dm. Hind tibia with long, brush-like pilosity. Abdomen elongate: parallel-sided or somewhat triangular. Tergite 2 with lateral tubercle at half of length. Tergites 3 and 4 fused. Sternites 1, 2 and 3 not separated by very wide membranes. Male genitalia: phallus furcate basally; epandrium with lateral ‘fenestrae’: well-defined, translucent, oval depressions; surstylus more or less oval.

#### Diagnosis.

Hind tibia with long, brush-like pilosity. Scutellum without calcars. Vein R4+5 with appendix. Tergite 2 with lateral tubercle at half of length.

#### Discussion.

Thusfar, *Ubristes* has been characterized by the brush-like pilosity of the hind tibia, giving the flies the appearance of stingless *Trigona*-like bees (Cheng and Thompson 2008, Thompson et al. 1976). Based on this definition, 31 species were assigned to this group by Thompson et al. (1976), including the type species of *Carreramyia*, *Hypselosyrphus* and *Stipomorpha*. The latter two groups were considered as ‘subgroups’ of *Ubristes* by Cheng and Thompson (2008), because the characters previously used to define the groups (abdominal shape) were considered of little taxonomic value.

Closer examination of the morphology reveals several important differences between these taxa. The structure of the male genitalia of *Ubristes* is very different from those of the species here included in *Carreramyia*, *Hypselosyrphus* and *Stipomorpha*: the phallus is long and slender and furcate near its base, the base of the hypandrium is not bulged and there are well-defined, translucent, oval lateral depressions in the epandrium (here called ‘fenestrae’). In external morphology *Ubristes* is readily distinguished from the mentioned genera by e.g. the lateral tubercles on tergite 2. For other differences see the accounts of the other taxa. Considering the phylogenetic results of Reemer and Ståhls (in press) and the morphological differences between these taxa, *Ubristes* sensu Thompson et al. (1976) and Cheng and Thompson (2008) is here considered to be polyphyletic, with *Carreramyia*, *Hypselosyrphus* and *Stipomorpha* each as separate lineages. Besides the type species, two undescribed species are assigned to *Ubristes*.

Thompson et al. (1976) and Cheng and Thompson (2008) ranked *Ubristes* as a subgenus of *Microdon*. However, the species of *Ubristes* differ in several characters from the species of *Microdon* s.s., as defined in the present paper. Here, the view is taken that it is better to treat *Ubristes* as a genus instead of a subgenus, in order to make sure that *Microdon* comprises less heterogeneous groups with uncertain affinities.

#### Diversity and distribution.

Described species: 1. Descriptions of two additional species are in preparation by the first author. Central and South America.

### Undescribed genus #1

Figs 422–426

Based on species AUS-01 of F.C. Thompson, in prep.

#### Diagnosis.

Basoflagellomere strongly widened, more or less triangular. Arista pilose.

#### Discussion.

This species has first been recognized as undescribed by Dr. F.C. Thompson, who is preparing a description of it. As the species posesses some unique characters not found in any other species of Microdontinae, the present authors feel that it belongs in a new genus. These characters are: basoflagellomere strongly widened and more or less triangular; arista pilose; phallus dorsobasally with long projection; epandrium with dorsolateral ridge. Other interesting characters are the undeveloped mouthparts (shared with *Masarygus*) and the lateral carinae on the face.

#### Diversity and distribution.

Described species: 1. Australia (Queensland).

### Undescribed genus #2

Figs 427 –432

Based on species MCR-2 of F.C. Thompson, in prep.

#### Diagnosis.

Basoflagellomere bifurcate. Abdomen more or less parallel-sided, slightly constricted between tergites 2 and 3.

#### Discussion.

This species has first been recognized as undescribed by Dr. F.C. Thompson, who is preparing a description of it. This taxon resembles *Carreramyia* in the bifurcate antenna, the wing venation and the structure of the male genitalia. It differs from that genus by the more or less flat vertex (strongly produced in *Carreramyia*), the short pilose hind tibia (long pilose in *Carreramyia*), and the more or less parallel-sided, slightly constricted abdomen (triangular in *Carreramyia*).

#### Diversity and distribution.

Only known from one species, collected in Costa Rica.

### Unplaced taxa

A small number of species is left unclassified. These are listed at the end of the following section on species classification. On a few of these taxa, comments are given below.

### Microdon sharpii

Mik, 1900

Figs 433–434

#### Discussion.

Based on external characters, no close relatives were recovered in the phylogenetic analysis of morphological characters by Reemer and Ståhls (in press). The species is characterized by its metallic blue colouration and golden pilosity, a long basoflagellomere, a medially widely bare face, a rectangular postero-apical corner of wing cell r4+5, and unfused tergites 3 and 4. The latter character may indicate affinity with *Ceratophya*, *Kryptopyga* or *Thompsodon*, but the species lacks other diagnostic characters for these taxa. This species is left unplaced for now.

### Nothomicrodon

Wheeler, 1924

#### Discussion.

Whether this taxon belongs to Microdontinae or Syrphidae at all is uncertain. It was described from larvae found in an ants nest (Wheeler 1924). Cheng and Thompson (2008) suspect it belongs to another family, perhaps Phoridae. Photographs of a larva can be found at http://syrphidae.lifedesks.org/pages/25523 .

## Discussion: diagnostic characters of Microdontinae

In order to find diagnostic characters for distinguishing Microdontinae from other Syrphidae, characters described by Hippa and Ståhls (2005), Hull (1949), Shatalkin (1975a, b), Speight (1987) and Thompson (1969, 1972) were evaluated based on the material examined for this study. The discussion of these characters below is subdivided into paragraphs corresponding with the following main body parts: head, thorax, wings, legs, abdomen, male genitalia. Terminology of the aforementioned authors is translated into the terminology used in the present paper (see section Material and Methods). This discussion concludes with a summarizing statement on diagnostic morphological characters of Microdontinae.

### Head

The simple, convex face of most Microdontinae has been used as a character for the group by Hull (1949) and Thompson (1969, 1972). A facial tubercle is only found in *Eurypterosyrphus*. In a few taxa (*Ceratrichomyia*, *Chrysidimyia*, *Rhopalosyrphus*) the ventral part of the face is somewhat bulged, but cannot be considered tuberculate. The diagnostic value of this character is limited, as the facial tubercle is also missing in several other Syrphidae, e.g. all Pipizini and Eumerini.

According to Thompson (1969, 1972), the face of Microdontinae is uniformly pilose. In the present study, however, several taxa were found in which the face is bare medially to varying extent (e.g. species of *Rhoga*, *Schizoceratomyia*, *Stipomorpha*), sometimes even entirely bare (e.g. *Masarygus planifrons*).

Thompson (1972) noted that the anterior oral margin in Microdontinae is not notched. There seems to be some confusion about the interpretation of this character, as Hippa and Ståhls (2005) have coded this character as ‘medially notched’ for *Microdon analis* (Macquart, 1842). Apparently, the latter authors have regarded the anterior oral margin as notched when the lateral oral margins are produced. Indeed, these laterally produced oral margins give the impression in anterior view that the anterior oral margin is narrowed (notched). In most other Syrphidae (except Pipizini), however, there is a slight additional narrowing at the extreme anterior part of the oral margin (see e.g. fig. 3 in Speight 1987 and fig. 1A in Hippa and Ståhls 2005). This additional narrowing seems to be correlated with the presence of an anteclypeus. In Microdontinae, no distinction can be made between an anteclypeus and a postclypeus (see below), so this additional narrowing of the anterior margin is not visible. In the present study, we follow Hippa and Ståhls (2005) in the interpretation of this character. Many Microdontinae were found with produced lateral oral margins, as it is in most other Syrphidae, so this character is not considered to be useful for distinguishing the subfamily from other Syrphidae.

According to Speight (1987), Microdontinae possess only one clypeus, whereas an anteclypeus and a postclypeus can be recognized in other Syrphidae. The presence of only one clypeus in Microdontinae can be confirmed based on the present study, but the character has not been studied in other Syrphidae. Speight (1987) mentioned two other characters of the mouthparts he considered to be unique for *Microdon*: 1. the maxillary sclerites are short, flange-like, oriented transversely rather than longitudinally; 2. the maxillary palps are rudimentary. These characters have not been studied in the present study, and thus cannot be commented upon. In general, the mouthparts of Microdontinae are reduced compared with other Syrphidae. No characters indicating the degree of reduction were included in the present study, but a considerable degree of variation was noticed. In certain taxa, the labella are well-developed and flattened, suggesting a capability of feeding on flat surfaces (e.g. leaves) (this can best be noticed in fresh or alcohol-preserved specimens, as the mouthparts tend to shrivel up when dry). In other taxa, the mouthparts are reduced to such an extent that there is not even an oral opening, indicating these species do not feed at all (*Masarygus palmipalpus* sp. n., *Masarygus planifrons*).

Unlike most other Syrphidae, the males are dichoptic (i.e. the eyes do not meet at the top of the head). In the present study, no holoptic Microdontinae were found except for *Spheginobaccha chillcotti* Thompson, 1974, although in a few taxa the male eyes approach each other quite closely (e.g. *Hypselosyrphus*). When taken into consideration that dichoptic males also occur in other subfamilies of Syrphidae (e.g. *Helophilus* Meigen, 1822 and related genera, *Neoascia* Williston, 1887, *Pelecocera* Meigen, 1822), this character has limited diagnostic value.

According to Thompson (1969, 1972) the arista of Microdontinae is bare. The only known exception, as found in the present study, is the Australian Undescribed genus #1. As a bare arista also occurs in many other Syrphidae, this character is of limited diagnostic value.

### Thorax

A pilose postpronotum has been considered to be an important and stable character for distinguishing Microdontinae (as well as Eristalinae) from Syrphinae (Thompson 1969, 1972). In the present study, the postpronotum was found to be pilose in the majority of Microdontinae, but certainly not in all. The postpronotum is bare in several taxa (e.g. *Ceriomicrodon petiolatus*, *Masarygus*, *Sulcodon*, *Surimyia*, *Paramixogaster*, *Piruwa*, *Schizoceratomyia*). This needs to be taken into account when using keys to genera of Syrphidae in which this character is used (e.g. Thompson 1999).

A few other characters involving the presence or absence of pile on thoracic sclerites have been used. Thompson (1969, 1972) noted that the anterior part of the anepisternum is pilose in Microdontinae, except in *Ceriomicrodon petiolatus*. In addition, a bare anterior anepisternum was found in an *Aristosyrphus* sp. n., a *Mixogaster* sp. n. and in some species of *Spheginobaccha*. According to Hull (1949) the metasternum is always pilose in Microdontinae. However, this was only true for slightly more than half of the presently studied taxa. The subscutellar hair fringe was absent in all studied Microdontinae (character of Thompson 1969, 1972). This character also applies to several other Syrphidae (Hippa and Ståhls 2005), so it is not by itself group-defining, although it could be useful in keys.

Another thoracic character considered of importance for Microdontinae (Thompson 1969, 1972) is the presence of a complete ‘postmetacoxal bridge’, formed by the connection of the metapleura. As already observed by Cheng and Thompson (2008), this bridge is lacking in *Spheginobaccha*. The present study revealed that the metapleura are also distinctly separated in certain species of *Rhoga* (*Rhoga maculata*, *Rhoga mellea*, *Rhoga sepulchrasilva*). In two other taxa (*Paramixogaster variegata*) and *Surimyia*) the metapleura seem to be touching only in one point, implying an intermediate state for this character. Among other Syrphidae, a complete postmetacoxal bridge is rare, but it is found in e.g. *Baccha elongata* (Fabricius, 1775), *Neoascia* Williston, 1886 and *Sphegina* Meigen, 1822 (Hippa and Ståhls 2005), and also in *Leucopodella* Hull, 1949.

The well-developed plumule, a plumose posterior extension of the subalar sclerite, is considered to be an important character of Syrphidae. In most Syrphinae and Eristalinae the plumule is usually strongly developed, except in *Ceriana* Rafinesque, 1815, *Sphiximorpha* Rondani, 1850, *Neoascia*, *Sphegina*, *Eosphaerophoria* Frey, 1946, *Allograpa ventralis* (Miller, 1921) and some species of *Ocyptamus* (Hippa and Ståhls 2005, Speight 1987, X. Mengual pers. comm.). As noticed by Thompson (1969, 1972), Speight (1987) and Hippa and Ståhls (2005), the plumule is strongly reduced in Microdontinae. This is confirmed by the results of the present study, although considerable variation was found. In a few taxa, the plumula is entirely absent (e.g. *Carreramyia*, *Masarygus*, *Spheginobaccha*), while in others a short plumula can be found, with both the length of this sclerite and the microtrichosity varying in length.

Speight (1987) drew attention to another character: “At the outer ends of the transverse sulcus of the mesoscutum, *Microdon* possesses a pair of shelf-like, semi-circular, sclerotized outgrowths of the mesoscutum, which do not seem to have an equivalent in other Syrphids”. This apparently indicates the notal wing lamina, which, however, is also well-developed in certain other syrphids besides *Microdon*, as noted by Hippa and Ståhls (2005). The present data indicate that the notal wing lamina is undeveloped in several Microdontinae, such as *Aristosyrphus*, *Eurypterosyrphus*, *Masarygus*, *Paragodon*, *Rhoga* and species of *Hypselosyrphus*, *Indascia* and *Paramixogaster*. A strongly developed notal wing lamina (in the sense of Hippa and Ståhls 2005) was only found in *Chrysidimyia*. This character has little diagnostic value for the Microdontinae as a subfamily.

As Speight (1987) noticed, the subscutellum (metanotum) is “unusually flat” in *Microdon*, whereas in many other Syrphidae often a convex plate is present. This character was found to be variable among Microdontinae, but in this group the subscutellum is never as strongly swollen as in several other Syrphidae. However, as many intermediate states occur, this character cannot be used conveniently as diagnostic at the subfamily level.

### Wings

The presence of the stigmal crossvein was mentioned as a character of the Microdontinae by Hull (1949) and Thompson (1969). The only exceptions found in the present dataset are *Spheginobaccha* and *Paramicrodon delicatulus* Hull, 1937 (the crossvein is present in other studied species of *Paramicrodon*). A quick but far from exhaustive scan of this character among other Syrphidae learned that the stigmal crossvein is also present in many Eristalinae.

Hull (1949) and Thompson (1969) noted that the apical crossveins M1 and dm-cu are positioned perpendicular to, respectively, vein R4+5 and vein M in most Microdontinae. Exceptions are *Aristosyrphus*, *Mixogaster*, *Spheginobaccha*, and to a lesser extent *Kryptopyga* and *Schizoceratomyia*, in which the anterior 1/3 or 1/2 is directed outward. Among other Syrphidae, perpendicular marginal crossveins can be found in e.g. *Neoascia* and *Ocyptamus* Macquart, 1834 (subgenus *Calostigma* Shannon, 1927).

In all Microdontinae, as noticed by Thompson (1969), crossvein r-m is positioned basal of the middle of cell dm. This is not an exclusive character of the subfamily, however, as it is shared with all Syrphinae and many Eristalinae.

An apparently universal character for Microdontinae is the basally curved vein R2+3. The first to introduce this character were Hippa and Ståhls (2005), who noted that the only other Syrphidae in which this character is found are the Cerioidini. No exceptions were found in the present dataset. In the present paper, an attempt is made to describe this important character in a way that makes it easier to judge it objectively (Fig. 435).

### Legs

The legs of most Microdontinae are marked with clear scars subbasally at the femora and subapically at the tibia, visible as creases surrounding the legs. These scars are named cicatrices, singular cicatrix (Hull 1949, Thompson 1969). In Microdontinae, this character is usually very pronounced, but a few exceptions were found among the studied taxa (e.g. *Masarygus palmipalpus*, *Piruwa phaecada*, *Schizoceratomyia flavipes*). These taxa are small in body size, and cicatrices are present in taxa which are considered closely related (e.g. *Schizoceratomyia barretoi*). This suggests that the apparent absence of cicatrices might merely be a matter of reduction or reduced visibility of the character. Vague cicatrices can also be seen in several Syrphinae and Eristalinae, although never as clear as in Microdontinae. With these considerations in mind, the character holds a good ‘indicating value’ for diagnosing the subfamily, but it should be applied with caution.

Speight (1987) found that all Syrphidae except *Microdon* posess a long, blade-like process projecting outwards from the antero-lateral end of the outer side of the posterior mesocoxite, which he termed “trochanteral process of the mesocoxite”. This character has not been examined in the present study.

### Abdomen

In Microdontinae, four preabdominal segments are found in the male, as has been noted by many previous authors (e.g. Thompson 1969, Hippa and Ståhls 2005). This character is shared with the Eristalinae, but constitutes a difference with the Syrphinae. No exceptions were found.

Another abdominal character, noted by Thompson (1969) is the position of the first abdominal spiracle, which is embedded in the metaepimeron in Microdontinae. In the present study, this character was confirmed for most taxa. In a few small taxa the character could not be verified because the spiracle could not be found, neither in the metaepimeron nor in the adjacent membranes. The diagnostic value of this character is limited, as the first abdominal spiracle is also embedded in the metaepimeron in many Syrphinae and Eristalinae (Hippa and Ståhls 2005).

### Male genitalia

The last published characterization of genitalia of Microdontinae is the one of Thompson (1969, with some additional notes in 1972). Although since then the understanding of the homologies of Diptera genitalic structures and their terminology has advanced (McAlpine 1981, Sinclair 2000), the characters listed by Thompson (1969) to distinguish Microdontinae from other Syrphidae are still useful. Some of these characters have also been noticed by other authors (Shatalkin 1975a, b, Speight 1987).

Most of the singularities of the genitalia of Microdontinae are found in the hypandrium (9th sternum) and its associated structures. The hypandrium itself is a simple structure in Microdontinae, lacking separate lobes.

In most taxa, the hypandrium seems to consist of a basal part and an apical part. In certain species this distinction is very clear, because the basal part is convex in lateral view (e.g. Figs 24, 363, 396), but in other ones these parts are smoothly fused and one needs to look carefully to distinguish them (e.g. Figs 171, 179, 319). However, distinction is possible in most cases because the apical part is usually less sclerotized than the basal part and it is covered with very fine microtrichia, while on the basal part these are lacking. There is no doubt that the basal part is the actual hypandrium, because it articulates with the epandrium basolaterally. Possibly, the apical part is homologous to the gonopods of other Diptera, which are usually simple in Muscomorpha and more or less absent in Syrphoidea (McAlpine 1981). In most Microdontinae the apical part consists of one single structure. If this structure is homologous to the gonopods indeed, then this would imply that the gonopods have become fused. In a few taxa (with a basal position in the phylogeny presented in Reemer and Ståhls in press), the apical part of the hypandrium consists of two separate lobes, e.g. in *Aristosyrphus* (incl. *Eurypterosyrphus*), *Mixogaster* and *Spheginobaccha* (Figs 29, 32, 246, 387). In these cases it is easier to imagine that these structures are homologous to gonopods. In only one studied taxon, *Menidon falcatus*, no apical part of the hypandrium seems to be present.

No postgonites (parameres of McAlpine 1981, superior lobes of Metcalf 1921 and Hippa and Ståhls 2005) can be distinguished in Microdontinae, a rare occasion among Diptera according to McAlpine (1981). Hippa and Ståhls (2005) supposed that in this subfamily the parameres are integrated into the phallus, but did not present evidence for this hypothesis.

The phallus (subdivided by Thompson 1969 into ejaculatory duct and ejaculatory hood) is tubular and elongate. Its structure is simple: no separate structures can be recognized, as is possible in other Syrphidae (basiphallus, distiphallus etc.). In most taxa, the basal part (termed ‘chitinous box’ in Metcalf 1921 and Thompson 1969) is swollen and spherical (e.g. Figs 29, 363, 385, 405), but in a few this is not obviously so (Figs 22, 386). This basal part might be formed out of the phallapodeme, as Thompson (1974) appears to suggest for *Spheginobaccha*. However, this seems unlikely, because in other Diptera the phallapodeme does not seem to have a sperm-guiding or -collecting function, while in Microdontinae the spherical base of the phallus clearly has an intermediate position between the sperm duct and the apical part of the phallus. Usually, no external lobes are present, but in some taxa a dorsobasal projection was found (Fig. 22). The aedaegus can be unfurcate or bifurcate. Furcate aedeagi can be divided into a number of types, depending on whether the furcation point is basal or apical, and on the length of the ejaculatory processes (see character nos. 163–165 in Reemer and Ståhls in press).

The phallus, or actually the ejaculatory hood, articulates ventrally with the hypandrium and dorsally with the subepandrial sclerites. The point of articulation with the hypandrium is basal, in contrast with all other Syrphidae. The only studied microdontine taxon in which the phallus was observed to articulate apically with the hypandrium is the African taxon *Spheginobaccha guttula* Dirickx, 1995, a representative of the *perialla*-group of Thompson (1974).

Except for the studied African species of *Spheginobaccha*, *Spheginobaccha guttula* and *Spheginobaccha dexioides* Hull, 1944, none of the studied Microdontinae has a clearly recognizable phallapodeme. In the Oriental species of *Spheginobaccha* this structure is also more or less spherical. According to Thompson (1972), the phallapodeme can be absent or “double” in this subfamily. No explanation is given, but judging from a figure of the genitalia of *Microdon manitobensis* Curran, 1924 in Thompson and Rotheray (1998) and Vockeroth and Thompson (1987), the phallapodeme in the sense of Thompson corresponds with the dark lines named ‘lateral strips’ in Reemer and Ståhls (in press, character no. 171). This seems unlikely, because the phallapodeme is a single structure while the lateral strips are paired.

Thompson (1969, 1972) pointed out that the ejaculatory apodeme of Microdontinae is ‘triangularly flared’ apically, except in *Paragodon*, in which it is not sclerotized. The present study has revealed no other taxa with an unsclerotized ejaculatory apodeme. The shape of this structure was found to be very variable, ranging from elongate, round, trapezoid, triangular, square to rectangular. The ejaculatory sac was found to be sclerotized in all taxa except *Paragodon* and *Surimyia*. This structure is also variable in shape.

No characters useful for diagnostic purposes at subfamily level were found in the epandrium and associated structures. The shapes of the cerci and surstyli are highly variable, so much even that it is difficult to use them at generic level.

### Summarizing statement

When the characters of Microdontinae described by previous authors are studied across a large set of taxa, as has been done in the present study, exceptions can be found for almost all of them. Characters for which no or few exceptions were found are listed in Table 2. The character of the basal shape of vein R2+3 seems to be the most exclusive external character to separate the subfamily from other Syrphidae. A key to distinguish Microdontinae from other Syrphidae is given below. As not all Syrphidae have been studied, doubtful cases may occur, so it is recommended to verify at least a few of the other characters in Table 2, preferably those of the male genitalia.

**Table 2.** Characters considered to be of good diagnostic value for separating Microdontinae from other Syrphidae, with indication of known exceptions. See text for Discussion.

**Character statement**	**State in Microdontinae**	**Exceptions**	**State in other Syrphidae**	**Exceptions**
**Head**				
eyes of male, contiguity	dichoptic	*Spheginobaccha chillcotti*	usually holoptic	several
**Thorax**				
postpronotum, pilosity	present	several, e.g. *Masarygus*, *Surimyia*, *Paramixogaster*	Syrphinae: bare<br/> Eristalinae: pilose	a few Syrphinae (e.g. *Allobaccha* Curran, 1928)
postmetacoxal bridge, presence	present	*Rhoga* (in part), *Spheginobaccha*	absent	e.g. *Baccha elongata* (Fabricius, 1775), *Leucopodella* Hull, 1949, *Neoascia*, *Sphegina*
plumule, degree of development	short or absent	none	long	Cerioidini, *Neoascia*, *Sphegina*, *Eosphaerophoria* Frey, 1946, *Allograpta ventralis* (Miller, 1921), *Ocyptamus* Macquart, 1834 in part.
**Wing**				
stigmal crossvein, presence	present	*Paramicrodon delicatulus*, *Spheginobaccha*	Syrphinae: absent<br/> Eristalinae: variable	unknown
vein R2+3, shape basal part	strongly curved (Fig. 435: angle A > angle B)	none	weakly curved (Fig. 435: angle A < angle B)	Cerioidini
**Legs**				
femora and tibiae, presence of subbasal and subdistal cicatrices	present	*Masarygus palmipalpus*, *Piruwa phaecada*, *Schizoceratomyia flavipes*	absent or weakly developed	none
**Abdomen**				
abdomen, number of preabdominal segments	four	none	Syrphinae: five, except four in Pipizini<br/> Eristalinae: four	none
**Male genitalia**				
postgonites, presence	absent	none	present	none
phallus, point of articulation with hypandrium	basal	*Spheginobaccha guttula*	apical	none
phallus, apical part, shape	tubular, elongate, without separate structures (often furcate)	none	rarely elongate, usually with separate structures	
phallus, basal part, shape	usually spherical	*Archimicrodon*	never spherical	none
phallapodeme, presence	absent	*Spheginobaccha* (African taxa only)	present	none

**Table:** 

1	Vein R2+3 weakly curved basally: angle A < angle B (Fig. 435)	Syrphinae and Eristalinae (ex. Cerioidini)
–	Vein R2+3 strongly curved basally: angle A > angle B (Fig. 435)	2
2	Antenna with terminal arista. Male holoptic.	Eristalinae (Cerioidini)
–	Antenna with dorsal arista, or without arista. Male dichoptic	Microdontinae

## Supplementary Material

XML Treatment for Afromicrodon

XML Treatment for Archimicrodon

XML Treatment for Aristosyrphus

XML Treatment for Bardistopus

XML Treatment for Carreramyia

XML Treatment for Ceratophya

XML Treatment for Ceratrichomyia

XML Treatment for Ceriomicrodon

XML Treatment for Cervicorniphora

XML Treatment for Chrysidimyia

XML Treatment for Domodon

XML Treatment for Furcantenna

XML Treatment for Heliodon

XML Treatment for Hypselosyrphus

XML Treatment for Indascia

XML Treatment for Kryptopyga

XML Treatment for Laetodon

XML Treatment for Masarygus

XML Treatment for Menidon

XML Treatment for Mermerizon

XML Treatment for Metadon

XML Treatment for Chymophila

XML Treatment for Dimeraspis

XML Treatment for Megodon

XML Treatment for Microdon

XML Treatment for Myiacerapis

XML Treatment for Syrphipogon

XML Treatment for Mixogaster

XML Treatment for Oligeriops

XML Treatment for Omegasyrphus

XML Treatment for Paragodon

XML Treatment for Paramicrodon

XML Treatment for Paramixogaster

XML Treatment for Parocyptamus

XML Treatment for Peradon

XML Treatment for Piruwa

XML Treatment for Pseudomicrodon

XML Treatment for Ptilobactrum

XML Treatment for Rhoga

XML Treatment for Rhopalosyrphus

XML Treatment for Schizoceratomyia

XML Treatment for Serichlamys

XML Treatment for Spheginobaccha

XML Treatment for Stipomorpha

XML Treatment for Sulcodon

XML Treatment for Surimyia

XML Treatment for Thompsodon

XML Treatment for Ubristes

XML Treatment for Undescribed genus #1

XML Treatment for Undescribed genus #2

XML Treatment for Microdon sharpii

XML Treatment for Nothomicrodon

XML Treatment for Archimicrodon malukensis

XML Treatment for Ceratrichomyia angolensis

XML Treatment for Ceratrichomyia behara

XML Treatment for Ceratrichomyia bullabucca

XML Treatment for Domodon zodiacus

XML Treatment for Furcantenna nepalensis

XML Treatment for Heliodon doris

XML Treatment for Heliodon elisabethanna

XML Treatment for Heliodon tiber

XML Treatment for Indascia gigantica

XML Treatment for Indascia spathulata

XML Treatment for Kryptopyga sulawesiana

XML Treatment for Masarygus palmipalpus

XML Treatment for Mermerizon inbio

XML Treatment for Metadon achterbergi

XML Treatment for Microdon hauseri

XML Treatment for Microdon mandarinus

XML Treatment for Microdon yunnanensis

XML Treatment for Paramixogaster piptotus

XML Treatment for Piruwa phaecada

XML Treatment for Pseudomicrodon polistoides

XML Treatment for Pseudomicrodon smiti

XML Treatment for Rhopalosyrphus (s.s.) ecuadoriensis

XML Treatment for Rhopalosyrphus (s.s.) robustus

XML Treatment for Rhopalosyrphus (s.l.) abnormoides

XML Treatment for Rhopalosyrphus (s.l.) oreokawensis

XML Treatment for Thompsodon conspicillifrons

## Figures and Tables

**Figures 3–10.**
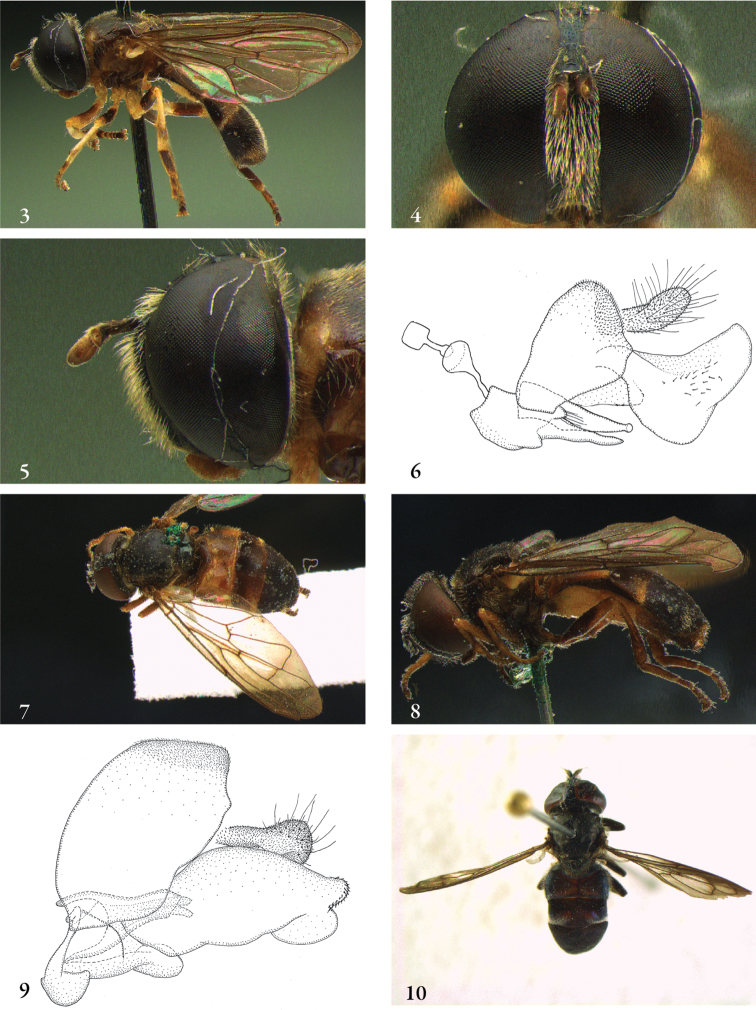
**3–5** *Afromicrodon madecassa* male (holotype): **3** habitus dorsal **4** head frontal **5** head lateral **6** *Afromicrodon johannae* male (paratype), genitalia lateral **7–9** *Archimicrodon* (s.s.) *simplicicornis* male (holotype): **7** habitus dorsal **8** habitus lateral **9** genitalia lateral **10** *Archimicrodon* (s.s.) *malukensis* male (holotype), habitus dorsal.

**Figures 11–19. F4:**
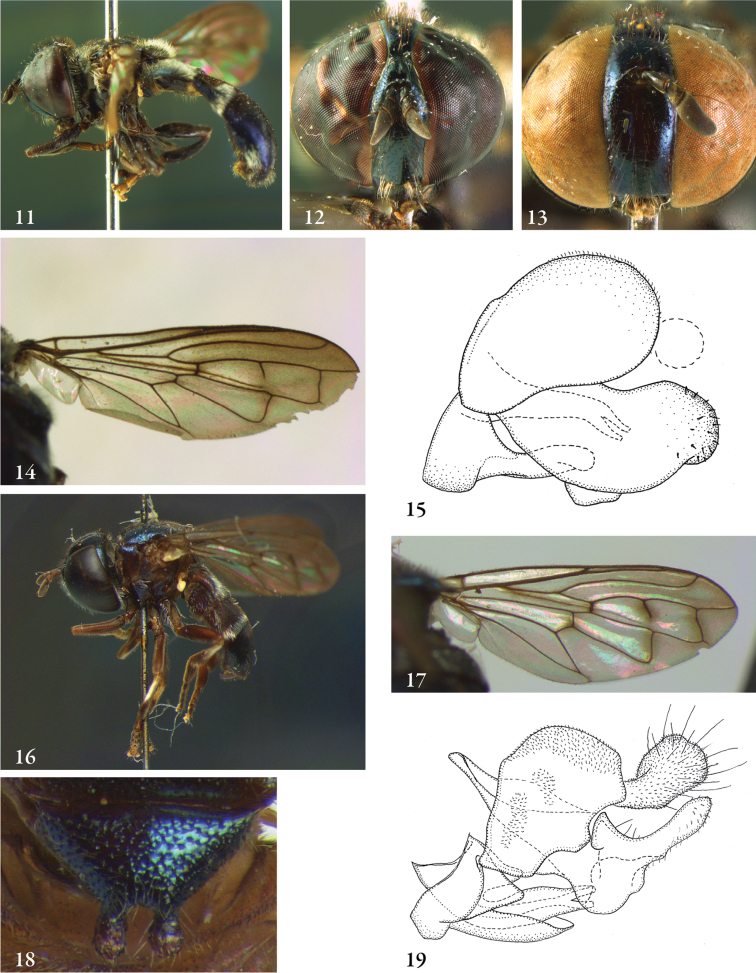
**11–15** *Archimicrodon* (s.s) *malukensis*: **11** male (holotype), habitus lateral **12** male (holotype), head frontal **13** female (paratype), head frontal **14** male (holotype, wing **15** male (paratype), genitalia lateral **16–17** *Archimicrodon (Hovamicrodon) silvester* male (holotype): **16** habitus lateral **17** wing **18–19** *Archimicrodon (Hovamicrodon)*: **18** *Archimicrodon nubecula* female (holotype), scutellum **19** *Archimicrodon silvester* male (holotype), genitalia lateral.

**Figures 20–26.**
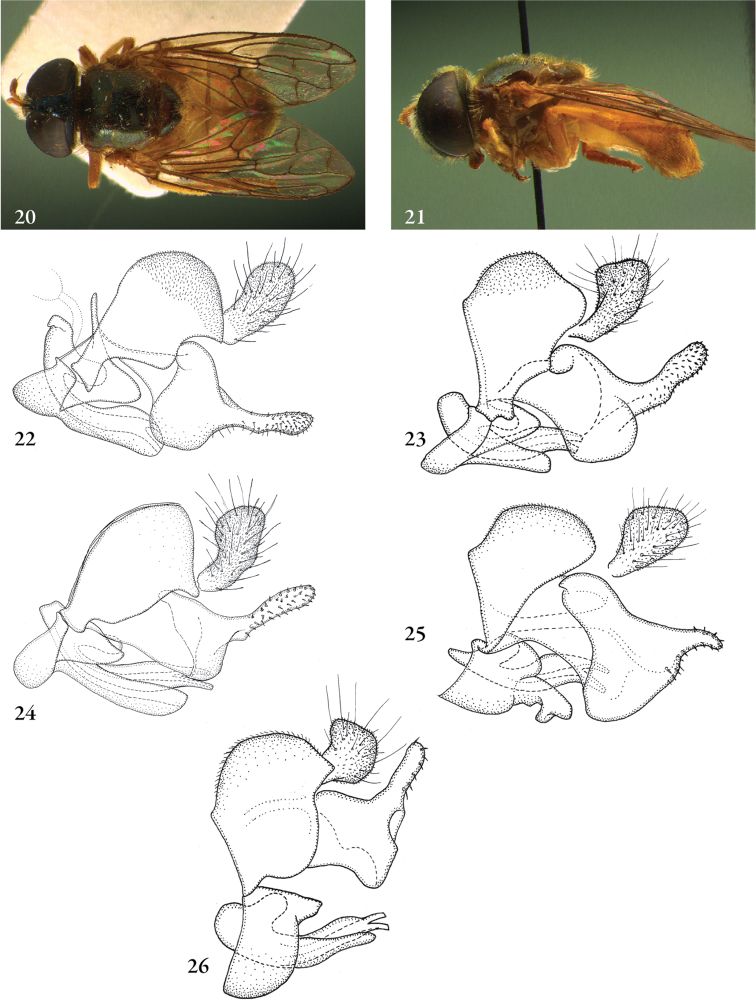
**20–22** *Archimicrodon* (s.l.) *fenestrellatus* male (holotype): **20** habitus dorsal **21** habitus lateral **22** genitalia lateral **23–26** *Archimicrodon* (s.l.), male genitalia lateral: **23** *Archimicrodon brevicornis* (syntype) **24** *Archimicrodon simplex* (South Korea, coll. RMNH) **25** *Archimicrodon venosus* (holotype *Microdon papuanus* van Doesburg, jun. syn.) **26** *Archimicrodon* cf. *fergusoni* (Australia, coll. CSCA).

**Figures 27–34.**
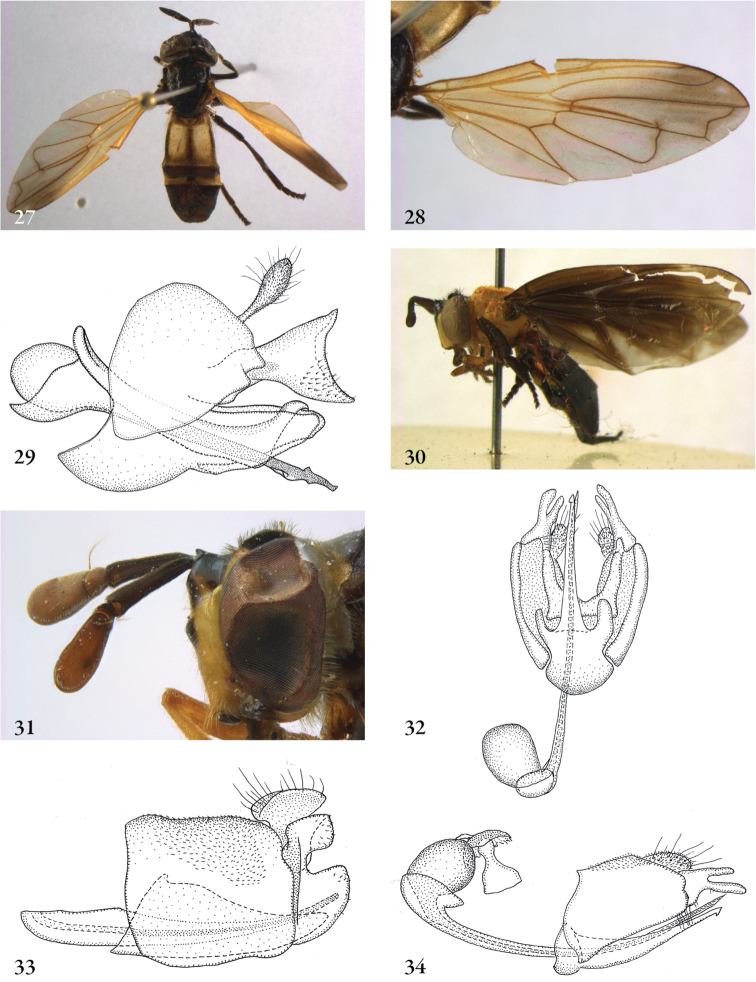
**27–29** *Aristosyrphus primus* male: **27** habitus dorsal (Brazil, coll. J.T. Smit) **28** wing (Brazil, coll. J.T. Smit) **29** genitalia lateral (Brazil, coll. SEMC) **30–34** *Aristosyrphus (Eurypterosyrphus)* males: **30** *Aristosyrphus currani* male (holotype), habitus lateral **31** *Aristosyrphus*. spec. (Brazil, coll. ZMAN), head lateral **32** *Aristosyrphus* spec. #1 (Costa Rica, coll. ZMAN), genitalia ventral **33** *Aristosyrphus* spec. #2 (Brazil, coll. ZMAN), genitalia lateral **34** *Aristosyrphus* spec. #1 (Costa Rica, coll. ZMAN), genitalia lateral.

**Figures 35–42.**
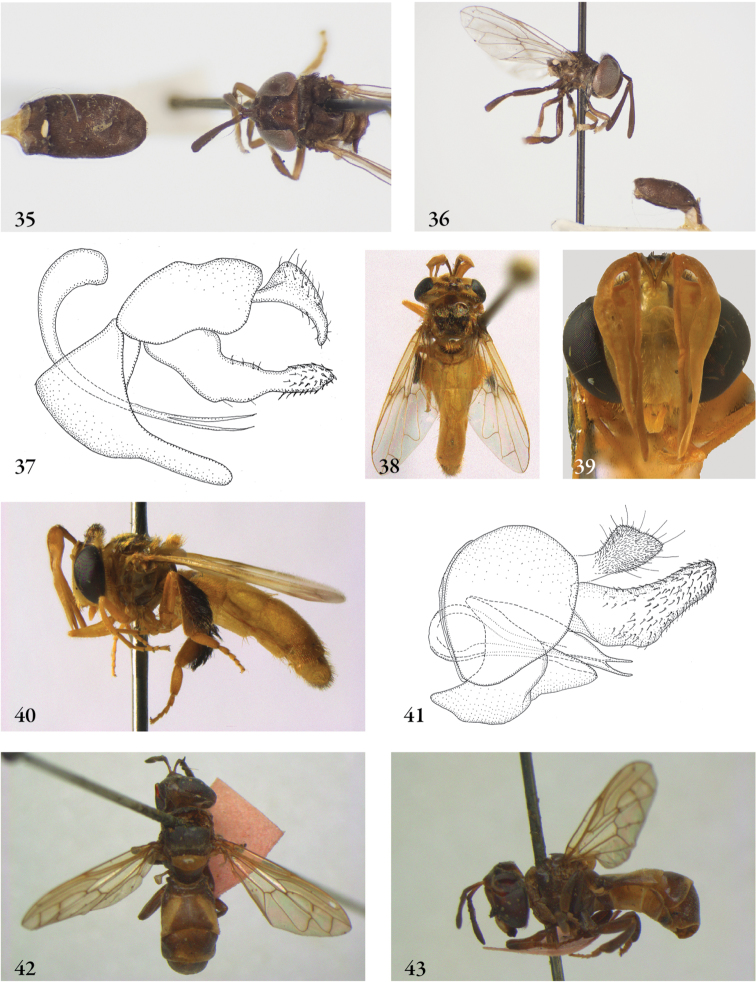
**35–37** *Bardistopus papuanum* male (holotype): **35** habitus dorsal **36** habitus lateral **37** genitalia lateral **38–41** *Carreramyia megacephalus* male (Costa Rica, coll. M. Hauser): **38** habitus dorsal **39** head frontal **40** habitus lateral **41** genitalia lateral (Costa Rica, coll. RMNH) **42–43** *Ceratophya notata* male (holotype): **42** habitus dorsal **43** habitus lateral.

**Figures 44–52.**
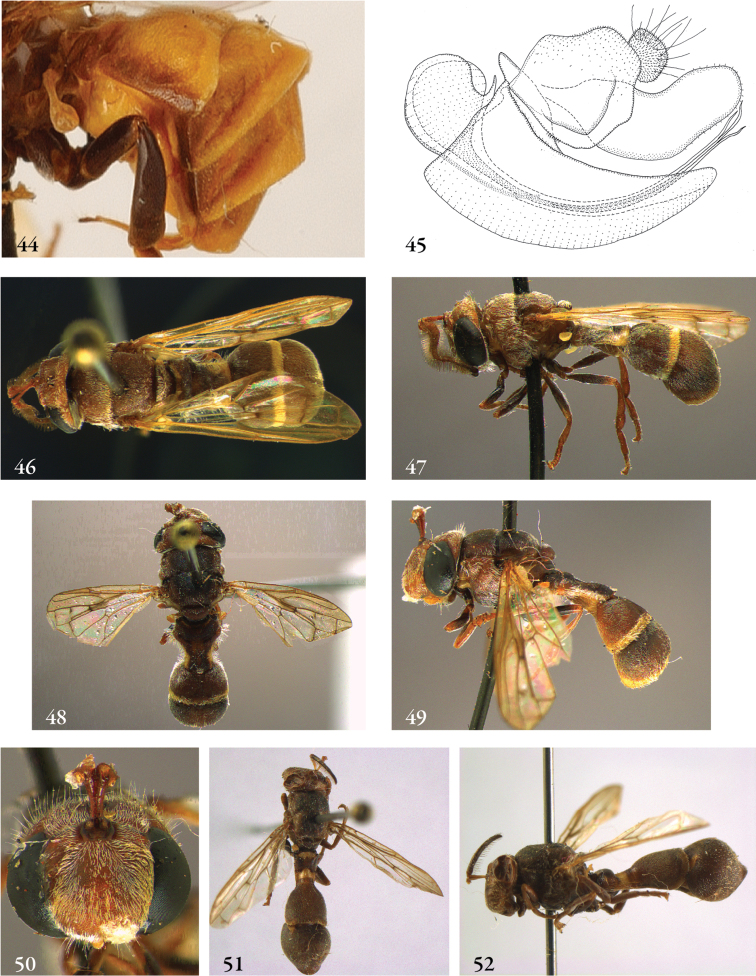
**44–45** *Ceratophya*: **44** *Ceratophya panamensis* female (paratype), abdomen lateral **45** *Ceratophya notata* male (holotype), genitalia **46–52** *Ceratrichomyia* males (holotypes): **46** *Ceratophya behara*, habitus dorsal **47** *Ceratophya behara*, habitus lateral **48** *Ceratophya bullabucca*, habitus dorsal **49** *Ceratophya bullabucca*, habitus lateral **50** *Ceratophya bullabucca*, head frontal **51** *Ceratophya angolensis*, habitus dorsal **52** *Ceratophya angolensis*, habitus lateral.

**Figures 53–60.**
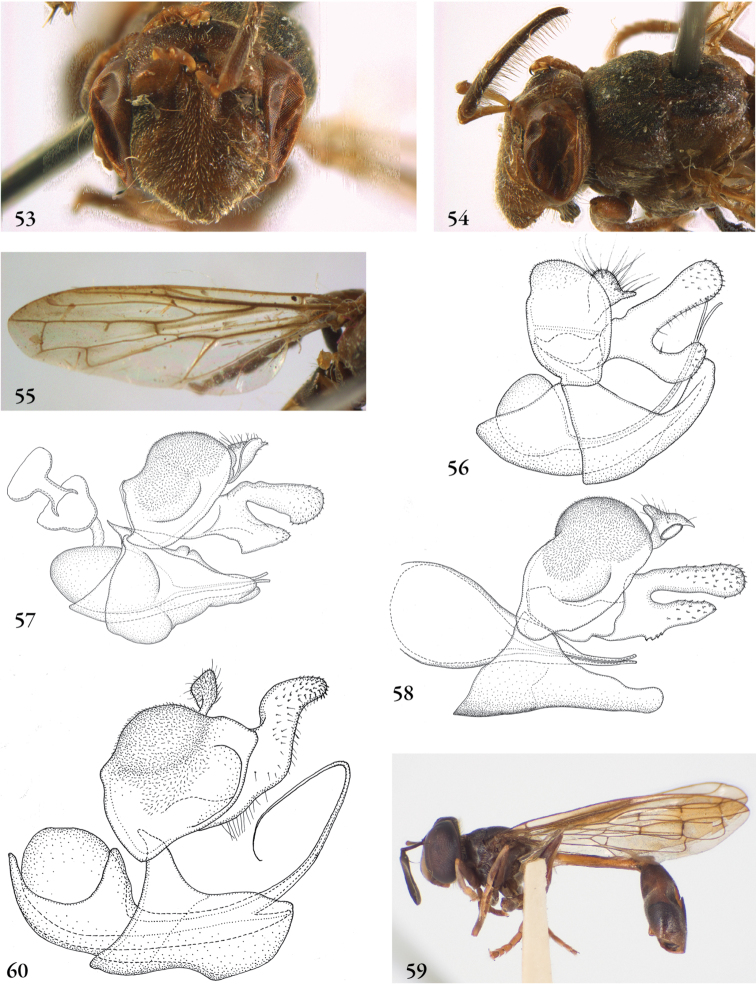
**53–55** *Ceratrichomyia angolensis* male (holotype): **53** head frontal **54** head lateral **55** wing. **56–58** *Ceratrichomyia* males (holotypes): **56** *Ceratrichomyia angolensis* **57** *Ceratrichomyia behara* **58** *Ceratrichomyia bullabucca* **59–60** *Ceriomicrodon petiolatus* male (holotype): **59** habitus lateral **60** genitalia lateral.

**Figures 61–67.**
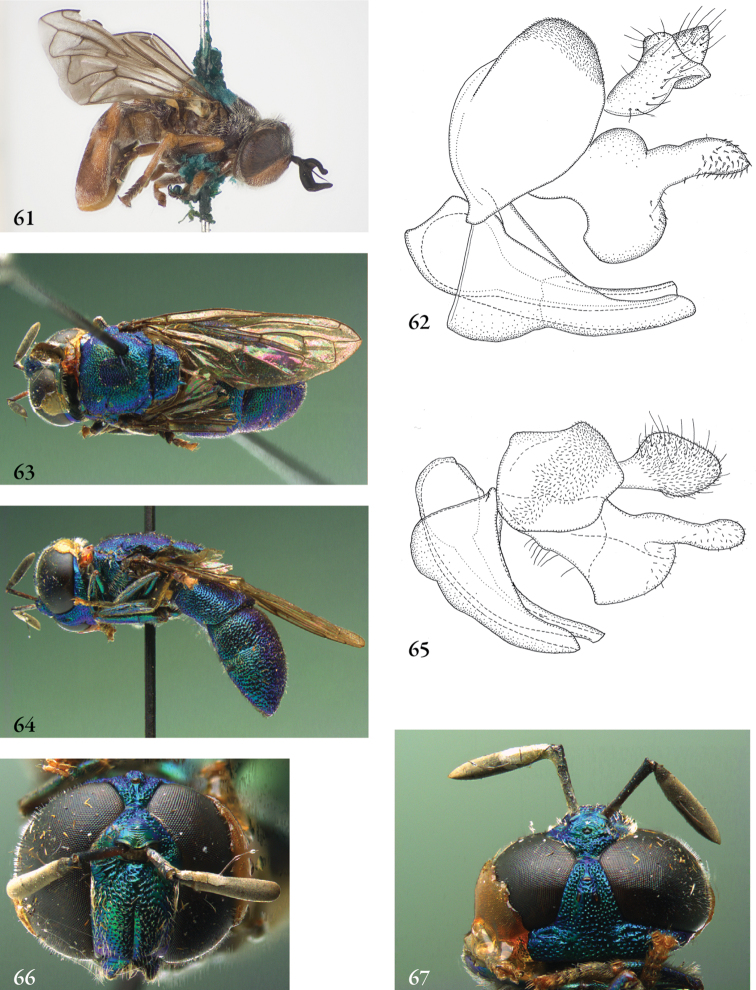
**61–62** *Cervicorniphora alcicornis* male (Australia, coll. USNM): **61** habitus lateral **62** genitalia lateral **63–65** *Chrysidimyia chrysidimima* male (holotype): **63** habitus dorsal **64** habitus lateral **65** genitalia lateral **66–67** *Chrysidimyia chrysidimima* male (holotype): **66** head frontal **67** head lateral.

**Figures 68–75.**
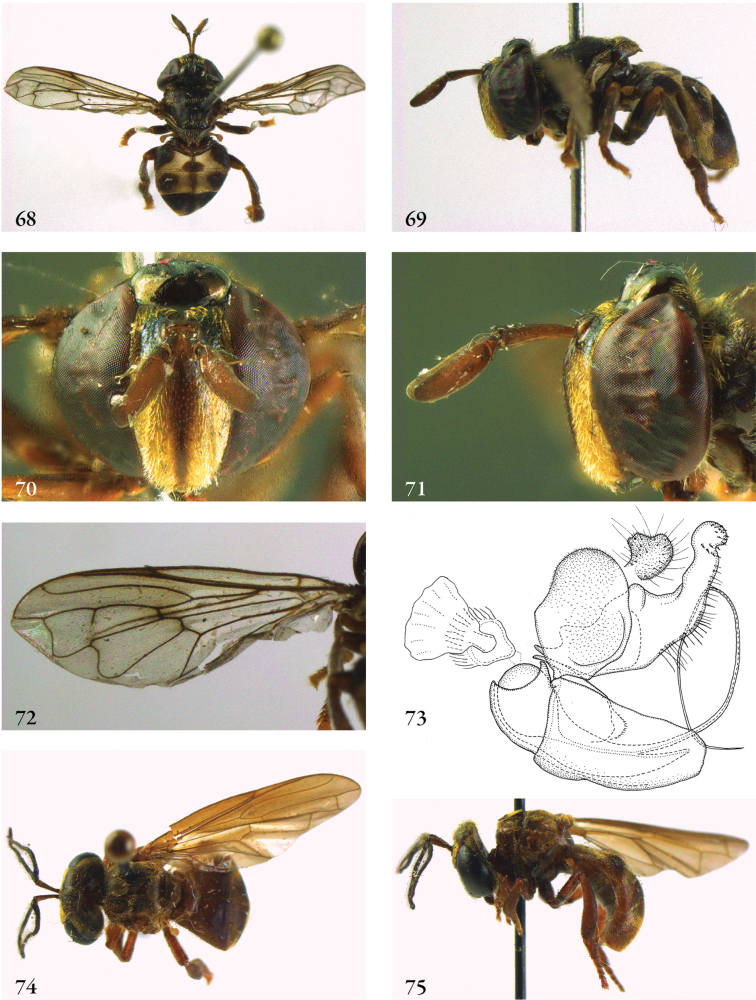
**68–73** *Domodon zodiacus* male (holotype): **68** habitus dorsal **69** habitus lateral **70** head frontal **71** head lateral **72** wing **73** genitalia lateral **74–75** *Furcantenna nepalensis* male (holotype): **74** habitus dorsal **75** habitus lateral.

**Figures 76–84.**
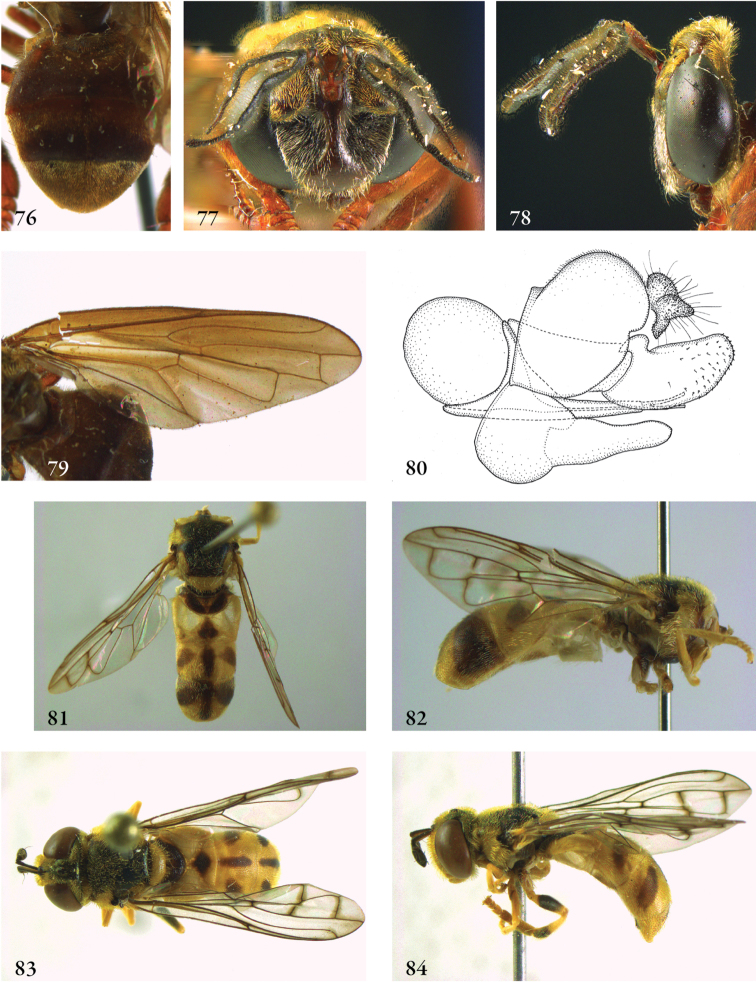
**76–80** *Furcantenna nepalensis* male (holotype): **76** abdomen dorsal **77** head frontal **78** head lateral **79** wing **80** genitalia lateral **81–82** *Heliodon doris* male (holotype): **81** habitus dorsal **82** habitus lateral **83–84** *Heliodon doris* female (paratype): **83** habitus dorsal **84** habitus lateral.

**Figures 85–93. F13:**
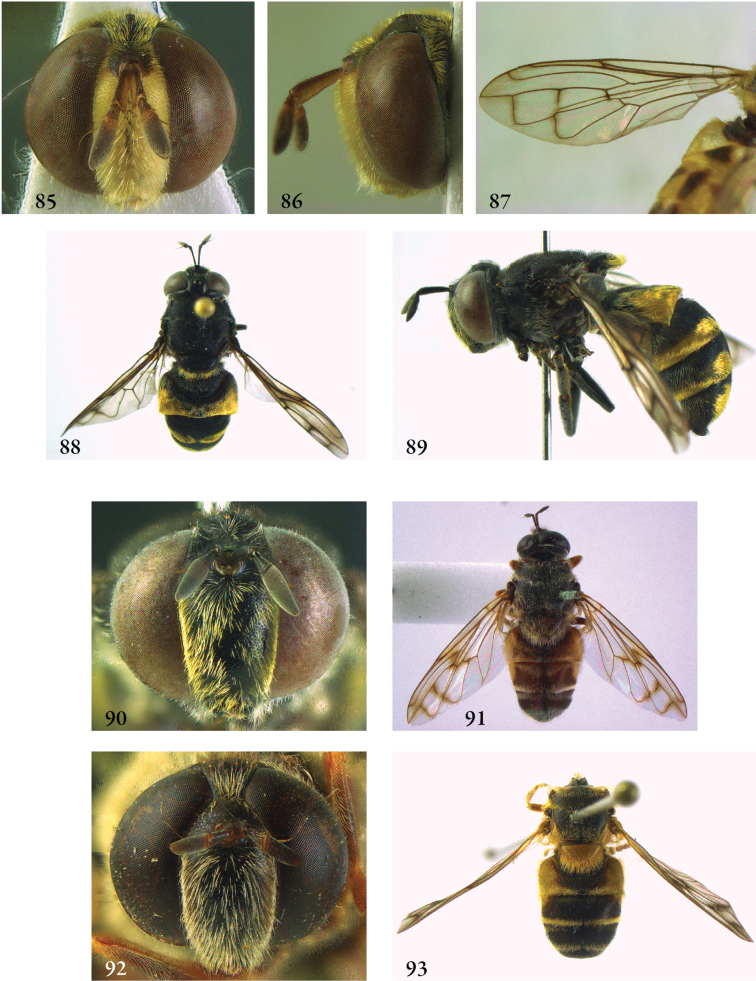
**85–87** *Heliodon doris* male (holotype): **85** head frontal **86** head lateral **87** wing **88–90** *Heliodon elisabethanna* female (holotype): **88** habitus dorsal **89** habitus lateral **90** head frontal **91–92** *Heliodon tiber* male (holotype): **91** habitus dorsal **92** head frontal **93** *Heliodon tiber* female (paratype), habitus dorsal.

**Figures 94–100.**
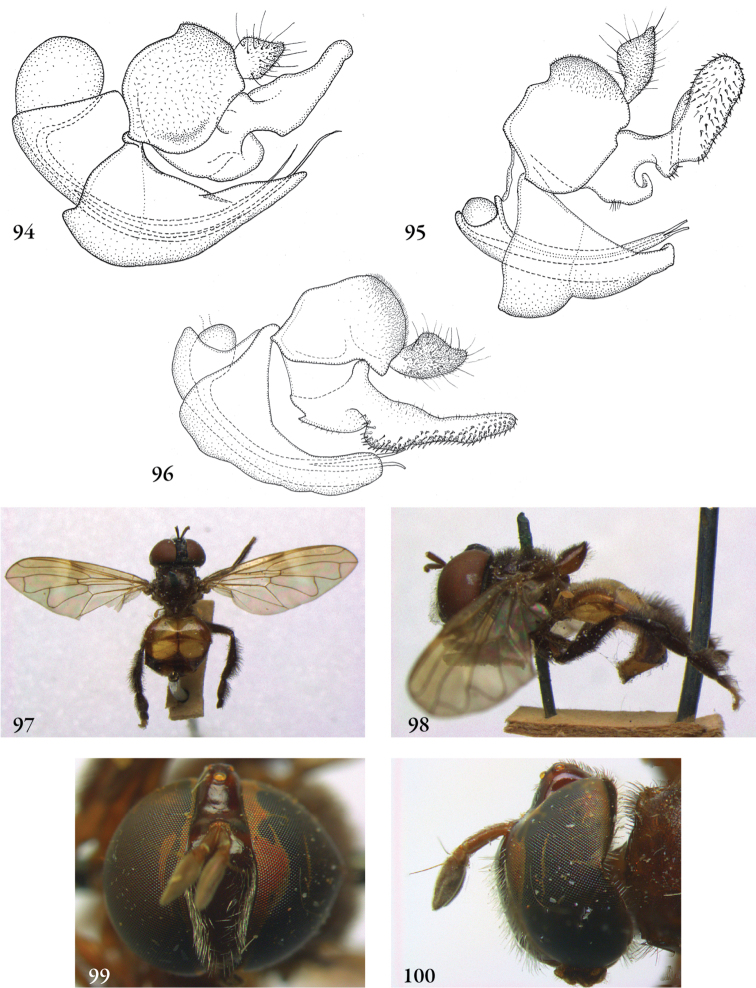
**94–96** *Heliodon*, male genitalia: **94** *Heliodon doris* (holotype) **95** *Heliodon tiber* (holotype) **96** *Heliodon gloriosus* (holotype of *Microdon aurivesta* Hull, jun. syn.) **97–98** *Hypselosyrphus amazonicus* female (holotype *Microdon scutellaris* Shannon): **97** habitus dorsal **98** habitus lateral **99–100** *Hypselosyrphus trigonus* male (holotype): **99** head frontal **100** head lateral.

**Figures 101–108.**
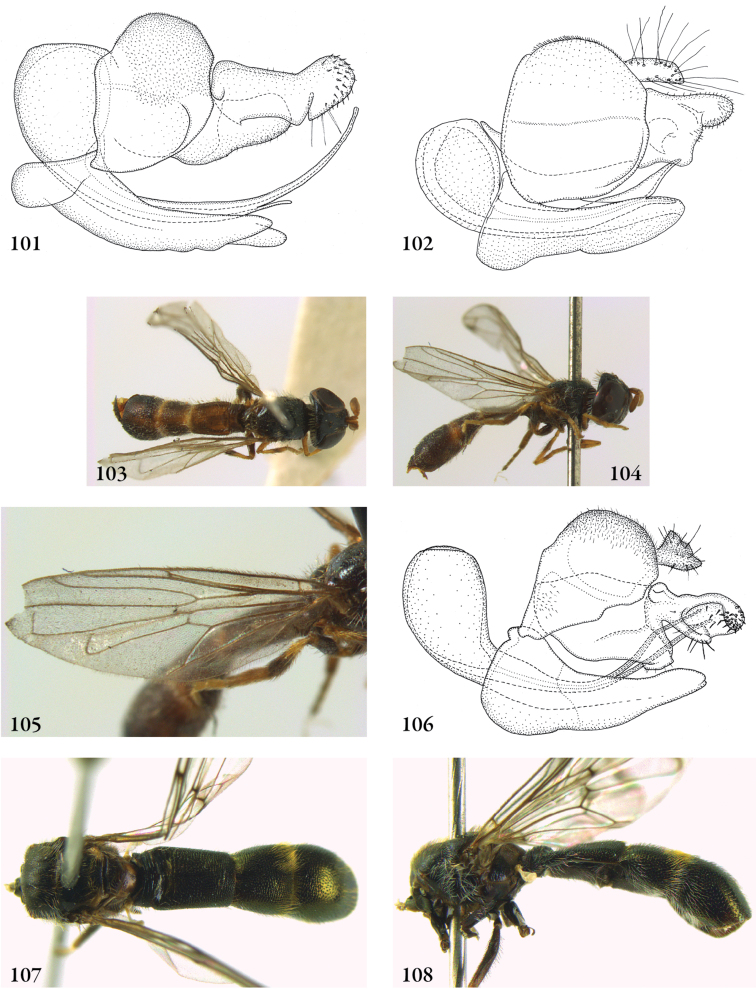
**101–102** *Hypselosyrphus*, male genitalia: **101** *Heliodon amazonicus* (Peru, coll. RMNH) (cercus missing) **102** *Heliodon analis* (holotype) **103–106** *Indascia gracilis* male (holotype): **103** habitus dorsal **104** habitus lateral **105** wing **106** genitalia **107–108** *Indascia gigantica* male (holotype): **107** habitus dorsal **108** habitus lateral.

**Figures 109–118.**
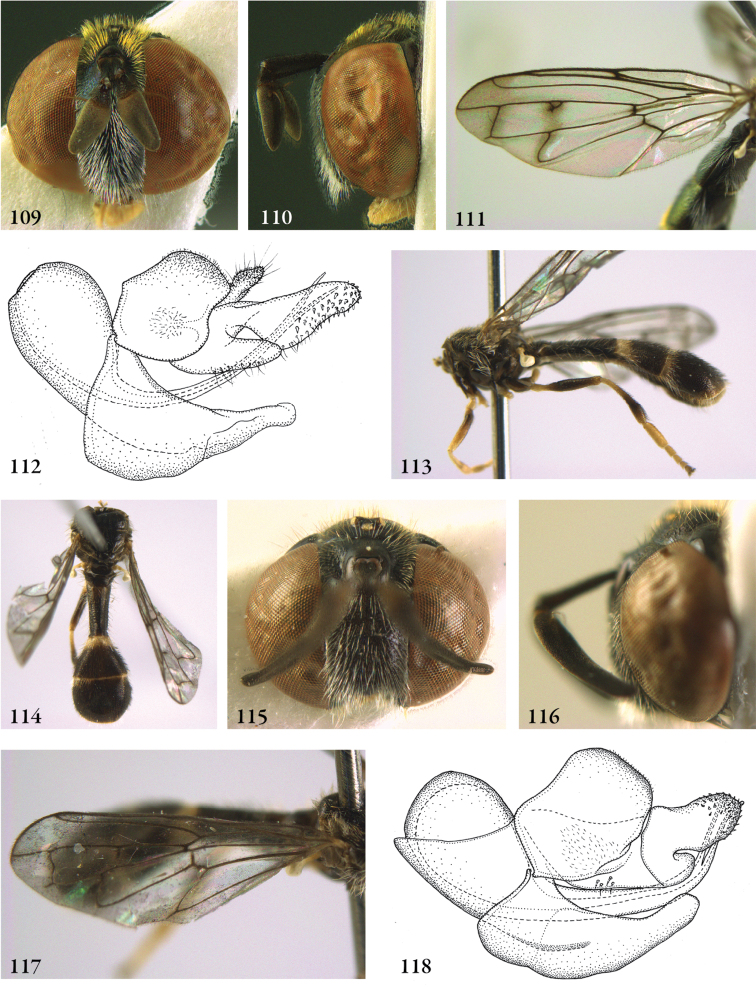
**109–112** *Indascia gigantica* male (holotype): **109** head frontal **110** head lateral **111** wing **112** genitalia lateral **113–118** *Indascia spathulata* male (holotype): **113** habitus lateral **114** dorsal **115** head frontal **116** head lateral **117** wing **118** genitalia lateral.

**Figures 119–127.**
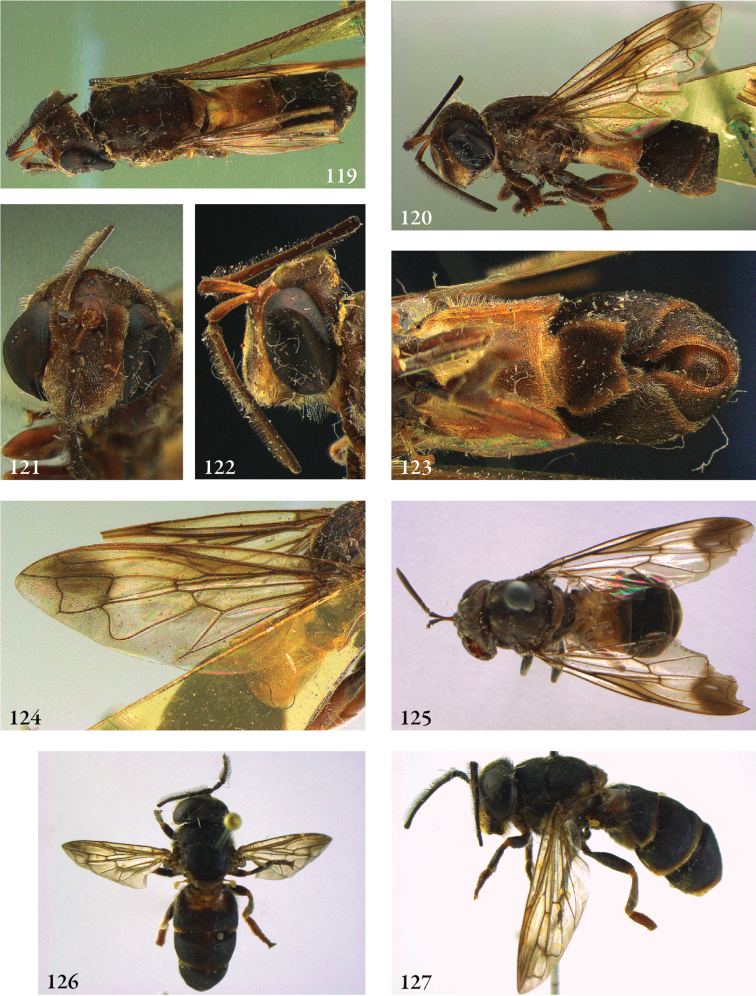
**119–124** *Kryptopyga pendulosa* male (holotype): **119** habitus dorsal **120** habitus lateral **121** head frontal **122** head lateral **123** abdomen ventral **124** wing **125** *Kryptopyga pendulosa* female (Indonesia, Bangka, coll. RMNH) **126–127** *Kryptopyga sulawesiana* male (holotype): **126** habitus dorsal **127** habitus lateral.

**Figures 128–135.**
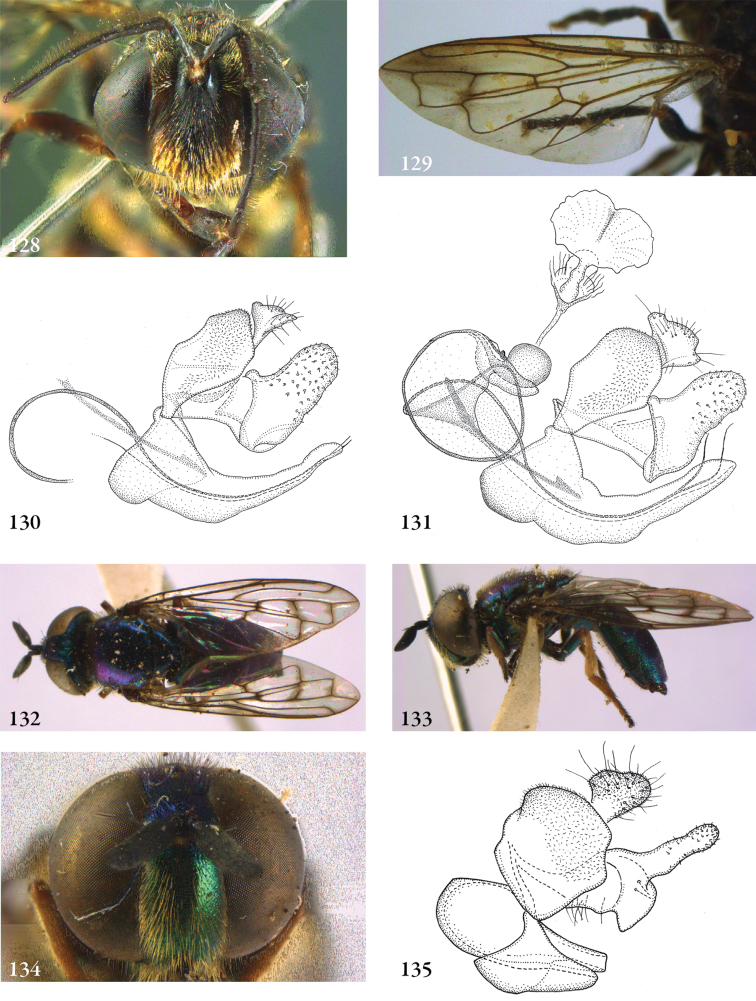
**Figures 128–129.** *Kryptopyga sulawesiana* male (holotype): **128** head frontal **129** wing **130–131** *Kryptopyga*, male genitalia **130** *Kryptopyga pendulosa* (holotype) **131** *Kryptopyga sulawesiana* (holotype) **132–134** *Laetodon violens* male (Jamaica, coll. RMNH): **132** habitus dorsal **133** habitus lateral **134** head frontal **135** *Laetodon laetus* male (USA, Georgia, coll. RMNH), genitalia lateral.

**Figures 136–144.**
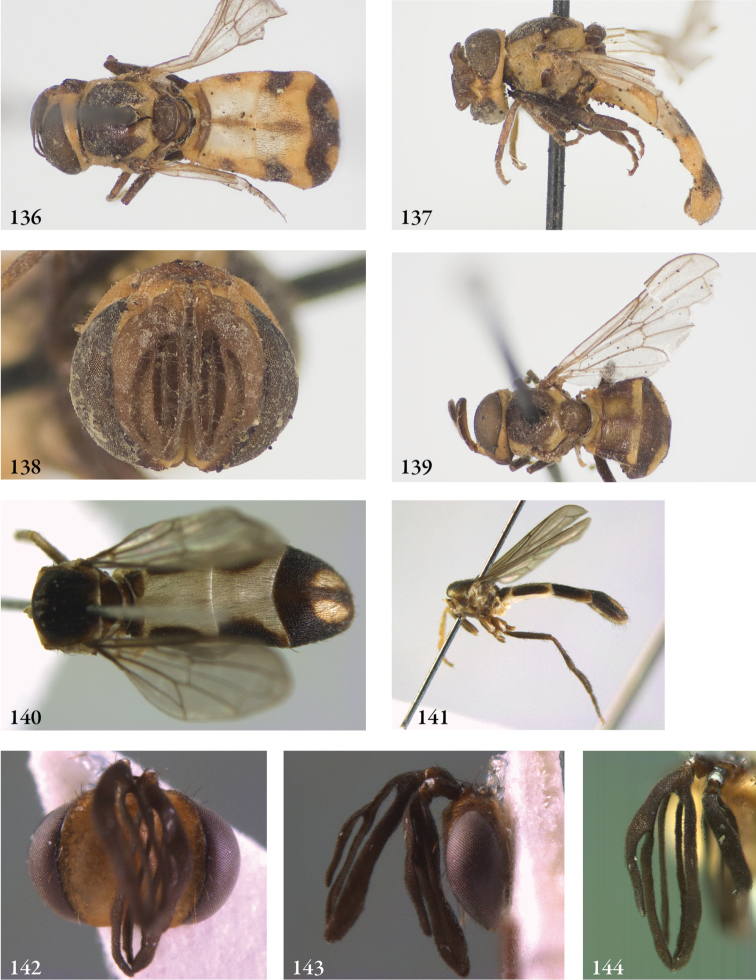
**136–138** *Masarygus planifrons* male (syntype): **136** habitus dorsal **137** habitus lateral **138** head frontal **139** *Masarygus planifrons* female (syntype), habitus dorsal **140–143** *Masarygus palmipalpus* male (holotype): **140** habitus dorsal **141** habitus lateral **142** head frontal **143** head lateral **144** antenna.

**Figures 145–153.**
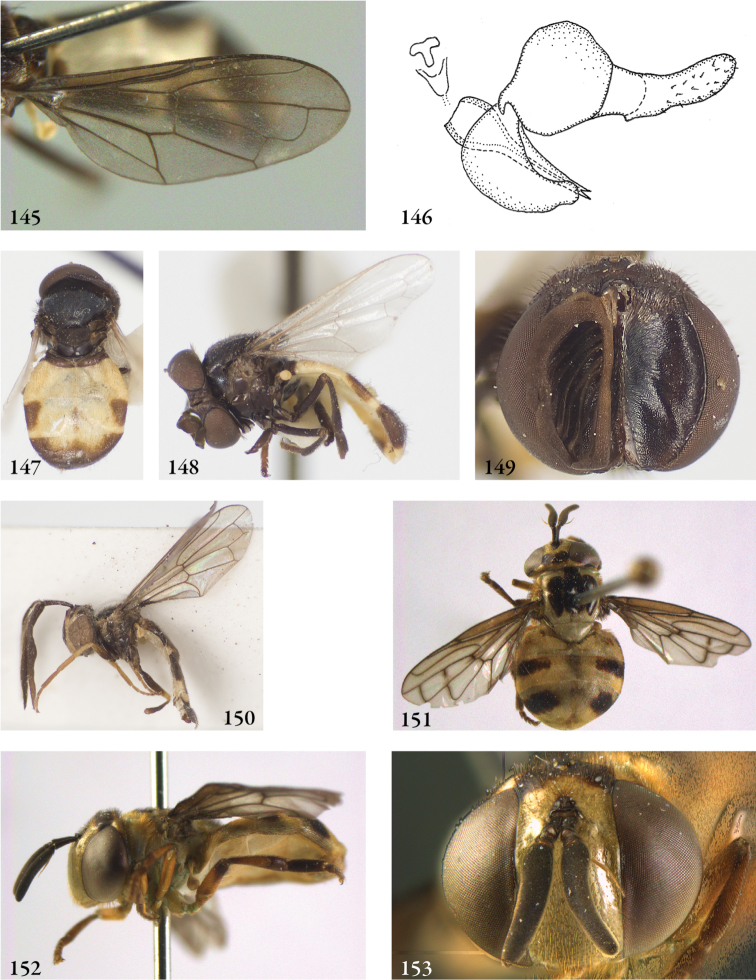
**145–146** *Masarygus palmipalpus* male (holotype): **145** wing **146** genitalia lateral **147–149** *Masarygus* spec. 1 male (Brazil, coll. USNM): **147** habitus dorsal **148** habitus lateral **149** head frontal **150** *Masarygus* spec. 2 male (Brazil, coll. USNM), habitus lateral **151–153** *Menidon falcatus* male (Costa Rica, coll. ZMAN): **151** habitus dorsal **152** habitus lateral **153** head frontal.

**Figures 154–162.**
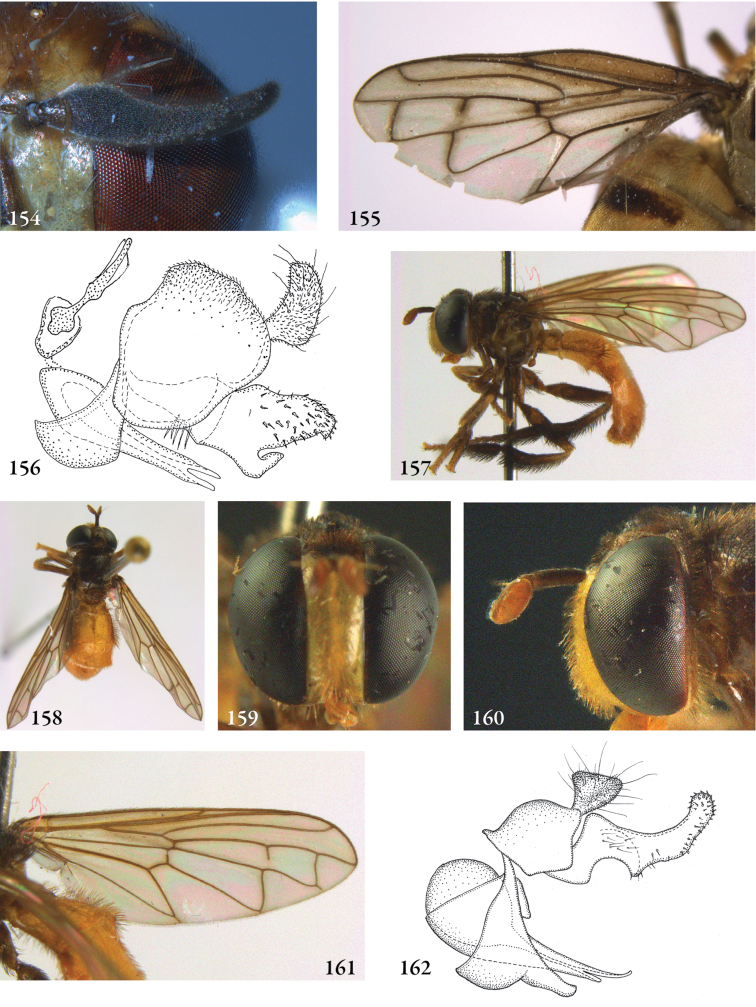
**154–155** *Menidon falcatus* male (Costa Rica, coll. ZMAN): **154** antenna **155** wing **156** *Menidon falcatus* male (Costa Rica, coll M. Hauser), genitalia lateral **157–162** *Mermerizon inbio* male (holotype): **157** habitus lateral **158** habitus dorsal **159** head frontal **160** head lateral **161** wing **162** genitalia lateral.

**Figures 163–170.**
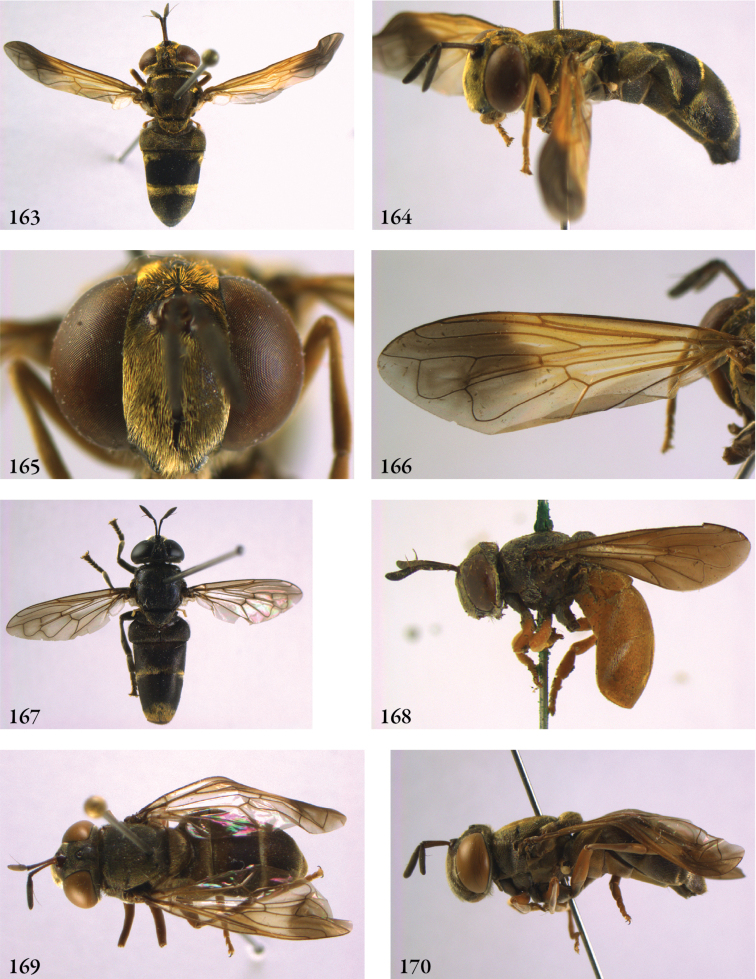
**163–166** *Metadon achterbergi* female (holotype) **163** habitus dorsal **164** habitus lateral **165** head frontal **166** wing **167–168** *Metadon*, habitus: **167** *Metadon bifasciatus* (Japan, coll. RMNH) **168** *Metadon inermis* (holotype) **169–170** *Metadon wulpii* female (Borneo, coll. RMNH): **169** habitus dorsal **170** habitus lateral.

**Figure:**
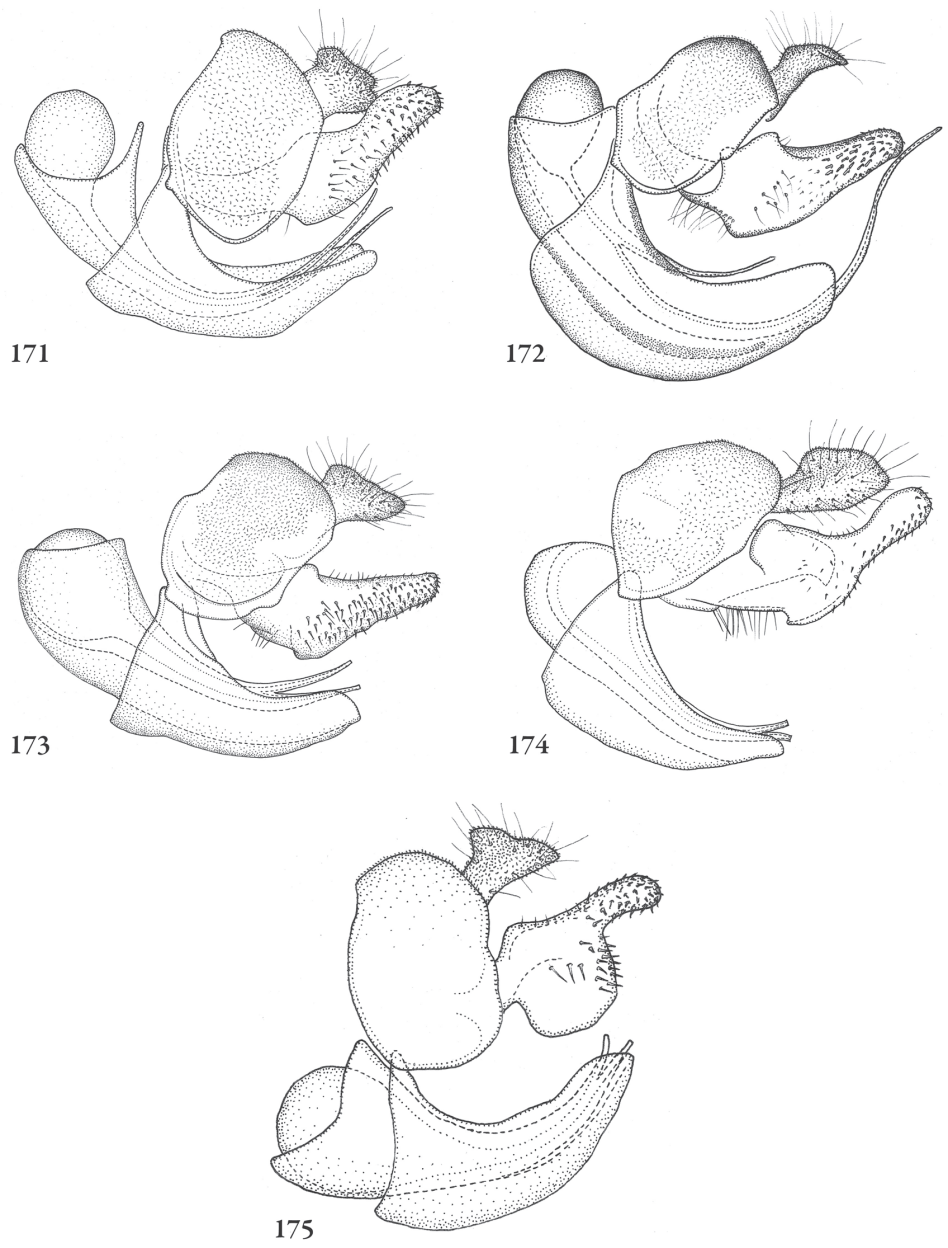
**Figures 171–175.** *Metadon*, male genitalia lateral: **171** *Metadon bicoloratus* (holotype) **172** *Metadon bifasciatus* (China, coll. RMNH) **173** *Metadon inermis* (holotype) **174** *Metadon montis* (holotype) **175** *Metadon punctulatus* (holotype).

**Figures 176–180.**
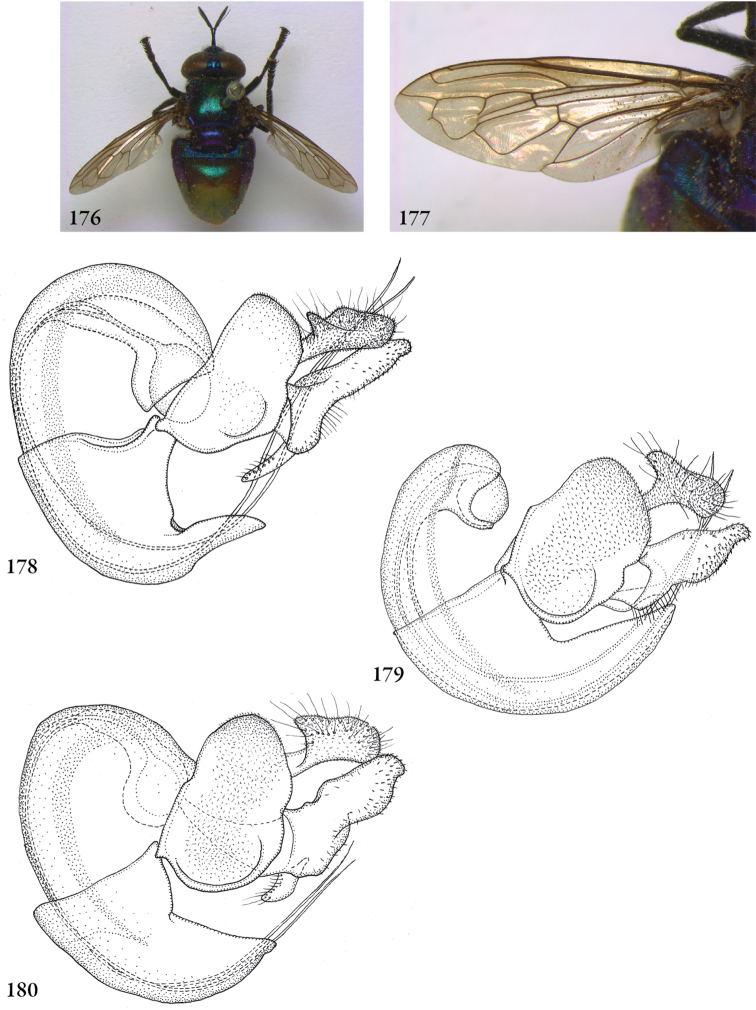
**176–178** *Microdon (Chymophila) instabilis* (holotype): **176** habitus dorsal **177** wing **178** genitalia lateral **179–180** *Microdon (Chymophila)*, male genitalia: **179** *Microdon lativentris* (holotype) **180** *Microdon stramineus* (Surinam, coll. RMNH).

**Figures 181–186.**
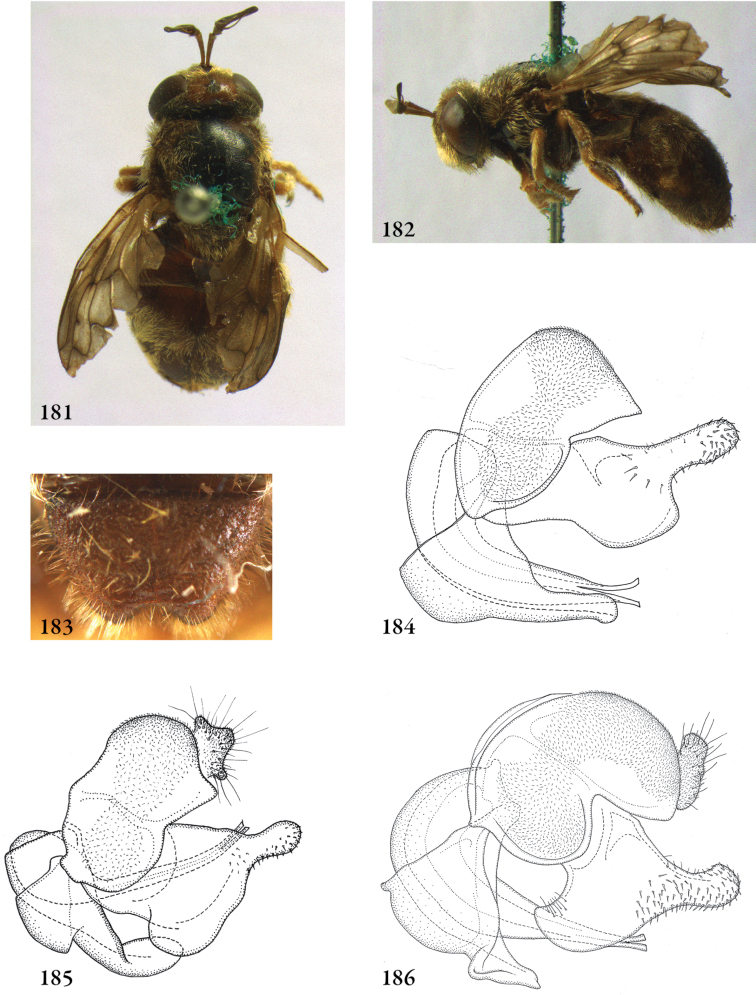
**181–182** *Microdon (Dimeraspis) marmoratus* male (holotype): **181** habitus dorsal **182** habitus lateral **183** *Microdon (Dimeraspis) globosus* male (USA, Pennsylvania, coll. RMNH), scutellum **184–186** *Microdon (Dimeraspis)*, male genitalia: **184** *Microdon globosus* (USA, Pennsylvania, coll. RMNH) **185** *Microdon abditus* (paratype, USA, Queens, coll. RMNH) **186** *Microdon fuscipennis* (USA, N-Carolina, coll. RMNH).

**Figures 187–195.**
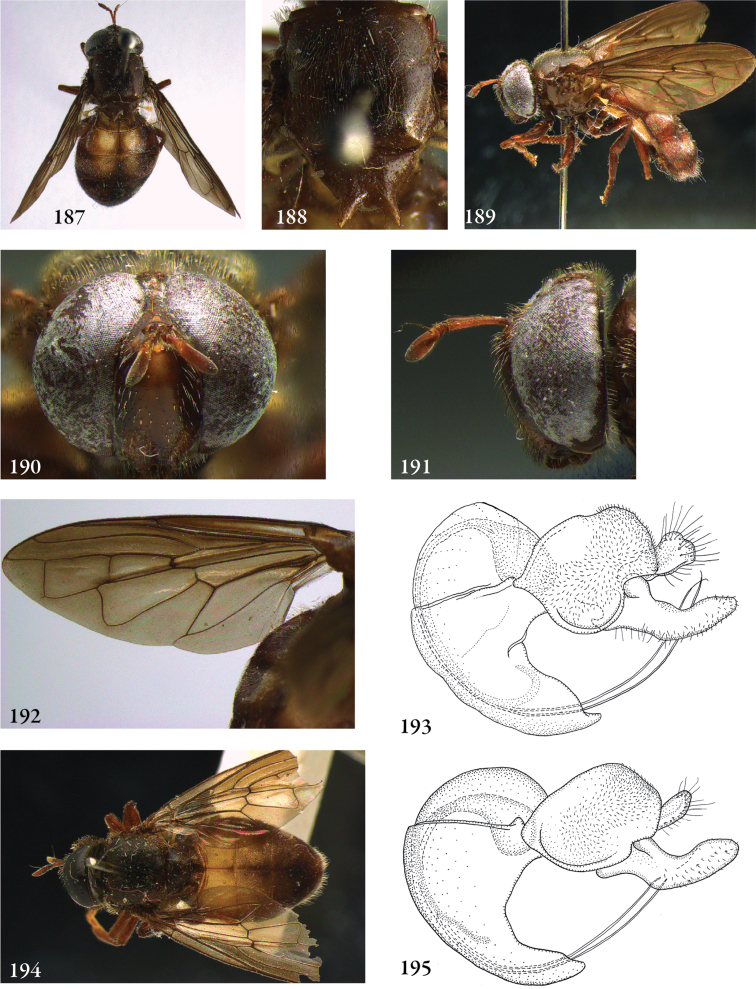
**187–190** *Microdon (Megodon) stuckenbergi* male (holotype): **187** habitus dorsal **188** thorax dorsal **189** habitus lateral **190** head frontal **191–193** *Microdon (Megodon) stuckenbergi* male (holotype): **191** head lateral **192** wing **193** genitalia **194–195** *Microdon (Megodon) planitarsus* male (holotype): **194** habitus dorsal **195** genitalia.

**Figure 206–212. F28:**
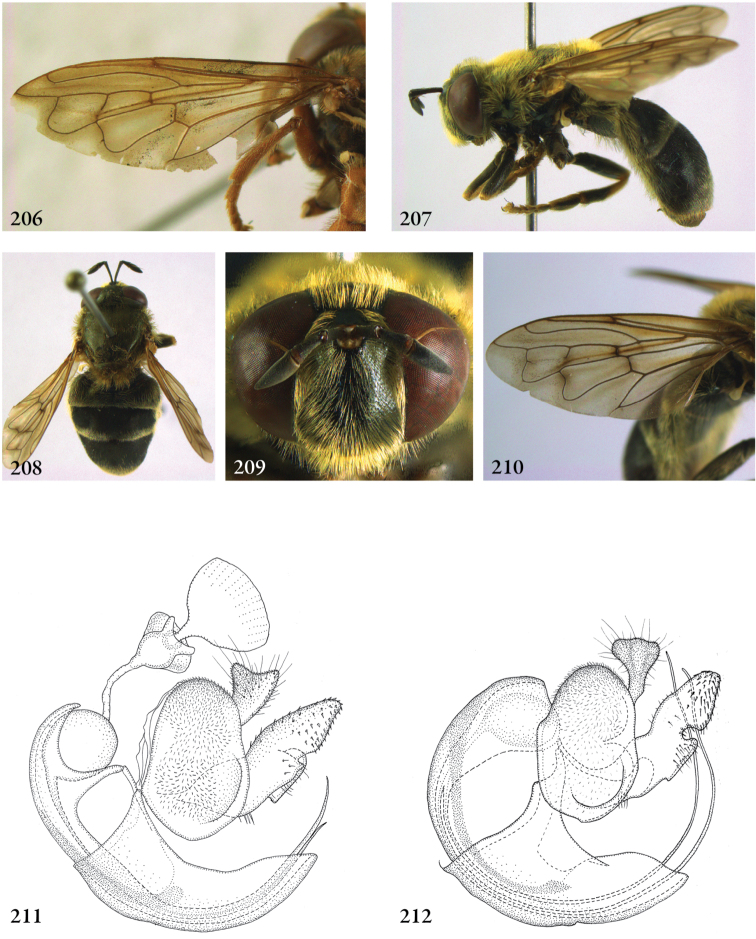
**206** *Microdon (Microdon) mandarinus* male (holotype), wing **207–210** *Microdon (Microdon) yunnanensis* male (holotype) **207** habitus lateral **208** habitus dorsal **209** head frontal **210** wing **211–212** *Microdon (Microdon)*, male genitalia: **211** *Microdon mutabilis* (Belarus, coll. M. Reemer) **212** *Microdon hauseri* (holotype).

**Figures 213–220.**
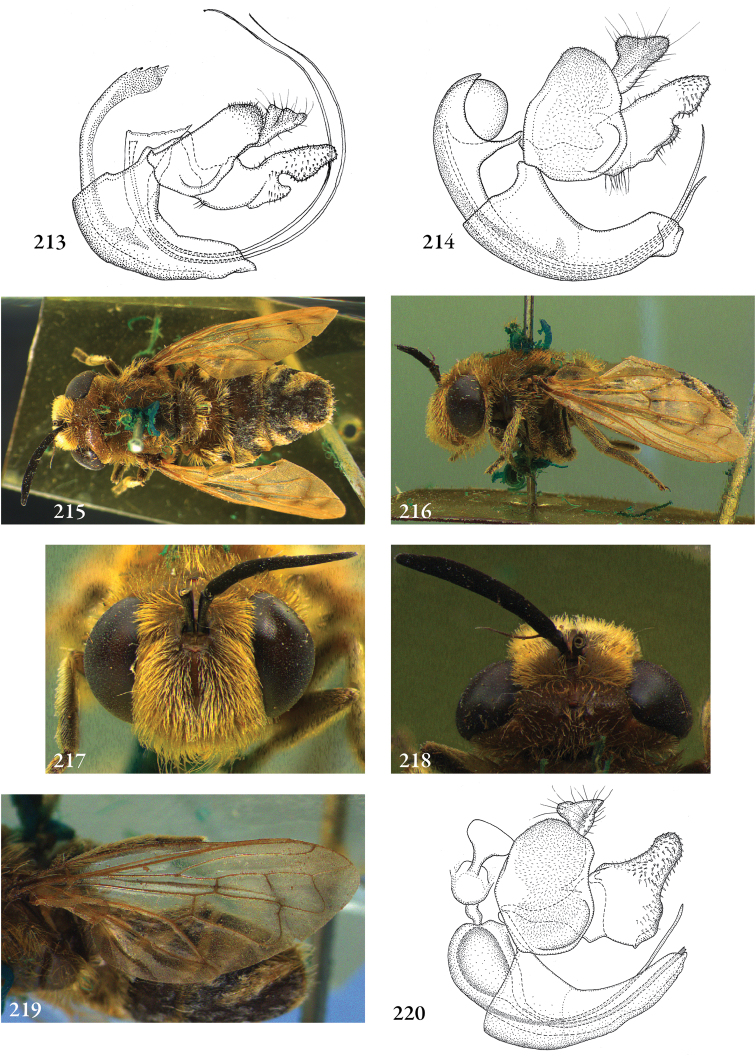
**213–214** *Microdon (Microdon)*, male genitalia: **213** *Microdon mandarinus* (holotype) **214** *Microdon yunnanensis* (holotype) **215–220** *Microdon (Myiacerapis) villosus* male (holotype): **215** habitus dorsal **216** habitus lateral **217** head frontal **218** head dorsal **219** wing **220** genitalia lateral.

**Figures 221–228.**
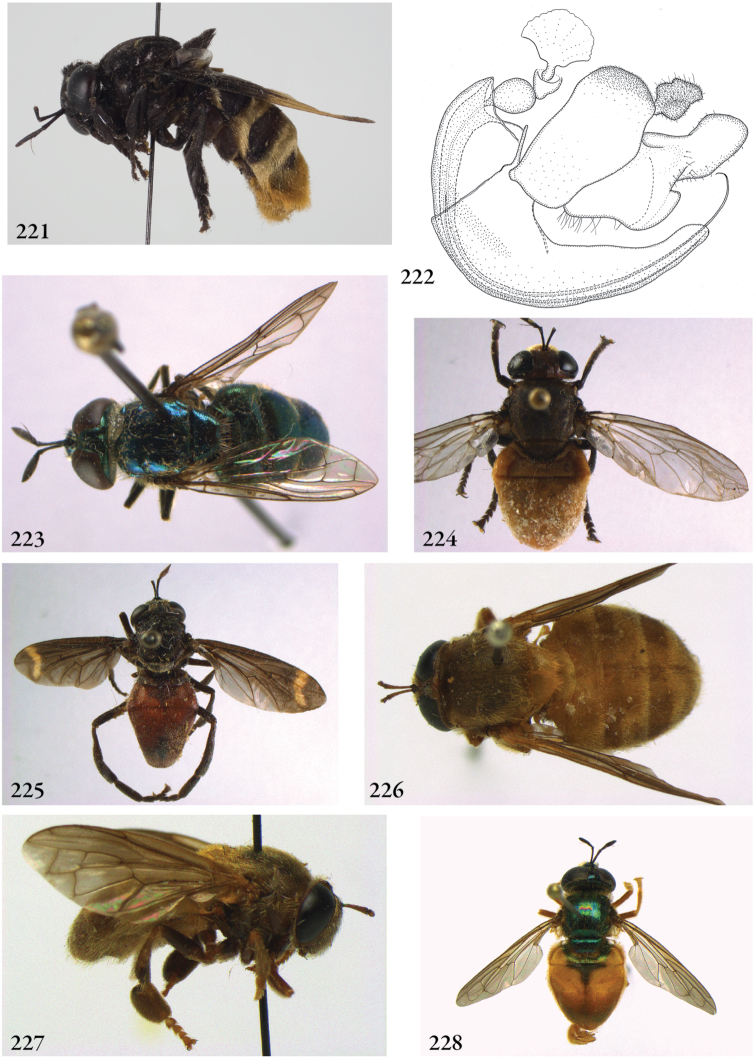
**221–222** *Microdon (Syrphipogon) fucatissimus* male (holotype): **221** habitus lateral **222** genitalia lateral **223–228** *Microdon* s.l. males, habitus: **223** *craigheadi*-group: *Microdon craigheadi* (USA, S-Carolina, coll. BMNH) **224** *erythros*-group: *Microdon luteiventris* (Kenya, coll. RMNH) **225** *mirabilis*-group: *Microdon bertonii* (Brazil, coll. RMNH) **226** *tarsalis*-group: *Microdon tarsalis* (holotype) **227** *Microdon tarsalis* **228** *virgo*-group: *Microdon rufiventris* (Surinam, coll. RMNH).

**Figures 251–259.**
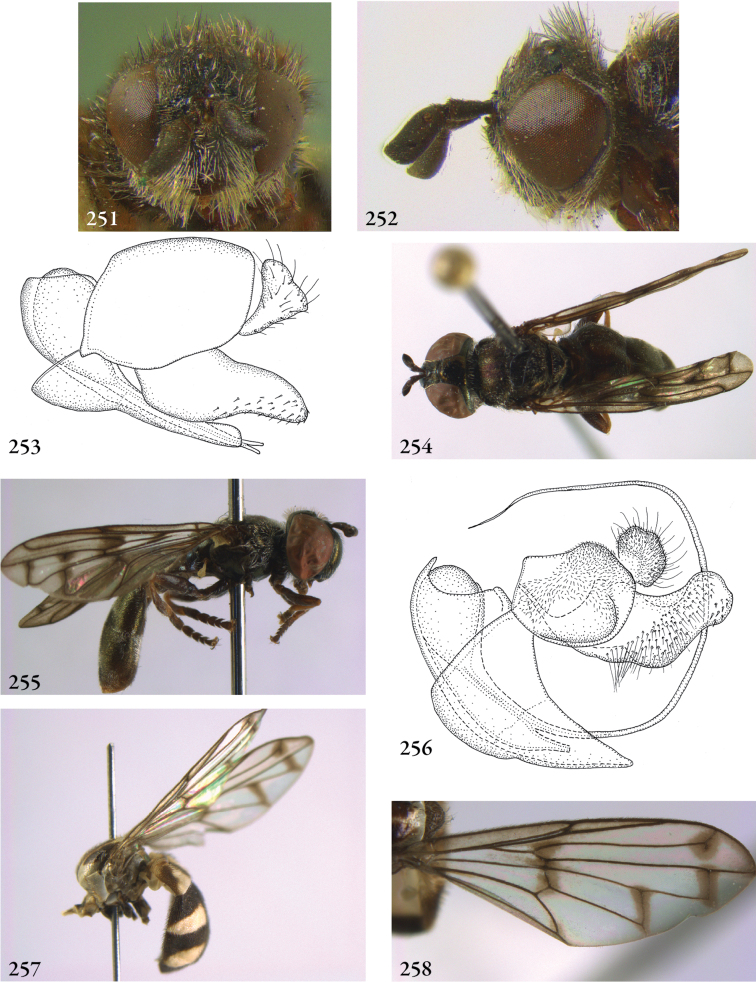
**251–252** *Oligeriops iridomyrmex* female (syntype): **251** head frontal **252** head lateral **253** *Oligeriops dimorphon* male (Australia, coll. USNM), genitalia lateral **254–255** *Omegasyrphus pallipennis* male (USA, California, coll. RMNH) **254** habitus dorsal **255** habitus lateral **256** *Omegasyrphus coarctatus* male (USA, Virginia, coll. RMNH), genitalia lateral. **257–258** *Paragodon paragoides* female (Costa Rica, coll. RMNH): **257** habitus lateral **258** wing.

**Figures 259–268.**
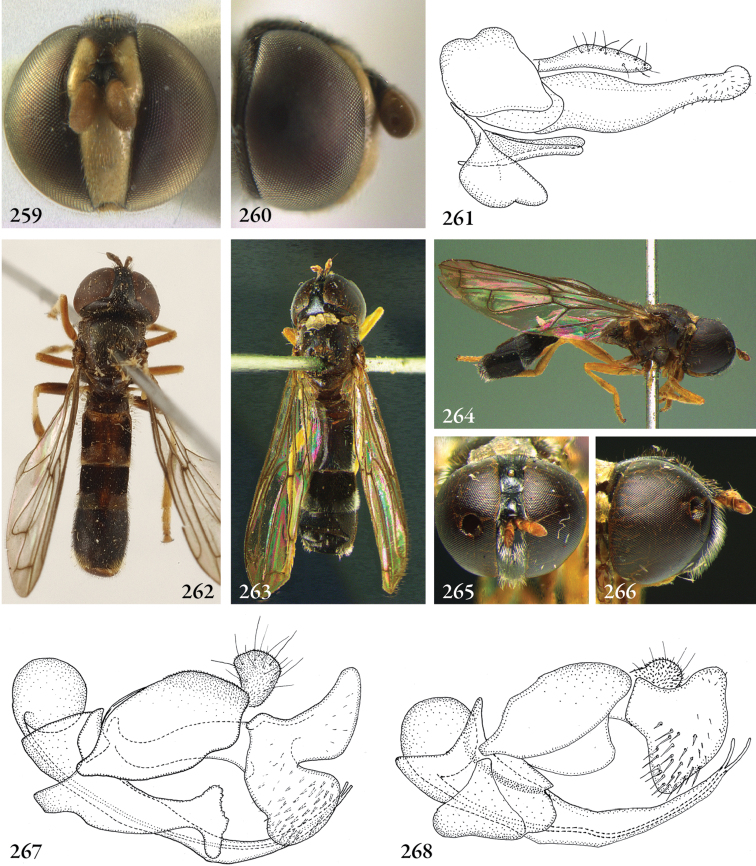
**259–260** *Paragodon paragoides* female (Costa Rica, coll. RMNH) **259** head frontal **260** head lateral **261** *Paragodon paragoides* male (Panama, coll. SEMC), genitalia lateral. **262** *Paramicrodon flukei* male (holotype), habitus dorsal. Photo: American Museum of Natural History **263–266** *Paramicrodon toxopei* male (holotype): **263** habitus dorsal **264** habitus lateral **265** head frontal **266** head lateral **267–268** *Paramicrodon*, male genitalia: **267** *Paramicrodon* cf. *flukei* (Peru, coll. RMNH) **268** *Microdon toxopei* (holotype).

**Figures 269–278.**
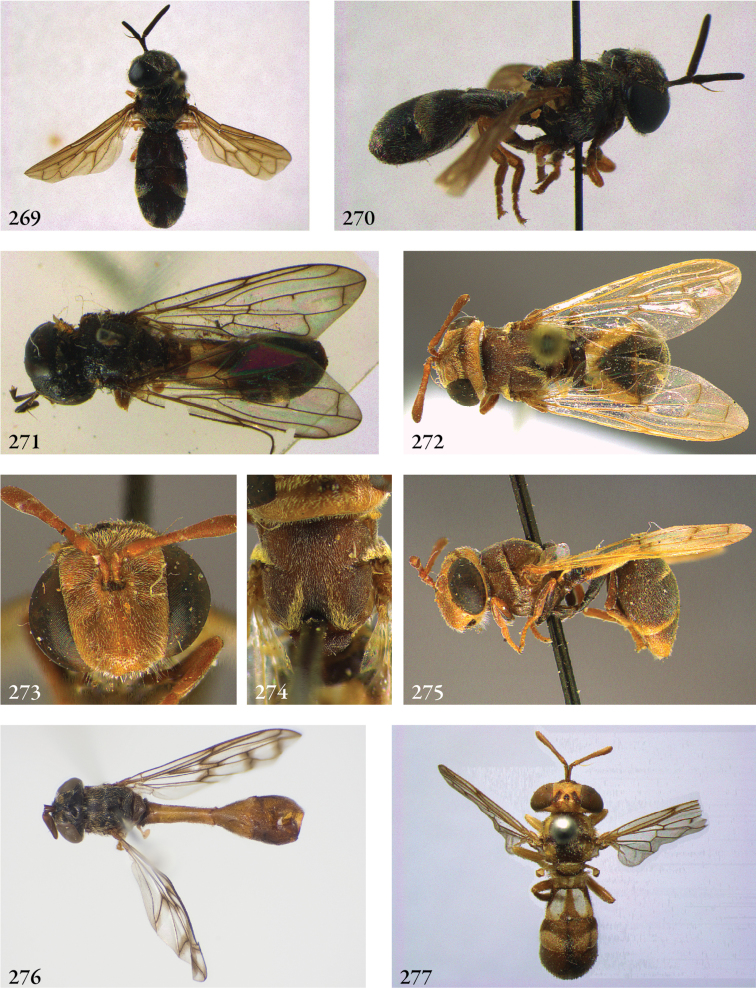
**269–270** *Paramixogaster acantholepidis* male (holotype): **269** habitus dorsal **270** habitus lateral **271** *Paramixogaster luxor* male (holotype), habitus dorsal **272–275** *Paramixogaster piptotus* female (holotype): **272** habitus dorsal **273** head frontal **274** thorax dorsal **275** habitus lateral **276–277** *Paramixogaster*, habitus dorsal: **276** *Paramixogaster omeanus* male (holotype) **277** *Paramixogaster vespiformis* female (syntype).

**Figures 278–284.**
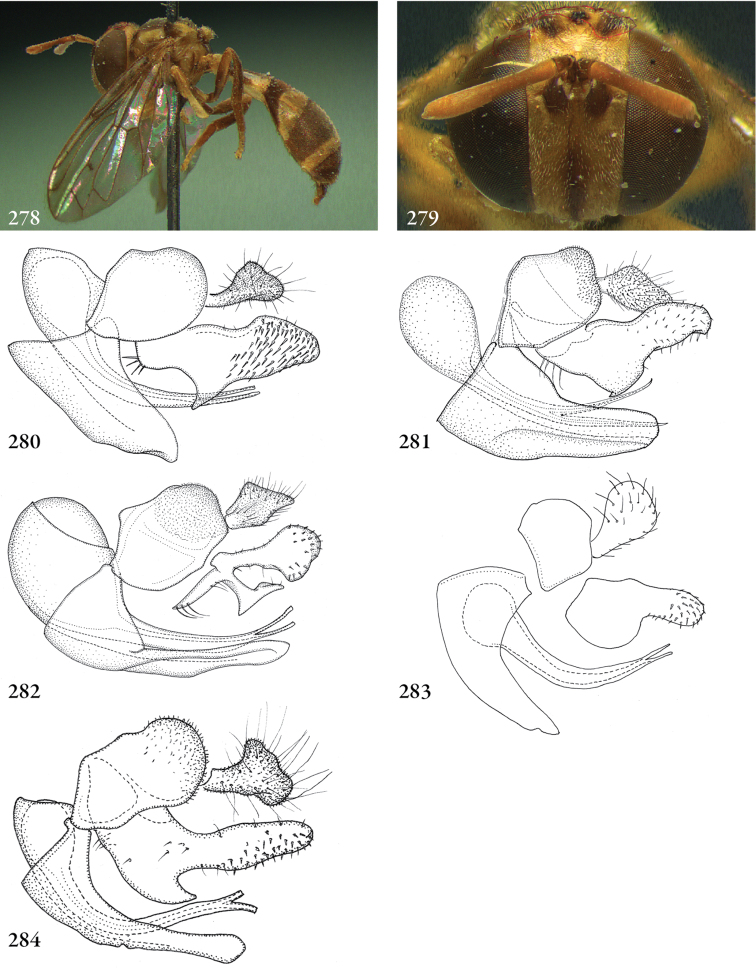
**278–279** *Paramixogaster vespiformis* female (syntype): **278** habitus lateral **279** head frontal **280–284** *Paramixogaster*, male genitalia: **280** *Paramixogaster acantholepidis* (holotype) **281** *Paramixogaster illucens* (holotype) **282** *Paramixogaster luxor* (holotype) **283** *Paramixogaster omeanus* (Australia, coll. USNM) **284** *Paramixogaster* cf. *vespiformis* (Vietnam, coll. RMNH).

**Figures 285–292.**
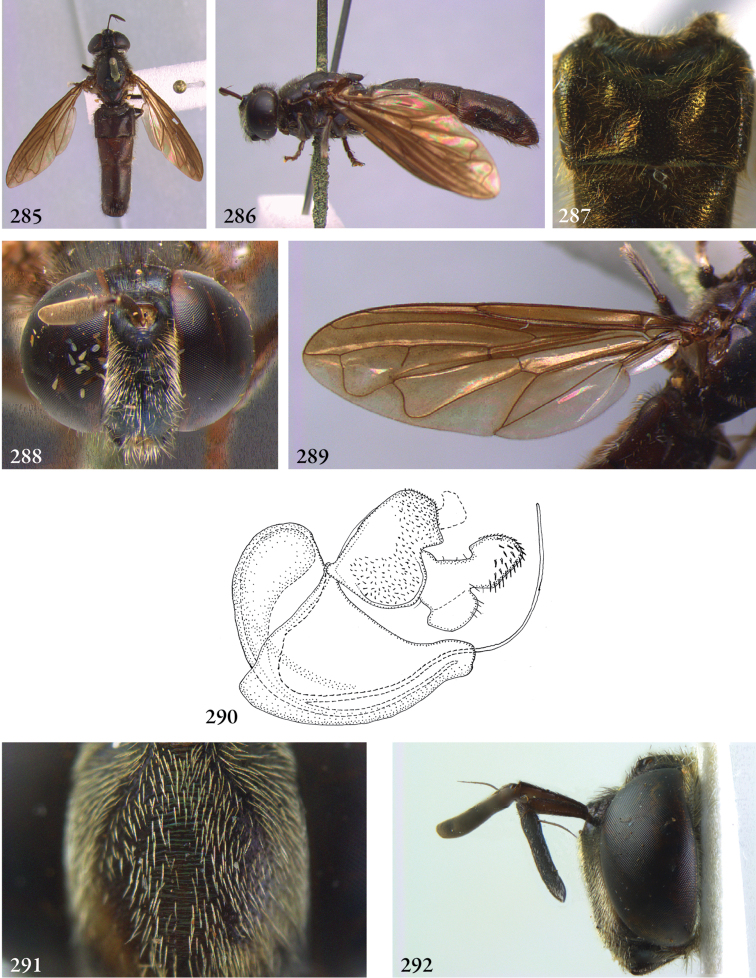
**285–289** *Parocyptamus sonamii* male (syntype, except 290 Thailand, coll. RMNH): **285** habitus dorsal **286** habitus lateral **287** tergite 2 dorsal **288** head frontal **289** wing **290** *Parocyptamus stenogaster* male (holotype), genitalia lateral **291–292** *Peradon bidens* male (Surinam, coll. RMNH): **291** face frontal **292** head lateral.

**Figure 293–298.**
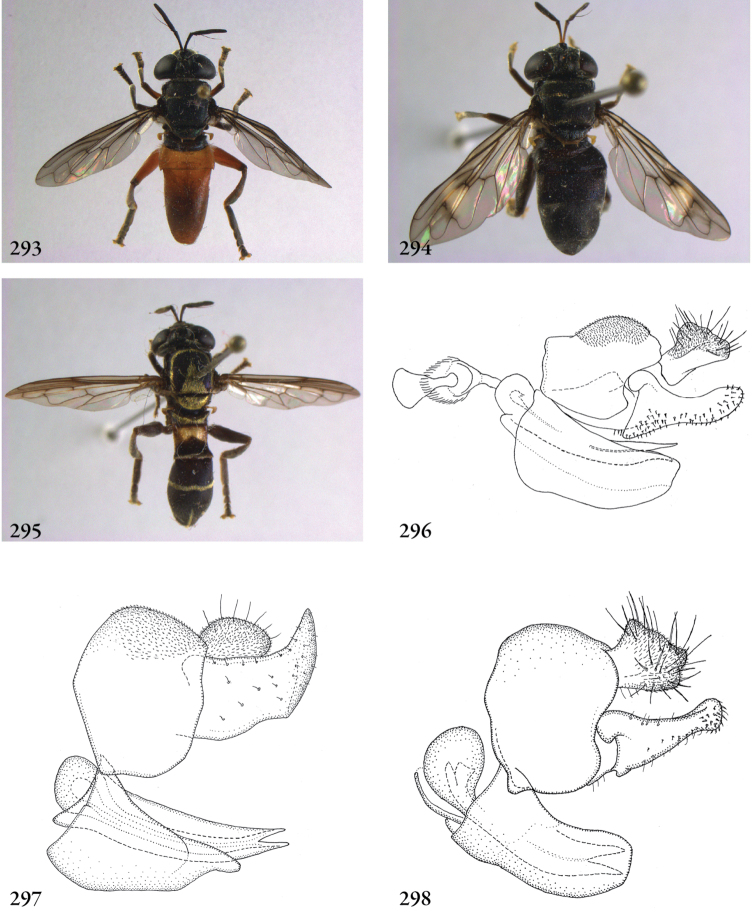
**293** *Peradon bidens* (Surinam, coll. RMNH), habitus dorsal **294** *Peradon flavofascium* female (Surinam, coll. RMNH), habitus dorsal **295** *Peradon trivittatum* male (Surinam, coll. RMNH), habitus dorsal **296–298** *Peradon*, male genitalia lateral: **296** *Peradon bidens* (Surinam, coll. RMNH) **296** *Peradon flavofascium* (holotype) **298** *Peradon trivittatum* (Surinam, coll. RMNH).

**Figures 299–307.**
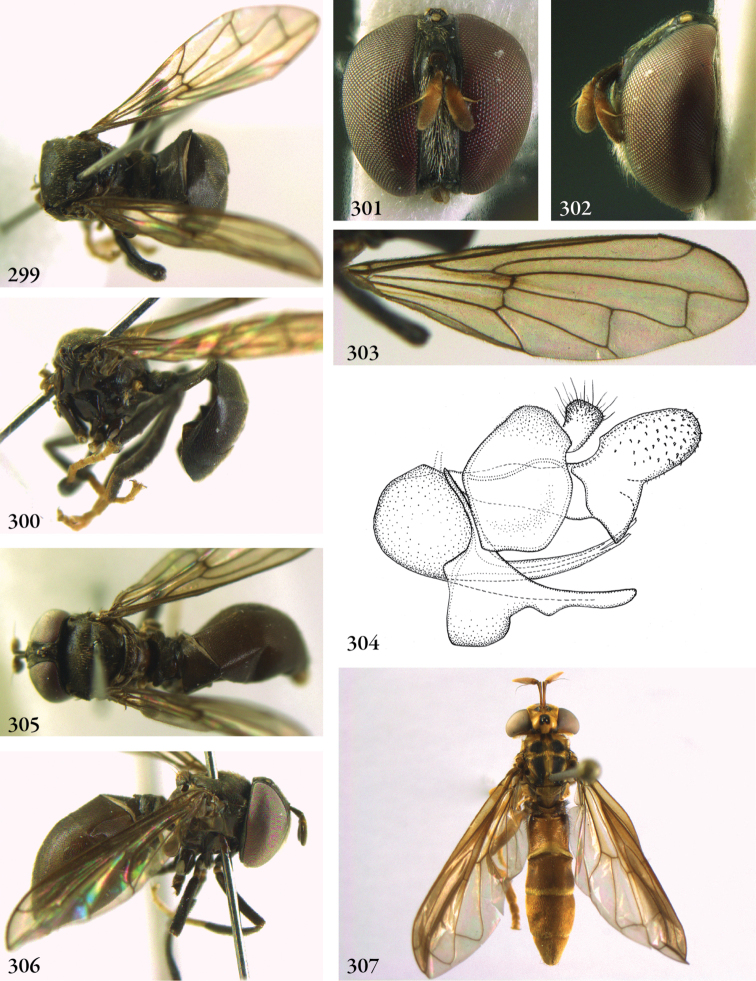
**299–304** *Piruwa phaecada* male (holotype): **299** habitus dorsal **300** habitus lateral **301** head frontal **302** head lateral **303** wing **304** genitalia lateral **305–306** *Piruwa phaecada* female (paratype) **305** habitus dorsal **306** habitus lateral **307** *Pseudomicrodon polistoides* female (holotype), habitus dorsal.

**Figures 308–316. F41:**
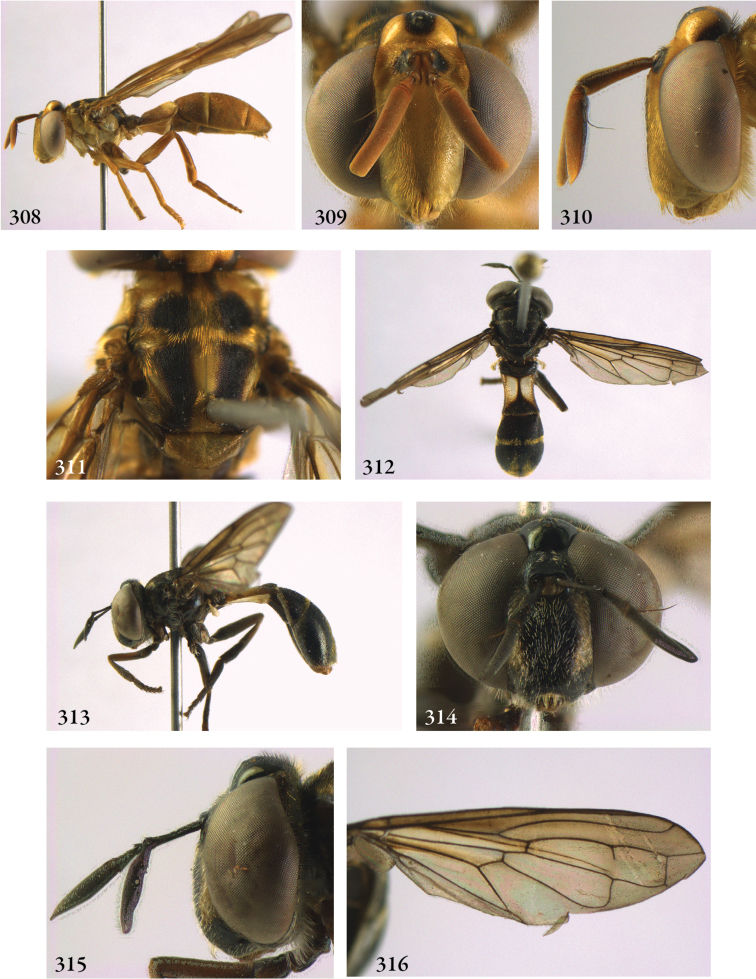
**308–311** *Pseudomicrodon polistoides* female (holotype) **308** habitus lateral **309** head frontal **310** head lateral **311** thorax dorsal **312–316** *Pseudomicrodon smiti* male (holotype): **312** habitus dorsal **313** habitus lateral **314** head frontal **315** head lateral **316** wing.

**Figures 317–324.**
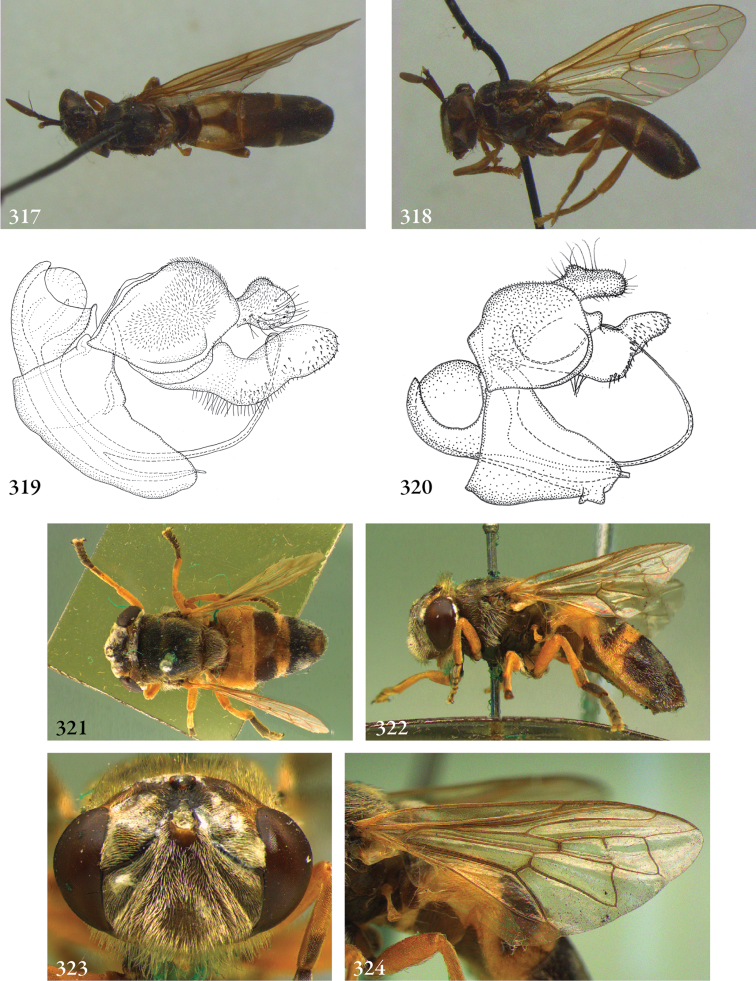
**317–319** *Pseudomicrodon biluminiferus* male (holotype): **317** habitus dorsal **318** habitus lateral **319** genitalia lateral **320** *Pseudomicrodon smiti* male (holotype), genitalia lateral **321–324** *Ptilobactrum neavei* male (holotype): **321** habitus dorsal **322** habitus lateral **323** head frontal **324** wing.

**Figures 325–331.**
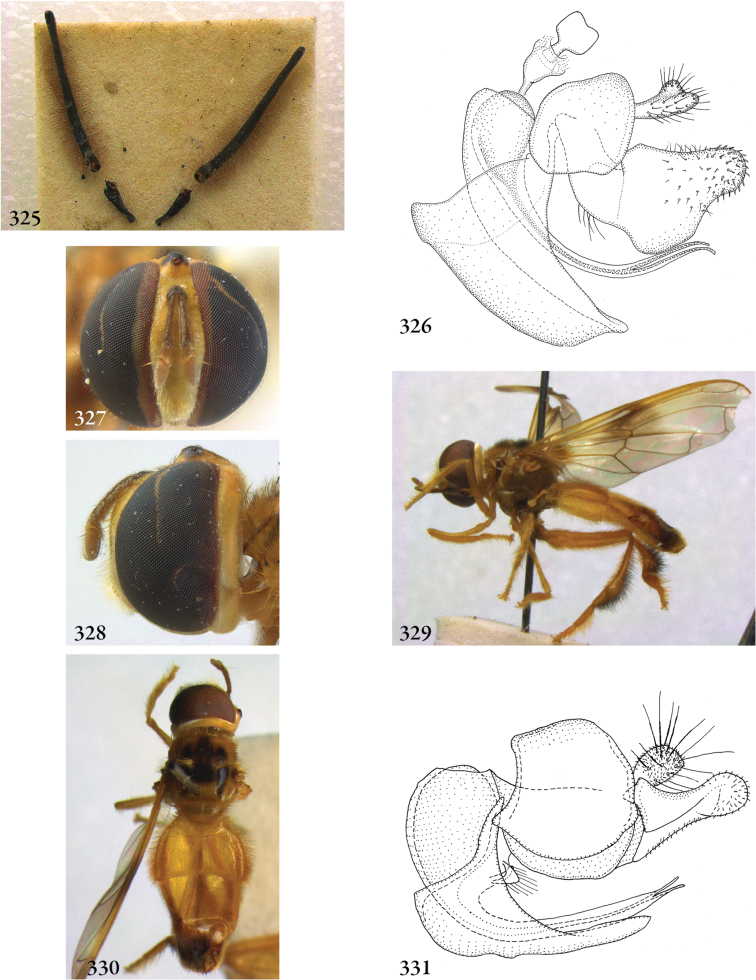
**325–326** *Ptilobactrum neavei* male (holotype): **325** antennae **326** genitalia lateral **327–328** *Rhoga sepulchrasilva* male (Brazil, coll. USNM): **327** head frontal **328** head lateral **329–330** *Rhoga mellea* male (holotype): **329** habitus lateral **330** habitus dorsal **331** *Rhoga sepulchrasilva* male (Brazil, coll. USNM), genitalia lateral.

**Figures 342–350. F45:**
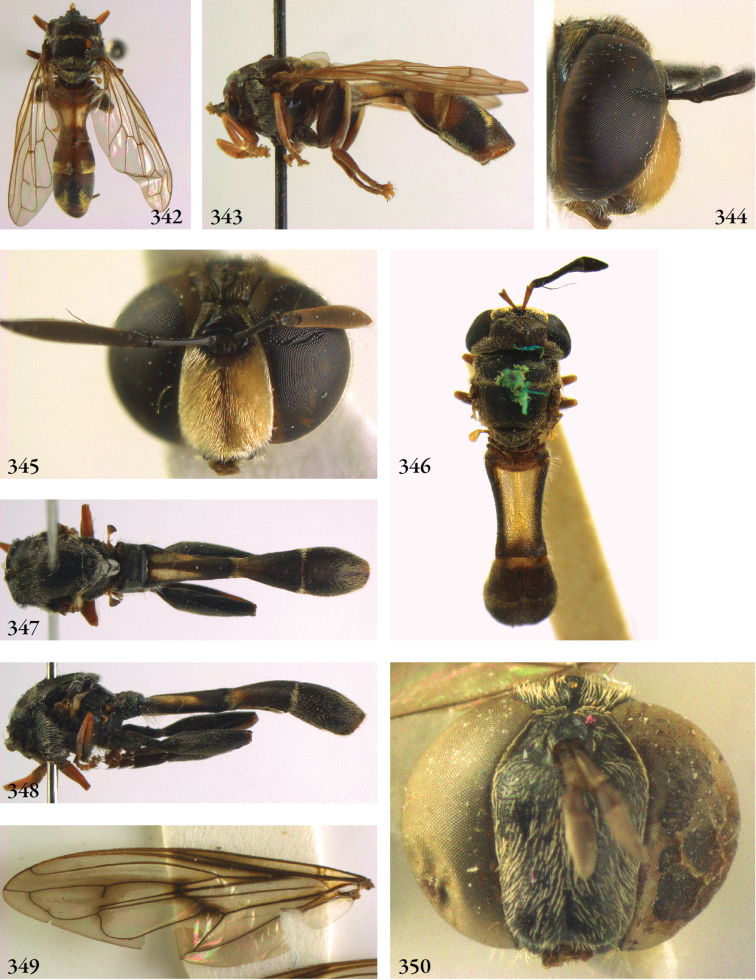
**342–345** *Rhopalosyrphus* (s.l.) *abnormoides* male (holotype): **342** habitus dorsal **343** habitus lateral **344** head lateral **345** head frontal **346** *Rhopalosyrphus* (s.l.) *cerioides* male (holotype), habitus dorsal **347–350** *Rhopalosyrphus* (s.l.) *oreokawensis* male (holotype): **347** habitus dorsal **348** habitus lateral **349** wing **350** head frontal.

**Figure 351–355.**
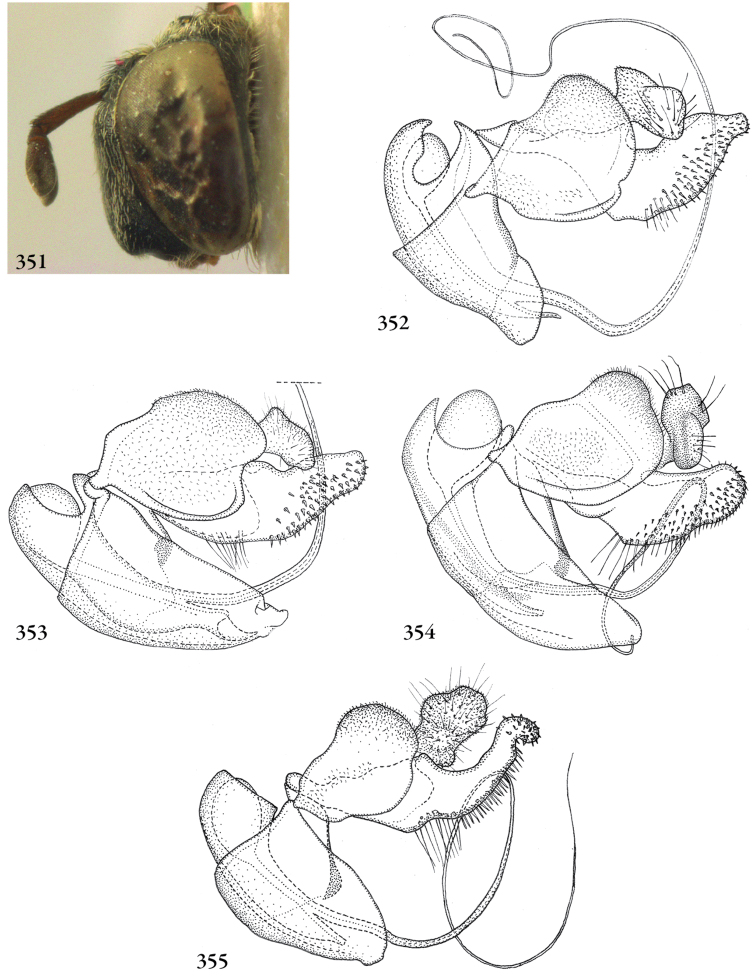
**351** *Rhopalosyrphus* (s.l.) *oreokawensis* male (holotype), head lateral **352–355** *Rhopalosyrphus*, male genitalia: **352** *Rhopalosyrphus ecuadoriensis* **353** *Rhopalosyrphus abnormoides* **354** *Rhopalosyrphus cerioides* **355** *Rhopalosyrphus oreokawensis*.

**Figures 356–363.**
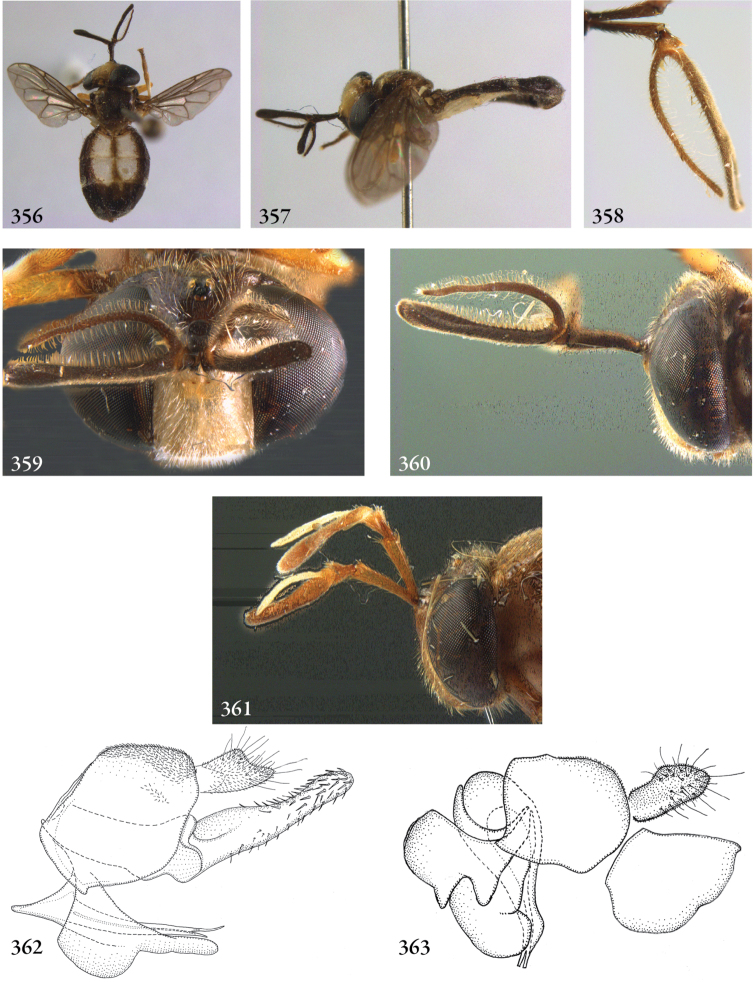
**356–360** *Schizoceratomyia barretoi* male (Surinam, coll. RMNH): **356** habitus dorsal **357** habitus lateral **358** antenna **359** head frontal **360** head lateral **361** *Schizoceratomyia barretoi* female (Brazil, coll. RMNH), head lateral **362** *Schizoceratomyia barretoi* male (Surinam, coll. RMNH), genitalia lateral **363** *Schizoceratomyia flavipes* male (Surinam, coll. RMNH), genitalia lateral.

**Figures 364–371.**
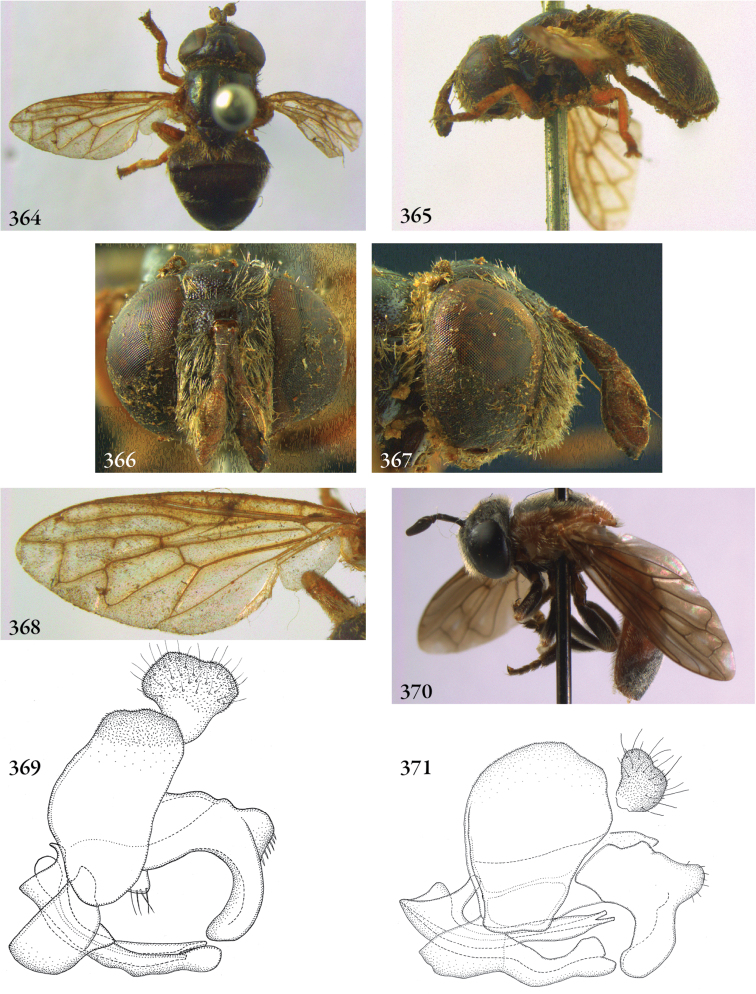
**364–369** *Serichlamys rufipes* male (holotype): **364** habitus dorsal **365** habitus lateral **366** head frontal **367** head lateral **368** wing **369** genitalia lateral **370–371** *Serichlamys scutifer* (USA, Texas, coll. RMNH): **370** habitus lateral **371** genitalia lateral.

**Figures 372–378.**
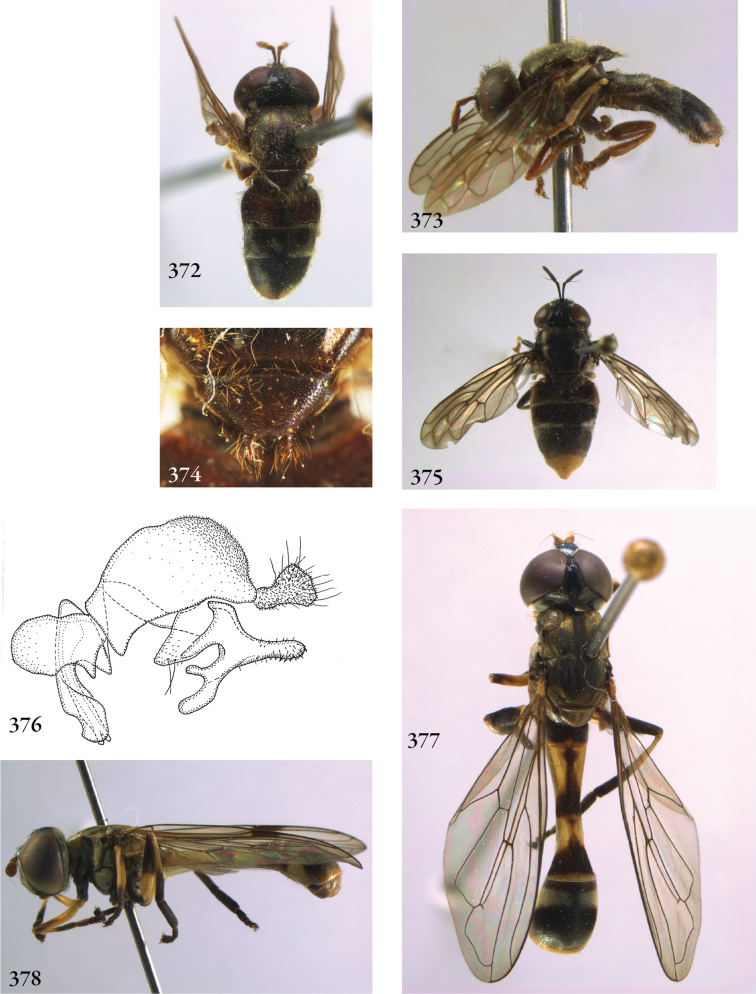
**372–373** *Serichlamys mitis* male (Brazil, coll. RMNH): **372** habitus dorsal **373** habitus lateral **374–375** *Serichlamys* spec.: **374** male, scutellum (Brazil, coll. RMNH) **375** female, habitus (Ecuador, coll. RBIN) **376** *Serichlamys mitis* male (Brazil, coll. RMNH), genitalia lateral **377–378** *Spheginobaccha macropoda* male (Vietnam, coll. RMNH): **377** habitus dorsal **378** habitus lateral

**Figure 379–387.**
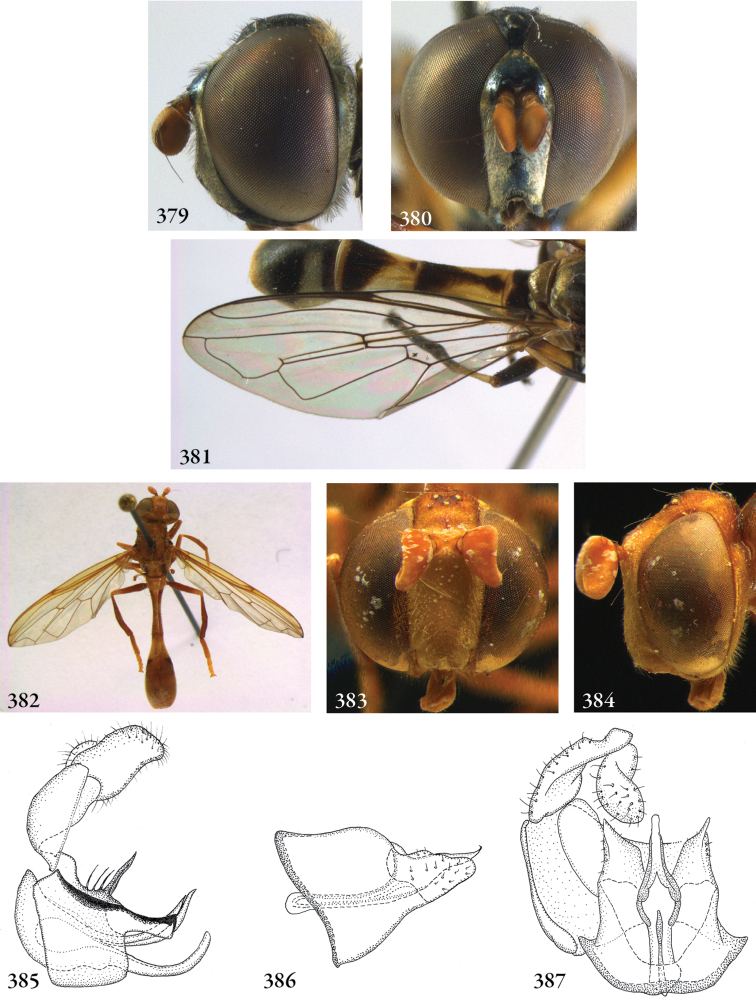
**379–381** *Spheginobaccha macropoda* male (Vietnam, coll. RMNH): **379** head lateral **380** head frontal **381** wing **382–384** *Spheginobaccha guttula* male (holotype): **382** habitus dorsal **383** head frontal **384** head lateral **385–387** *Spheginobaccha*, male genitalia: **385** *Spheginobaccha macropoda* (Vietnam, coll. RMNH), lateral **386** *Spheginobaccha guttula* (holotype), hypandrium, including aedeagus, lateral **387** *Spheginobaccha guttula* (holotype), ventral.

**Figures 388–396.**
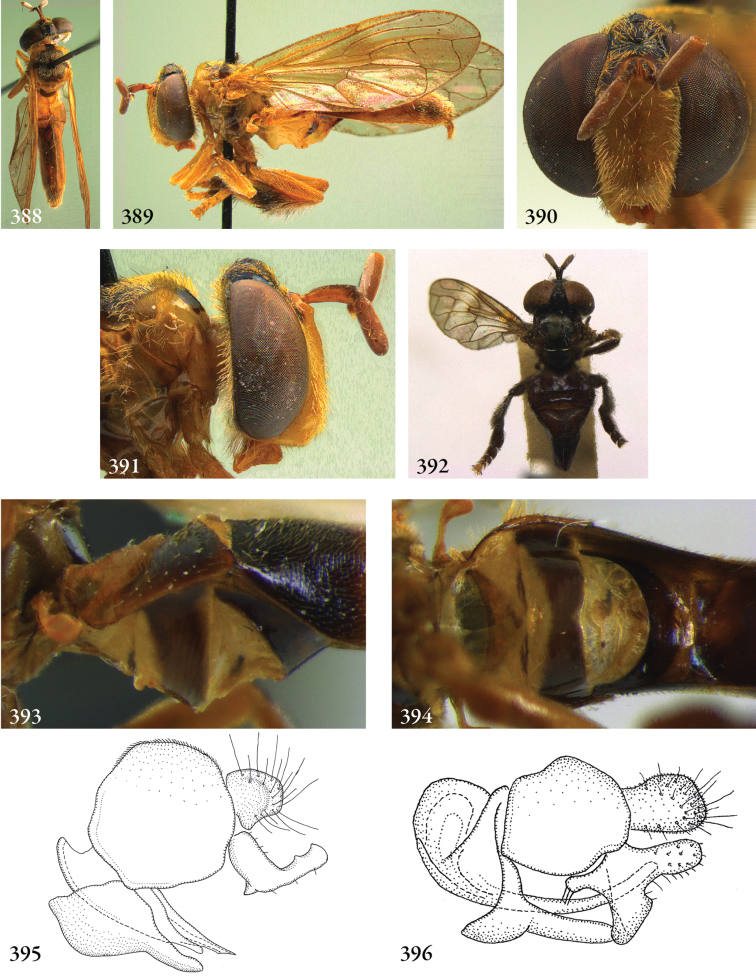
**388–391** *Stipomorpha tenuicauda* female (holotype) **388** habitus dorsal **389** habitus lateral **390** head frontal **391** head lateral **392** *Stipomorpha lacteipennis* male (holotype), habitus dorsal **393–394** *Stipomorpha goettei* male (Surinam, coll. RMNH), base of abdomen: **393** lateral **394** ventral **395–396** *Stipomorpha*, male genitalia: **395** *Spheginobaccha tenuicauda* (Bolivia, coll. M. Hauser) **396** *Spheginobaccha lacteipennis* (Surinam, coll. RMNH).

**Figures 397–405.**
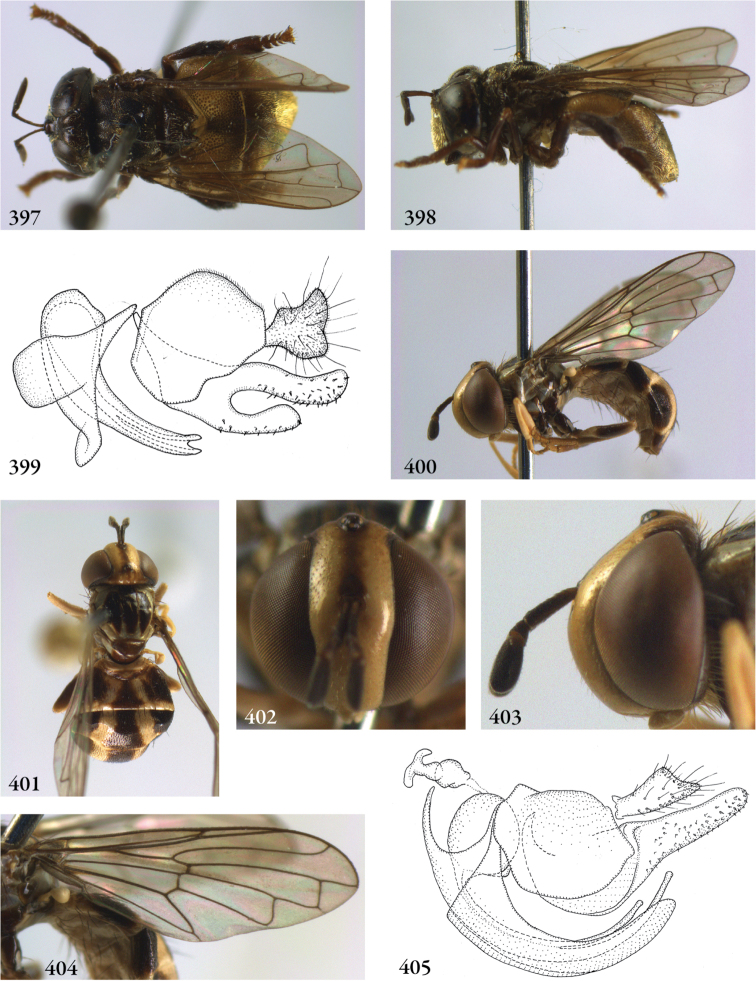
**397–399** *Sulcodon sulcatus* female (Indonesia, Java, coll. RMNH): **397** habitus dorsal **398** habitus lateral **399** genitalia lateral **400–404** *Surimyia rolanderi* female (Surinam, coll. RMNH): **400** habitus lateral **401** habitus dorsal **402** head frontal **403** head lateral **404** wing **405** *Surimyia rolanderi* male (holotype), genitalia lateral.

**Figures 406–414.**
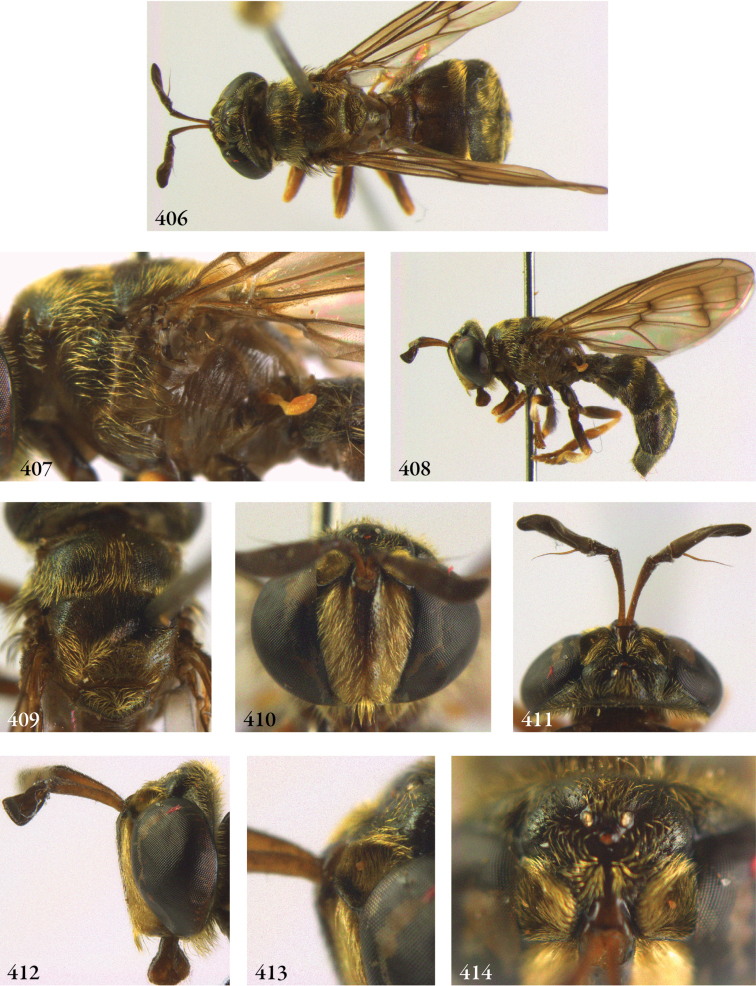
**406–411** *Thompsodon conspicillifrons* female (holotype): **406** habitus dorsal **407** thorax lateral **408** habitus lateral **409** thorax dorsal **410** head frontal **411** head dorsal **412–414** *Thompsodon conspicillifrons* female (holotype): **412** head lateral **413** frons lateral **414** frons frontal.

**Figures 415–421.**
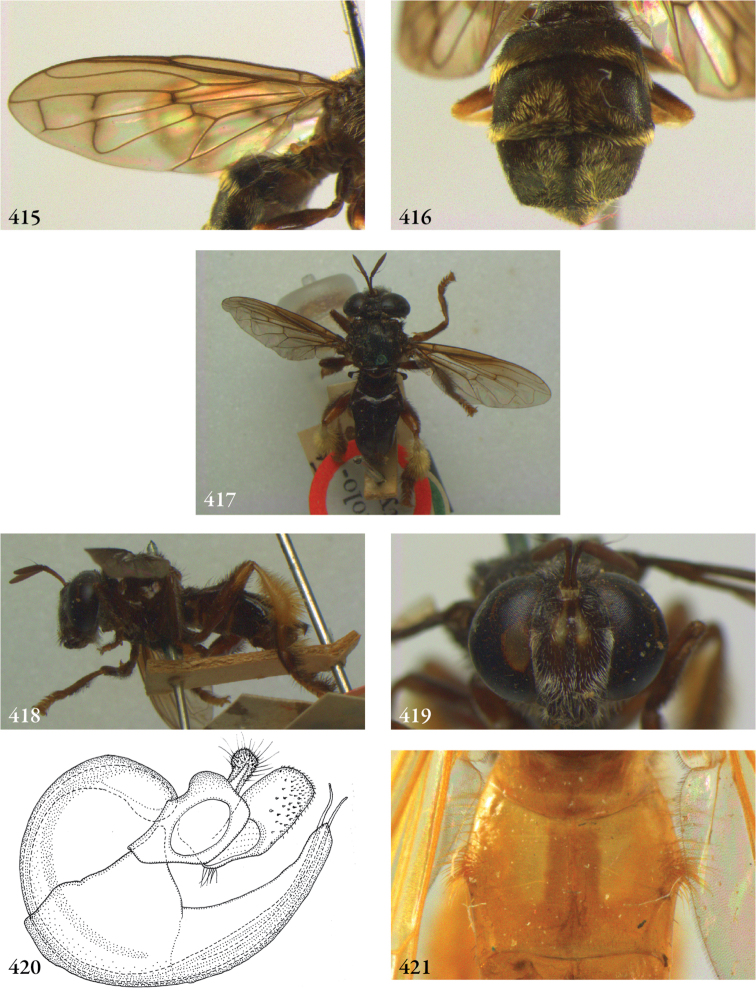
**415–416** *Thompsodon conspicillifrons* female (holotype): **415** wing **416** abdomen dorsal **417–420** *Ubristes flavitibia* male (holotype) **417** habitus dorsal **418** habitus lateral **419** head frontal **420** genitalia lateral **421***Ubristes* spec. female (Brazil, coll. USNM), tergite 2 dorsal.

**Figures 422–429.**
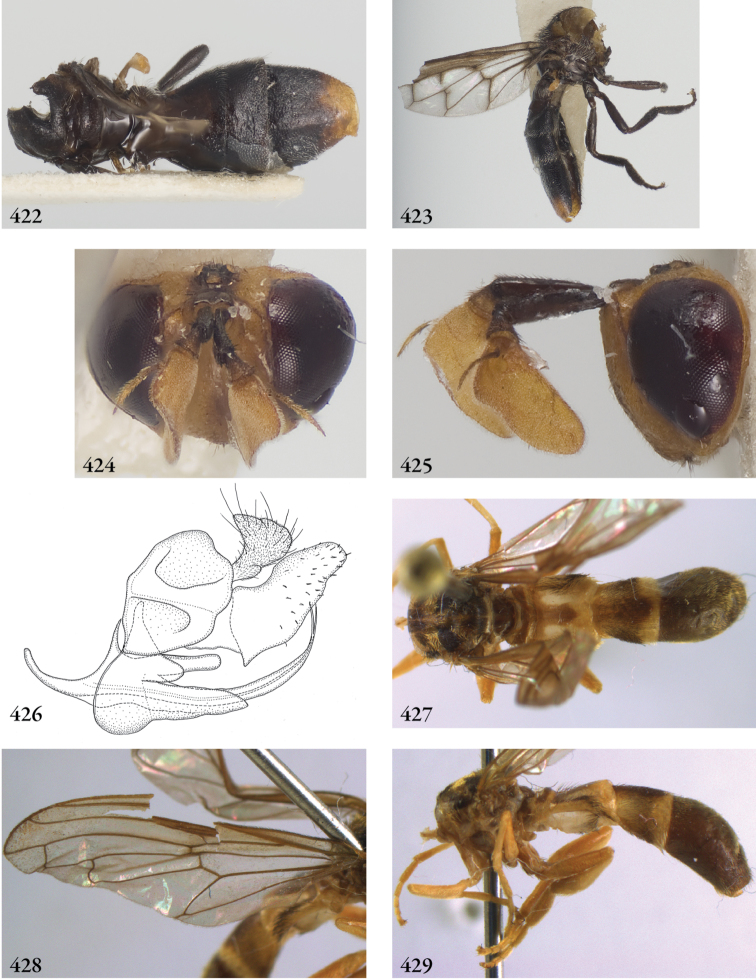
**422–426** Undescribed genus #1 species AUS-01 Thompson in prep., male (Australia, coll. USNM): **422** habitus dorsal **423** habitus lateral **424** head frontal **425** head lateral **426** genitalia lateral **427–429** Undescribed genus #2 species MCR-2 Thompson in prep., male (Costa Rica, coll. RMNH): **427** habitus dorsal **428** wing **429** habitus lateral.

**Figures 430–435.**
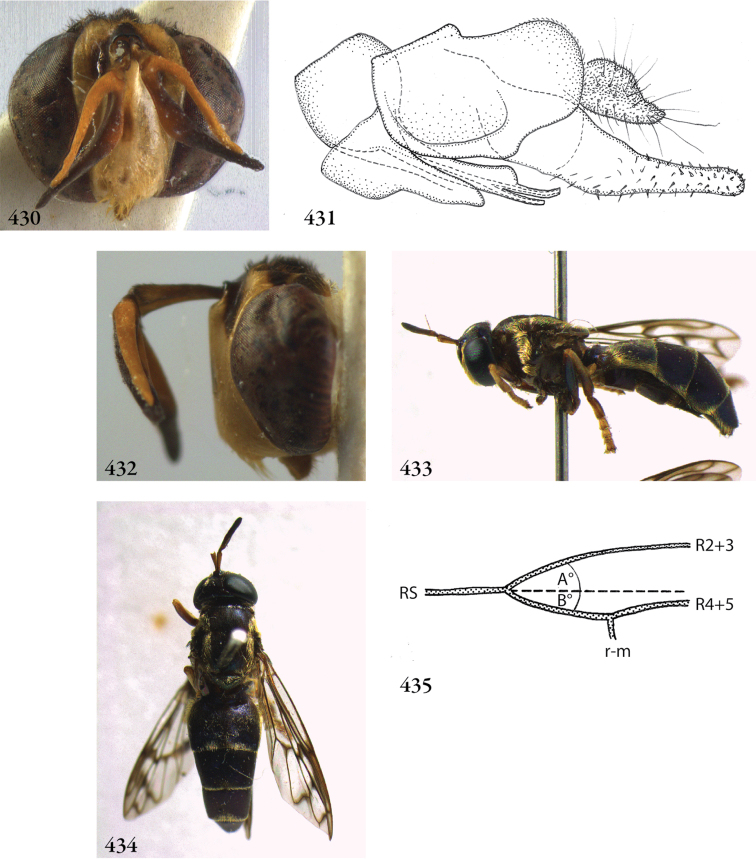
**430–432** Undescribed genus #2 species MCR-2 male (Costa Rica, coll. RMNH): **430** head frontal **431** genitalia lateral **432** head lateral **433–434** *Microdon sharpii* female (New Guinea, coll. BMNH, compared with holotype) **433** habitus dorsal **434** habitus lateral **435** Wing veins in Microdontinae (wing base on left side). The dashed line indicates an (imaginary) apical extrapolation of vein RS. In Microdontinae, vein R2+3 is strongly curved basally, resulting in angle A always being larger than angle B.

## Appendix 1. Descriptions of new species

This section contains descriptions of 26 previously undescribed species, and a redescription of *Ceratrichomyia behara* Séguy, 1951. Most of these were included in the phylogenetic analyses of Reemer and Ståhls (in press). In addition, some new species are described which were considered interesting for other reasons, for instance because they considerably extend the known range of a genus (e.g. *Ceratrichomyia* from mainland Africa, *Kryptopyga* from Sulawesi). *Ceratrichomyia behara* Séguy, 1951 is redescribed, because the type series was found to contain three different species (see genus account in previous section). Characters additional to those mentioned in the descriptions can be found in the morphological character matrix of Reemer and Ståhls (in press).

Line breaks on the original specimen labels are indicated with a slash (/).

As in all other known Microdontinae except *Spheginobaccha chillcotti* Thompson, 1974, the males are dichoptic (i.e. eyes not touching each other dorsally).

### Archimicrodon malukensis

Reemer sp. n.

urn:lsid:zoobank.org:act:385134E2-9482-409E-AC2D-D432E8315FE4

http://species-id.net/wiki/Archimicrodon_malukensis

#### Type specimens.

HOLOTYPE. Adult male. INDONESIA. Label 1: “INDONESIA: HALMAHEIRA / near Payake. 115 m. / Mal. trap. 18.II-18.III.1995 / C. v. Achterberg & R. de Vries”. Coll. RMNH.

PARATYPES. One male and one female from same locality and date as holotype. One male from Halmaheira, near Akeiamo, alt. 175 m., 18.II-18.III.1995, leg. C. van Achterberg & R. de Vries, coll. RMNH (this specimen was used in the morphological matrix of Reemer and Ståhls in press; voucher code MR124).

#### Diagnosis.

The entirely black head, thorax (including femora and tibiae) and abdomen (whether or not with metallic hues) are shared with five other described *Archimicrodon*-species of the Indo-Australian region (Australia excluded). *Archimicrodon boharti* (Curran, 1947) (Solomon Islands) differs from this species by the metallic blue shining scutellum, clearly contrasting with the non-metallic mesonotum (in *Archimicrodon malukensis* sp. n. mesonotum and scutellum are of the same black colour). The same character also applies to *Archimicrodon limbinervis* (de Meijere, 1908) and *Archimicrodon incisuralis* (Walker, 1865) from New Guinea, and *Archimicrodon purpurescens* (Shiraki, 1963) from Micronesia, which also differ by the black pilose scutellum (white pilose in *Archimicrodon malukensis*). *Archimicrodon grageti* (de Meijere, 1908) (New Guinea) differs by the brownish abdomen and reddish yellow pregenital segments (black in *Archimicrodon malukensis*).

#### Description (based on holotype).

Adult male. Body size: 8 mm.

**Head.** Face occupying about 1/5 of head width in frontal view; black; black pilose, except white pilose on ventral 1/4. Gena hardly developed; black; white pilose. Oral margin not produced. Frons black; black pilose, except white pilose along lateral margin. Vertex black; black pilose. Occiput black; black pilose dorsally, white pilose ventrally. Eye bare. Antennal fossa about as high as wide. Antenna black; antennal ratio approximately as 2:1:3.

**Thorax.** Thorax black, except pleurae brownish. Mesoscutum black pilose, except pale yellow pilose along anterior margin, laterally between postpronotum and notopleuron, and in posterolateral corners. Postpronotum pale yellow pilose. Postalar callus black pilose, except pale yellow pilose at posterior apex. Scutellum semicircular, without calcars; entirely pale yellow pilose. Anepisternum with shallow dorsomedian sulcus; white pilose anterodorsally and posterodorsally, widely bare in between. Anterior anepimeron entirely white pilose. Katepimeron sparsely white pilose along dorsal margin, otherwise bare. Katatergum long microtrichose, anatergum short microtrichose. Calypter and halter pale yellow.

*Wing*: Hyaline, slightly infuscated antero-apically; microtrichose, except bare on cell sc, basal 1/2 of cell c, basal 2/5 of cell r1, most of cell br except microtrichose along vena spuria, posterobasal 1/5 of cell r4+5, basal 5/6 of cell bm, anterobasal 3/5 of cell cup, basomedian 2/3 of alula and basal 1/6 of anal lobe.

*Legs*: Black, except fifth tarsomeres brown; black pilose, except femora posterobasally white pilose and tarsi ventrally golden yellow pilose. Coxae black; white pilose. Trochanters brown; white pilose.

**Abdomen.** Tergites black with faint metallic hues, except for a dull black fascia on anterior 2/5 of tergite 3 and a very narrow, medially interrupted dull black fascia along anterior margin of tergite 4. Tergites 1 and 2 yellowish white pilose. Tergites 3 and 4 black pilose, except white pilose posterolaterally. Sternites blackish brown; sternite 1 bare; sternite 2 yellow pilose; sternite 3 black pilose except yellow pilose along posterior margin; sternite 4 black pilose. Male genitalia as in Fig. 15.

**Female.** 9.5 mm. As male, except for usual sexual differences. Tergite 5 black pilose, except white pilose posteriolaterally.

#### Etymology.

The specific epithet (adjective) is derived from Maluku, the group of islands to which Halmaheira, where the species was found, belongs.

### Ceratrichomyia angolensis

Reemer sp. n.

urn:lsid:zoobank.org:act:7F7925F3-C102-432C-A1C4-167902226596

http://species-id.net/wiki/Ceratrichomyia_angolensis

#### Type specimens.

HOLOTYPE. Male. Label 1: “ANGOLA 30 km NE / Duque de Bragan - / za, Nov. / Dec. 1957”; label 2: “Collector / G.H. Heinrich”. Coll. CNC.

#### Diagnosis.

This species differs from both other known species of *Ceratrichomyia* by the bare postpronotum and katepimeron, the downward projecting face, and the absence of a ventrolateral ridge on the epandrium.

#### Description (based on holotype).

Adult male. Body size: 10 mm.

**Head.** Face occupying approximately 1/2 of head width in frontal view; yellowish brown, except for blackish marks dorsally along eye margin; entirely yellow pilose; with pit-like depressions on dorsal 1/3; face profile more or less straight, strongly produced ventrally below eye margin. Gena yellowish brown. Lateral oral margins not produced. Frons and vertex yellowish brown, a little blackish at and around ocellar triangle; yellow pilose. Occiput yellow; dorsally wide and yellow pilose, ventrally narrow and whitish pilose. Eye bare. Antennal fossa about as wide as high. Antenna orange brown, except basoflagellomere blackish brown; antennal ratio approximately as 6:1:18. Basoflagellomere very long, entirely covered with pile at least as long as 1.5 times diameter of basoflagellomere. Arista very small, shorter than pedicel; situated at about 1/3 from base of basoflagellomere.

**Thorax.** Mesonotum dorsally black, with margins widely yellowish brown, and with pair of narrow yellow submedian vittae, dividing black into three approximately equally wide parts; short, appressed yellow pilose; transverse suture yellowish brown. Postpronotum yellowish brown; bare. Postalar callus yellowish brown; yellowish pilose. Scutellum blackish brown; yellow pilose; without calcars. Pleurae orange brown. Anepisternum with deep sulcus separating anterior and posterior part; entirely yellow pilose. Anepimeron entirely yellow pilose. Katepisternum yellow pilose dorsally and ventrally. Katepimeron bare. Katatergum with short microtrichia, anatergum bare. Calypter yellowish brown. Halter yellow.

*Wing*: hyaline; microtrichose, except bare on cells bc and c, basal 1/2 of cell r1, almost entirely on cells br, bm, cup and on alula, and posterobasal 1/5 of cell r4+5. Vein bm-cu shorter than basal section of CuA1.

*Legs*: Orange brown except femora blackish brown on basal 1/2; yellow pilose. Coxae and trochanters blackish brown; pale pilose.

**Abdomen.** Constricted at 2nd segment, with narrowest point at posterior margin, widest halfway of tergite 4 (slightly wider than thorax). Tergites 1-3 fused. Tergite 1 dark brown; yellow pilose. Tergite 2 pale yellow; yellow pilose along lateral margin. Tergites 3 and 4 dark brown; yellow pilose. Sternite 1 yellow; bare. Sternite 2 yellow anteriorly, brown posteriorly; mixed yellow and black pilose. Sternite 3 dark brown; black pilose. Sternite 4 concealed behind genital capsule; brown; yellow pilose. Genitalia as in Fig. 56.

**Female unknown.**

#### Etymology.

The specific epithet (adjective) is derived from Angola, where the species was found.

### Ceratrichomyia behara

Séguy, 1951 (redescription)

http://species-id.net/wiki/Ceratrichomyia_behara

Figs 46, 47 , 57

Ceratrichomyia behara Séguy, 1951: 14. Type locality: Madagascar, Tananarivo. Coll. MNHN.

#### Type specimens.

LECTOTYPE. Male. Label 1: “Madagascar, Behara”; label 2 (blue): “Museum Paris, III-38, A. Seyrig”; label 3 (red): “Type”; label 4: “*Ceratrichomyia behara* type du genre [male symbol] Séguy 50”. Coll. MNHN. See genus account of *Ceratrichomyia* for notes on lectotype designation.

#### Diagnosis.

This species differs from *Ceratrichomyia angolensis* sp. n. by the pilose postpronotum and katepimeron. From *Ceratrichomyia bullabucca* sp. n. it differs by the straight face profile and the parallel-sided tergite 2.

#### Redescription (based on lectotype).

Adult male. Body size: 7 mm.

**Head.** Face occupying approximately 1/2 of head width in frontal view; yellow; entirely yellowish white pilose; depressed on lateral 1/3; face profile more or less straight. Gena yellow. Lateral oral margins not produced. Frons and vertex yellow; yellow pilose. Occiput yellow; dorsally wide and yellow pilose, ventrally narrow and whitish pilose. Eye bare. Antennal fossa about as wide as high. Antenna orange brown, getting dark brown towards apex of basoflagellomere; antennal ratio approximately as 1:0,2:3,5. Basoflagellomere very long, entirely covered with pile at least twice as long as diameter of basoflagellomere. Arista very small, shorter than pedicel.

**Thorax.** Mesoscutum, postpronotum, postalar callus and scutellum reddish brown; short, yellow pilose. Scutellum without calcars. Pleurae orange brown. Anepisternum with deep sulcus separating anterior and posterior part; entirely whitish pilose. Anepimeron entirely pale pilose. Katepisternum densely white pilose dorsally; sparsely pilose ventrally. Katepimeron white pilose. Katatergum with long microtrichia, arranged in oblique rows. Anatergum short microtrichose. Calypter brownish. Halter yellow.

*Wing*: hyaline; microtrichose, except bare on cell bc, basally on cell r1 along vein Rs, on most of cell br except microtrichose along vena spuria, on most of cell bm except apical 1/8, basal 1/2 of cell cup. Vein bm-cu shorter than basal section of CuA1.

*Legs*: Orange except femora blackish with orange apical 1/4; pale pilose, except tarsi dorsally black pilose. Coxae and trochanters blackish brown; pale pilose.

**Abdomen.** Constricted at 2nd segment, with tergite 2 parallel-sided, widest at tergite 3 and 4 (slightly wider than thorax). Tergite 1 dark brown; white pilose. Tergite 2 dorsoventrally flattened, dark brown with large, triangular yellow maculae along lateral margin, posteriorly interconnected and reaching posterior margin, which is entirely yellow; white pilose. Tergite 3 and 4 dark brown with yellow posterior margins; white to yellow pilose. Tergite 4 with two faint submedian grooves from anterior margin to just before posterior margin. Sternite 1 yellow; bare. Other sternites brown; white pilose. Genitalia as in Fig. 57.

**Female.** Unknown.

### Ceratrichomyia bullabucca

Reemer sp. n.

urn:lsid:zoobank.org:act:2B7970AE-82E2-45A6-B496-213889A5EA60

http://species-id.net/wiki/Ceratrichomyia_bullabucca

#### Type specimens.

HOLOTYPE [this specimen is one of the paratypes of *Ceratrichomyia behara* Séguy]. Male. Label 1: “Madagascar, Bekily, Rég. sud de l’ile”; label 2 (blue): “Museum Paris, X.36, A. Seyrig”; label 3: “*Ceratrichomyia behara* cotype, male, Séguy 1950”. Coll. MNHN.

#### Diagnosis.

This species differs from *Ceratrichomyia angolensis* sp. n. by the pilose postpronotum and katepimeron. From *Ceratrichomyia behara* Séguy, 1951 it differs by the convex face profile and the anteriorly widened tergite 2.

#### Description.

Adult male. Body size: 8.5 mm. As *Ceratrichomyia behara*, except for differences listed below.

**Head.** Face occupying almost 3/5 of head width in frontal view. Face profile clearly convex.

**Thorax.** Katepisternum bare ventrally. Calypter yellow.

*Wing*: cell r1 entirely microtrichose, cell br bare on posterobasal 3/5. Vein bm-cu longer than basal section of CuA1. **Abdomen.** Tergite 2 not parallel-sided: narrowest point at about half its length; lateral yellow macula, not connected posteriorly. Genitalia as in Fig. 58.

**Female.** Unknown.

#### Etymology.

The specific epithet (noun in apposition) contains the Latin words *bulla* (bubble, knob) and *bucca* (cheek) and refers to the swollen face, a character to distinguish the species from *Ceratrichomyia behara*.

### Domodon zodiacus

Reemer sp. n.

urn:lsid:zoobank.org:act:78CD4D02-D4C0-434D-ACDE-1A9D214DD9B2

http://species-id.net/wiki/Domodon_zodiacus

#### Type specimens.

HOLOTYPE. Male, SURINAM, Paramaribo Zoo, 05°50'30"N, 55°09'29"W, malaise trap, 18-27.II.2006, leg. M. Reemer. Coll. RMNH.

#### Diagnosis.

Three undescribed species belonging to this genus are known. From those, *Domodon zodiacus* sp. n. can be distinguished by the following combination of characters: face with black median vitta, alula entirely microtrichose, tergites 3 and 4 partly yellow.

#### Description (based on holotype).

Adult male. Body size: 7 mm.

**Head.** Dichoptic. Face occupying about 1/3 of total head width in frontal view; pale yellow with brown median vitta of 1/5 of facial width; entirely yellow pilose; not pollinose; eye margins slightly converging at level of frons, with smallest distance approximately equal to three times width of antennal fossa. Gena black. Oral margin laterally produced; black. Antennal fossa about as wide as high. Frons black with metallic green shine; golden pilose. Vertex convexly produced; shining black; sparsely short pilose. Ocellar triangle not elevated; anterior angle about 100°. Occiput narrow; black; golden yellow pilose dorsally, white pilose ventrally. Eye bare. Antenna dark brown; antennal ratio approximately 4:1:4; basoflagellomere elongate with rounded apex, with small sensory pit located at about 1/3 from base; arista slender, about 2/3 of length of basoflagellomere.

**Thorax.** Mesoscutum black with faint metallic hues; black pilose, except for a narrow sutural and a wide prescutellar fascia of golden pilosity. Postpronotum blackish; yellow pilose. Postalar callus brown; yellow pilose. Scutellum with two apical calcars of 1/4 of length of scutellum; brown with faint metallic hues. Pleurae blackish brown. Anepisternum with anterior and posterior part separated by clear sulcus; anterior part short black pilose, posterior part long yellow pilose, with bare area in between. Anterior anepimeron entirely pale yellow pilose. Katepisternum yellow pilose dorsally, bare ventrally. Katatergum with long black microtrichia. Anatergum short pale microtrichose. Other pleurites bare. Calypter and halter yellow.

*Wing*: Hyaline, faintly darker around crossvein r-m; microtrichose, except bare on cell bc, posterobasal 1/2 of cell c, basally on cell r1 along vein Rs, on cell br except along vena spuria and extreme apex, on posterobasal 1/2 of cell bm, on anterobasal 1/2 of cell cup.

*Legs*: Anterior four legs pale brown, with vaguely defined darker and paler parts; femora black pilose except mid-femur pale pilose posteriorly; tibiae pale pilose dorsally, black pilose ventrally; tarsi black pilose except last tarsomere yellow pilose. Hind femur blackish with apical 1/3 yellow; black pilose anteriorly, pale pilose posteriorly. Hind tibia dark brown with pale apex; black pilose dorsally, pale pilose ventrally. Hind tarsus brown with last tarsomere yellowish; black pilose, except last tarsomere yellow pilose.

**Abdomen.** Ratio of median tergal lengths approximately as 1:2:3:5. Tergites 3 and 4 not clearly fused, only laterally. Tergite 1 black; pale pilose. Tergite 2 pale yellow with lateral 1/4 black and with posteriomedian black macula; yellow parts yellow pilose, black parts black pilose. Tergite 3 pale yellow with extreme lateral margins black, with sublateral oblique black maculae of slightly less than 1/3 of tergal width, with narrow median black vitta on anterior 2/3; black pilose except yellow pilose along posterior margin. Tergite 4 black except yellow along lateral and posterior margins; black pilose except yellow pilose on yellow parts. Sternite 1 black; bare. Other sternites yellow, sparsely pilose. Genitalia as in 84.

**Female.** Unknown**.**

#### Etymology.

The name *zodiacus* (adjective, Greek: ‘of animals’) was chosen because the type specimen was collected at the Paramaribo Zoo.

### Furcantenna nepalensis

Reemer sp. n.

urn:lsid:zoobank.org:act:603D5305-E43A-4BDD-90F6-CE796366D40C

http://species-id.net/wiki/Furcantenna_nepalensis

#### Type specimens.

HOLOTYPE. Adult male. NEPAL. Label 1: “NEPAL, Ktmd. / Godavari 6000’ / 13 Aug. 1967 / Can. Nepal Exped.”. Coll. CNC.

#### Diagnosis.

Three characters are mentioned by Cheng and Thompson (2008) to distinguish *Furcantenna* Cheng, 2008 from the Neotropical genus *Schizoceratomyia* Carrera, Lopes & Lane, 1947: scutellum apicomedially sulcate, katepisternum pilose, metasternum developed and pilose. All three characters are found in the species described here. Only one other species of *Furcantenna* is known (*Furcantenna yangi* Cheng, 2008). From that species, *Furcantenna nepalensis* sp. n. differs by the following characters (characters of *Furcantenna yangi* in parentheses, based on Cheng and Thompson 2008): body colour brownish, without violet shine (black, with violet shine); mesoscutum entirely golden pilose (black and white pilose); katepimeron pilose (bare); tergite 2 with ratio of median length : width of posterior margin approximately as 1:3 (1:6).

#### Description (based on holotype).

Adult male. Body size: 10 mm.

**Head.** Blackish brown. Face occupying about 1/2 of head width in frontal view; laterally depressed and dull, medially with shining carina; white pilose. Gena white pilose. Oral margin not produced. Frons, vertex and occiput golden pilose. Eye bare. Antennal fossa slightly higher than wide. Antenna: scape pale brown, pedicel and basoflagellomere black; antennal ratio approximately as 5:1:17.5; basoflagellomere bifurcate at base, both branches entirely long pilose; arista absent.

**Thorax.** Mesoscutum black, except pale brown along margins; golden pilose. Postpronotum and postalar callus brown; golden pilose. Scutellum trapezoid, although slightly sulcate apicomedially; without calcars; brown; golden pilose. Pleurae brown. Anepisternum with deep median sulcus; golden pilose, except bare on ventral 1/5. Anterior anepimeron entirely golden pilose. Katepisternum white pilose dorsally, bare ventrally. Katepimeron white pilose. Katatergum long microtrichose, anatergum short microtrichose. Calypter and halter pale yellow.

*Wing*: Hyaline, tinged with brown, especially on anterior half. Microtrichose, except bare on basal 2/3 of cell bc, posterobasal 1/2 of cell br, and anterobasal 1/2 of cell bm.

*Legs*: Brown. Front leg golden yellow pilose, except tarsus dorsally black pilose. Mid and hind legs black pilose, except femora largely golden yellow pilose and tarsi ventrally golden pilose. Coxae and trochanters brown; whitish pilose.

**Abdomen.** Tergites dark brown, a little paler along lateral margins and entirely on tergite 4. Tergite 1 yellow pilose. Tergites 2 and 3 golden yellow pilose anteriorly and laterally, black pilose medially and posteriorly. Tergite 4 entirely golden pilose. Sternites dark brown; all sternites yellowish white pilose. Male genitalia as in Fig. 80.

**Female.** Unknown.

#### Etymology.

The name *nepalensis* (noun in the genitive case) refers to the type locality.

### Heliodon doris

Reemer sp. n.

urn:lsid:zoobank.org:act:264A9CEC-A0C4-44AE-8160-7D44E7C8DACD

http://species-id.net/wiki/Heliodon_doris

#### Type specimens.

HOLOTYPE. Adult male. THAILAND. Label 1: “THAILAND Ubon Ratchathani, Pha Taem / NP, west of Huay Pok substation, 438 m, / Malaise trap, 15°37.212'N, 105°36.903'E, / 25.iv–2.v.2007, Bunlu Sapsiri leg. T2173”; label 2: “Voucher code M. Reemer / 314 / DNA voucher labcode MZH:Y1074”. Coll. QSBG.

PARATYPES. THAILAND: Adult female; label 1: “THAILAND, Loei, T485 / Phu Kradueng NP, Malaise / 16°51.958'N, 101°50.668'E / 16.VIII-23.VIII.2006. 280 m / Sutin Khonglasae leg.”; coll. RMNH. MALAYSIA: Adult male; label 1: “G.6722 / Malaya / Selangor / F.E.S. Serdang / 22.3.1956”; label 2: “COM INST ENT. / COLL NO 14927”; label 3: “Microdon / [female sign] not in B.M. / van Emdendet. 1956”; label 4: “Pres. by / Com. Inst. Ent. / B.M. 1956-712”; label 5: “Microdontini / ? new genus / N.P. Wyatt det. 1985”. Coll. BMNH.

#### Diagnosis.

Within *Heliodon*, no other species has tergites 3 and 4 predominantly yellow.

#### Description (based on holotype).

Adult male. Body size: 9 mm.

**Head.** Face occupying slightly more than 1/4 of head width in frontal view; yellow; yellow pilose. Gena yellow; yellow pilose. Lateral oral margins weakly produced. Frons blackish brown; golden pilose. Vertex blackish; golden pilose. Occiput black; golden yellow pilose dorsally, whitish yellow pilose ventrally. Eye bare. Antennal fossa about as high as wide. Antenna brown, except scape and pedicel yellow ventrally; antennal ratio approximately as 3.5:1:2.5.

**Thorax.** Mesoscutum black with metallic hues, except yellow around postpronotum, anteriad of notopleuron and around postalar callus; golden pilose. Postpronotum, postalar callus and scutellum yellow; golden pilose. Scutellum semicircular; with pair of apical calcars with mutual distance slightly larger than length of scutellum. Pleurae yellow, except anepisternum dark brown along anterior margin, katepisternum dark brown ventrally, meron and metanotum dark brown; all pilosity golden yellow. Anepisternum enitrely pilose, except narrowly bare along ventral margin; with shallow sulcus separating anterior from posterior part. Anepimeron entirely pilose. Katepisternum pilose dorsally, bare ventrally. Katatergum long microtrichose, anatergum short microtrichose. Calypter and halter pale yellow.

*Wing*: Hyaline, slightly darkened around apical crossveins; microtrichose, except bare on cell sc, basal 3/4 of cell c, basal 1/3 of cell r1, entirely on cell br except microtrichose along vena spuria, basal 1/4 of cell r4+5, basal 3/4 of cell bm, anterobasal 3/4 of cell cup and basomedian 1/2 of alula.

*Legs*: Front leg yellow; yellow pilose. Other legs missing in holotype. [Paratype male: mid leg yellow, yellow pilose; hind femur dark brown except yellow on basal 1/4 and apical 1/10, yellow pilose; hind tibia yellow on basal 3/5, dark brown on apical 2/5, yellow pilose; hind tarsus with first tarsomere dark brown dorsally, otherwise yellow, yellow pilose.] Front and mid coxae yellow; yellow pilose. Hind coxa dark brown basally, yellow apically; yellow pilose. Trochanters yellow; yellow pilose.

**Abdomen.** Slightly constricted, with narrowest point at posterior margin of tergite 2. Tergite 1 dark brown; yellow pilose. Tergite 2 yellow with dark brown, triangular median macula, with narrowest part at anterior margin and widest part close to posterior margin; yellow pilose, except dark pilose laterally. Tergite 3 yellow with median dark brown vitta and pair of oblique, lateral, dark brown maculae; yellow pilose, except dark pilose on and around lateral dark maculae. Tergite 4 with colour pattern similar to tergite 3, but lateral maculae anteriorly confluent with median vitta; golden yellow pilose, except dark pilose on lateral maculae. Sternites yellow, slightly darkened on sternite 1, 3 and 4; yellow pilose. Male genitalia as in Fig. 94.

**Female.** As male, except for following differences. Dark maculae on tergite 4 not confluent anteriorly. Tergite 5 yellow with dark brown median vitta and pair of small, round, submedian dark brown spots; golden yellow pilose, except for black pile anteromedially and sublaterally. Sternite 3 dark brown medially. Sternites 4 and 5 dark brown.

#### Etymology.

This species is named after my daughter Doris. The epithet is a noun in apposition.

### Heliodon elisabethanna

Reemer sp. n.

urn:lsid:zoobank.org:act:B6CB7238-C92A-4641-B824-EE738907F179

http://species-id.net/wiki/Heliodon_elisabethanna

#### Type specimens.

HOLOTYPE. Adult female. THAILAND. Label 1: “THAILAND / 2007”; label 2: “Voucher code M. Reemer / 316 / DNA voucher labcode MZH:Y1062”. Coll. QSBG. No further locality data available.

#### Diagnosis.

No other species of *Heliodon* has entirely black legs.

#### Description (based on holotype).

Adult female. Body size: 12 mm.

**Head.** Face occupying about 1/3 of head width in frontal view; black; entirely golden pilose. Gena black; golden pilose. Lateral oral margins produced. Frons black; black pilose, except golden pilose posterolaterally. Vertex black; black pilose, except golden pilose along anterior margin and white pilose along posterior margin. Occiput black; black pilose on dorsal 1/3, white pilose on ventral 2/3. Eye pale pilose, with pile approximately as long as half the diameter of frontal ocellus. Antennal fossa about as high as wide. Antenna brown; antennal ratio approximately as 3:1:2.5.

**Thorax.** Mesoscutum black; black pilose, with inconspicuous pale pile along anterior margin and along lateral 1/3 of transverse suture. Postpronotum brownish; pale pilose. Postalar callus black on anterior 3/4, brown on posterior 1/4; black pilose dorsally, pale pilose laterally. Scutellum semicircular; with pair of distinct apical calcars with mutual distance about 1/3 of width of scutellum at base; black; black pilose anteriorly and dorsally, long golden pilose posteriorly. Pleurae black. Anepisternum black pilose, except white pilose on ventral 1/3 and narrowly along anterior margin; with very small bare patch ventrally on anterior part; with deep sulcus separating anterior from posterior part. Anepimeron black pilose on dorsal 1/3, white pilose on ventral 2/3. Katepisternum white pilose dorsally, bare ventrally. Katatergum long microtrichose, anatergum short microtrichose. Calypter greyish. Halter white.

*Wing*: Hyaline, except vaguely infuscated around apical crossveins, around spur on vein R4+5, around base of R2+3 and crossvein r-m; microtrichose, except bare on cell bc, basal 1/2 of cell c, basally on cell r1 along vein Rs, entirely bare on cell br except microtrichose along vena spuria, on basal 1/2 of cell r4+5, basal 1/2 of cell bm, basal 1/3 of cell cup, basomedian 2/3 of alula.

*Legs*: Black, except front femur brown anteriorly; white pilose, except golden pilose basoventrally on front and mid femora and apicoventrally on front and mid tibiae, and golden pilose ventrally on tarsi. Coxae and trochanters black; white pilose.

**Abdomen.** Tergites black, except for large yellow maculae posterolaterally on tergite 2, and narrow, medially interrupted yellow fasciae along posterior margins of tergites 3 and 4. Tergite 1 inconspicuously pale pilose. Tergite 2 with thick, conspicuous, appressed golden pile (tomentum), except narrowly black pilose along anterior margin. Tergite 3 with medially interrupted fascia of golden tomentum on posterior 2/5, inconspicuous golden pile on lateral 1/3 and inconspicuous black pile anteriorly. Tergite 4 with medially interrupted fascia of golden tomentum on posterior 1/2, inconspicuous golden pile on lateral 1/4 and inconspicuous black pile anteriorly. Tergite 5 medially with pair of large, medially connected patches of golden tomentum, mixed black and golden pilose otherwise. Sternites brownish; sternites 1-3 yellowish pilose; sternites 4 and 5 black pilose.

**Female.** Unknown.

#### Etymology.

This species is named after my partner Elisabeth (Liesbeth) Anna. The epithet is a noun in apposition.

### Heliodon tiber

Reemer sp. n.

urn:lsid:zoobank.org:act:E3CFF355-9DA9-42C1-B41E-17A873962AA0

http://species-id.net/wiki/Heliodon_tiber

#### Type specimens.

HOLOTYPE. Adult male. INDONESIA (Sumatra). Label 1: “Fort de Kock / (Sumatra) 920 M. / 1924 / leg. E. Jacobson”. Coll. ZMAN.

PARATYPE. Adult male. INDONESIA (Sumatra). Label 1: “Fort de Kock / (Sumatra) 920 M. / 1925 / leg. E. Jacobson”; label 2: “Microdon / fascipennis / Sack”. Coll. ZMAN.

#### Diagnosis.

This is the only known species of *Heliodon* in which the hind femur is entirely yellow.

#### Additionally studied specimens.

MALAYSIA: 2 males, Selangor, Gombak Field Stn., 14.XI.1977, leg. B. Bendell; 1 male, Pahang, Frazer’s Hill, 27.X-3.XI.1977, leg. B. Bendell. THAILAND: 1 female, Loei, T1108, Phu Ruea NP, 17°29.652'N, 101°21.020'E, 1167 m., 5-6.xi.2006, pan trap, leg. Patikhom Tumtip, coll. M. Hauser; 1 female, Phetchabun, Nam Nao NP, Heliport, 16°43.156"N, 101°35.118"E, 890 m., 18-25.xii.2006, leg. Noopean Hongyothi, coll. RMNH. VIETNAM: 1 female, Chu Yang Sin Nat. Park, 1-10.VI.2007, mal. trap, leg. C. van Achterberg & R. de Vries; DNA voucher labcode MZH:Y1072, in coll. RMNH.

#### Description (based on holotype).

Adult male. Body size: 12 mm (paratype 10 mm).

**Head.** Face occupying about 1/3 of head width in frontal view; black, except brownish yellow on lateral 1/6; entirely white pilose. Gena black; white pilose. Lateral oral margins weakly produced. Frons and vertex black; white pilose. Occiput black; white pilose. Eye pilose, with pile approximately as long as diameter of ocelli. Antennal fossa about as high as wide. Antenna brown; antennal ratio approximately as 3.5:1:2.

**Thorax.** Mesoscutum black; yellow pilose, with pile thicker and more appressed along anterior margin, along transverse suture and along posterior margin, forming three transverse fasciae. Postpronotum and postalar callus brown; yellow pilose. Scutellum semicircular; with pair of distinct apical calcars with mutual distance about 1/4 of width of scutellum at base; brown; yellow pilose. Pleurae shining black; all pilosity yellowish white. Anepisternum pilose, except anterior part bare ventromedially; with deep sulcus separating anterior from posterior part. Anepimeron entirely pilose. Katepisternum pilose dorsally, bare ventrally. Katatergum long microtrichose, anatergum short microtrichose. Calypter and halter pale yellow.

*Wing*: Hyaline, except infuscated around apical crossveins, around spur on vein R4+5 and around base of R2+3, crossvein r-m and bm-cu; microtrichose, except bare on cell bc, posterobasal 1/10 of cell c, basally on cell r1 along vein Rs, basal 3/4 of cell br, anterobasal 1/5 of cell cup, basomedian 2/3 of alula.

*Legs*: Brownish yellow, with tibiae slightly infuscated medially; entirely yellow pilose. Coxae blackish brown; yellow pilose. Trochanters yellow; yellow pilose.

**Abdomen.** Tergites dark brown, except tergites 2-3 yellow laterally. Tergite 1 yellowish pilose. Tergite 2 yellowish pilose, except silvery white pilose along posterior margin. Tergite 3 silvery white pilose along anterior and posterior margins, yellow pilose along lateral margins, black pilose medially. Tergite 4 silvery white pilose along anterior margin and on posterior 1/2, black pilose medially. Sternites brown. Sternite 1 and 2 white pilose, sternite 3 and 4 black pilose. Male genitalia as in Fig. 95.

**Female.** As male, except for following differences. Body size 8–12 mm. Overall colouration paler: whereas pale parts are brownish in the examined males, these parts are yellowish in the examined females. The scutellar spines are less strongly developed, and in one of the examined females from Thailand even totally absent.

#### Etymology.

This species is named after my son Tiber. The epithet is a noun in apposition.

#### Notes.

In the paratype and in all additionally studied specimens, the pilosity of thorax and abdomen is more golden yellow than in the holotype, also in the parts which are silvery white in the holotype. In most specimens the legs are entirely yellow, without infuscated parts.

The paratype has a label stating “*Microdon fascipennis* Sack” (or possibly *fuscipennis*) in what seems to be the handwriting of J.C.H. De Meijere (judged by comparison with figures of de Meijere’s handwriting in De Jong 2000). However, no such name is known to have been given to any Microdontinae, neither by Sack nor by any other author. Either De Meijere was mistaken, or the name is an unpublished manuscript name.

### Indascia gigantica

Reemer sp. n.

urn:lsid:zoobank.org:act:F26FF6CC-E819-4A7B-AFA3-9E7DB82C004B

http://species-id.net/wiki/Indascia_gigantica

#### Type specimens.

HOLOTYPE. Adult male. THAILAND. Label 1: “THAILAND: Chiang Mai, Doi Inthanon NP / Checkpoint 2, 18°31.554'N, 98°29.94'E 1700 m / Malaise trap 8-15.v.2007, Y. Areeluck leg. T1832”; label 2: “Syrphidae / T1832 / W. Porras, 08”; label 3: “Voucher code M. Reemer / 319 / DNA voucher labcode MZH:Y0909”. Coll. QSBG.

#### Diagnosis.

Within *Indascia*, this exceptionally large species shares the presence of a posterior appendix on vein R4+5 only with *Indascia spathulata* sp. n. From that species, *Indascia gigantica* differs by tergite 2 being about 1.5 times as long as wide, and the basoflagellomere being 2 times as long as wide.

#### Description (based on holotype).

Adult male. Body size: 9.5 mm.

**Head.** Face occupying about 1/4 of head width in frontal view; black; entirely silvery white pilose. Gena black, white pilose. Oral margin not produced. Frons and vertex black; golden pilose, except for few black pile at ocellar triangle. Occiput black; yellowish pilose dorsally, white pilose ventrally. Eye bare. Antennal fossa about as high as wide. Antenna black; antennal ratio approximately as 4:1:4.

**Thorax.** Thorax black, except postalar callus and metanotum yellowish and posterior pleurites narrowly brownish along margins. Mesoscutum mixed golden and black pilose, with white pile at and around notopleuron. Postpronotum whitish pilose. Postalar callus black pilose anteriorly, yellow pilose posteriorly. Scutellum somewhat triangular, without calcars; black pilose dorsally, golden pilose along lateral and posterior margins. Anepisternum with deep sulcus separating anterior and posterior part; entirely long white pilose. Anepimeron entirely long white pilose. Katepisternum long white pilose dorsally, bare ventrally. Katatergum long microtrichose, anatergum short microtrichose. Calypter greyish. Halter pale yellow.

*Wing*: Hyaline, subtly darkened around apical crossveins and appendix of vein R4+5; microtrichose, except bare on cell sc, posterobasal 2/3 of cell c, basal 1/4 of cell r1, most of cell br except microtrichose along vena spuria, basal 5/6 of cell bm and basal 1/2 of cell cup.

*Legs*: Mid femur blackish, gradually turning yellow at apical 1/4; black pilose. Mid tibia yellow at basal 1/2, blackish at apical 1/2; black pilose. [Mid tarsus and other legs missing in holotype.] Coxae and trochanters black.

**Abdomen.** Tergites bronze-black. Tergite 1 long white pilose laterally, short black pilose sublaterally, bare medially. Tergite 2 with long white pile laterally on anterior 1/2, short black pilose over dorsal surface, short golden pilose narrowly along posterior margin. Tergite 3 long white pilose laterally on anterior 1/2, short black pilose over dorsal surface, long golden pilose on posterior 1/3. Tergite 4 with pilosity more or less as tergite 3, but much more sparse. Sternites blackish brown; sternite 1 bare; sternites 2-4 short black pilose anteriorly, long white pilose posteriorly. Male genitalia as in Fig. 112.

**Female.** Unknown.

#### Etymology.

The specific epithet (adjective) refers to the large size of this species in comparison with other known species of *Indascia*.

#### Notes.

In the holotype, the only specimen available, the posterior appendix of vein R4+5 is composed of two short vein stumps, which are confluent at their apices, forming a triangle with part of vein R4+5. This is unusual, although similar aberrations can be found in single specimens of Microdontinae from different genera and species groups. Whether the venation as found in the holotype is representative of this species remains uncertain.

### Indascia spathulata

Reemer sp. n.

urn:lsid:zoobank.org:act:4E7D2BF5-2BD0-4D88-B5D5-D6FCD848DA8D

http://species-id.net/wiki/Indascia_spathulata

#### Type specimens.

HOLOTYPE. Adult male. VIETNAM. Label 1: “C. VIETNAM: Ha Tinh / Vu Quang N.P., 166 m, N 18° / 17'39"E 105°25'27", 24.ix. - / 5.x.2009, Mal. tr. 12, RMNH’09 / C. v. Achterberg & R. de Vries”; label 2: “Voucher code M. Reemer / 285 / DNA voucher labcode MZH:Y1100”. Coll. RMNH.

#### Diagnosis.

Within *Indascia*, this species only shares the presence of an appendix on vein R4+5 with *Indascia gigantica* sp. n. From that species, *Indascia spathulata* differs by tergite 2 being more than twice as long as wide, and the basoflagellomere being 5 times as long as wide.

#### Description (based on holotype).

Adult male. Body size: 6 mm.

**Head.** Face occupying slightly less than 1/3 of head width in frontal view; black; entirely silvery white pilose. Gena black, white pilose. Oral margin not produced. Frons and vertex black; yellowish white pilose, except for few black pile at ocellar triangle. Occiput black; black pilose dorsally, yellowish pilose laterally and ventrally. Eye bare. Antennal fossa about as high as wide. Antenna black; basoflagellomere with dorsal margin somewhat concave; antennal ratio approximately as 5:1:9.

**Thorax.** Thorax black, except postalar callus and postpronotum yellowish brown and ventral pleurae brown. Mesoscutum black pilose, except yellow pilose laterally. Postpronotum and postalar callus yellow pilose. Scutellum semicircular, without calcars; yellow pilose. Anepisternum weakly sulcate; dorsal 3/5 yellowish pilose, ventral 2/5 bare. Anepimeron entirely long yellowish white pilose. Katepisternum long white pilose dorsally, bare ventrally. Katatergum long microtrichose, anatergum short microtrichose. Calypter greyish. Halter yellowish white.

*Wing*: Hyaline; microtrichose, except bare on cell sc, basally on cell r1 along vein Rs, basal 1/2 of cell br, basal 3/4 of cell bm, anteriorly on cell cup along vein CuA.

*Legs*: pale yellow, except blackish brown on basal 1/3 of front, basal 3/4 of mid and most of hind femur (except extreme base and apex yellow in the latter), and distal 2/3 of hind tibia; yellow pilose, except black pilose on 4th and 5th tarsomere of front and mid leg, and on dark parts of hind femur and tibia. Coxae blackish brown; yellow pilose. Trochanters yellow; yellow pilose.

**Abdomen.** Tergites black, except anterior 1/4 of tergite 3 yellow and narrow anterior margin of tergite 4 yellow. Tergites 1 and 2 white pilose. Tergite 3 white pilose on yellow part, black pilose on black part. Tergite 4 black pilose, except white pilose in anterolateral corners and along posterior margin. Sternites black, except sternite 3 yellow on anterior 1/4. Sternite 1 bare. Sternite 2 white pilose. Sternite 3 white pilose on yellow part and along lateral margins. Sternite 4 black pilose, except white pilose along anterior margin. Male genitalia as in Fig. 118.

**Female.** Unknown.

#### Etymology.

Even more so than its congenerics, this species has a spoon-shaped abdomen, due to the strongly constricted second segment. This character inspired its name: *spathulata* (Latin adjective for ‘spatulate’, spoon-shaped).

### Kryptopyga sulawesiana

Reemer sp. n.

urn:lsid:zoobank.org:act:8F15F979-79AC-4F43-815A-1547719D0808

http://species-id.net/wiki/Kryptopyga_sulawesiana

#### Type specimens.

HOLOTYPE. – Male. Label 1: “INDONESIA; N. Sulaw.; / 20 km N. Bitung: Tang- / koko N.P.; 0–200 m; / 1°N, 125°12 E; 19 / IV 1988; R. Hensen.” Coll. RMNH.

#### Diagnosis.

This species differs from *Kryptopyga pendulosa* by the less modified abdomen: tergite 4 is not perpendicular to tergite 3 and sternite 4 is well visible in ventral view.

#### Description (based on holotype).

Adult male. Body size: 14 mm.

**Head.** Face occupying about 2/5 of head width in frontal view; black on median 1/2, pale brown on lateral 1/4; entirely long appressed yellowish pilose, golden on ventral half. Gena widely developed; blackish; long yellow pilose. Oral margin anteriorly notched, laterally produced. Frons black; short golden pilose. Vertex strongly swollen; black; short golden pilose anteriorly, long black pilose posterior to ocellar triangle. Ocellar triangle not elevated. Occiput strongly swollen dorsally, narrow laterally; black; black pilose dorsally, golden pilose ventrally. Eye bare. Antennal fossa about as high as wide. Antenna blackish brown, scape a little paler basally; ratio of lengths of scape and basoflagellomere approximately as 1:4, pedicel very short; basoflagellomere very long (4 mm), parallel-sided, with very long black pilosity, about 1,5 times as long as width of basoflagellomere. Arista absent.

**Thorax.** Mesoscutum black; black pilose, except for some golden pile along transverse suture and along lateral margins. Postpronotum and postalar callus brown, black pilose. Scutellum yellow; black pilose. Pleuron blackish brown. Anepisternum with deep sulcus separating posterior from anterior part; mixed yellow and black pilose anteriorly, black pilose posterodorsally, yellow pilose posteriorly. Anepimeron entirely long yellow pilose. Katepisternum long pale yellow pilose dorsally and ventrally. Katatergum long microtrichose, anatergum short microtrichose. Calypter greyish yellow. Halter yellow.

*Wing*: hyaline, slightly darkened anteroapically; microtrichose, except bare on 1st and cell c, basal 1/3 of cell r1, basal 1/4 of cell r2, basal 1/2 of cell r4+5, basal 1/2 of cell dm, entirely on cells R and BM, entirely on cell br and BM, anterobasally on cell dm, most of cell cup and most of alula (only microtrichose along margins).

*Legs*: Brown, more blackish on femora and fore- and mid-tibiae; femora pale pilose anteriorly, black pilose posteriorly; tibiae and tarsi yellow pilose.

**Abdomen.** Elongate, more or less oval, with widest point at posterior margin of tergite 2; high in lateral view; tergites 3 and 4 not fused, with posterior margin of tergite 3 strongly overlapping tergite 4. Tergites blackish, except terigte 1 yellowish brown and other tergites narrowly yellowish brown along margins; short black pilose, except longer yellowish pilose along lateral margins of all tergites and posterolateral margins of tergites 3-4. Sternite 1 blackish; bare. Sternites 2-4 dark brown on anterior 2/3, yellow on posterior 1/3; entirely long yellow pilose. Genitalia as in Fig. 131.

**Female.** Unknown**.**

#### Etymology.

The specific epithet (noun in the genitive case) is derived from the Indonesian island Sulawesi, the type locality.

### Masarygus palmipalpus

Reemer sp. n.

urn:lsid:zoobank.org:act:596FD7FE-BBBE-4990-B2C9-4658FFBF0C05

http://species-id.net/wiki/Masarygus_palmipalpus

#### Type specimens.

HOLOTYPE. Adult male. PERU. Label 1: “PERU. Madre de Dios, Rio / Tambopata, Sachavacayoc Centre / 12°51'S–69°22'W. Mal. trap / 28–30.X.2008. Leg. J.T. Smit”. Coll. RMNH (preliminary deposition, to be transferred to relevant Peruvian collection later).

#### Diagnosis.

This is the only known species of Microdontinae in which the antenna of the male is furcate into five branches.

#### Description (based on holotype).

Adult male. Body size: 4 mm.

**Head.** Head unusually flat. Face wide: occupying about 3/4 of head width in frontal view; somewhat concave laterally; yellow; yellow pilose, except black pilose laterally on dorsal 1/2. Gena yellow; yellow pilose. Oral margin not produced; oral opening barely visible; mouth parts undeveloped. Frons brown; black pilose; very short; distance between frontal ocellus and antennal fossa shorter than height of antennal fossa. Vertex blackish brown medially, yellow laterally; black pilose; ocelli arranged almost in a straight line, with frontal ocellus weakly developed, much smaller than the other two. Occiput yellow; black pilose dorsally, yellow pilose ventrally. Eye bare. Antennal fossa about 1,5 times as wide as high. Antenna black; black pilose; ratio of scape:basoflagellomere approximately as 1:8; pedicel very short. Basoflagellomere furcate into five branches, four of which about equally long, the fifth branches off from one of the other at about ¼ from the base of the segment, with a length of about 2/5 of the other branches. Arista absent.

**Thorax.** Mesoscutum black, except narrowly pale yellow along margins; black pilose. Postpronotum pale yellow; bare. Postalar callus pale yellow; black pilose. Scutellum black; black pilose; semicircular; without calcars; flat, appearing even slightly concave; smooth and shining along margins, dull dorsally due to micropunctation; black pilose. Anepisternum pale yellow along dorsal margin, brown otherwise; with sparse long black pile, also ventrally; without sulcus. Other pleurae yellowish to brown; bare (also without microtrichia). Calypter pale yellow. Halter pale yellow with greyish margin.

*Wing*: Hyaline; microtrichose, except bare on cell sc and basal 1/4 of cell cup.

*Legs*: Front and mid leg pale yellow, except dark brown on basal 3/4; black pilose. Hind leg dark brown, except fifth tarsomere yellow; black pilose. Front coxa exceptionally long: about 4/5 of length of femur, longer than tibia; pale brown; bare. Other coxae and trochanters shorter; pale brown; very sparsely black pilose.

**Abdomen.** Strongly flattened dorsoventrally. Tergite 1 blackish; black pilose; medially interrupted by the whitish antetergite, which is almost entirely fused with the tergite. Tergites 2 and 3 whitish, except black on lateral 1/5, the black part most narrow at posterior margin; black pilose. Tergite 4 black, except for a pair of whitish, submedian, oval maculae at posterior 1/2. Sternite 1 whitish; bare. Sternite 2 whitish; yellow pilose. Sternite 3 whitish, except for lateral dark brown, round macula at anterior 1/2, of about 1/4 of tergite width; yellow pilose, except black pilose anteromedially. Sternite 4 whitish, except for pair of dark brown, oval maculae, almost confluent medially; black pilose anteriorly, yellow pilose posteriorly. Male genitalia as in Fig. 146.

**Female.** Unknown.

#### Etymology.

The specific epithet (noun in apposition) is composed of the Latin words *palma* (hand) and *palpus* (feeler, here interpreted as antenna). The name refers to the hand-like antenna of the male of this species.

### Mermerizon inbio

Reemer sp. n.

urn:lsid:zoobank.org:act:5282F07F-EAD6-4A3D-92BD-18A5C32AA2E0

http://species-id.net/wiki/Mermerizon_inbio

#### Type specimen.

HOLOTYPE. COSTA RICA. Male. Label 1: “COSTA RICA. Prov. Guanacaste, P.N. / Rincón de la Vieja, Send. a las aguas / termales, 900–1000 m, 6–7 OCT / 2001. D. Briceño, Red con Aguamiel. / L_N_305843_392970 #64950”; label 2: “INB0003380896 / INBIOCRI COSTA RICA”; label 3 (red): “Ultimo especimen en / BD A. Lépiz / 2-7-2002” / other side: “?MCR-25”. Coll. INBIO.

#### Diagnosis.

Distinguished from the other two known (yet undescribed) species of *Mermerizon* by the black pilose mesoscutum.

#### Description (based on holotype).

Adult female. Body size: 7,5 mm.

**Head.** Face occupying about 1/4 of head width in frontal view; yellow; yellow pilose, with narrow bare median line on dorsal half. Gena yellow. Frons black; yellow pilose laterally, black pilose posteriorly. Vertex dark yellow, except black at and around ocellar triangle; black pilose. Occiput black, except yellow posteriad of vertex; black pilose on dorsal half, yellow pilose on ventral half. Eye bare. Antennal fossa about as high as wide. Antenna with scape dark brown, pedicel and basoflagellomere yellowish brown; antennal ratio approximately as 4:1:4.

**Thorax.** Scutum blackish brown, except yellow on notopleuron and around postpronotum and postalar callus; black pilose. Postpronotum, postalar callus and scutellum yellow; black pilose. Scutellum semicircular, without calcars, Anepisternum blackish brown; convex, without sulcus; black pilose on anterior part and along posterior margin, widely bare in between. Anepimeron brown; black pilose on dorsal 1/4. Katepisternum yellow dorsally, brown ventrally; bare. Katepimeron yellow. Katatergum long microtrichose. Anatergum short pale microtrichose. Calypter blackish. Halter yellowish brown.

*Wing*: hyaline; microtrichose, except bare on cell bc, basal 1/4 of cell br, basal 1/3 of cell bm, anterobasal 1/4 of cell cup.

*Legs*: Front and mid legs yellowish brown; black pilose. Hind leg blackish brown, except basal 1/2 of tibia and apical four tarsomeres yellowish brown. Front and mid coxae and trochanters yellowish brown; yellow pilose apically. Hind coxa and trochanter dark brown; black pilose.

**Abdomen.** Tergites and sternites yellowish; yellow pilose, except sternite 1 bare. Genitalia as in Fig. 162.

**Female.** Unknown.

#### Etymology.

The specific epithet (noun in apposition) is based on InBio, an acronym of Instituto Nacional de Biodiversidad, the Costa Rican institute which holds the holotype of this species.

### Metadon achterbergi

Reemer sp. n.

urn:lsid:zoobank.org:act:5468A969-1FE2-4D32-969E-B878838546C0

http://species-id.net/wiki/Metadon_achterbergi

#### Type specimens.

HOLOTYPE. Adult female. VIETNAM. Label 1: “C. VIETNAM: Ha Tinh / Vu Quang N.P., 53 m, N 18° / 20'50" E105°26'37.8", 22.ix.- / 6.x.2009, Mal. trap 1, RMNH-09 / C. v. Achterberg & R. de Vries”. Coll. RMNH. Voucher code M. Reemer: 284. DNA voucher labcode MZH:Y1086.

#### Diagnosis.

Within *Metadon*, five other described Oriental species have a dark (sub)apical wingspot. These species are listed here (in parentheses a character is given that distinguishes them from *Metadon achterbergi* sp. n.): *Microdon auricinctus* Brunetti, 1908 (tergite 4 red); *Metadon bicoloratus* Hull, 1944 (thorax and abdomen without fasciae of golden pile); *Metadon fuscicornis* Sasakawa, 1960 (wing infuscated at entire apical half); *Metadon pendleburyi* Curran, 1931 (thorax and abdomen without fasciae of golden pile); *Metadon wulpii* Mik, 1899 (mesoscutum without fascia of golden pile along transverse suture, scutellum reddish brown).

#### Description (based on holotype).

Adult female. Body size: 13 mm.

**Head.** Face occupying about 1/3 of head width in frontal view; dark brown; golden pilose, very narrowly bare medially on ventral half. Gena brown, golden pilose. Lateral oral margin produced. Frons, vertex and occiput brown; golden pilose, except occiput ventrally white pilose. Eye bare. Antennal fossa about as high as wide. Antenna black; antennal ratio approximately as 4:1:3.

**Thorax.** Mesoscutum blackish brown; golden pilose, with transverse fasciae of thicker golden pile along anterior margin, transverse suture (fascia medially interrupted) and posterior margin. Postpronotum and postalar callus yellow; golden pilose. Scutellum semicircular; blackish brown; golden pilose. Anepisternum with deep sulcus; yellowish brown; entirely golden pilose. Anepimeron yellowish brown; entirely pilose. Katepisternum blackish brown; golden pilose dorsally, very sparsely pilose ventrally. Other pleurites yellowish brown. Katatergum long microtrichose, anatergum short microtrichose. Calypter and halter yellowish.

*Wing*: Yellow on basal 2/3, blackish on apical 1/3, with colouration in posterior half less conspicuous. Microtrichose, except bare on cell bc, narrowly along vein Rs in cell r1, basal 3/4 of cell br, basomedian 3/4 of cell bm, anterobasal 1/4 of cell cup, and basomedian 9/10 of alula.

*Legs*: Yellow; yellow pilose. Front coxa yellow, mid and hind coxae blackish brown; yellowish pilose. Trochanters blackish brown; yellowish pilose.

**Abdomen.** Tergites black. Tergites 1 and 2 golden pilose. Tergite 3 golden pilose anterolaterally; black pilose otherwise. Tergite 4 with fascia of golden pile along anterior margin, laterally widening and expanding along lateral margin, and with pair of sublateral oblong maculae of golden pile, black pilose in between. Tergite 5 golden pilose anterolaterally, black pilose otherwise. Sternites blackish brown; pale pilose, except sternite 5 mostly black pilose.

**Male.** Unknown.

#### Etymology.

This species is named after its collector, Dr. C. van Achterberg, in acknowledgment of the many ways in which he has been helpful to the senior author during his PhD work. The epithet is a noun in the genitive case.

### Microdon hauseri

Reemer sp. n.

urn:lsid:zoobank.org:act:2B1D3B15-0332-49CA-8B12-F6ED443CDC38

http://species-id.net/wiki/Microdon_hauseri

#### Type specimens.

HOLOTYPE. Adult male. CHINA. Label 1: “Yunnan. Tengchong / 50 km NNW: Houqiao / N25.388° E 98.211° / 1700 m / 01.VI.2009 leg. / Blank, Liston, Taeger / 008 China”; label 2: “Voucher code M. Reemer / 302 / DNA voucher labcode MZH:Y1096”. Coll. CSCS.

#### Diagnosis.

In the keys of Shiraki (1968), Huo et al. (2007) and – depending on how characters are interpreted – Hironaga and Maruyama (2004), this species keys to *Microdon auricomus* Coquillet, 1898, from which it differs by the largely orange legs and the long, orange-golden pilosity on the anterodorsal part of the hind femur. These characters also apply for distinguishing *Microdon hauseri* sp. n. from *Microdon murayamai* Hironaga & Maruyama, 2004, to which specimens of the species will key in the key of Hironaga and Maruyama (2004). The same characters apply for separating it from *Microdon lateus* Violovitsh, 1975, to which it keys using Violovitsh (1983). In the key of Shiraki (1930) this species keys to *Microdon formosanus* Shiraki, 1930, from which it differs by the black pilosity medially on the mesoscutum (entirely pale in *Microdon formosanus*).

#### Description (based on holotype).

Adult male. Body size: 12.5 mm.

**Head.** Face occupying about 1/3 of head width in frontal view; black; entirely yellowish pilose. Gena black, yellowish pilose. Oral margin not produced. Frons black; black pilose, except narrowly yellow pilose along lateral and posterior margins. Vertex black; black pilose, except narrowly yellow pilose along all margins. Occiput black; yellow pilose. Eye bare. Antennal fossa about as high as wide. Antenna black; antennal ratio approximately as 3.5:1:2.5.

**Thorax.** Entire thorax blackish with bronze hues. Mesoscutum black pilose medially, widely yellow pilose along margins. Postpronotum, postalar callus and scutellum yellow pilose. Scutellum trapezoid with slightly concave posterior margin; without calcars. Anepisternum yellow pilose anteriorly, mixed black and yellow pilose posteriorly, with widely bare part in between; with shallow sulcus separating anterior from posterior part. Anepimeron entirely yellow pilose. Katepisternum yellow pilose dorsally, very sparsely yellow pilose ventrally. Katatergum long microtrichose, anatergum short microtrichose. Calypter and halter pale yellow.

*Wing*: Hyaline, subtly darkened around apical crossveins; microtrichose, except bare on basal 3/5 of cell br and basomedian 1/3 of alula.

*Legs*: Orange, except basal 1/4 of femora blackish, apex of femora narrowly darkened and tibiae dorsally darkened. Front femur black pilose, except for patch of orange-golden pile anterobasally; mid femur orange-golden pilose anteriorly and posteriorly on basal 2/3, with patch of orange-golden pile anteroventrally on basal 1/4, black pilose dorsally and ventrally; hind femur with long orange-golden pile anterodorsally and posteriorly, with orange-golden pile on basal 1/3, black pilose otherwise. Front and mid tibia orange-golden pilose, except black pilose dorsally. Hind tibia orange-golden pilose (long dorsally, short ventrally), except black pilose laterally. Tarsi black pilose. Coxae and trochanters black; pale pilose.

**Abdomen.** Tergites black with bronze hues. Tergites 1 and 2 golden pilose. Tergite 3 golden pilose on lateral 1/4, orange-golden pilose medially (colour transition gradual). Tergite 4 orange-golden pilose, except for pair of submedian patches of black pile on anterior 1/2; each about as wide as 1/4 of the tergite. Sternites black with bronze hues; entirely whitish to golden pilose. Male genitalia as in Fig. 212.

**Female.** Unknown.

#### Etymology.

This species is named after Martin Hauser, in acknowledgement for the many interesting specimens of Microdontinae he sent to the author. The epithet is a noun in the genitive case.

### Microdon mandarinus

Reemer sp. n.

urn:lsid:zoobank.org:act:B34669D3-D2D9-4CE8-8B96-CBF2A61D1DA3

http://species-id.net/wiki/Microdon_mandarinus

#### Type specimens.

HOLOTYPE. Adult male. CHINA. Label 1: “Yunnan: Deqin / 10 km SW: Meili mts. / N28.423°, E98.868° / 2700 m / 20.VI.2009 leg. / Blank, Liston, Taeger / 048 China”; label 2: “Voucher code M. Reemer / 299 / DNA voucher labcode MZH:Y1093”. Coll. CSCS.

PARATYPE. CHINA. Label 1: “Yunnan: / Deqin 33 km SE / N28.282°, E99.162° / 3200 m / 18.VI.2009 leg. / Blank, Liston, Taeger / 040 China”. Coll. CSCS.

#### Diagnosis.

The orange colouration of large parts of this species’ body, most notably its head, legs and the lateral parts of the tergites, precludes confusion with any other known Palaearctic or Oriental species of *Microdon* s.s.

#### Description (based on holotype).

Adult male. Body size: 11 mm.

**Head.** Face occupying about 1/3 of head width in frontal view; orange yellow; entirely yellow pilose. Gena black, yellow pilose. Oral margin laterally weakly produced. Frons black; yellow pilose. Vertex yellow; yellow pilose. Occiput black; yellow pilose. Eye almost bare, sparse and short pile visible only under high magnification. Antennal fossa about as high as wide. Antenna pale brown; antennal ratio approximately as 3:1:2.5.

**Thorax.** Mesoscutum blackish bronze with green metallic hues, except yellow along lateral margins; entirely yellow pilose. Postpronotum, postalar callus and scutellum yellow; yellow pilose. Scutellum trapezoid with slightly concave posterior margin; with minute, barely discernable posterolateral calcars, their mutual distance about equal to 1/3 of width of scutellum. Pleuron blackish, except anepisternum anterodorsally with small yellow spot and katatergum medially with small yellow spot; all pilosity yellow. Anepisternum with deep sulcus separating anterior from posterior part; pilose anteriorly and posteriorly, with widely bare part in between. Anepimeron entirely pilose. Katepisternum pilose dorsally; bare ventrally. Katatergum long microtrichose, anatergum short microtrichose. Calypter and halter yellowish white.

*Wing*: Hyaline; microtrichose, except bare on posterobasal 1/4 of cell br.

*Legs*: Yellow, except narrowly blackish around basal cicatrix on femora; yellow pilose. Coxae and trochanters yellow, except hind coxa black on basal half; yellow pilose.

**Abdomen.** Tergite 1 black; yellow pilose. Tergite 2 black, except orange yellow on lateral 1/6; erect yellow pilose , except for fascia of appressed golden pile along posterior margin. Tergite 3 medially with semicircular black mark, anteriorly as wide as the black part on tergite 2, posteriorly narrow and just reaching posterior margin; laterally orange yellow; short black pilose on most of anterior half, except yellow pilose along lateral margins, with fascia of appressed golden pile along posterior margin. Tergite 4 largely orange yellow, except for vaguely defined blackish mark anteromedially; largely short yellow pilose, except for anterolateral patches of black pile. Sternite 1 black; yellow pilose. Sternite 2 and 3 yellow, except blackish near lateral margins; yellow pilose. Sternite 4 yellow; yellow pilose. Male genitalia as in Fig. 213.

**Female.** As male, except for the following differences. Body size: 14 mm. Frons largely yellow, except for small triangular black area posteriad of lunula. Antenna: scape and pedicel yellowish. Mesoscutum with pair of small submedian yellow spots at posterior margin. Scutellum without any sign of calcars. Anepimeron, dorsal part of katepisternum, katepimeron, katatergum and anatergum yellow. Tergite 4 with fascia of appressed golden pile on posterior half. Tergite 5 largely orange yellow, except blackish anteromedially; entirely appressed golden pilose.

#### Etymology.

The species name (adjective) refers to ‘mandarin’, which has a number of meanings. It’s an orange citrus fruit, it’s the most spoken language in China, and it used to be a high governmental function in imperial China. The name is considered appropriate for this species because of the characteristic orange colour of several body parts and the Chinese origin of the type material.

### Microdon yunnanensis

Reemer sp. n.

urn:lsid:zoobank.org:act:9A81751D-97B5-445A-9F78-9A5E5067E394

http://species-id.net/wiki/Microdon_yunnanensis

#### Type specimens.

HOLOTYPE. Adult male. CHINA. Label 1: “Yunnan: Tengchong / 25 km NNW / N25.189°, E98.333° / 1900 m. / 01.VI.2009 leg. / Blank, Liston, Taeger / China 010”; label 2: “ Voucher code M. Reemer / 301 / DNA voucher labcode MZH:Y1095”. Coll. CSCS.

#### Diagnosis.

This species keys to *Microdon japonicus* Yano, 1915 in the keys of Huo et al. (2007) and Shiraki (1930, 1968). From that species it is distinguished by the entirely yellow pilose mesoscutum (with patches of black pile in *Microdon japonicus*). In the key of Hironaga and Maruyama (2004) it keys to *Microdon kidai* Hironaga & Maruyama, 2004, from which it differs by its partly yellow legs (entirely black in M. *kidai*). In the key of Violovitsh (1983) this species keys to *Microdon eggeri* Mik, 1897 (= *Microdon analis* (Macquart, 1842)), from which it differs by its pale brown scutellum (black in *Microdon analis*) and the shape of tergite 2, which is at its widest clearly before the posterior margin (widest at posterior margin in *Microdon analis*).

#### Description (based on holotype).

Adult male. Body size: 11 mm.

**Head.** Face occupying a little less than 1/2 of head width in frontal view; black; entirely golden yellow pilose. Gena black, golden yellow pilose. Oral margin not produced. Frons, vertex and occiput black; golden yellow pilose. Eye bare. Antennal fossa about as high as wide. Antenna black; antennal ratio approximately as 2.5:1:1.5.

**Thorax.** Entire thorax blackish with bronze hues, except scutellum brownish; all pilosity yellow. Scutellum trapezoid with slightly concave posterior margin; with slender calcars as long as 1/5 of length of scutellum, their mutual distance about equal to 1/3 of width of scutellum. Anepisternum with shallow sulcus separating anterior from posterior part; pilose anteriorly and posteriorly, with widely bare part in between. Anepimeron entirely pilose. Katepisternum pilose dorsally and ventrally. Katatergum long microtrichose, anatergum short microtrichose. Calypter and halter yellowish white.

*Wing*: Hyaline, subtly darkened around apical crossveins; microtrichose, except bare on basal 1/2 of cell br.

*Legs*: Black, except basal 3/5 of tibiae and ventral side of tarsi yellow; yellow pilose. Coxae and trochanters black; yellow pilose.

**Abdomen.** Tergites black. Tergites 1 and 2 yellow pilose. Tergite 3 black pilose, except narrowly whitish pilose along lateral and posterior margins. Tergite 4 black pilose, except narrowly whitish pilose along lateral margins and whitish pilose on posterior 1/3. Sternites black; whitish pilose. Male genitalia as in Fig. 214.

**Female.** Unknown.

#### Etymology.

This species is named after the Chinese province of Yunnan, in which it was found. The epithet is a noun in the genitive case.

### Paramixogaster piptotus

Reemer sp. n.

urn:lsid:zoobank.org:act:C4B02C42-9193-4281-AB2D-13194B38A444

http://species-id.net/wiki/Paramixogaster_piptotus

#### Type specimens.

HOLOTYPE. Female. Label 1: “Madagascar / Bekily / Reg sud de l’ile”; label 2 (blue): “Museum Paris / I.37 / A. Seyrig”; label 3: “Ceratrichomyia / behara type du genre [female sign] Séguy 1950”. Coll. MNHN. [this specimen is one of the paralectotypes of *Ceratrichomyia behara*; see redescription of that species above]

#### Diagnosis.

This is the only known species of *Paramixogaster* with a pair of submedian vittae of golden pilosity on the posterior half of the mesoscutum.

#### Description (based on holotype).

Adult female. Body size: 7 mm.

**Head.** Face occupying about 3/5 of head width in frontal view; yellow; entirely yellow pilose. Gena yellow. Lateral oral margins very slightly produced. Frons and vertex yellow; yellow pilose. Occiput yellow; dorsally wide and yellow pilose, ventrally narrow and whitish pilose. Eye bare. Antennal fossa about as wide as high. Antenna orange; antennal ratio approximately as 1:0,25:6. Basflagellomere elongate; with sensory pit at apical 1/7. Arista yellow, about 2/5 of length of basoflagellomere.

**Thorax.** Postpronotum yellow; bare. Mesoscutum reddish brown; short yellow pilose, with lateral fasciae of dense golden pile along transverse suture and with two vittae of dense golden pile on posterior half. Postalar callus and scutellum reddish brown; short yellow pilose. Scutellum without calcars. Pleuron reddish brown. Anepisternum with deep sulcus separating anterior and posterior part; white pilose, except with golden pilosity along posterior margin, as an extension of the golden fascia from the mesonotal transverse suture. Anepimeron entirely white pilose. Katepisternum white pilose dorsally; bare ventrally. Katatergum long microtrichose, anatergum short microtrichose. Other pleurites bare. Calypter and halter yellow.

*Wing*: hyaline; microtrichose, except bare on cell bc, basal 1/2 of cell c, basally on cell r1 along vein Rs, almost entirely on on cells R, BM andl CuP, on alula except along margins.

*Legs*: Yellow, except: front femur brownish on basal half, middle and hind femur dark brown on basal 4/5. Legs entirely pale pilose. Coxae and trochanters brown; pale pilose.

**Abdomen.** Constricted at 2nd segment, narrowest at anterior margin of tergite 2, widest at tergite 3 (slightly wider than thorax). Ratio of tergal lengths approximately as 1:4:3:3:3. Tergite 1 dark brown; pale pilose. Tergite 2 dorsoventrally flattened, dark brown with large, oblique yellow maculae over entire length, which are interconnected anteriorly, leaving anterolateral corners and a large posteriomedian triangle dark brown; yellow pilose. Tergite 3 and 4 dark brown and short yellow pilose; with fasciae of golden pile along posterior margins; tergite 4 also with posterolateral margins yellow in ground colour. Tergite 5 brown with posterior 2/5 and median part yellow; yellow pilose. Sternite 1 dark brown; bare. Sternite 2 yellow; yellow pilose. Other sternites brown; yellow pilose.

**Male.** Unknown.

#### Etymology.

The specific epithet (adjective) is derived from the Greek word *piptotos* (that which has fallen). This name refers to the fact that this species ‘fell’ out of the genus *Ceratrichomyia*, for the holotype is also part of the paratype series of *Ceratrichomyia behara* Séguy, 1951.

#### Notes.

This description is based on the female paratype of *Ceratrichomyia behara*. For discussion see genus account of *Ceratrichomyia*.

### Piruwa phaecada

Reemer sp. n.

urn:lsid:zoobank.org:act:F75F1363-5403-4D38-A7C6-97B79488059A

http://species-id.net/wiki/Piruwa_phaecada

#### Type specimens.

HOLOTYPE. Adult male. PERU. Label 1: “PERU. Madre de Dios, Rio / Tambopata, Sachavacayoc Centre / 12°51'S, 69°22'W. Mal. trap / 4-10.IX.2009. Leg. J.T. Smit”; label 2: “Voucher code M. Reemer / 287”. Coll. RMNH (preliminary deposition, to be transferred to relevant Peruvian collection later).

PARATYPES. 2 adult females with label data as holotype, but collection dates are 28–30.X.2008 and VIII.2009. Coll. RMNH & J.T. Smit.

#### Diagnosis.

See generic key and genus account of *Piruwa* (only one species known).

#### Description (based on holotype).

Adult male. Body size: 4 mm.

**Head.** Face occupying about 1/6 of head width in frontal view; black; entirely white pilose. Gena hardly developed; black, white pilose. Oral margin not produced. Frons, vertex and occiput black; white pilose. Eye bare. Antennal fossa about as high as wide. Antenna: scape black, pedicel and basoflagellomere pale brown; antennal ratio approximately as 2.5:1:3.

**Thorax.** Black. Mesoscutum short appressed yellowish pilose, except for sparse bristly black pile anterolaterally. Postpronotum bare. Postalar callus black pilose dorsally, pale pilose laterally. Scutellum semicircular; without calcars; black; short pale pilose and with a few long, bristly, pale setae along posterior margin. Anepisternum convex, without sulcus; pale pilose on dorsal half. Katatergum and anatergum short pilose. All other pleurites bare. Calypter grey. Halter yellow with blackish knob.

*Wing*: Hyaline. Microtrichose, except bare cell bc, basal 1/2 of cell br and cell bm, basal 1/3 of cell cup.

*Legs*: Tibiae and femora black. Tarsi whitish yellow, except first tarsomere of hind leg black. Legs black pilose, except tarsi of front and mid leg dorsally yellow pilose. Coxae and trochanters blackish brown. Coxae white pilose apically. Trochanters bare.

**Abdomen.** Tergites black. Tergites yellowish pilose, except tergite 4 laterally and posteriorly mixed black and yellow pilose. Sternites blackish brown; sternite 1-2 bare; sternites 3-4 short black pilose. Genitalia as in Fig. 304.

**Female.** As male, except for following differences (based on paratype collected VIII.2009). Face golden yellow pilose. Mesoscutum and scutellum mixed golden yellow and black pilose. Pleuron partly brownish. Anepisternum black pilose dorsally. Anepimeron with bristly black pile along dorsal margin. Coxae apically black pilose. Sternite 5 blackish; short black pilose, withlong, bristly black pile along posterior margin. The other female paratype is apparently a teneral specimen, as parts of its body are yellowish brown.

#### Etymology.

The specific epithet *phaecada* (adjective) is derived from the Greek word *phaikas*, which is a kind of white shoe. The name refers to the whitish yellow tarsi of the species that contrast with the entirely black rest of the body.

### Pseudomicrodon polistoides

Reemer sp. n.

urn:lsid:zoobank.org:act:BFB07B79-EE6E-4391-AB42-AB3F970AEA5B

http://species-id.net/wiki/Pseudomicrodon_polistoides

#### Type specimens.

HOLOTYPE. Adult female. PERU. Label 1: “PERU. Madre de Dios, Tambopata / Sachavacayoc Centre, Quebrada / trail. S12°51'20.1"–W69°22'20.1". / Alt. 166 m. Malaise trap / 14–25.VI.2010. Leg. J.T. Smit”. Label 2: “Voucher code M. Reemer / 346 / DNA voucher labcode MZH:Y1319”. Coll. RMNH (preliminary deposition, to be transferred to relevant Peruvian collection later).

#### Diagnosis.

In three other described *Pseudomicrodon* speciesthe alula is completely microtrichose: *Pseudomicrodon chrysostypus* (Thompson, 2004), *Pseudomicrodon pilosops* (Marinoni, 2004) and *Pseudomicrodon smiti* sp. n. From these species, *Pseudomicrodon polistoides* sp. n. differs by the entirely orange coloured abdomen, as well as by the yellow median vitta on the mesoscutum.

#### Description (based on holotype).

Adult female. Body size: 12.5 mm.

**Head.** Face occupying approximately 1/3 of head width in frontal view; yellow; yellow pilose on lateral 1/3, black pilose on median 1/3. Gena yellow; yellow pilose. Lateral oral margins weakly produced. Frons yellow, except for black markings directly laterad of antennal fossa; yellow pilose, except for sparse black pile at black markings. Vertex yellow, except for black markings at and around ocellar triangle and posterolaterally; bare on anterior 1/3, black pilose on posterior 2/3. Occiput yellow, except black adjacent to black markings on vertex; yellow pilose, except black pilose directly posteriad of vertex. Eye almost bare, with very sparse and short white pile. Antennal fossa about as wide as high. Antenna orange yellow, scape a little darker; antennal ratio approximately as 5:1:6; longer than distance between antennal fossa and anterior oral margin.

**Thorax.** Mesoscutum black with widely yellow margins and wide median yellow vitta over entire length, also narrowly yellow along transverse suture. Black pilose, except for fasciae of orange golden pile along anterior margin, transverse suture and posterior margin, as well as along posterolateral margin. Postpronotum and postalar callus yellow; black pilose. Scutellum semicircular; yellow; black pilose, except sparsely golden pilose anterolaterally; without calcars. Pleuron yellow, except dorsomedial and posterior parts of anepisternum partly blackish, and anatergum and lateral margins of mediotergite blackish. Anepisternum sulcate; mixed orange and black pilose anterodorsally, black pilose posteriorly, widely bare in between. Anepimeron entirely yellow pilose. Katepisternum yellow pilose dorsally, bare ventrally. Katatergum long microtrichose, anatergum short microtrichose. Calypter and halter yellowish white.

*Wing*: Yellowish to brown in cells bc and c, cell br, R1, R2+3, anteriorly in cell r4+5, hyaline in other parts. Microtrichose, except bare on cell bc, posterobasal 1/4 of cell br, posterobasal 1/4 of cell bm and basal 1/10 of cell cup.

*Legs*: Yellow; yellow pilose, except femora posteriorly black pilose and hind tarsus dorsally black pilose. Front and mid coxae and trochanters yellow; yellow pilose. Hind coxa yellow anteriorly, blackish brown laterally and posteriorly; yellow pilose, except black pilose apically and laterally. Hind trochanter brownish, mixed yellow and black pilose.

**Abdomen.** Constricted, about as wide as thorax, with narrowest point in posterior 3/4 of tergite 2. Tergites orange, except tergite 1 laterally and tergite 2 anterolaterally dark brown and tergites 2 and 3 with posterior margins yellow. Tergite 1 black pilose laterally, yellow pilose medially. Tergites 2-4 black pilose, except yellow pilose posteriorly. Tergite 5 yellow pilose, except black pilose medially. Sternite 1 brownish; bare. Other sternites yellow; yellow pilose.

**Male.** Unknown.

#### Etymology.

The specific epithet (adjective) emphasizes the resemblance of this species to certain Polistinae (Hymenoptera: Vespidae).

### Pseudomicrodon smiti

Reemer sp. n.

urn:lsid:zoobank.org:act:D6CF66F8-DD63-4075-8459-977E3181CB17

http://species-id.net/wiki/Pseudomicrodon_smiti

#### Type specimens.

HOLOTYPE. Adult male. PERU. Label 1: “PERU. Madre de Dios, Tambopata / Sachavacayoc Centre, Bridge / Condonado trail, S 12°51'25.7” - / W 69°22'23.1”. Alt. 184 m. / 5.VI.2010. Leg. J.T. Smit”. Label 2: “Voucher code M. Reemer / 345 / DNA voucher labcode MZH:Y1318”. Coll. RMNH (preliminary deposition, to be transferred to relevant Peruvian collection later).

PARATYPES. A male and a female from same locality as holotype, collected on 10.VI and 8.VI.2010, respectively. Male in coll. J.T. Smit, female in coll. RMNH.

#### Diagnosis.

In three other described *Pseudomicrodon* speciesthe alula is completely microtrichose: *Pseudomicrodon chrysostypus* (Thompson, 2004), *Pseudomicrodon pilosops* (Marinoni, 2004) and *Pseudomicrodon polistoides* sp. n. From these species, *Pseudomicrodon smiti* sp. n. differs by the combination of the black postpronutum and the partly black hind tibia.

#### Description (based on holotype).

Adult male. Body size: 9.5 mm.

**Head.** Face occupying a little more than 1/4 of head width in frontal view; black, except yellow on lateral 1/5 in dorsal 2/3; entirely yellow pilose; medially with vitta of transversely wrinkled texture. Gena black; yellow pilose. Lateral oral margins weakly produced. Frons black; white pilose. Vertex black; bare on anterior half, black pilose on posterior half. Occiput black; golden pilose on dorsal half, silvery white pilose on ventral half. Eye almost bare, with very sparse and short white pile. Antennal fossa about 1.5 times as wide as high. Antenna black; antennal ratio approximately as 2.5:1:3.5; longer than distance between antennal fossa and anterior oral margin.

**Thorax.** Blackish brown with bronze and green metallic hues. Mesoscutum appressed black pilose, except for medially interrupted fasciae of appressed golden pile along anterior margin and transverse suture. Postpronotum and postalar callus black pilose. Scutellum semicircular; black pilose, except golden pilose posterolaterally; with calcars about as long as 1/3 of length of scutellum, with mutual distance approximately 1/4 of scutellar width. Anepisternum sulcate; black pilose anterodorsally, white pilose posteriorly, widely bare in between. Anepimeron entirely silvery white pilose. Katepisternum silvery white pilose dorsally, bare ventrally. Katatergum long microtrichose, anatergum short microtrichose. Calypter and halter yellowish white.

*Wing*: Hyaline, brownish in cells bc, c and sc, and cell r1. Microtrichose, except bare cell bc, posterobasal 3/4 of cell br, basal 2/3 of cell bm and basal 1/6 of cell cup.

*Legs*: Blackish brown, except whitish on basal 1/3 of mid tibia and basal 2/5 of hind tibia, paler brown on apical half of femora. Femora black pilose, except hind femur white pilose posteriorly and anterobasally. Tibiae white pilose, except black pilose ventrally, and mid tibia black pilose on apical 2/5. Tarsi dorsally black pilose. Coxae brown; front and mid coxae black pilose, hind coxa white pilose. Trochanters brown; black pilose.

**Abdomen.** Constricted, narrower than thorax, with narrowest point halfway of tergite 2. Tergites black, except tergite 2 largely occupied by pair of rectangular yellow maculae on basal 3/4. Tergite 1 long yellowish white pilose laterally, black pilose dorsally. Tergite 2 black pilose, except narrowly golden pilose along posterior margin. Tergite 3 black pilose, except white pilose along lateral margin and with medially interrupted fascia of golden pile along posterior margin. Tergite 4 black pilose, except white pilose anterolaterally and along lateral margin, and with pair of submedian vittae of golden pile on posterior 3/4, widening towards apex. Sternite 1 black; bare. Sternite 2 whitish yellow; bare. Sternites 3 and 4 black; black pilose. Male genitalia as in Fig. 320.

**Female.** Body size: 11 mm. As male, except for usual sexual differences. Tergite 5 golden pilose medially, white pilose laterally.

#### Etymology.

This species is named after John T. Smit, who collected this species in Peru, along with several other interesting Microdontinae. The epithet is a noun in the genitive case.

### Rhopalosyrphus (s.s.) ecuadoriensis

Reemer sp. n.

urn:lsid:zoobank.org:act:E823BA6D-86C3-4014-950D-AEBE9A00B943

http://species-id.net/wiki/Rhopalosyrphus_ecuadoriensis

#### Type specimens.

HOLOTYPE. Adult male. ECUADOR. Label 1: “ECUADOR: Orellana Province / Yasuni Research Station, / Trap, Canopy - 27 m / Malaise M7-1, AT934 / 11-18.vii.2008, A. Tishechkin”; label 2: “Voucher code M. Reemer / 294 / DNA voucher labcode MZH:Y1089”. Coll. RMNH )preliminary deposition, to be transferred to relevant Ecuadorian collection later’.

#### Diagnosis.

In the key of Weems et al. (2003) this species keys to *Rhopalosyrphus australis* Thompson, 2003 because tergite 3 is short: a little more than half as long as tergite 2. However, in *Rhopalosyrphus australis* tergite 3 is about 1/3 as long as tergite 2, which places *Rhopalosyrphus ecuadoriensis* somewhat intermediate between *Rhopalosyrphus australis* and the other two known species of *Rhopalosyrphus*, as far as this character is concerned. Other differences with *Rhopalosyrphus australis* are (character state in *Rhopalosyrphus australis* in parentheses): antennal ratio approximately as 5:1:8 (5:1:10), mesoscutum almost entirely black pilose (white pilose), tergite 3 black (yellow).

#### Description (based on holotype).

Adult male. Body size: 9 mm.

**Head.** Face occupying slightly less than 1/3 of head width in frontal view; black, except yellow on lateral 1/4 on dorsal 2/3; golden yellow pilose, most densely at yellow lateral parts. Gena black, yellow pilose. Lateral oral margins produced. Frons black; yellow pilose. Vertex black; yellow pilose, except black pilose posteriad of ocelli. Occiput black; yellow pilose. Eye bare; with narrow, horizontal area frontally at level of antenna with enlarged ommatidia. Antennal fossa about 1.5 times as wide as high. Antenna black; antennal ratio approximately as 5:1:8; longer than distance between antennal fossa and anterior oral margin.

**Thorax.** Black with faint metallic hues. Mesoscutum black pilose, except narrowly white pilose along anterior margin and with small patches of white pile at notopleuron. Postpronotum and postalar callus white pilose. Scutellum semicircular; black pilose dorsally, white pilose along margins; with small apical calcars, with mutual distance about 1/5 of basal width of scutellum. Anepisternum without sulcus; entirely white pilose, except for small patch of black pile posterodorsally. Anepimeron entirely white pilose. Katepisternum white pilose dorsally, bare ventrally. Katepimeron white pilose anteriorly. Katatergum long microtrichose, anatergum short microtrichose. Calypter and halter yellowish white.

*Wing*: Hyaline, except faintly infuscated around spur on vein R4+5, vein dm-cu, r-m and bm-cu. Microtrichose, except bare on cell bc, basal 1/2 of cell c, basally on cell r1 along vein Rs, entirely on cell br, basal 3/4 of cell bm, anterobasal 1/2 of cell cup and basomedian 1/10 of alula.

*Legs*: Front and mid femora blackish brown, except narrowly yellow at apex; white pilose, except for sparse long, black pile posterodorsally. Front and mid tibiae pale yellow basally, dark yellow apically; white pilose, except for sparse black pile postero-apically. Front and mid tarsi yellow; black pilose dorsally, yellow pilose ventrally. Hind femur black; white pilose anteriorly and dorsally, black pilose posteriorly and with dense, bristly to spiny black pile ventrally. Hind tibia pale yellow on basal 3/5; yellow pilose, except black pilose posteriorly at apical 1/4. Hind tarsus brown; black pilose dorsally, yellow pilose ventrally. Coxae and trochanters brown to blackish; white pilose.

**Abdomen.** Constricted, about as wide as thorax, with narrowest point just before posterior margin of tergite 2. Tergites black with bronze hues, except tergite 2 yellow along posterior margin. Tergite 1 white pilose, except white pilose on median 1/4. Tergite 2 white pilose, except black pilose dorsomedially on apical 1/2. Tergite 3 white pilose, except for dorsomedian triangle of black pile over entire length, which is widest at posterior margin; white pile posterolaterally thicker and more conspicuous, thus forming a medially interrupted fascia at posterior margin. Tergite 4 black pilose, except white pilose along lateral margins and with fascia of golden yellow pile on posterior 1/3, which is partly interrupted by black pile anteromedially. Sternite 1 dark brown; bare. Sternite 2 brown on anterior 2/3, yellow on posterior 1/3; white pilose. Sternite 3 yellow anteriorly and along posterior margin, brown medially; white pilose. Sternite 4 brown; black pilose, except white pilose along posterior margin. Male genitalia as in Fig. 352.

**Female.** Unknown.

#### Etymology.

The specific epithet (noun in the genitive case) refers to the country where the type has been collected.

### Rhopalosyrphus (s.s.) robustus

Reemer sp. n.

urn:lsid:zoobank.org:act:C36F4365-0BCF-467F-8BD0-35B4F9B28402

http://species-id.net/wiki/Rhopalosyrphus_robustus

#### Type specimens.

HOLOTYPE. Adult female. FRENCH GUYANA. Label 1: “FRENCH GUYANA. Patawa, / 4°32.658"N-52°9.132"W / VIII.2008. Malaise trap in / pine forest. Leg. O. Morvan”; label 2: “J. Skevington / mol. specimen no / JS3616 - CNC coll.”; label 3: “Voucher code M. Reemer / 271 / DNA voucher labcode MZH:Y1066”. Coll. CNC.

#### Diagnosis.

Care should be taken in assessing the presence of pile on the katepimeron: in this species this pilosity is very sparse and limited to the anterior margin. Within *Rhopalosyrphus* s.s. this species is readily distinguished by the pair of large yellow maculae on tergite 2.

#### Description (based on holotype).

Adult female. Body size: 14.5 mm.

**Head.** Face occupying about 1/4 of head width in frontal view; black, except yellow on lateral 1/6 on dorsal 2/3; entirely silvery white pilose. Gena black, white pilose. Lateral oral margins produced. Frons black; silvery white pilose. Vertex black; black pilose, except white pilose along eye margin. Occiput black; white pilose, except black pilose dorsolaterally. Eye bare; with narrow, horizontal area frontally at level of antenna where ommatidia are separated from each other by wide spaces; the ommatidia present in this area are larger than elsewhere on the eye. Antennal fossa about 1.5 times as wide as high. Antenna black; antennal ratio approximately as 5:1:9; longer than distance between antennal fossa and anterior oral margin.

**Thorax.** Black with faint metallic hues. Mesoscutum anteriad of transverse suture appressed white pilose, except for three patches of appressed black pile, and narrowly erect white pilose along anterior margin; mesoscutum posteriad of transverse suture appressed black pilose, except erect white pilose on posterior 1/3. Postpronotum and postalar callus white pilose, except postalar callus laterally black pilose. Scutellum semicircular; white pilose dorsally, golden pilose along margins; without calcars. Anepisternum with shallow sulcus; entirely silvery white pilose. Anepimeron entirely silvery white pilose. Katepisternum silvery white pilose dorsally, bare ventrally. Katepimeron almost bare: with very sparse white pile along anterior margin. Katatergum long microtrichose, anatergum short microtrichose. Calypter and halter yellow.

*Wing*: Hyaline, faintly infuscated on anterior 1/3. Microtrichose, except bare on cell bc, basal 1/3 of cell r1, entirely on cell br, posterobasally on cell r4+5 posteriad of vena spuria, posterior 1/2 of cell bm, anterior 1/2 of cell cup, and basomedian 3/5 of alula.

*Legs*: Front femur brown dorsally, black ventrally; white pilose, except for sparse black pile posteriorly. Front tibia black, except brown anteriorly on basal 1/4 and apical 1/3; white pilose, except black pilose ventrally. Front tarsus black, except fifth tarsomere brown; yellow pilose. Mid femur black, except brown anteriorly on basal half; white pilose. Mid tibia black, except yellowish on basal 1/6; white pilose. Mid tarsus black on basal three tarsomeres (other tarsomeres missing in holotype); yellow pilose. Hind femur strongly swollen, about 5 times as wide as mid femur; black; white pilose, except sparsely black pilose dorsally and densely occupied with short, bristly, black pile ventrally. Front coxa and trochanter brown; white pilose. Mid coxa black; white pilose. Mid trochanter brown; white pilose. Hind coxa and trochanter black; white pilose.

**Abdomen.** Constricted, about as wide as thorax, narrowest just before posterior margin of tergite 2. Tergites black with faint metallic hues, except tergite 2 with pair of large, elongate yellow maculae from anterior 1/4 to posterior 1/3. Tergite 1 white pilose. Tergite 2 white pilose, except for patch of black pile dorsally between middle of tergite and posterior 1/6. Tergite 3 black pilose, except narrowly white pilose along posterior and lateral margins. Tergite 4 pilose as tergite 3, but with sparse yellowish pile intermixed among the black pile on posterior 2/3. Tergite 5 mostly yellowish white pilose, with sparse black pile intermixed anteriorly, and colour of pile more whitish near posterior and lateral margins. Sternite 1 brown; white pilose. Sternite 2 yellow; white pilose. Sternite 3 brown; white pilose. Sternite 4 brown; white pilose, on anterior half, mostly black pilose on posterior half, yellowish pilose along posterior margin. Sternite 5 brown; mixed yellowish and black pilose.

**Male.** Unknown.

#### Etymology.

The Latin adjective *robustus* (strong as oak – *Robur*) was chosen as the specific epithet because of the size, robustness and stout hind femora of this species, which evoke the impression of a stout fly (strong animal).

### Rhopalosyrphus (s.l.) abnormoides

Reemer sp. n.

urn:lsid:zoobank.org:act:A572ADE8-FBFD-45BD-841E-4AB3A7A41A93

http://species-id.net/wiki/Rhopalosyrphus_abnormoides

#### Type specimens.

HOLOTYPE. Adult male. PARAGUAY. Label 1: “Paraguay / Fiebrig”; label 2: “S. Bernar- / dino”; label 3: “Myxogaster”; label 4: “Ropalosyrphus [male symbol] / ? auricinctus Sack / det. v. Doesburg”; label 5: “Voucher code M. Reemer / 289”. Coll. RMNH.

#### Diagnosis.

Within *Rhopalosyrphus* s.l. this species is closely related to *Rhopalosyrphus abnormis* (Curran, 1925). From that species it differs by the following characters (with character state in *Rhopalosyrphus abnormis* in parentheses): eye bare (pilose); antennal ratio approximately as 4:1:9 (2:1:2.5); scutellum without calcars (with calcars); anterior margin of tergite 2 clearly wider than posterior margin (about as wide).

#### Description (based on holotype).

Adult male. Body size: 11 mm.

**Head.** Face occupying a little less than 1/3 of head width in frontal view; yellow, with narrow, vaguely defined brown median vitta; entirely golden yellow pilose. Gena black, white pilose. Lateral oral margins produced. Frons black; silvery white pilose. Vertex black; black pilose, except yellow pilose along anterior and lateral margins. Occiput black; black pilose dorsally, golden pilose laterally, silvery white pilose on ventral half. Eye bare; with narrow, horizontal area frontally at level of antenna where ommatidia are separated from each other by wide spaces; the ommatidia present in this area are larger than elsewhere on the eye. Antennal fossa about 1.5 times as wide as high. Antenna black; antennal ratio approximately as 4:1:9; longer than distance between antennal fossa and anterior oral margin.

**Thorax.** Blackish brown with bronze and green metallic hues. Mesoscutum appressed black pilose, except for fasciae of appressed golden pile along anterior margin, transverse suture and posterior margin. Postpronotum and postalar callus white pilose. Scutellum semicircular; golden pilose; without calcars. Anepisternum with shallow sulcus; golden pilose anterodorsally, silvery white pilose posteriorly, widely bare anteroventrally. Anepimeron entirely silvery white pilose. Katepisternum silvery white pilose dorsally, bare ventrally. Katatergum long microtrichose, anatergum short microtrichose. Calypter and halter yellow.

*Wing*: Hyaline. Microtrichose, except bare on cell bc, basal 5/6 of cell br, basal 1/6 of cell cup.

*Legs*: Yellow, except hind femur dark brown and hind tibia medially dark brown. Front and mid legs white to yellow pilose, except mid femur dorsally, anteriorly and ventrally black pilose. Hind leg white to yellow pilose, except femur ventrally densely occupied with short, black, bristly pile. Front coxa orange, mid and hind coxae brown; all coxae white pilose. Front and mid trochanters yellow, hind trochanter brown; all trochanters white pilose.

**Abdomen.** Constricted, narrower than thorax, with narrowest point halfway tergite 2. Tergites brown with faint metallic hues, except tergite 2 with pair of large rectangular yellow maculae on basal 3/5. Tergite 1 white pilose. Tergite 2 yellow pilose, except sparsely black pilose medially and white pilose along posterior margin. Tergite 3 black pilose, except white pilose anterolaterally and along lateral margin, and with fascia of golden pile along posterior margin; this fascia medially interrupted and gradually narrowing towards lateral margins. Tergite 4 black pilose, except golden pilose anterolaterally and along lateral margin, and with pair of large triangular patches of golden pile over posterior 2/3. Sternite 1 brown; bare. Sternite 2 yellow; short yellow pilose. Sternites 3 and 4 brown; white pilose. Male genitalia as in Fig. 353.

**Female.** Unknown.

#### Etymology.

The name *abnormoides* (adjective) was chosen to underline the similarity of this species to *Microdon abnormis* Curran.

### Rhopalosyrphus (s.l.) oreokawensis

Reemer sp. n.

urn:lsid:zoobank.org:act:A17706B2-D5D4-462B-A00A-9166597A8FC8

http://species-id.net/wiki/Rhopalosyrphus_oreokawensis

#### Type specimens.

HOLOTYPE. Adult male. FRENCH GUYANA. Label 1: “FRENCH GUYANA / Kaw Mountains / 04°32.893"N-52°10.245"W / 27.XI.2002. leg. V. Soon”. Coll. RMNH.

#### Diagnosis.

Within *Rhopalosyrphus* s.l. this species is singular because of its short antenna (slightly shorter than distance between antennal fossa and anterior oral margin) and the shape of the ventral part of the face.

#### Description (based on holotype).

Adult male. Body size: 13 mm.

**Head.** Face occupying a little more than 1/3 of head width in frontal view; black; entirely white pilose; ventral part of face anteriad of oral margin with lateral bulges, medially separated by shallow, smooth sulcus. Gena black, white pilose. Lateral oral margins slightly produced. Frons, vertex and occiput black; white pilose, except for sparse black pile on frons. Eye bare. Antennal fossa about as high as wide. Antenna brown; antennal ratio approximately as 4:1:3; slightly shorter than distance between antennal fossa and anterior oral margin.

**Thorax.** Black. Mesoscutum black pilose, except for fasciae of white pile along anterior margin and transverse suture, and patches of white pile posterolaterally. Postpronotum and postalar callus white pilose. Scutellum semicircular; white pilose; with small calcars with mutual distance about equal to 1/4 of width of scutellum. Anepisternum with deep sulcus; black pilose anterodorsally, white pilose posteriorly, widely bare in between. Anepimeron entirely white pilose. Katepisternum white pilose dorsally, bare ventrally. Katatergum long microtrichose, anatergum short microtrichose. Calypter yellow. Halter brown.

*Wing*: Hyaline, but infuscated at apical 1/2 of cell c, cell sc, around vein r-m, around vein R4+5 and posterior appendix of that vein, around vein dm-cu and around bm-cu. Microtrichose, except bare on cell bc, basal 1/2 of cell c, basal 1/2 of cell r1, basal 1/3 of cell r2+3, posterobasal 1/4 of cell r4+5, entirely bare on cell br, basal 9/10 of cell bm, basal 2/3 of cell cup, and entirely on alula.

*Legs*: Front and mid legs orange brown, except mid femur blackish brown on basal 2/5; white pilose, except front tibia black pilose on apicodorsal 1/4, tarsi dorsally black pilose and mid femur on apical 1/2 posterodorsally with sparse bristle-like pile among long white pile. Hind leg black, except basal 1/2 of tibia and apical four tarsomeres dark brown; white pilose, except tarsus dorsally black pilose and femur on apical 1/5 with row of short, black, bristle-like pile anteroventrally; femur swollen: about 2.5 times as wide as mid femur. Front coxa brown, mid and hind coxae black; all coxae white pilose. Front and mid trochanters brown, hind trochanter black; all trochanters white pilose.

**Abdomen.** Constricted, narrower than thorax, with narrowest point at transition between segments 2 and 3. Tergites black, except tergite 2 with pair of large elongate yellow maculae on basal 3/5 and narrowly yellow along posterior margin, and tergite 3 vaguely brownish yellow along anterior margin. Tergite 1 white pilose. Tergite 2 short black pilose, except with long white pile anterolaterally and with thick, appressed white pile along posterior margin. Tergite 3 short black pilose, except for medially interrupted fasciae of thick, appressed white pile along anterior and posterior margins. Tergite 4 yellow pilose on lateral 1/4 and posterior 3/5, black pilose anteromedially and on narrow median vitta on posterior 3/5. Sternite 1 black; bare. Sternite 2 yellow except black along posterior margin; with sparse long white pile. Sternite 3 yellow at anterior 3/5, black posteriorly; short black pilose on posterior 1/2 to 3/5, long, thick white pilose along posterior and posterolateral margins. Sternite 4 brown; black pilose medially, yellow pilose laterally. Male genitalia as in Fig. 355.

**Female.** Unknown.

#### Etymology.

The specific epithet (noun in the genitive case) is composed of the Greek *oreos* (mountain) and Kaw, the name of the French Guyanan mountain region in which the species was found.

#### Notes.

This species is very aberrant from other known species of *Rhopalosyrphus* because of the short antenna, the straight facial profile, the bare katepisternum and the long and slender second abdominal segment. These characters suggest that the species may not belong in *Rhopalosyrphus*. However, it is certainly related to that genus, considering the structure of the male genitalia and the constricted abdomen. If a new genus were to be erected for this species, more evidence on its phylogenetic affinities to *Rhopalosyrphus* and other related genera (e.g. *Pseudomicrodon*) should be available.

### Thompsodon conspicillifrons

Reemer sp. n.

urn:lsid:zoobank.org:act:3E0D62D7-5B4C-40B9-A8E6-F20C82361A00

http://species-id.net/wiki/Thompsodon_conspicillifrons

Figs 406 –416

Microdon MCR-12 Thompson, in litt.

#### Type specimens.

HOLOTYPE. Female. Label 1: “COSTA RICA, Prov. Limón, / A.C.L.A.C., Talamanca, San Miguel, / Albergue, CASACODE, Send Cerillos. / 10-30 m. 23-26 FEB 1999. M. Lobo. / L_S_391000_612000 #52454”; label 2: “MCR-12”; label 3 (barcode): “INB0003024775 / INBIOCRI COSTA RICA”. Coll. INBIO.

#### Diagnosis.

See generic key and genus account of *Thompsodon* (only one species known).

#### Description (based on holotype).

Adult male. Body size: 8 mm.

**Head.** Face occupying about 1/3 of head width in frontal view; yellow with black median vitta, which is dorsally about as wide as the antennal fossa and gradually narrows downward, becoming absent in ventral 1/4; yellow pilose, except for sparse black pile submedially, narrowly bare medially. Gena blackish; yellow pilose. Lateral oral margins not produced. Frons black; golden pilose; laterally with round, concave areas, filled with dense golden pile, ventrally delimited by a sharply defined ridge. Vertex irregularly swollen; black; short golden pilose. Ocellar triangle not elevated. Occiput narrow ventrally, strongly widened dorsally; black; golden pilose. Eye bare. Antennal fossa about as high as wide. Antenna with scape pale brown, pedicel and basoflagellomere blackish brown; antennal ratio approximately as 4:1:4.

**Thorax.** Mesoscutum black; golden pilose, except for pair of black pilose patches anteriad of transverse suture and wide fascia of black pile posteriad of transverse suture. Postpronotum and postalar callus brown, golden pilose. Scutellum black; golden pilose. Pleuron yellowish brown, except anepisternum and anepimerond blackish. Anepisternum with deep sulcus separating posterior from anterior part; entirely mixed yellow and black pilose. Anepimeron entirely mixed yellow and black pilose. Katepisternum long yellow pilose dorsally, bare ventrally. Katatergum short microtrichose, anatergum bare. Calypter dark greyish. Halter yellow.

*Wing*: hyaline, slightly brownish, especially anteriorly; microtrichose, except bare on cell bc, posterobasal 1/6 of cell c, basal 1/6 of cell r1, entirely on cell br except microtrichose on vena spuria, basal 1/2 of cell bm, and basal 1/2 of cell cup.

*Legs*: Femora blackish brown, except yellow apically; black pilose. Tibiae and tarsi yellow. Tibiae yellow pilose, except black pilose apically. Tarsi black pilose. Coxae and trochanters blackish brown; black pilose.

**Abdomen.** More or less oval, but tergite 1 very narrow, so appears constricted basally. Tergites 3 and 4 not fused, able to articulate independently. Tergites blackish. Tergite 1 yellow pilose. Tergite 2 short black pilose, with medially interrupted fascia of longer golden pile along posterior margin. Tergite 3 with similar pattern of pile as tergite 2, but fascia of golden pile medially strongly extended over median part of tergite. Tergite 4 largely golden pilose, except for narrow median vitta of black pile and sublateral oblique vittae of black pile. Tergite 5 golden pilose. Sternites black. Sternite 1 bare, other sternites golden pilose.

**Male.** Unknown.

#### Etymology.

The specific epithet (noun in apposition) is composed of the Latin words *conspicillum* (spectacles) and *frons* (forehead). The name refers to the concave lateral areas on the frons which (in the eyes of susceptible beholders) evoke the impression of glasses on a forehead.

#### Notes.

This species was first recognized as an undescribed taxon by F.C. Thompson, who gave it the preliminary code-name *Microdon* MCR-12.

## Appendix 2. Species classification of Microdontinae

In total, 552 species group names (excluding misspellings) applying to Microdontinae are currently known, including 98 synonyms and 26 species described in the present paper. Based on the generic diagnoses and discussions in the preceding section of this paper, the classification of all but a few of these species is re-evaluated. This has resulted in a new species classification, partly based on examination of type material. Primary types (or, in seven of these cases, photographic images of those) of 347 specific taxa were examined. In addition, the classification of six species is based on paratypes. In several cases, no type material was examined, e.g. in the case of well-known taxa from temperate regions, in the case of groups that have been revised by other authors (*Mixogaster*, *Spheginobaccha*), in the case of recently described species of which good illustrations are available, and in cases of species of which the types could not be found. For these cases, original descriptions, additional material and literature have been consulted. For each taxon, the source of the information on which the classification was based is indicated (for legend see below).

Of all 552 available species group names, 454 are here considered as valid, 98 as synonyms (19 of which are new synonyms proposed here). Of all available species group names, 267 are presented here in new combinations, while 20 species names (including three synonyms) are left unplaced.

The following format is used:

**species name** Author, Year: page (*Original genus*). COLLECTION: KIND_OF_TYPE SEX/STAGE. **[SOURCE]** **Taxonomic act (e.g. comb. n., stat. n.).** Remarks.

*: An asterisk denotes information which supplements or corrects information in Systema Dipterorum (Thompson 2010).

Acronyms for type information follow Systema Dipterorum (Thompson 2010):

KIND_OF_TYPE: HT = holotype; LT = lectotype; NT = neotype; ST = syntype(s); T = unspecified.

SEX/STAGE: A = adult; F = female; L = larva; M = male; P = puparium.

[SOURCE]: This indicates the source of the information on which the classification is based. The following codes are used:

1a = primary type(s) studied

1b = photograph(s) of primary type(s) studied

1c = paratype(s) studied

2 = description studied

3 = non-type specimens studied

4 = additional literature studied

Synonymies are based on Thompson (2010), unless they are marked with “**Syn. n.**”. In the latter case, they are based on the judgement of the first author. Information on the type locality and a full reference to the description is omitted, as this can be found in Thompson (2010) and the regional Diptera catalogues.

**Genus *Afromicrodon* Thompson, 2008**

**Afrotropical**

**comoroensis** De Meyer, De Bruyn & Janssens, 1990: 571 (*Ceratophya*). RMCA*: HT* M*. **[1a]**

**johannae** Doesburg, 1957: 109 (*Ceratophya*). MNHN*: HT* M*. **[1a]** Paratypes in RMNH.

**luctiferus** Hull, 1941: 320 (*Microdon*). ANSP: T F*. [**3: RMCA**] **Comb. n.**

**madecassa** Keiser, 1971: 256 (*Ceratophya*). MNHN*: HT* M*. **[1a]** Possible junior synonym of *Afromicrodon luctiferus* (Hull, 1941).

**stuckenbergi** Keiser, 1971: 258 (*Ceratophya*). MNHN*: HT* F*. **[1a]**

**Genus *Archimicrodon* Hull, 1945 stat. n.**

**Subgenus *Archimicrodon* Hull, 1945**

**Afrotropical**

**ampefyanus** Keiser, 1971: 239 (*Microdon*). MNHN*: HT* M*. **[1a]** **Comb. n.**

**brevicornis** Loew, 1858: 376 (*Microdon*). NHRS*: ST* MF*. **[1a]** **Comb. n.**

**caeruleomaculatus** Keiser, 1971: 241 (*Microdon*). MNHN*: HT* F*. **[1a]** **Comb. n.**

**clatratus** Keiser, 1971: 240 (*Microdon*). MNHN*: HT* F*. **[1a]** **Comb. n.**

**fenestrellatus** Keiser, 1971: 242 (*Microdon*). MNHN*: HT* M*. **[1a]** **Comb. n.**

**kavitahaius** Keiser, 1971: 243 (*Microdon*). MNHN*: HT* F*. **[1a]** **Comb. n.**

**liberiensis** Curran, 1929: 4 (*Microdon*). AMNH: HT F*. **[1c]** **Comb. n.** Paratypes (1 male & 1 female) in RMCA.

**malagasicus** Keiser, 1971: 244 (*Microdon*). MNHN*: HT* F*. **[1a]** **Comb. n.**

**nigrocyaneus** Hull, 1964: 460 (*Microdon*). MZLU: HT* F*. **[1a]** **Comb. n.**

**obesus** Herve-Bazin, 1913: 100 (*Microdon*). RMCA*: HT* M*. **[1a]** **Comb. n.** Holotype (male) & allotype (female) in RMCA.

**ranavalonae** Keiser, 1971: 246 (*Microdon*). MNHN*: HT* F*. **[1a]** **Comb. n.**

**sudanus** Curran, 1923: 146 (*Microdon*). BMNH: HT F*. **[1a]** **Comb. n.**

**tenuifrons** Curran, 1929: 5 (*Microdon*). AMNH: HT M. **[1a]** **Comb. n.**

**testaceus** Walker, 1857: 152 (*Microdon*). BMNH (lost)*: T A. **[3: RMNH & ZMAN]** **Comb. n.**

**wainwrighti** Curran, 1938: 6 (*Microdon*). AMNH: HT M*. **[1a]** **Comb. n.**

**Australian / Oceanian**

**barringtonensis** Ferguson, 1926: 180 (*Microdon*). ANIC*: HT* M*. **[1b]** **Comb. n.**

**boharti** Curran, 1947: 2 (*Microdon*). AMNH: HT F. **[1a]** **Comb. n.**

**brachycerus** Knab & Malloch, 1912: 235 (*Microdon*). USNM*: HT* M. **[1a]** **Comb. n.** Type specimen with empty puparium.

**browni** Thompson, 1968: 44 (*Microdon*). MCZ: HT M. **[1b, 2]** **Comb. n.**

**fergusoni** Goot, 1964: 220 (*Microdon*). QMBA*: T M*. **[1a]** **Comb. n.** Replacement name for *Microdon modestus* Ferguson, 1926.

= *modestus* Ferguson, 1926: 179 (*Microdon*). **Comb. n.** Preocc. Knab, 1917.

**=** *fergusoni* Thompson, 1968: 44 (*Microdon*). **Comb. n.** Preocc. Goot, 1964.

**grageti** Meijere, 1908: 207 (*Microdon*). HNHM (lost)*: T M. **[2]** **Comb. n.** Type lost (de Jong 2000).

**incisuralis** Walker, 1865: 113 (*Paragus*). BMNH: HT* F. **[1a]** **Comb. n.**

**limbinervis** Meijere, 1908: 208 (*Microdon*). HNHM (lost)*: T F. **[3: ZMAN, ID by De Meijere].** **Comb. n.** Type lost (de Jong 2000). Non-type female identified by de Meijere in ZMAN.

**luctiferus** Walker, 1865: 113 (*Paragus*). BMNH: T F. **[1a]** **Comb. n.**

**malukensis** Reemer, sp. n. (*Archimicrodon*). RMNH: HT M. **[1a] Sp.n.**

**nicholsoni** Ferguson, 1926: 173 (*Microdon*). ANIC*: HT* F. **[1b]** **Comb. n.**

**novaeguineae** Meijere, 1908: 206 (*Microdon*). ZMAN*: ST* F*. **[1a]** **Comb. n.**

**purpurescens** Shiraki, 1963: 147 (*Microdon*). USNM: HT F. **[2]** **Comb. n.**

**tabanoides** Hull, 1944: 246 (*Microdon*). BMNH: HT* F. **[1a]** **Comb. n.**

**venosus** Walker, 1865: 112 (*Paragus*). BMNH: T M*. **[1a]** **Comb. n.**

= *papuanus* Doesburg, 1959: 234 (*Microdon*). RMNH: HT* **[1a]** **Syn. n., comb. n.**

**vittatus** Macquart, 1850: 433 (*Aphritis*). OUMNH*: T F. **[1a]** **Comb. n.**

= *transiens* Walker, 1852: 225 (*Eumerus*). BMNH: T F. **[1a]** **Comb. n.**

= *pachypus* Bigot, 1884: 541 (*Paragus*). BMNH: HT* **[1a]** **Comb. n.**

**Oriental**

**caeruleus** Brunetti, 1908: 92 (*Microdon*). ZSI*: HT F. **[2]** **Comb. n.**

**clavicornis** Sack, 1926: 592 (*Microdon*). USNM*: HT* F. **[1a]** **Comb. n.**

**investigator** Hull, 1937: 20 (*Microdon*). MCZ: HT M. **[1b]** **Comb. n.**

**lanka** Keiser, 1958: 213 (*Microdon*). NMB Basel: HT* F. **[1a] Comb. n.**

**minuticornis** Curran, 1931: 342 (*Microdon*). BMNH: HT F*. **[1c: USNM]** **Comb. n.**

**simplicicornis** Meijere, 1908: 205 (*Microdon*). ZMAN: HT* M. **[1a]** **Comb. n.**

= *digitator* Hull, 1937: 19 (*Microdon*). MCZ: HT M. **[1b]** **Syn. n., comb. n.**

**varicornis** Sack, 1926: 594 (*Microdon*). USNM*: HT* F. **[1a] Comb. n.**

**Palaearctic**

**simplex** Shiraki, 1930: 15 (*Microdon*). NIAS: T A. **[3: RMNH & coll. M. Hauser]** **Comb. n.** Described as var. of the Oriental *Microdon caeruleus* Brunetti, 1908.

**Subgenus *Hovamicrodon* Keiser, 1971**

**Afrotropical**

**flavifacies** Keiser, 1971: 248 (*Hovamicrodon*). MNHN*: T F*. **[1a]** **Comb. n.**

**fuscipennis** Keiser, 1971: 249 (*Hovamicrodon*). MNHN*: T F*. **[1a]** **Comb. n.**

**hova** Herve-Bazin, 1913: 398 (*Microdon*). MNHN*: ST* MF*. **[1a]** **Comb. n.**

**nubecula** Keiser, 1971: 250 (*Hovamicrodon*). MNHN*: T F*. **[1a]** **Comb. n.**

**silvester** Keiser, 1971: 251 (*Hovamicrodon*). MNHN*: HT* M*. **[1a]** **Comb. n.**

**vulpicolor** Hull, 1941: 321 (*Microdon*). ANSP: T F*. **[3: RMCA] Comb. n.**

**Genus *Aristosyrphus* Curran, 1941**

**Subgenus *Aristosyrphus* Curran, 1941**

**Neotropical**

**boraceiensis** Papavero, 1962: 319 (*Ceratophya*). MZUSP: HT F. **[2]**

**carpenteri** Hull, 1945: 76 (*Ceratophya*). MCZ: HT F. **[1b]**

**minutus** Thompson, 2004: 567 (*Aristosyrphus*). DZUP: HT* M*. **[2]**

**primus** Curran, 1941: 252 (*Aristosyrphus*). AMNH: HT M. **[1a]**

**Subgenus *Eurypterosyrphus* Barretto & Lane, 1947**

**Neotropical**

**currani** Goot, 1964: 214 (*Microdon*). BMNH*: F*. **[1a]** Replacement name for *Microdon clavicornis* Curran, 1940.

= *clavicornis* Curran, 1940: 6 (*Microdon*). BMNH: T F. Preocc. Sack, 1926.

**macropterus** Curran, 1941: 254 (*Ceratophya*). AMNH: HT F. **[1a]**

**melanopterus** Barretto & Lane, 1947: 142 (*Eurypterosyrphus*). MZUSP: HT F. **[1a]**

**Genus *Bardistopus* Mann, 1920**

**Australian / Oceanian**

**papuanum** Mann, 1920: 61 (*Bardistopus*). USNM: HT M. **[1a]**

**Genus *Carreramyia* Doesburg, 1966 stat. n.**

**Neotropical**

**flava** Sack, 1941: 117 (*Ceratophya*). SNSD*: HT* F*. **[1a]** **Comb. n.**

**megacephalus** Shannon, 1925: 213 (*Microdon*). USNM: HT M. **[1a]**

[Descriptions of two additional species from the Neotropical region are in preparation by the first author.]

**Genus *Ceratophya* Wiedemann, 1830**

**Neotropical**

**carinifacies** Curran, 1934: 376 (*Microdon*). AMNH: T F. **[1a]**

**notata** Wiedemann, 1824: 14 (*Ceratophya*). NMW*: M*. **[1a]**

**panamensis** Curran, 1930: 6 (*Microdon*). AMNH: HT M. **[1a]**

**scolopus** Shannon, 1927: 20 (*Microdon*). BMNH: T M. **[1a]** **Comb. n.**

[The description of an additional species from the Neotropical region is in preparation by the first author.]

**Genus *Ceratrichomyia* Séguy, 1951**

**Afrotropical**

**angolensis** Reemer sp. n. (*Ceratrichomyia*). CNC: HT M. **[1a] Sp. n.**

**behara** Séguy, 1951: 14 (*Ceratrichomyia*). MNHN: LT* M*. **[1a]**

**bullabucca** Reemer sp. n. (*Ceratrichomyia*). MNHN: HT M. **[1a] Sp. n.**

**Genus *Ceriomicrodon* Hull, 1937 stat. n.**

**Neotropical**

**petiolatus** Hull, 1937: 25 (*Ceriomicrodon*). USNM: T M*. **[1a]**

**Genus *Cervicorniphora* Hull, 1945 stat. n.**

**Australian**

**alcicornis** Ferguson, 1926: 171 (*Microdon*). ANIC*: HT* M. **[1b]**

**Genus *Chrysidimyia* Hull, 1937 stat. n.**

**Neotropical**

**chrysidimima** Hull, 1937: 116 (*Chrysidimyia*). CM*: M*. **[1a]**

= *chrysidiformis* Hull, 1944: 241 (*Chrysidimyia*). Misspelling of *C. chrysidimima*.

= *granulatus* Curran, 1940: 9 (*Microdon*). BMNH: HT F. **[1a]** **Syn. n., comb. n.**

= *lazuli* Hull, 1944: 241 (*Chrysidimyia*). BMNH: HT F. **[1a] Syn. n.**

**Genus *Domodon* Reemer gen. n.**

**Neotropical**

**zodiacus** Reemer sp. n. (*Domodon*). RMNH: HT M. **[1a] Sp. n.**

**Genus *Furcantenna* Cheng, 2008**

**Oriental**

**nepalensis** Reemer sp. n. (*Furcantenna*). CNC: HT M. **[1a] Sp. n.**

**yangi** Cheng, 2008: 29 (*Furcantenna*). CASB: HT M. **[2]**

**Genus *Heliodon* Reemer gen. n.**

**Oriental**

**chapini** Hull, 1941: 438 (*Microdon*). USNM: HT M. **[1a]** **Comb. n.**

**doris** Reemer sp. n. (*Heliodon*). RMNH: HT M. **[1a] Sp. n.**

**elisabeth** Keiser, 1958: 211 (*Microdon*). NMB Basel: HT M. **[1a] Comb. n.**

**elisabethanna** Reemer sp. n. (*Heliodon*). QSBG: HT F. **[1a] Sp. n.**

**gloriosus** Hull, 1941: 439 (*Microdon*). USNM: HT M. **[1a] Comb. n.**

= *aurivesta* Hull, 1950: 611 (*Microdon*). BMNH: HT M. **[1a]** **Syn. n.**

**klossi** Curran, 1931: 343 (*Microdon*). BMNH: HT M. **[1a] Comb. n.**

**tiber** Reemer sp. n. (*Heliodon*). ZMAN: HT M. **[1a] Sp. n.**

**tricinctus** Meijere, 1908: 208 (*Microdon*). ZMAN: ST* MF*. **[1a]** **Comb. n.**

**Genus *Hypselosyrphus* Hull, 1937 stat. n.**

**Neotropical**

**amazonicus** Reemer, in prep. (*Hypselosyrphus*). **Nom. n.** Replacement name for *Microdon scutellaris* Shannon, 1927.

= *scutellaris* Shannon, 1927: 20 (*Microdon*). BMNH: T F. **[1a]** **Comb. n.** Preocc. Schummel, 1842

**anax** Thompson, 1976: 61 (*Microdon*). **Comb. n.** Replacement name for *Microdon analis* Curran, 1940.

= *analis* Curran, 1940: 3 (*Microdon*). AMNH: HT M. **[1a] Comb. n.** Preocc. Macquart, 1842.

**corbiculipes** Papavero, 1962: 320 (*Hypselosyrphus*). MZUSP: HT F. **[1a]**

**plaumanni** Curran, 1940: 3 (*Microdon*). AMNH: T F. **[1a]** **Comb. n.**

**trigonus** Hull, 1937: 21 (*Hypselosyrphus*). MCZ: T M. **[1a]** **Comb. n.**

**ulopodus** Hull, 1944: 34 (*Ubristes*). CU: T F. **[1a]** **Comb. n.**

[Descriptions of five additional species from the Neotropical region are in preparation by the first author.]

**Genus *Indascia* Keiser, 1958**

**Oriental**

**brachystoma** Wiedemann, 1824: 33 (*Ascia*). ZMUC: LT M. **[1a]**

**gigantica** Reemer sp. n. (*Indascia*). QSBG: HT M. **[1a] Sp. n.**

**gracilis** Keiser, 1958: 223 (*Indascia*). NMB: HT M. **[1a]**

**spathulata** Reemer sp. n. (*Indascia*). RMNH: HT M. **[1a] Sp. n.**

**Genus *Kryptopyga* Hull, 1944**

**Oriental**

**pendulosa** Hull, 1944: 130 (*Kryptopyga*). BMNH: HT M. **[1a]**

**sulawesiana** Reemer sp. n. (*Kryptopyga*). RMNH: HT M. **[1a] Sp. n.**

**Genus *Laetodon* Reemer gen. n.**

**Nearctic**

**laetoides** Curran, 1935: 3 (*Microdon*). AMNH: HT F. **[1a]** **Comb. n.**

**laetus** Loew, 1864: 74 (*Microdon*). MCZ (lost): ST MF. **[3: USNM] Comb. n.**

= *scitulus* Williston, 1887: 10 (*Microdon*). USNM: HT M. **[4: Thompson 1981b] Comb. n.**

**solitarius** Curran, 1930: 8 (*Microdon*). AMNH*: HT F*. **[1a]** **Comb. n.**

**violens** Townsend, 1895: 34 (*Microdon*). SEMC*: T F. **[3: USNM] Comb. n.**

**Neotropical**

**geijskesi** Doesburg, 1966: 80 (*Microdon*). RMNH*: T M. **[1a]** **Comb. n.**

**Genus *Masarygus* Brèthes, 1908**

**Neotropical**

**palmipalpus** Reemer sp. n. (*Masarygus*). RMNH: HT M. **[1a] Sp. n.**

**planifrons** Brèthes, 1908: 442 (*Masarygus*). MACN: ST MF*. **[1a]**

**Genus *Menidon* Reemer gen. n.**

**Neotropical**

**falcatus** Williston, 1887: 9 (*Microdon*). USNM: LT M. **[3: RMNH & USNM] Comb. n.**

= *aquilinus* Giglio-Tos, 1892: 2 (*Microdon*). MRSN: HT F. **[4: Thompson 2007]** **Comb. n.**

= *hondurania* Hull, 1940: 247 (*Microdon*). CNC*: HT* M*. **[4: Thompson 2007]** **Comb. n.**

= *mellogutta* Hull, 1943: 104 (*Microdon*). BMNH: HT F. **[4: Thompson 2007] Comb. n.**

**Genus *Mermerizon* Reemer gen. n.**

**Neotropical**

**inbio** Reemer, sp. n. (*Mermerizon*). INBIO: HT M. **[1a] Sp. n.**

**Genus *Metadon* Reemer gen. n.**

**Afrotropical**

**aethiopicus** Rondani, 1873: 282 (*Microdon*). MCGD*: T F*. **[3: BMNH] Comb. n.**

**apis** Speiser, 1913: 145 (*Microdon*). Type lost?*: T F*. **[2]** **Comb. n.** Type not in NMSA, not in SAMC.

**appendiculatus** Curran, 1929: 6 (*Microdon*). AMNH*: HT* M*. **[1a]** **Comb. n.** Paratype female in RMCA.

**aureomagnificus** Hull, 1944: 242 (*Microdon*). BMNH: HT* M*. **[1a]** **Comb. n.**

**captum** Speiser, 1913: 146 (*Microdon*). T F*. **[3: ZMAN & coll. M. Hauser] Comb. n.** Male described by Van Doesburg 1956, but this description seems to apply better to the male of *Microdon punctulatus* Wiedemann.

**erythrocephalus** Bezzi, 1915: 130 (*Microdon*). BMNH: T F*. **[1a]** **Comb. n.**

**inappendiculatus** Curran, 1929: 7 (*Microdon*). AMNH: HT M. **[1a]** **Comb. n.**

**inermis** Loew, 1858: 376 (*Microdon*). NHRS*: T M*. **[1a]** **Comb. n.**

**modesticolor** Hull, 1944: 251 (*Microdon*). BMNH*: HT* M*. **[1a]** **Comb. n.**

**mydas** Bezzi, 1915: 128 (*Microdon*). BMNH: ST* MF*. **[1a]** **Comb. n.**

**mynthes** Seguy, 1953: 157 (*Microdon*). MNHN: T M*. **[1a]** **Comb. n.**

**pallidus** Bezzi, 1915: 133 (*Microdon*). BMNH: ST* MF*. **[1a]** **Comb. n.**

**punctulatus** Wiedemann, 1824: 32 (*Microdon*). ZMUC*: HT* M*. **[1a]** **Comb. n.**

= *microtuberculatus* Hull, 1964: 459 (*Microdon*). MZLU: HT* F*. **[1a]** **Syn. n., comb. n.** Paratype female also in MZLU.

**rugosus** Bezzi, 1915: 126 (*Microdon*). BMNH: HT* M*. **[1a] Comb. n.**

= *concolor* Bezzi, 1923: 349 (*Microdon*). MNHN*: HT* M*. **[1a]** **Comb. n.**Described as subspecies of *Microdon rugosus* Bezzi, 1915.

= *fuscus* Bezzi, 1921: 21 (*Microdon*). T M*. **[2]** **Comb. n.** Described as var. of *Microdon rugosus* Bezzi, 1915. Junior primary homonym of *Microdon fuscus* Meijere, 1908.

= *melas* Bezzi, 1921: 21 (*Microdon*). T M*. **[2]** **Comb. n.** Described as var. of *Microdon rugosus* Bezzi, 1915.

= *minor* Bezzi, 1921: 20 (*Microdon*). T M*. **[2]** **Comb. n.** Described as var. of *Microdon rugosus* Bezzi, 1915.

**Australian / Oceanian**

**apicalis** Walker, 1858: 94 (*Microdon*). BMNH: T F. **[1a]** **Comb. n.**

**fulvicornis** Walker, 1858: 94 (*Microdon*). BMNH: HT* F*. **[1a]** **Comb. n.**

= *tuberculatus* Meijere, 1913: 359 (*Microdon*). ZMAN: T M. **[1a]** **Syn. n., comb. n.**

**Oriental**

**achterbergi** Reemer sp. n. (*Metadon*). RMNH: HT F. **[1a] Sp. n.**

**albofascia** Hull, 1944: 253 (*Microdon*). BMNH: HT F. **[1a]** **Comb. n.**

**annandalei** Brunetti, 1907: 13 (*Microdon*). ZSI: HT M. **[3: BMNH & SEMC] Comb. n.**

**auricinctus** Brunetti, 1908: 93 (*Microdon*). BMNH: HT M*. **[1a]** **Comb. n.**

**auroscutatus** Curran, 1928: 152 (*Microdon*). BMNH: HT M*. **[1a]** **Comb. n.**

=*variventris* Curran, 1928: 154 (*Microdon*). BMNH: HT F. **[1a]** **Comb. n.** Described as var. of *Microdon auroscutatus* Curran, 1928.

**bicolor** Sack, 1922: 272 (*Microdon*). DEI*: T M*. **[1a]** **Comb. n.**

**bicoloratus** Hull, 1944: 254 (*Microdon*). BMNH: HT M. **[1a] Comb. n.**

**flavipes** Brunetti, 1908: 92 (*Microdon*). ZSI: HT F. **[2]** **Comb. n.**

**fulvipes** Meijere, 1908: 203 (*Microdon*). RMNH*: T F. **[1a]** **Comb. n.**

=*aurilinea* Hull, 1944: 258 (*Microdon*). BMNH: HT M*. **[1a] Syn. n., comb. n.**

**fuscicornis** Sasakawa, 1960: 451 (*Microdon*). T M. **[2]** **Comb. n.**

**fuscus** Meijere, 1908: 204 (*Microdon*). ZMAN*: T F. **[1a]** **Comb. n.**

**montis** Keiser, 1958: 214 (*Microdon*). NMB Basel: HT M. **[1a]** **Comb. n.**

**pendelburyi** Curran, 1931: 305 (*Microdon*). BMNH*: HT* F*. **[1a]** **Comb. n.**

**pretiosus** Curran, 1931: 304 (*Microdon*). BMNH: HT M. **[1a]** **Comb. n.**

**robinsoni** Curran, 1928: 154 (*Microdon*). BMNH: HT F. **[1a]** **Comb. n.**

**ruficaudus** Brunetti, 1907: 93 (*Microdon*). BMNH: HT F. **[1a]** **Comb. n.**

**rutiliventris** Vockeroth, 1975: 371 (*Microdon*). AMNH*: T F*. **[1b]** **Comb. n.** Replacement name for *Microdon rufiventris* Curran, 1942.

= *rufiventris* Curran, 1942: 4 (*Microdon*). BMNH: HT F. **[1b]** **Comb. n.** Preocc. Rondani, 1848.

**rutilus** Keiser, 1952: 173 (*Microdon*). NMB Basel: HT M. **[1a]** **Comb. n.**

**sacki** Goot, 1964: 220 (*Microdon*). USNM?*: HT M*. **[3: type of var. *nigritus* Hull]** **Comb. n.** Replacement name for *Microdon flavipennis* Sack, 1926.

= *flavipennis* Sack, 1926: 593 (*Microdon*). USNM?*: T M. **[2]** **Comb. n.** Preocc. Curran, 1925.

= *nigrita* Hull, 1944: 257 (*Microdon*). BMNH: HT M. **[1a]** **Comb. n.** Described as var. of *Microdon flavipennis* Sack, 1926.

**squamipennis** Brunetti, 1923: 316 (*Microdon*). ZSI: HT F. **[2]** **Comb. n.**

**taprobanicus** Keiser, 1958: 212 (*Microdon*). NMB Basel: HT M. **[1a]** **Comb. n.**

**wulpii** Mik, 1899: 143 (*Microdon*). RMNH*: T F*. **[1a]** **Comb. n.** Replacement name for *Microdon apicalis* Wulp, 1881.

= *apicalis* Wulp, 1892: 29 (*Microdon*). RMNH*: HT* F. **Comb. n.** Preocc. Walker, 1859. Knutson et al. (1975) erroneously cite publication year as 1881.

= *wulpii* Brunetti, 1908: 93 (*Microdon*). RMNH*: T F*. **Comb. n.** Unnecessary replacement name for *Microdon apicalis* Wulp, 1881. Preocc. Mik, 1899.

**Palaearctic**

**bifasciatus** Matsumura, 1916: 254 (*Microdon*). SEHU*: T F. **[3: RMNH] Comb. n.**

**brunneipennis** Huo, Ren & Zheng, 2007: 398 (*Microdon*). HU*: HT* F*. **[2]** **Comb. n.**

**pingliensis** Huo, Ren & Zheng, 2007: 401 (*Microdon*). HU*: HT* M*. **[2]** **Comb. n.**

**spuribifasciatus** Huo, Ren & Zheng, 2007: 403 (*Microdon*). HU*: HT* M*. **[2]** **Comb. n.**

**Genus *Microdon* Meigen, 1803**

**Subgenus *Chymophila* Macquart, 1834**

**Nearctic**

**fulgens** Wiedemann, 1830: 82 (*Microdon*). ZMHU: LT F. **[1a]**

= *euglossoides* Gray, 1832: 779 (*Microdon*). OUMNH: T A. **[4: Thompson et al. 1976]**

= *splendens* Macquart, 1834: 486 (*Chymophila*). OUMNH: LT M. **[1a]**

**Neotropical**

**angulatus** Hull, 1943: 715 (*Microdon*). BMNH: T M. **[1a]** **Comb. n.**

**argentinae** Hull, 1937: 18 (*Microdon*). MCZ: T M. **[1b]** **Comb. n.**

**aurifacius** Hull, 1937: 169 (*Microdon*). USNM*: M*. **[1a]** **Comb. n.**

**aurifex** Wiedemann, 1830: 85 (*Microdon*). NMW: T M*. **[1a] Comb. n.**

= *trochilus* Walker, 1852: 216 (*Microdon*). BMNH: T M*. **[1a] Comb. n.**

**barbiellinii** Curran, 1936: 6 (*Microdon*). AMNH: T M. **[1a]** **Comb. n.**

**bruchi** Shannon, 1927: 38 (*Microdon*). USNM: ST F. **[1a]** **Comb. n.**

**cyaneiventris** Macquart, 1846: 249 (*Aphritis*). OUMNH: ST* F. **[1a]** **Comb. n.**

= *cyanoventris* Williston, 1886: 310 (*Aphritis*). **Comb. n.**Misspelling.

**cyaneus** Perty, 1833: 186 (*Microdon*). ZSM*: A. **[2]** **Comb. n.**

**emeralda** Hull, 1943: 719 (*Microdon*). BMNH: HT* M. **[1a]** **Comb. n.**

**flavoluna** Hull, 1943: 718 (*Microdon*). BMNH: HT* M. **[1a]** **Comb. n.**

**histrio** Wiedemann, 1830: 83 (*Microdon*). ZMHU: T F. **[1a] Comb. n.**

**inaequalis** Loew, 1866: 40 (*Microdon*). MCZ (lost)*: T M. **[3: USNM] Comb. n.**

**instabilis** Wiedemann, 1830: 83 (*Microdon*). ZMHU: T F. **[1a]** **Comb. n.**

= *dives* Rondani, 1848: 72 (*Aphritis*). UTOR*: T M. **[2] Comb. n.**

**limbatus** Wiedemann, 1830: 85 (*Microdon*). ZMHU: T A. **[1a]** **Comb. n.**

**marceli** Curran, 1936: 7 (*Microdon*). AMNH: T M. **[1a]** **Comb. n.**

**nero** Curran, 1936: 6 (*Microdon*). AMNH: T M. **[1a]** **Comb. n.**

**nestor** Curran, 1940: 11 (*Microdon*). AMNH: T M. **[1a]** **Comb. n.**

**opulentus** Bigot, 1883: 319 (*Microdon*). BMNH*: HT* M*. **[1a]** **Comb. n.**

**pulcher** Williston, 1887: 5 (*Microdon*). USNM: LT* F. **[1a]** **Comb. n.**

**shannoni** Curran, 1940: 8 (*Microdon*). AMNH: T F. **[1a]** **Comb. n.**

**splendens** Wiedemann, 1830: 84 (*Microdon*). NMW: T M. **[3: USNM] Comb. n.**

**stramineus** Hull, 1943: 703 (*Microdon*). BMNH: HT* F. **[1a]** **Comb. n.**

**superbus** Wiedemann, 1830: 82 (*Microdon*). SMF: HT* F. **[1a]** **Comb. n.**

**tigrinus** Curran, 1940: 11 (*Microdon*). AMNH: T M. **[1a]** **Comb. n.**

**willistoni** Mik, 1899: 143 (*Microdon*). AMNH*: HT* M*. **[1a]** **Comb. n.** Replacement name for *Microdon inermis* Williston, 1888.

= *inermis* Williston, 1888: 258 (*Microdon*). AMNH: T M. **Comb. n.** Preocc. Loew, 1858.

**Oriental**

**aenoviridis** Curran, 1931: 302 (*Microdon*). BMNH: HT M. **[1a]** **Comb. n.**

**baramus** Curran, 1942: 3 (*Microdon*). AMNH*: HT F. **[1a] Comb. n.**

**beatus** Curran, 1942: 4 (*Microdon*). AMNH*: HT F*. **[1a] Comb. n.**

**latiscutellaris** Curran, 1931: 341 (*Microdon*). BMNH: HT F. **[1a]** **Comb. n.**

**lativentris** Meijere, 1921: 52 (*Microdon*). ZMAN: T M. **[1a]** **Comb. n.**

= *grandis* Curran, 1928: 159 (*Microdon*). BMNH: HT M. **[1a]** **Syn. n., comb. n.**

**lundura** Curran, 1942: 3 (*Microdon*). AMNH*: HT M. **[1a] Comb. n.**

**stilboides** Walker, 1849: 538 (*Microdon*). BMNH: HT* M*. **[1a]** **Comb. n.**

**Palaearctic**

**katsurai** Maruyama & Hironaga, 2004: 174 (*Microdon*). SEHU: HT M. **[2, 3: RMNH] Comb. n.**

**Subgenus *Dimeraspis* Newman, 1838**

**Nearctic**

**abditus** Thompson, 1981: 732 (*Microdon*). USNM: HT M. **[1a]** **Comb. n.**

**adventitius** Thompson, 1981: 735 (*Microdon*). USNM: HT M. **[1a]** **Comb. n.**

**fuscipennis** Macquart, 1834: 488 (*Ceratophya*). OUMNH: LT F. **[3: USNM] Comb. n.**

= *agapenor* Walker, 1849: 539 (*Microdon*). BMNH: ST F. **[1a]** **Comb. n.** See Thompson 1981.

= *pachystylum* Williston, 1887: 8 (*Microdon*). USNM: HT M. **[1a]** **Comb. n.** See Thompson 1981.

**globosus** Fabricius, 1805: 185 (*Mulio*). MNHN: LT F. **[1a]** **Comb. n.** Original type lost (see Zimsen 1964); lectotype designation by Thompson 1981b.

= *albipilis* Curran, 1925: 54 (*Microdon*). CNC: HT M. **[4: Thompson 1981b] Comb. n.**

= *conflictus* Curran, 1925: 58 (*Microdon*). CNC*: LT M. **[4: Thompson 1981b4] Comb. n.**

= *hutchingsi* Curran, 1927: 89 (*Microdon*). CNC: HT F. **[4: Thompson 1981b] Comb. n.**

= *marmoratus* Bigot, 1883: 320 (*Microdon*). BMNH*: ST* MF*. **[1a]** **Comb. n.**

= *podagra* Newman, 1838: 373 (*Dimeraspis*). BMNH: HT M. **[1a] Comb. n.**

= *pseudoglobosus* Curran, 1925: 57 (*Microdon*). SEMC*: HT* M*. **[4: Thompson 1981b] Comb. n.**

**Neotropical**

**remotus** Knab, 1917: 142 (*Microdon*). USNM: T M. **[4: Thompson 1981a]** **Comb. n.**

= *banksi* Hull, 1942: 91 (*Microdon*). MCZ: T F. **[4: Thompson 1981a] Comb. n.**

**Subgenus *Megodon* Keiser, 1971**

**Afrotropical**

**planitarsus** Keiser, 1971: 245 (*Microdon*). MNHN*: HT* M*. **[1a]**

**stuckenbergi** Keiser, 1971: 253 (*Megodon*). MNHN*: HT* M*. **[1a]** **Comb. n.**

**Subgenus *Microdon* Meigen, 1803 s.s.**

**Nearctic**

**abstrusus** Thompson, 1981: 735 (*Microdon*). USNM*: HT M. **[1a].** Paratype male in BMNH.

**albicomatus** Novak, 1977: 664 (*Microdon*). WSU: HT M*. **[3: ZMAN]**

**aurulentus** Fabricius, 1805: 185 (*Mulio*). MNHN: LT F. **[1a]**

**cothurnatus** Bigot, 1883: 320 (*Microdon*). BMNH*: HT* M*. **[1a]**

= *cockerelli* Jones, 1922: 17 (*Microdon*). USNM: ST M. **[4: Thompson 1981b]**

**lanceolatus** Adams, 1903: 222 (*Microdon*). SEMC: HT M*. **[3: USNM]**

= *coloradensis* Cockerell & Andrews, 1916: 53 (*Microdon*). USNM: HT M*. **[1a]**

= *modestus* Knab, 1917: 139 (*Microdon*). USNM: HT M*. **[1a]**

= *senilis* Knab, 1917: 139 (*Microdon*). USNM: HT F*. **[1a]**

= *similis* Jones, 1917: 219 (*Microdon*). USNM: LT F*. **[1a]** Described as var. of *Microdon cothurnatus* Bigot, 1883.

**manitobensis** Curran, 1924: 227 (*Microdon*). CNC: LT M*. **[1a]**

**megalogaster** Snow, 1892: 34 (*Microdon*). SEMC: HT M*. **[3: BMNH & RMNH]**

= *bombiformis* Townsend, 1895: 33 (*Microdon*). SEMC: HT F*. **[4: Thompson 1981b]**

**newcomeri** Mann, 1924: 94 (*Microdon*). USNM: HT M*. **[1a]**

**ocellaris** Curran, 1924: 227 (*Microdon*). USNM: LT F*. **[1a]**

**piperi** Knab, 1917: 136 (*Microdon*). USNM: LT M*. **[1a].** Holotype with empty puparium.

**ruficrus** Williston, 1887: 7 (*Microdon*). USNM: HT M*. **[1a]** Described as var. of *Microdon tristis* Loew, 1864.

= *basicornis* Curran, 1925: 79 (*Microdon*). CNC: HT M*. **[4: Thompson 1981b]**

= *champlaini* Curran, 1925: 71 (*Microdon*). USNM: HT M*. **[1a]**

**tristis** Loew, 1864: 73 (*Microdon*). MCZ (lost)*: T F*. **[3: CNC, RMNH, USNM]**

= *robusta* Telford, 1939: 14 (*Microdon*). UMSP: HT F*. **[4: Thompson 1981b]**

**xanthopilis** Townsend, 1895: 611 (*Microdon*). SEMC: LT M*. **[3: USNM]**

**Neotropical**

**aureopilis** Marinoni, 2004: 569 (*Microdon*). CNC: HT* M*. **[2]**

**barbouri** Hull, 1942: 89 (*Microdon*). MCZ: T F. **[1b]**

**bassleri** Curran, 1940: 10 (*Microdon*). AMNH: T F. **[1a]**

**bonariensis** Lynch Arribalzaga, 1891: 194 (*Microdon*). MACN: HT F. **[2]**

**brutus** Hull, 1944: 37 (*Microdon*). CU: T M. **[1a]**

**caesar** Curran, 1940: 10 (*Microdon*). AMNH: T M. **[1a]**

**crassitarsis** Macquart, 1848: 198 (*Aphritis*). OUMNH: HT* M. **[1a]**

**eutristis** Curran, 1925: 74 (*Microdon*). SEMC: T M. **[2]**

**macquartii** Lynch Arribalzaga, 1891: 126 (*Microdon*). OUMNH*: HT* F*. **[1a]** Replacement name for *Aphritis angustus* Macquart, 1848.

= *angustus* Macquart, 1848: 198 (*Aphritis*). OUMNH*: HT* F*. **[1a]** Preocc. Macquart, 1846.

= *angustatus* Fluke, 1957: 29 (*Microdon*). Misspelling of *Microdon angustatus* (Macquart, 1848).

**mourei** Marinoni, 2004: 569 (*Microdon*). CNC: HT* M*. **[2]**

**remus** Curran, 1941: 250 (*Microdon*). AMNH: T F. **[1a]**

**rufiventris** Rondani, 1848: 73 (*Aphritis*). MZUN: T F. **[3: BMNH & RMNH]**

**violaceus** Macquart, 1842: 13 (*Aphritis*). MNHN: ST* M*. **[1a]** The description by Macquart (1842) was based on a male from Chile, collected by M. Gay, which corresponds with the data on the label of a specimen in the Macquart collection of the MNHN. There is also a female in the same collection, but without a data label. There are also 12 specimens among the Macquart material in the OUMNH, but these too are without data labels (pers. comm. Z. Simmons).

**virgo** Curran, 1940: 7 (*Microdon*). AMNH: T M. **[1a]**

**Oriental**

**aeneus** Keiser, 1952: 172 (*Microdon*). NMB*: HT* M*. **[1a]**

**alboscutatus** Curran, 1931: 303 (*Microdon*). AMNH*: HT M*. **[1a]**. There is a specimen labelled as ‘holotype’ in the BMNH-collection, but locality information of that specimen is not correct. The real holotype is in AMNH.

**bellus** Brunetti, 1923: 315 (*Microdon*). BMNH: HT F. **[1a]**

**formosanus** Shiraki, 1930: 22 (*Microdon*). NIAS: ST* MF*. **[2]**

**fulvopubescens** Brunetti, 1923: 313 (*Microdon*). BMNH: HT F. **[1a]**

**fumipennis** Hull, 1944: 259 (*Microdon*). BMNH: HT M. **[1a]**

**metallicus** Meijere, 1904: 98 (*Microdon*). ZMAN: T M. **[1a]**

**sumatranus** Wulp, 1892*: 29 (*Microdon*). RMNH*: HT* F. **[1a]**. Publication year wrong in Knutson et al. 1975.

**sumbanus** Keiser, 1952: 174 (*Microdon*). NMB: HT F. **[1a]**

**Palaearctic**

**analis** Macquart, 1842: 72 (*Aphritis*). MNHN: HT* M*. **[1a]**

= ?*ammerlandia* Spix, 1824: 124 (*Scutelligera*). L. **[2] Syn. n.** Described as a mollusc.

= *brevicornis* Egger, 1862: 783 (*Microdon*). NMW: ST B. **[4: Doczkal and Schmid 1999]** Preocc. Loew, 1857.

= *eggeri* Mik, 1897: 66 (*Microdon*). NMW: T A. **[3: several coll.]** Replacement name for *Microdon brevicornis* Egger, 1862.

= *fuscitarsis* Schummel, 1842: 115 (*Microdon*). Lost*: T A **[4: Doczkal and Schmid 1999]**

= *latifrons* Loew, 1856: 599 (*Microdon*). Lost*: T A. **[4: Doczkal and Schmid 1999]**

= ?*reticulata* Torrez Minguez, 1924: 108 (*Buchanania*). HT* L*. **[2]** **Syn. n.** Described as a mollusc.

**auricomus** Coquillett, 1898: 320 (*Microdon*). USNM: HT* M*. **[1a]**

**devius** Linnaeus, 1761: 446 (*Musca*). Lost: T A. **[3: several coll.]**

= *anthinus* Meigen, 1822: 165 (*Microdon*). MNHN: HT M. **[4: Doczkal and Schmid 1999]**

= *conica* Panzer, 1793: 21 (*Stratiomys*). SNSD?: T A. *Musca devius* Linnaeus, 1761 **[4: Doczkal and Schmid 1999]**

= *micans* Wiedemann in Meigen, 1822: 165 (*Microdon*). MNHN: ST A **[4: Doczkal and Schmid 1999]**

= *picticornis* Mik, 1897: 66 (*Microdon*). A. **[4: Doczkal and Schmid 1999]** Described as var. of *Microdon devius* (Linnaeus, 1761).

= *pigra* Schrank, 1803: 97 (*Stratiomys*). A. **[4: Doczkal and Schmid 1999]**

= *viridescens* Villers, 1789: 463 (*Musca*). Coll. Villers: T A. **[4: Doczkal and Schmid 1999]**

**hauseri** Reemer sp. n. (*Microdon*). CSCS: HT M. **[1a] Sp. n.**

**ignotus** Violovitsh, 1976: 160 (*Microdon*). ZISP: HT M. **[2]**

**japonicus** Yano, 1915: 5 (*Microdon*). T A. **[3: BMNH & RMNH]**

= *jezoensis* Matsumura, 1916: 255 (*Microdon*). NIAS: ST F **[3: BMNH]**

**kidai** Hironaga & Maruyama, 2004: 90 (*Microdon*). SEHU: HT M. **[2]**

**lateus** Violovitsh, 1976: 160 (*Microdon*). A. **[4: Doczkal and Schmid 1999]**

**lehri** Mutin, 1999: 360 (*Microdon*). HT* M*. **[2]**

**macrocerus** Hironaga & Maruyama, 2004: 88 (*Microdon*). NSMT, Toyko: HT M. **[2]**

**major** Andries, 1912: 307 (*Microdon*). ZFMK*: NT* P*. **[3, 4: Schmid 2004]**

**mandarinus** Reemer sp. n. (*Microdon*). CSCS: HT M. **[1a] Sp. n.**

**maritimus** Violovitsh, 1976: 161 (*Microdon*). ZISP: HT M. **[3: USNM]**

**miki** Doczkal & Schmid, 1999: 48 (*Microdon*). SMNS*: HT* M*. **[2]** Paratypes in SMNS and ZMHU.

**murayamai** Hironaga & Maruyama, 2004: 97 (*Microdon*). SEHU: HT M. **[2]**

**mutabilis** Linnaeus, 1758: 592 (*Musca*). BMNH: LT F. [3: several coll.]

= *apiarius* Fabricius, 1805: 46 (*Mulio*). Lost: T A. **[4: Doczkal and Schmid 1999]** Type lost, only name label remains (Zimsen 1964).

= *apiformis* De Geer, 1776: 128 (*Musca*). NHRS: T A. **[4: Doczkal and Schmid 1999]**

= *auropubescens* Latreille, 1805: 358 (*Aphritis*). T A. **[4: Doczkal and Schmid 1999]**

= ?*cocciformis* von Heyden, 1825: 589 (*Parmula*). HT* L*. **[2]** Described as mollusc.

= *rhenanus* Andries, 1912: 307 (*Microdon*). ZFMK*: LT* P*. **[4: Schmid 2004]**

= *scutellatus* Schummel, 1842: 116 (*Microdon*). **[4: Doczkal and Schmid 1999]**

**myrmicae** Schönrogge, Barr, Wardlaw, Napper, Gardner, Breen, Elmes & Thomas, 2002: 315 (*Microdon*). BMNH: HT F. **[3: several coll.]**

**mysa** Violovitsh, 1971: 62 (*Microdon*). ZISP: HT M. **[2]**

**nigripes** Shiraki, 1930: 22 (*Microdon*). NIAS: ST MF. **[2]** Described as var. of *Microdon auricomus* Coquillett, 1898.

**nigrodorsatum** Mutin, 2011: 19 (*Microdon*). SEHU: HT M. **[2]**

**novus** Schrank, 1776: 93 (*Musca*). A. **[4: Peck 1988]**

**oitanus** Shiraki, 1930: 18 (*Microdon*). NIAS: HT F. **[2]**

**podomelainum** Huo, Ren & Zheng, 2007: 402 (*Microdon*). HU*: HT* F*. **[2]**

**ursitarsis** Stackelberg, 1926: 90 (*Microdon*). A. **[2]**

**yokohamai** Hironaga & Maruyama, 2004: 94 (*Microdon*). HUS: HT M. **[2]**

**yunnanensis** Reemer sp. n. (*Microdon*). CSCS: HT M. **[1a] Sp. n.**

**Subgenus *Myiacerapis* Hull, 1949**

**Afrotropical**

**villosus** Bezzi, 1915: 135 (*Microdon*). BMNH: HT* M*. **[1a]**

**Subgenus *Syrphipogon* Hull, 1937**

**Neotropical**

**fucatissimus** Hull, 1937: 120 (*Syrphipogon*). CM: HT M. **[1a]**

**gaigei** Steyskal, 1953: 1 (*Microdon*). MZM: HT F. **[2]**

**Species groups of *Microdon* s.l.**

***craigheadii*-group**

**Nearctic**

**craigheadii** Walton, 1912: 463 (*Microdon*). USNM: HT M. **[1a]**

***erythros*-group**

**Afrotropical**

**erythros** Bezzi, 1908: 382 (*Microdon*). T M*. **[3: RMCA & RMNH]**

= *erytherus* Bezzi, 1921: 21 (*Microdon*). M*. Misspelling.

**luteiventris** Bezzi, 1915: 132 (*Microdon*). BMNH: ST* MF*. **[1a]**

***mirabilis*-group**

**Neotropical**

**bertonii** Bezzi, 1910: 319 (*Microdon*). MCSN: ST* MF*. **[1a]**

= *arcuata* Curran, 1941: 250 (*Microdon*). AMNH: T M. **[1a]** **Syn. n.**

**iheringi** Bezzi, 1910: 320 (*Microdon*). MCSN: LT* M. **[1a]** For lectotype designation see Genus accounts.

**mirabilis** Williston, 1888: 257 (*Microdon*). AMNH: ST* MF*. **[1a]**

***tarsalis*-group**

**Afrotropical**

**tarsalis** Hervé-Bazin, 1913: 98 (*Microdon*). RMCA*: HT* M*. **[1a]** Holotype in RMCA, not in MNHN. See also Hervé-Bazin (1913: 69).

= *bequaerti* Curran, 1929: 3 (*Microdon*). AMNH: HT M. **[1a] Syn. n.** Paratype female in RMCA.

**Unplaced species of *Microdon* s.l.**

**Afrotropical**

**tsara** Keiser, 1971: 247 (*Microdon*). MNHN*: HT* M*. **[1a]**

**Australian / Oceanian**

**amabilis** Ferguson, 1926: 175 (*Microdon*). QMBA: T F. **[3: CNC]**

**macquariensis** Ferguson, 1926: 174 (*Microdon*). ANIC*: HT* M. **[1b, 3: USNM]**

**nigromarginalis** Curran & Bryan, 1926: 132 (*Microdon*). ANIC*: HT* F*. **[1b, 3: RMNH]**

**pictipennis** Macquart, 1850: 433 (*Aphritis*). MNHNP: HT* F. **[1a]**

= *pictulipennis* Hull, 1944: 249 (*Microdon*). BMNH: HT* M. **[1a]** **Syn. n.**

**rieki** Paramonov, 1957: 815 (*Microdon*). ANIC*: HT* M. **[1b, 1c: USNM]**

**waterhousei** Ferguson, 1926: 174 (*Microdon*). AMS: T F. **[3: coll. M. Hauser]**

**Oriental**

**carbonarius** Brunetti, 1923: 314 (*Microdon*). ZSI: HT M. **[1c]** Paratype and three additional specimens in BMNH.

**pagdeni** Curran, 1942: 6 (*Microdon*). AMNH*: HT F. **[1a]** Type not found in BMNH. Specimen labelled as such in AMNH.

**trimacula** Curran, 1928: 156 (*Microdon*). BMNH: ST* M. **[1a]**

**unicolor** Brunetti, 1915: 255 (*Microdon*). ZSI*: HT M. **[2]**

**Genus *Mixogaster* Macquart, 1842**

**Nearctic**

**breviventris** Kahl, 1897: 137 (*Mixogaster*). CU: F*. **[2]**

**delongi** Johnson, 1926: 301 (*Mixogaster*). MCZ: A. **[2]**

**johnsoni** Hull, 1941: 162 (*Mixogaster*). CNC: HT* A. **[2]**

**Neotropical**

**anthermus** Walker, 1849: 547 (*Ascia*). BMNH: ST* M*. **[1a]**

**cicatrix** Hull, 1954: 9 (*Mixogaster*). CU: T M. **[2]**

**conopsoides** Macquart, 1842: 14 (*Mixogaster*). MNHN: T F. **[1a]**

= *conopoides* Kertesz, 1910: 351 (*Mixogaster*). A. **[2]** Emendation.

=*conopseus* Williston, 1886: 309 (*Mixogaster*). A. **[2]** Misspelling.

**cubensis** Curran, 1932: 1 (*Mixogaster*). AMNH: T M. **[1a]**

**currani** Hull, 1954: 5 (*Mixogaster*). AMNH: T M. **[1a]**

**dimidiata** Giglio-Tos, 1892: 1 (*Mixogaster*). MRSN: HT F. **[2]**

= *dimitiata* Fluke, 1957: 37 (*Mixogaster*). A. **[2]** Misspelling.

**flukei** Hull, 1954: 15 (*Mixogaster*). AMNH: T M. **[1a]**

**imitator** Thompson, 2004: 572 (*Mixogaster*). USNM: HT* M*. **[1c: BMNH]**

**lanei** Carrera & Lenko, 1958: 473 (*Mixogaster*). MZUSP: T M. **[2]**

**lopesi** Carrera & Lenko, 1958: 477 (*Mixogaster*). MZUSP: T M. **[2]**

**mexicana** Macquart, 1846: 251 (*Mixogaster*). MRHNB: T F*. **[2]**

**orpheus** Hull, 1944: 36 (*Mixogaster*). MCZ: T F. **[2]**

**pithecofascia** Hull, 1944: 512 (*Mixogaster*). CNC: HT M. **[2]**

**polistes** Hull, 1954: 4 (*Mixogaster*). AMNH: T F. **[1a]**

**rarior** Shannon, 1925: 111 (*Mixogaster*). USNM: T M. **[1a]**

= *rarissima* (var.) Shannon, 1925: 111 (*Mixogaster*). USNM*: M*. **[1a]** Described as var. of *Mixogaster rarior* Shannon, 1925.

**sartocrypta** Hull, 1954: 8 (*Mixogaster*). AMNH: T F. **[1a]**

**strictor** Hull, 1941: 1 (*Mixogaster*). AMNH: T M. **[1a]**

**thecla** Hull, 1954: 6 (*Mixogaster*). AMNH: T F. **[1a]**

**Genus *Oligeriops* Hull, 1937 stat. n.**

**Australian / Oceanian**

**chalybeus** Ferguson, 1926: 176 (*Microdon*). Coll. Hardy: T M. **[2]** **Comb. n.**

**dimorphon** Ferguson, 1926: 177 (*Microdon*). ANIC*: HT* A. **[1b]** **Comb. n.**

**iridomyrmex** Shannon, 1927: 85 (*Microdon*). BMNH: ST* F. **[1a]** **Comb. n.**

**moestus** Ferguson, 1926: 518 (*Microdon*). ANIC: T F. **[2]** **Comb. n.**

**occidentalis** Ferguson, 1926: 176 (*Microdon*). SAMA: T F. **[2]** **Comb. n.**

**Genus *Omegasyrphus* Giglio-Tos, 1891**

**Nearctic**

**baliopterus** Loew, 1872: 86 (*Microdon*). MCZ (lost)*: ST MF*. **[3: USNM]**

= *brunnipennis* Hull, 1944: 400 (*Microdon*). NMW: HT M. **[2]** Described as var. of *Microdon baliopterus* Loew, 1872.

**coarctatus** Loew, 1864: 74 (*Microdon*). MCZ (lost)*: ST MF*. **[3: USNM]**

**gracilis** Bigot, 1883: 320 (*Microdon*). BMNH*: HT* M*. **[1a]** **Comb. n.**

**painteri** Hull, 1922: 370 (*Microdon*). CNC: HT M*. **[1b]**

**pallipennis** Curran, 1925: 89 (*Microdon*). SEMC: ST A. **[3: USNM]**

**Genus *Paragodon* Thompson, 1969**

**Neotropical**

**paragoides** Thompson, 1969: 81 (*Paragodon*). CNC: HT M. **[2]**

**Genus *Paramicrodon* Meijere, 1913**

**Australian / Oceanian**

**lorentzi** Meijere, 1913: 360 (*Paramicrodon*). ZMAN: T F. **[1a]**

**toxopei** Meijere, 1929: 410 (*Paramicrodon*). ZMAN: T M. **[1a]**

**Neotropical**

**delicatulus** Hull, 1937: 24 (*Paramicrodon*). MCZ: T M. **[1a]**

**flukei** Curran, 1936: 2 (*Paramicrodon*). AMNH: T M. **[1a]**

**Oriental**

**cinctellus** Sack, 1926: 590 (*Myxogaster*). DEI?*: T F. **[2]**

**miranda** Herve-Bazin, 1926: 74 (*Syrphinella*). MNHN*: HT* F*. **[1a]**

**nigripennis** Sack, 1922: 275 (*Myxogaster*). T M. **[2]**

**novus** Hull, 1937: 22 (*Paramicrodon*). MCZ: T F. **[2]**

**Genus *Paramixogaster* Brunetti, 1923**

**Afrotropical**

**acantholepidis** Speiser, 1913: 141 (*Microdon*). NMSA*: HT* M*. **[1a]** **Comb. n.**

**crematogastri** Speiser, 1913: 143 (*Microdon*). NMSA*: HT* F*. **[1a]** **Comb. n.**

**elisabethae** Keiser, 1971: 254 (*Pseudomicrodon*). MNHN*: T F*. **[1a]** **Comb. n.**

**illucens** Bezzi, 1915: 121 (*Microdon*). BMNH: T M*. **[1a]** **Comb. n.**

**piptotus** Reemer sp. n. (*Paramixogaster*). MNHN: HT M. **[1a] Sp. n.**

**Australian / Oceanian**

**aphritinus** Thomson, 1869: 491 (*Mixogaster*). NHRS: HT* M*. **[1a]** **Stat. n., comb. n.**

**daveyi** Knab & Malloch, 1912: 233 (*Microdon*). USNM: T F. **[1a]** **Comb. n.**

**gayi** Paramonov, 1957: 814 (*Microdon*). ANIC*: HT* F. **[1b]** **Comb. n.**

**odyneroides** Meijere, 1908: 213 (*Microdon*). HNHM (lost)*: T A. **[2, 4: Sack 1926]** **Comb. n.**Type lost (de Jong 2000).

**omeanus** Paramonov, 1957: 813 (*Microdon*). ANIC: HT* F. **[1b, 1c: USNM]** **Comb. n.**

**petiolata** Hull, 1944: 248 (*Microdon*). BMNH: HT* F. **[1a]** **Comb. n.**

**praetermissus** Ferguson, 1926: 182 (*Microdon*). SAMA?: T F. **[2]** **Comb. n.**

**variegatus** Walker, 1852: 220 (*Ceratophya*). BMNH*: F*. **[1a]** **Comb. n.**

**wegneri** Keiser, 1964: 84 (*Paramixogaster*). NMB Basel*: HT* M*. **[2]**

**Oriental**

**brunettii** Reemer, sp. n. **Nom. n.** New replacement name for *Mixogaster vespiformis* Brunetti, 1913.

= *vespiformis* Brunetti, 1913: 169 (*Mixogaster*). ZSI: HT M*. **[2]** Preocc. De Meijere, 1908.

**contractus** Brunetti, 1923: 310 (*Microdon*). BMNH: HT F. **[1a]** **Comb. n.**

**conveniens** Brunetti, 1923: 311 (*Microdon*). BMNH: HT F. **[1a]** **Comb. n.**

**decipiens** Meijere, 1917: 242 (*Paramicrodon*). ZMAN*: HT* F*. **[1a]** **Comb. n.** Puparia also in ZMAN.

**fujianensis** Cheng, 695 (*Paramixogaster*). CASB: HT M. **[2]**

**icariiformis** Pendlebury, 1927: 38 (*Paramixogaster*). BMNH (lost)*: T F. **[2]** Type not in BMNH.

**indicus** Doleschall, 1857: 404 (*Ceratophya*). HNHM (lost)*: T A. **[2]** **Comb. n.**

**luxor** Curran, 1931: 306 (*Microdon*). BMNH: HT M. **[1a]** **Comb. n.**

**sacki** **Nom. n., comb. n.** New replacement name for *Myxogaster variegata* Sack, 1922.

= *variegata* Sack, 1922: 274 (*Myxogaster*). F*. **[2] Comb. n.** Preocc. Walker, 1852.

**vespiformis** Meijere, 1908: 210 (*Microdon*). ZMAN*: ST* F*. **[1a]** **Comb. n.**

**wegneri** Keiser, 1964: 84 (*Paramixogaster*). NMB Basel*: HT* F*. **[2]**

**yunnanensis** Cheng, 2012: 696 (*Paramixogaster*). CASB: HT M. **[2]**

**Genus *Parocyptamus* Shiraki, 1930**

**Oriental**

**sonamii** Shiraki, 1930: 12 (*Parocyptamus*). NIAS*: ST M. **[1a]**

= *purpureus* Hull, 1937: 26 (*Stenomicrodon*). CNC*: HT F. **[1b]** **Syn. n.**

**stenogaster** Curran, 1931: 344 (*Microdon*). BMNH: HT M*. **[1a]** **Comb. n.**

**Genus *Peradon* Reemer gen. n.**

**Neotropical**

***bidens*-group**

**angustiventris** Macquart, 1855: 105 (*Aphritis*). OUMNH: HT* M. **[1a]** **Comb. n.**

**angustus** Macquart, 1846: 250 (*Aphritis*). MNHN (lost)*: T M. **[2]** **Comb. n.** Type not found in MNHN.

**aurifascia** Hull, 1944: 245 (*Microdon*). BMNH: HT* M. **[1a]** **Comb. n.**

**bidens** Fabricius, 1805: 185 (*Mulio*). UZMC: HT* M. **[1a]** **Comb. n.**

=*bicolor* Walker, 1857: 151 (*Ceratophya*). BMNH: HT* F*. **[1a]** **Syn. n., comb. n.**

**bispina** Hull, 1943: 707 (*Microdon*). BMNH: HT* M. **[1a]** **Comb. n.**

**elongata** Hull, 1943: 706 (*Microdon*). BMNH: HT M. **[1a]** **Comb. n.**

**flavipennis** Curran, 1925: 342 (*Microdon*). MCZ: T F. **[1b]** **Comb. n.**

**flavomarginatum** Curran, 1925: 245 (*Microdon*). CU: T M. **[1a]** **Comb. n.**

**langi** Curran, 1925: 341 (*Microdon*). AMNH*: T M. **[1a] Comb. n.**

**luridescens** Walker, 1857: 151 (*Ceratophya*). BMNH: T F. **[1a]** **Comb. n.**

**niger** Williston, 1891: 4 (*Microdon*). BMNH: HT* M. **[1a]** **Comb. n.**

=*manni* Shannon, 1923: 80 (*Microdon*). USNM: T F. **[1a] Comb. n.**

**normalis** Curran, 1925: 343 (*Microdon*). AMNH: T F. **[1a]** **Comb. n.**

**oligonax** Hull, 1944: 35 (*Microdon*). CU: T F. **[1a]** **Comb. n.**

***flavofascium*-group**

**aurigaster** Hull, 1941: 160 (*Microdon*). MCZ: T M. **[1b]** **Comb. n.**

**chrysopygus** Giglio-Tos, 1892: 1 (*Ubristes*). MRSN*: HT* F*. **[1b]** **Comb. n.**

**flavofascium** Curran, 1925: 346 (*Microdon*). CU: T M. **[1a]** **Comb. n.**

***trivittatus*-group**

**aureoscutus** Hull, 1943: 709 (*Microdon*). BMNH: HT* M. **[1a]** **Comb. n.**

**aureus** Hull, 1944: 35 (*Microdon*). MCZ: T F. **[1b]** **Comb. n.**

**diaphanus** Sack, 1921: 146 (*Microdon*). DEI: T M. **[3: USNM] Comb. n.**

**fenestratus** Hull, 1943: 712 (*Microdon*). BMNH: HT* M. **[1a]** **Comb. n.**

**hermetia** Curran, 1936: 3 (*Microdon*). AMNH: HT* M. **[1a]** **Comb. n.**

**hermetoides** Curran, 1940: 8 (*Microdon*). BMNH: HT* M. **[1a]** **Comb. n.**

**trilinea** Hull, 1943: 710 (*Microdon*). BMNH: HT* M. **[1a]** **Comb. n.**

**trivittatus** Curran, 1925: 344 (*Microdon*). AMNH: T M. **[1a]** **Comb. n.**

**Genus *Piruwa* Reemer gen. n.**

**Neotropical**

**phaecada** Reemer sp. n. (*Piruwa*). RMNH: HT M. **[1a] Sp. n.**

**Genus *Pseudomicrodon* Hull, 1937**

**Neotropical**

**auricinctus** Sack, 1931: 148 (*Rhopalosyrphus*). DEI*: F*. **[3: RMNH & USNM]** **Comb. n.**

**batesi** Shannon, 1927: 22 (*Microdon*). BMNH: HT* F. **[1a]** **Comb. n.**

**beebei** Curran, 1936: 4 (*Microdon*). AMNH: T F. **[1a]**

**bellulus** Williston, 1891: 1 (*Mixogaster*). BMNH: HT* M. **[1a]**

**biluminiferus** Hull, 1944: 399 (*Microdon*). NMW: T M. **[1a]** **Comb. n.**

**chrysostypus** Thompson, 2004: 571 (*Microdon*). USNM: HT* M*. **[2]** **Comb. n.**

**claripennis** Hine, 1914: 334 (*Mixogaster*). OHSU: T M. **[2]** **Comb. n.**

**conops** Curran, 1940: 4 (*Microdon*). AMNH: T M. **[1a]** **Comb. n.**

**corona** Curran, 1940: 9 (*Microdon*). AMNH: T M. **[1a]** **Comb. n.**

**nigrispinosus** Shannon, 1927: 21 (*Microdon*). BMNH: ST* M. **[1a]**

**pilosops** Marinoni, 2004: 572 (*Microdon*). BMNH: HT* M*. **[1a]**

**polistoides** Reemer sp. n. (*Pseudomicrodon*). RMNH: HT F. **[1a] Sp. n.**

**rheochryssus** Hull, 1944: 38 (*Microdon*). CU: T M. **[2]** **Comb. n.**

**seabrai** Papavero, 1962: 317 (*Pseudomicrodon*). Seabra*: T M. **[2]**

**smiti** Reemer sp. n. (*Pseudomicrodon*). RMNH: HT M. **[1a] Sp. n.**

**Genus *Ptilobactrum* Bezzi, 1915**

**Afrotropical**

**neavei** Bezzi, 1915: 137 (*Ptilobactrum*). BMNH: HT* M*. **[1a]**

**Genus *Rhoga* Walker, 1857**

**Neotropical**

**lutescens** Walker, 1857: 157 (*Rhoga*). BMNH (lost)*: T F. **[2]** Type not present in BMNH, probably lost.

**maculata** Shannon, 1927: 21 (*Microdon*). BMNH: HT F*. **[1a]**

**mellea** Curran, 1940: 5 (*Microdon*). BMNH: T M. **[1a]**

**sepulchrasilva** Hull, 1937: 28 (*Papiliomyia*). NMW: T M. **[1a]**

**xanthoprosopa** Barretto & Lane, 1947: 145 (*Rhoga*). MZUSP: HT M. **[2]**

**Genus *Rhopalosyrphus* Giglio-Tos, 1891**

**Sensu stricto**

**Nearctic**

**ramulorum** Weems & Deyrup, 2003: 189 (*Rhopalosyrphus*). USNM: HT M. **[1c: BMNH]**

[*Rhopalosyrphus guentherii*, a mainly Neotropical species, is also known from southern states of the USA. However, it is only listed among the Neotropical species below so as to avoid species being mentioned more than once.]

**Neotropical**

**australis** Thompson, 2003: 188 (*Rhopalosyrphus*). AMNH: HT F. **[1c: BMNH]**

**ecuadoriensis** Reemer sp. n. (*Rhopalosyrphus*). RMNH: HT M. **[1a] Sp. n.**

**guentherii** Lynch Arribalzaga, 1891: 195 (*Holmbergia*). MACN: T A. **[3: RMNH & USNM]**

= *carolae* Capelle, 1956: 174 (*Rhopalosyrphus*). SEMC: HT F. **[4: Weems et al. 2003]**

**robustus** Reemer sp. n. (*Rhopalosyrphus*). CNC: HT M. **[1a] Sp. n.**

**Sensu lato (Neotropical)**

**abnormis** Curran, 1925: 345 (*Microdon*). MCZ: HT* F. **[1b]** **Comb. n.**

**abnormoides** Reemer sp. n. (*Rhopalosyrphus*). RMNH: HT M. **[1a] Sp. n.**

**cerioides** Hull, 1943: 716 (*Microdon*). BMNH: T M. **[1a]** **Comb. n.**

**oreokawensis** Reemer sp. n. (*Rhopalosyrphus*). RMNH: HT F. **[1a] Sp. n.**

**Genus *Schizoceratomyia* Carrera, Lopes & Lane, 1947**

**Neotropical**

**barretoi** Carrera, Lopes & Lane, 1947: 245 (*Schizoceratomyia*). MZUSP*: M*. **[1a]**

**carrerai** Papavero, 1962: 324 (*Masarygus*). MZUSP: HT M. **[1a]**

**flavipes** Carrera, Lopes & Lane, 1947: 247 (*Schizoceratomyia*). MZUSP: HT M. **[1a]**

**malleri** Curran, 1947: 1 (*Johnsoniodon*). AMNH: HT M*. **[1a]**

**Genus *Serichlamys* Curran, 1925 stat. n.**

**Nearctic**

**?diversipilosus** Curran, 1925: 76 (*Microdon*). SEMC: HT M. **[2]** **Comb. n.**

**rufipes** Macquart, 1842: 71 (*Aphritis*). OUMNH: HT* M*. **[1a]**

= *limbus* Williston, 1887: 8 (*Microdon*). USNM: HT F*. **[4: Thompson 1981b]**

**scutifer** Knab, 1917: 141 (*Microdon*). USNM: HT F*. **[3: RMNH & USNM] Comb. n.**

**Neotropical**

**mitis** Curran, 1940: 7 (*Microdon*). AMNH: T M. **[1a]** **Comb. n.**

**mus** Curran, 1936: 5 (*Microdon*). AMNH: T M. **[1a]** **Comb. n.**

**Genus *Spheginobaccha* de Meijere, 1908**

**Afrotropical**

**dexioides** Hull, 1944: 131 (*Spheginobaccha*). BMNH*: M*. **[1a]**

**dubia** Thompson, 1974: 280 (*Spheginobaccha*). NMSA*: HT* M*. **[2]**

**guttula** Dirickx, 1995: 155 (*Spheginobaccha*). MNHN*: M*. **[1a]**

**perialla** Thompson, 1974: 284 (*Spheginobaccha*). BMNH*: HT* M*. **[2]**

**rotundiceps** Loew, 1858: 376 (*Ocyptamus*). NHRS*: HT* F*. **[4: Thompson 1974]**

= *funeralis* Hull, 1944: 131 (*Spheginobaccha*). BMNH*: HT* M*. **[4: Thompson 1974]**

**ruginosa** Dirickx, 1995: 152 (*Spheginobaccha*). MNHN*: F*. **[1a]**

**Oriental**

**aethusa** Walker, 1849: 559 (*Xylota*). BMNH*: HT* F*. **[4: Thompson 1974]**

**chillcotti** Thompson, 1974: 274 (*Spheginobaccha*). CNC*: HT* M*. **[2]**

**demeijerei** Doesburg, 1968: 161 (*Spheginobaccha*). RMNH*: HT* M*. **[1a]**

**duplex** Walker, 1857: 18 (*Syrphus*). BMNH*: HT* M*. **[4: Thompson 1974]**

**humeralis** Sack, 1926: 571 (*Doros*). USNM*: LT* M*. **[1a]**

**knutsoni** Thompson, 1974: 271 (*Spheginobaccha*). USNM*: HT* F*. **[2]**

**lieftincki** Doesburg, 1968: 160 (*Spheginobaccha*). RMNH*: HT* M*. **[1a]**

**macropoda** Bigot, 1883: 331 (*Sphegina*). BMNH*: F*. **[4: Thompson 1974]**

= *robusta* Brunetti, 1907: 11 (*Baccha*). ZSI*: A. **[4: Thompson 1974]**

**melancholica** Hull, 1937: 174 (*Spheginobaccha*). USNM: F*. **[4: Thompson 1974]**

**vandoesburgi** Thompson, 1974: 273 (*Spheginobaccha*). BMNH*: HT* M*. **[2]**

**Genus *Stipomorpha* Hull, 1945 stat. n.**

**Neotropical**

**apicula** Curran, 1930: 5 (*Microdon*). AMNH: HT M. **[1a]** **Comb. n.**

**fraudator** Shannon, 1927: 20 (*Microdon*). BMNH: T M. **[1a]** **Comb. n.**

**goettei** Shannon, 1927: 19 (*Microdon*). BMNH: T F. **[1a]** **Comb. n.**

**guianica** Curran, 1925: 340 (*Microdon*). MCZ: T F. **[1a]** **Comb. n.**

**inarmata** Curran, 1925: 5 (*Microdon*). MCZ: T M. **[1a]** **Comb. n.**

**lacteipennis** Shannon, 1927: 18 (*Microdon*). BMNH: T M. **[1a]** **Comb. n.**

= *triangularis* Curran, 1940: 6 (*Microdon*). AMNH: T M. **[1a]** **Syn. n., comb. n.**

**lanei** Curran, 1936: 5 (*Microdon*). AMNH: HT F. **[1a]** **Comb. n.**

**litoralis** Papavero, 1964: 21 (*Ubristes*). MZUSP: T M. **[1a] Comb. n.**

**mackiei** Curran, 1940: 5 (*Microdon*). AMNH: HT F. **[1a]** **Comb. n.**

**micromidas** Shannon, 1925: 112 (*Microdon*). USNM: HT F. **[1a]** **Comb. n.**

**mixta** Curran, 1940: 6 (*Microdon*). BMNH: T F. **[1a]** **Comb. n.**

**puerilis** Doesburg, 1966: 86 (*Ubristes*). RMNH*: T F. **[1a]** **Comb. n.**

**simillima** Hull, 1950: 611 (*Microdon*). BMNH: T M. **[1a]** **Comb. n.**

**tenuicauda** Curran, 1925: 339 (*Microdon*). CU: T F. **[1a]** **Comb. n.**

**trigoniformis** Shannon, 1927: 19 (*Microdon*). BMNH: T M. **[1a]** **Comb. n.**

**wheeleri** Mann, 1928: 168 (*Microdon*). USNM: T M. **[1a]** **Comb. n.**

[Descriptions of nine additional species from the Neotropical region are in preparation by the first author.]

**Genus *Sulcodon* Reemer gen. n.**

**Oriental**

**sulcatus** Hull, 1944: 256 (*Microdon*). BMNH: HT F. **[1a]** **Comb. n.**

**Genus *Surimyia* Reemer, 2008**

**Neotropical**

**minutula** Doesburg, 1966: 89 (*Ceratophya*). RMNH: HT* M*. **[1a]**

**rolanderi** Reemer, 2008: 180 (*Surimyia*). RMNH*: HT* M*. **[1a]**

**Genus *Thompsodon* Reemer gen. n.**

**Neotropical**

**conspicillifrons** Reemer sp. n. (*Thompsodon*). INBIO: HT F. **[1a] Sp. n.**

**Genus *Ubristes* Walker, 1852**

**Neotropical**

**flavitibia** Walker, 1852: 217 (*Ubristes*). BMNH: T M. **[1a]**

= *procteri* Curran, 1941: 251 (*Microdon*). AMNH: T M. **[1a]** **Syn. n.**

= *procedens* Curran, 1941: 251 (*Microdon*). AMNH: T M. **[1a]** **Syn. n.**

[Descriptions of two additional species from the Neotropical region are in preparation by the first author.]

**Unplaced Microdontinae**

**Afrotropical**

**schultzei** Simroth, 1907: 796 (*Ceratoconcha*). T A. Described as mollusc. Only known from larva. Preocc. Kramberger-Gorjanovic, 1889.

**Australian / Oceanian**

**hardyi** Ferguson, 1926: 171 (*Microdon*). Coll. Hardy: T M. **[2]**

**obscurus** Wulp, 1898: 421 (*Microdon*). HNHM (lost)*: T F. **[2]** Type lost. Van der Wulp (1898) states that the type was a female, but this is doubtful, considering his description of the head.

**sharpii** Mik, 1900: 148 (*Microdon*). BMNH: HT* F*. **[1a]** Replacement name for *Microdon pictipennis* Sharp, 1899.

= *pictipennis* Sharp, 1899: 390 (*Microdon*). BMNH: HT* A. Preocc. Macquart, 1850.

**Neotropical**

**aeolidiformis** Wheeler, 1924: 239 (*Microdon*). USNM: HT* A. **[2]** Described from larva.

**aztecarum** Wheeler, 1924: 243 (*Nothomicrodon*). USNM: T L*. **[1a]** Described from larva. Uncertain whether it belongs to Syrphidae.

**bruesi** Hull, 1945: 77 (*Microdon*). MCZ: HT* F. **[1b]**

**ignobilis** Rondani, 1848: 73 (*Aphritis*). MZUN: ST MF. **[2]**

**longicornis** Wiedemann, 1824: 14 (*Ceratophya*). NMW (lost)*: T F. **[2]** Type not in NMW (pers. comm. P. Sehnal).

**pauper** Rondani, 1848: 74 (*Aphritis*). MZUN: T M. **[2]**

**rubriventris** Lynch Arribalzaga, 1891: 128 (*Microdon*). MACN: T A. **[2]**

**viridis** Townsend, 1895: 610 (*Microdon*). Lost*: HT F*. **[4: Thompson 1981b]** See Thompson (1981b).

**Oriental**

**apidiformis** Brunetti, 1925: 78 (*Microdon*). ZSI*: M*. **[2]** Replacement name for *Microdon apiformis* Brunetti, 1923.

= *apiformis* Brunetti, 1923: 314 (*Microdon*). ZSI: HT M. Preocc. De Geer, 1776.

**dimidiatus** Curran, 1942: 3 (*Microdon*). BMNH (lost)*: HT M. **[2]** Type not found in AMNH and BMNH.

**laxiceps** Curran, 1942: 2 (*Microdon*). BMNH (lost)*: HT F. **[2]** Type not found in AMNH and BMNH.

**shirakii** **Nom. n.** New replacement name for *Microdon tuberculatus* Shiraki, 1968. See notes under Genus account of *Kryptopyga*.

= *tuberculatus* Shiraki, 1968: 11 (*Microdon*). NIAS: HT F. **[1a]** Preocc. de Meijere, 1913.

**trigonospilus** Bezzi, 1927: 4 (*Microdon*). MCSN*: HT* F*. **[1a]**

**Taxa previously considered to belong to Microdontinae**

**Afrotropical**

**varius** Walker, 1849: 540 (*Microdon*). F*. **[4: Thompson 2010]** Species of *Graptomyza* (Syrphidae: Eristalinae).

**Neotropical**

**grandis** Lynch Arribalzaga, 1892: 255 (*Argentinomyia*). MACN*: A. **[4]** Species of *Argentinomyia* (Syrphidae: Syrphinae)

**testaceipes** Lynch Arribalzaga, 1891: 199 (*Argentinomyia*). MACN*: A. **[4]** Species of *Argentinomyia* (Syrphidae: Syrphinae)

**Palaearctic**

**apiarius** Fabricius, 1781: 422 (*Syrphus*). A **[4: Thompson et al. 1982]** Synonym of *Mesembrina mystacea* (Linneaus) (Muscidae). Wrongly listed by Peck (1988) as synonym of *Microdon mutabilis* (Linnaeus).

**sophianus** Drensky, 1934: 122 (*Microdon*). Lost?: T F. **[4: Doczkal and Schmid 1999]** Probably a species of *Chrysotoxum* Meigen (Syrphidae).
